# The PhenoGen Informatics website: tools for analyses of complex traits

**DOI:** 10.1186/1471-2156-8-59

**Published:** 2007-08-30

**Authors:** Sanjiv V Bhave, Cheryl Hornbaker, Tzu L Phang, Laura Saba, Razvan Lapadat, Katherina Kechris, Jeanette Gaydos, Daniel McGoldrick, Andrew Dolbey, Sonia Leach, Brian Soriano, Allison Ellington, Eric Ellington, Kendra Jones, Jonathan Mangion, John K Belknap, Robert W Williams, Lawrence E Hunter, Paula L Hoffman, Boris Tabakoff

**Affiliations:** 1 Department of Pharmacology, University of Colorado at Denver and Health Sciences Center, Aurora, CO 80045, USA; 2 Department of Preventive Medicine and Biometrics, University of Colorado at Denver and Health Sciences Center, Aurora, CO 80045, USA; 3 MRC Clinical Sciences Centre, Faculty of Medicine, Imperial College, London W12 0NN, UK; 4 US Department of Veterans Affairs Medical Center, Portland, Oregon 97239, USA; 5 Department of Anatomy and Neurobiology, University of Tennessee Health Science Center, 855 Monroe Avenue, Memphis, TN 38163, USA

## Abstract

**Background:**

With the advent of "omics" (e.g. genomics, transcriptomics, proteomics and phenomics), studies can produce enormous amounts of data. Managing this diverse data and integrating with other biological data are major challenges for the bioinformatics community. Comprehensive new tools are needed to store, integrate and analyze the data efficiently.

**Description:**

The PhenoGen Informatics website  is a comprehensive toolbox for storing, analyzing and integrating microarray data and related genotype and phenotype data. The site is particularly suited for combining QTL and microarray data to search for "candidate" genes contributing to complex traits. In addition, the site allows, if desired by the investigators, sharing of the data. Investigators can conduct "*in-silico*" microarray experiments using their own and/or "shared" data.

**Conclusion:**

The PhenoGen website provides access to tools that can be used for high-throughput data storage, analyses and interpretation of the results. Some of the advantages of the architecture of the website are that, in the future, the present set of tools can be adapted for the analyses of any type of high-throughput "omics" data, and that access to new tools, available in the public domain or developed at PhenoGen, can be easily provided.

## Background

The need for data sharing in the context of "omics" data has been underscored in a number of recent articles [1,2]. Funding agencies have also emphasized the need and expectation of data sharing among scientists [3]. In particular, the National Institutes of Health (NIH) implemented a data sharing policy that requires that any applications for funding of $500,000 or more to specifically indicate a plan for data sharing. Most "omics" studies reach well beyond this fiscal threshold.

The magnitude and complexity of "omics" data have fueled interactions and cooperation among multiple investigators and institutions. These interactions have led to the formation of a number of microarray-based gene expression databases [4-12], both within public and commercial domains, and the development of associated tools necessary for high-throughput data analysis (see Table 1). Some of these databases also aid in understanding the biological inferences of the results (Table 1). The PhenoGen toolbox was originally created to facilitate interactions within the INIA consortium of investigators. In brief, the goals and purpose of the **INIA **(**I**ntegrative **N**euroscience **I**nitiative on **A**lcoholism) consortium are to identify the molecular, cellular, and behavioral neuroadaptations that occur in the brain reward circuits associated with the extended amygdala and its connections as a result of exposure to ethanol. Although PhenoGen web tools were initially created for the consortium members, the integrated tools described here are now accessible to the global scientific community.

**Table 1:** Comparison of microarray databases (and associated tools) in public domain

**Functionality**	**Array Express**	**GEO**	**GeneNetwork/WebQTL**	**Stanford Microarray database**	**BASE**	**PhenoGen**
**Data warehouse (MIAME compliant)**	**Yes**	**Yes**	No	**Yes**	**Yes**	**Yes**
**Microarray platform support**						
Oligonucleotide	**Yes**	**Yes**	**Yes**	**Yes**	No	**Yes**
cDNA	**Yes**	**Yes**	**Yes**	**Yes**	**Yes**	**Yes**
**Microarray data search**						
Gene based	**Yes**	**Yes**	**Yes**	**Yes**	**Yes**	No
Hybridization (array) based	**Yes**	**Yes**	No	**Yes**	**Yes**	**Yes**
Expt based	**Yes**	**Yes**	**Yes**	**Yes**	**Yes**	**Yes**
Sample attributes	**Yes**	**Yes**	No	**Yes**	**Yes**	**Yes**
**Microarray data retrieval**						
Gene based	**Yes**	**Yes**	**Yes**	**Yes**	**Yes**	**Yes**
Hybridization (array) based	**Yes**	**Yes**	No	**Yes**	**Yes**	**Yes**
Expt based	**Yes**	**Yes**	No	**Yes**	**Yes**	**Yes**
**Microarray data analysis**						
QC	No	No	No	**Yes**	No	**Yes**
Normalization	**Yes**	No	**Yes**	**Yes**	**Yes**	**Yes**
Filtering	**Yes**	No	No	**Yes**	**Yes**	**Yes**
**"*in-silico analysis*"**						
Data sharing	**Yes**	No	?	**Yes**	**Yes**	**Yes**
"in-silico experiments"	**Yes**	No	No	?	?	**Yes**
**Statistics**			"Different options"		"Plug-Ins"	
Basic stats	**Yes**	No	**Yes**	**Yes**	?	**Yes**
ANOVA	**Yes**	No	No	**Yes**	?	**Yes**
Clustering	**Yes**	No	**Yes**	**Yes**	?	No
Correlation	No	No	**Yes**	No	?	**Yes**
**Gene lists**						
Annotations (Dynamic)	No	No	**Yes**	**Yes**	No	**Yes**
Comparisons	No	No	**Yes**	?	No	**Yes**
eQTL	No	No	**Yes**	No	No	**Yes**
pQTL – Gene location overlapp	No	No	No	No	No	**Yes**
pQTL – eQTL overlapp	No	No	No	No	No	**Yes**
Promoter analysis	No	No	No	No	No	**Yes**
Literature search	No	No	No	No	No	**Yes**
**System requirements**						
Local	**Yes**	No	No	**Yes**	**Yes**	No
Web-based	**Yes**	**Yes**	**Yes**	**Yes**	**Yes**	**Yes**
**LIMS**						
Built-in	**Yes**	No	No	**Yes**	**Yes**	No
**Programming expertise needed**						
Expert				**Yes**	**Yes**	
Novice	**Yes**			**Yes**	**Yes**	
None		**Yes**	**Yes**			**Yes**

***Functionality index (Yes/No ratio)***	**2.333**	**0.666**	**0.933**	**3.83**	**1.888**	**6.500**

This table describes various microarray databases. The majority of the functions listed are described in detail in Additional file 2 (PhenoGen user manual). "**System requirements**" indicates whether there are special computational requirements to use the database and associated tools; "**LIMS**" (Laboratory Information Management System) indicates whether LIMS is available; and "**Programming expertise needed**" is self-explanatory. Functionality index is a ratio of the number of "**Yeses" **to "Nos" for a given database. "?" indicates that the functionality of the database was not readily evident ("?" were not used to determine functionality index).

## Construction and content

**Figure 1:**
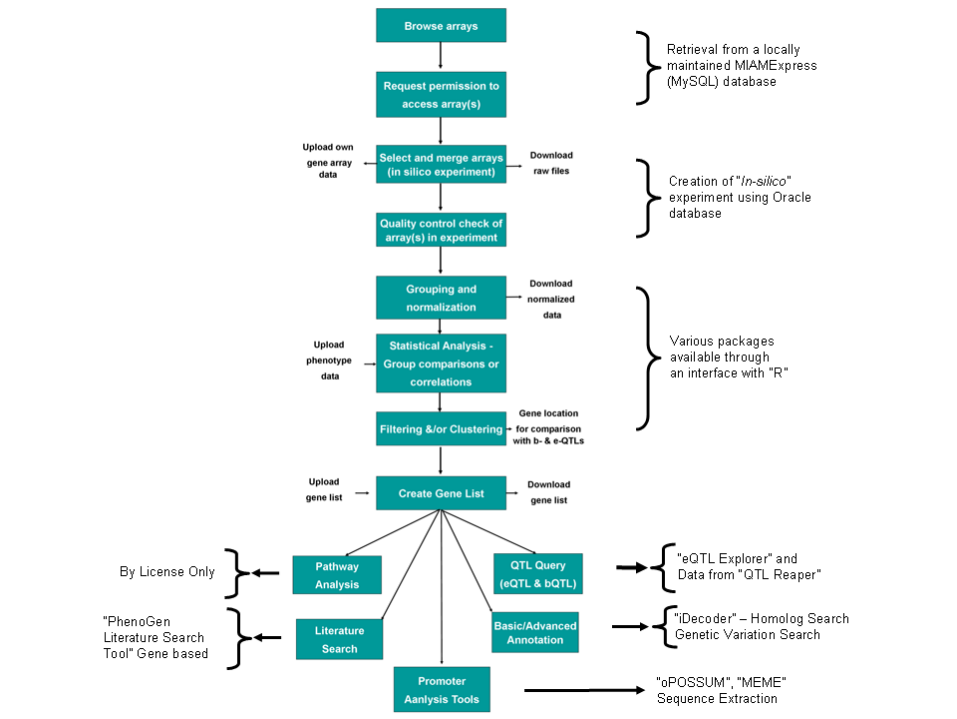
**The work-flow at PhenoGen**. This flow chart demonstrates how the work-flow for analysis of data at the PhenoGen website can be organized and shows different programming languages and tools available at PhenoGen.

An example of the work-flow at PhenoGen is shown in Figure 1. We maintain a local copy of MIAMExpress to insure MIAME/MAGE compliance of the microarray data stored in our database. For normalization, filtering and/or statistical analyses we have used various packages available in "R". A brief demonstration of how investigators can utilize our site to search for "candidate genes" for a given complex trait, e.g., fear conditioning in mice, is provided in Additional file 1.

The PhenoGen website is currently constructed to allow data to be classified as "Semi-public" or "Open Access". All of the information about the data uploaded at the PhenoGen website is visible to every registered user. The total number of microarrays, under different categories, from which data are available on PhenoGen is given in Table 2. Registered users have full access to data that are classified as "Open Access" and do not need to obtain permission from the curator of the data. However, users cannot access or download the "Semi-public" data unless the curator of the data (the Principal Investigator) grants permission to do so. Registered users can use the data for "*in-silico*" experiments on the website or can download the data for use with their own statistical software. At PhenoGen the curator(s) of the data (the Principal Investigators) also have an option to submit the data to microarray data repositories, such as ArrayExpress, as required by a number of journals [see Additional file 2: pages 115 to 116 in PhenoGen user manual].

**Table 2:** Data available on PhenoGen

**Current array count**				
**Organism**	**Category/genetic modification**	**Open access**	**Requires permission**	**Total**

Fly		0	24	24
Human		0	4	4
Mouse		557	142	699
	C57BL/6JxFVB/N F1	12	0	12
	Gene knock out	8	16	24
	Inbred strain	229	78	307
	Knock down	20	0	20
	None	0	4	4
	Recombinant inbred strain	168	0	168
	Selective breeding	70	0	70
	Transgenic	50	44	94
Rat		0	302	302
	Congenic strain	0	10	10
	Inbred strain	0	16	16
	None	0	7	7
	Recombinant inbred strain	0	146	146
	Selective breeding	0	123	123
**Total arrays**		557	472	1029

At present **PhenoGen **has microarray data from 1029 "samples" from different categories. Each "sample" represents mRNA obtained from an individual (animal, human or insect) sample and hybridized to an array (either Affymterix, CodeLink or a Custom oligonucleotide array). Data from any of these well-identified individual arrays can be used to conduct an "*in-silico*" experiment.

### Utility

To perform an "*in-silico*" experiment, users can select arrays from the current database (see Table 2) residing on the site (arrays can only be included in an "*in-silico*" experiment if the user has been granted access to the data or the data are in the Open Access domain). Microarray data from different laboratories can be combined to form "*in-silico*" experiments. Users can also upload microarray data, either Affymterix, CodeLink (Applied Microarrays, Inc., Tempe, AZ, now manufactures these arrays) or custom arrays (if chip definition files are provided), using MIAMExpress, for further analyses. A series of quality control steps can be carried out, once the user has selected arrays to perform the "*in-silico*" experiment. This should be done to ensure compatibility and overall quality of the arrays [see Additional file 2: pages 31–42 in PhenoGen user manual]. The data from arrays in an "*in-silico*" experiment can be normalized, filtered and statistically analyzed utilizing several normalization and statistical procedures available on-site. At numerous points in this process the user can download data, raw or normalized, from experiments being performed on site, for use with other statistical packages of his/her choice.

As with some other databases, PhenoGen offers a range of options for microarray data normalization, filtering and statistical analyses, including corrections for multiple comparisons. Users can compare gene expression profiles in two groups using any one of the available options, or can use one-way or two-way ANOVA models to check for overall differences when comparing more than two groups. We plan to provide tools to carry out clustering (k-means and hierarchical) analyses of microarray data in the near future. Furthermore, once an "*in silico*" experiment has been created, and the microarray data normalized, the user can search the database to determine the expression levels of any particular transcript(s). After choosing the correct (created) experiment, the user enters the probeset ID, or gene name or symbol (or any other annotation ID from the most popular genomic databases) [see Additional file 2: pages 60, 76–79 in PhenoGen user manual] and clicks "search", leading to display of expression data for the gene or genes in the chosen experiment. These data can be downloaded.

In addition to the standard statistical tools for assessing differential gene expression between or among groups, users can analyze the correlation between gene expression levels and phenotype (behavioral, biochemical or physiological). Such correlation analyses have been used in recent studies, by others [13-15] and our group [16], to ascertain "candidate genes" for complex traits with either a panel of recombinant inbred (RI) strains of mice (or rats) or a panel of inbred strains of mice. Users can access data available on site for whole brain gene expression profiles from either 20 inbred and 30 RI (BXD RI) strains of mice or 27 RI strains from the HXB/BXH panel of rats [17], and compare these data on gene expression to phenotypic data obtained with these same strains and species of animals in the user's laboratory, or to other phenotypic data (in the literature) related to brain function. Users can upload phenotype data (as a ".txt" file) for evaluating the correlation of gene expression with the phenotype. An example of such analysis of correlation of whole brain gene expression profiles with the contextual fear conditioning response in a panel of BXD RI strains, carried out using tools available on PhenoGen, is given in Additional file 1.

Another distinguishing feature of PhenoGen is its multiple offerings for further data analysis, once a list of differentially expressed or correlated genes is generated on site or up-loaded *de novo*. Complex behavioral traits reflect variations in biochemistry, physiology, and anatomy that are determined by the action and interaction of several or many genes. We [16,18], and others [19,20] have indicated that the combined use of gene expression data together with QTL (quantitative trait locus) analysis can provide for a better understanding of the genetics of complex traits. The availability of techniques of genetic mapping and statistical analysis has allowed association of complex behavioral traits with genomic loci (QTL analysis). In short, QTLs are the genomic regions on the chromosomes that can explain a portion of the genetic variation within a given complex trait. Most complex traits are also significantly susceptible to environmental influences.

A premise of QTL analysis is that the genetic material that contributes to the variance in the trait of interest is located in the area of the genome defined by the QTL(s) for the trait. A number of different factors, such as polymorphism(s) in the coding or regulatory region(s) of gene(s), resulting in either a change in function and/or a change in expression (mRNA) of the gene(s), may contribute to a QTL. Therefore one can "filter" the differentially expressed genes in the brains of animals which differ significantly in the manifestation of the trait, or genes whose expression levels correlate with the magnitude of the trait of interest across multiple strains of animals, through a QTL filter. In other words, one can ascertain the genomic location of differentially expressed or correlated genes, and determine whether the location of these genes falls into QTLs determined for the trait of interest. Localization of differentially expressed or correlated genes within a QTL for a trait of interest adds significant weight to the supposition that the gene located within the QTL is one contributing to the variance in that trait. The PhenoGen website allows users to access information for gene location in the genomes of mouse, rat and human, to access data (MGI) on QTLs for a number of traits, and to analyze whether the location of genes falls within relevant phenotypic QTLs.

A major caveat to considering **only **the genes that have a physical location **within **behavioral or physiological QTLs as candidates for contributing to trait variance, is that the expression levels of genes that reside outside the behavioral/physiologic QTLs may be regulated from within the behavioral/physiologic QTLs (an example of trans-regulation). The regulatory factor(s), themselves, would not have to be differentially expressed if a polymorphism resides in the target gene's expression regulatory region, and affects the **function **of the regulatory factor. Thus, any gene whose ultimate expression level is dependent on a genetic factor (cis or trans) within a behavioral/physiologic QTL becomes a candidate for contributing variance to the trait of interest.

We, and our colleagues, have used genomic marker data and information we have gathered on brain gene expression in male BXD RI mice and in HXB RI rats to determine the QTLs for the expression levels of genes in the brain (e-QTLs). This e-QTL data, available on PhenoGen, allows for ascertainment of the genomic site of control of expression for a multitude of genes and allows for determination of whether the genes are cis- or trans-regulated.

In essence, the differentially expressed genes that reside within behavioral/physiologic QTLs, and have their expression regulated from within the same QTL (cis-regulated), and genes residing outside of the behavioral/physiologic QTLs, but whose expression is regulated from within a relevant behavioral/physiologic QTL (trans-regulated), could form the list of candidates contributing to a quantitative trait of interest. It has to be clear, however, that polymorphisms in the coding region of a gene can and do produce altered function of a gene product and can also significantly contribute to the trait of interest. Such polymorphisms, even when located in highly significant QTLs, would not be amenable to being identified by an analysis which relies on the premise that differential expression of a gene contributes to trait variance.

In addition to the "QTL Query tools", the PhenoGen website offers a wide variety of tools to "interpret" a gene list derived on site or up-loaded by the user. Such a list can include a few or hundreds of differentially expressed genes derived from a typical microarray experiment. At PhenoGen, users have access to tools, including annotation (basic and advanced), promoter analysis (to understand transcriptional regulation) and literature searches (including "co-citation" searches) for the entries in a list of differentially expressed genes.

One of the ways to derive a "biological interpretation" of the results of the gene expression data is to analyze the biological annotations associated with the genes in a list of "candidates". i-Decoder, the underlying annotation tool used at PhenoGen, translates gene identifiers among many different nomenclatures, including gene symbols, RefSeq IDs, and probe names from both Affymetrix and CodeLink arrays, even when an up-loaded gene list contains multiple types of (non-identical) gene identifiers. This is accomplished by maintaining a local database of equivalents between identifiers that are available from the following eight sources: Affymetrix, GE Healthcare (formerly Amersham Biosciences), Ensembl, FlyBase, MGI, NCBI, RGD, and SwissProt [see Additional file 2]. In "Basic Annotation" tables every entry in the gene list is linked to the respective annotation in Entrez, MGI (or RGD), UniProt, and UCSC databases. A link is also provided to the "*in-situ*" hybridization images available at the Allen Brain Atlas to obtain regional distribution patterns of expression for genes in mouse brain. Another link is provided to the information at MGI about availability of genetically modified animals (transgenics, null mutants, etc.) for the genes in a gene list. Entries in the list of genes are also linked to information about genetic variations (e.g., single nucleotide polymorphism, insertion/deletion etc.), associated with the gene. In "Advanced Annotation Tables", users can personally select the available annotation information they wish to be displayed for the gene list.

To understand the transcriptional regulation of differentially expressed genes, users can use either oPOSSUM or MEME on the PhenoGen site. oPPOSUM uses human-mouse orthologs in calculating the over-representation of conserved transcription factor binding sites [21]. On the other hand, MEME explores the occurrences of previously uncharacterized transcriptional motifs [22]. Alternatively, the user can download the upstream sequences of genes of interest using the PhenoGen site and carry out similar analysis using other tools [23].

The literature search option on PhenoGen is an automated literature search that can be tailored to particular area(s) of interest by selecting a set of query terms. The automated literature search looks for articles in PubMed that mention any of the genes, including synonyms, in the gene list generated on site or uploaded by the user, and one or more of the chosen query terms. The results of the search are organized by the user-defined categories and by gene name, and contain direct links to PubMed citations. Also included in the results of a search is a list of articles where two or more of the genes from the gene list are cited in the same article (co-citation results). This allows the user to easily identify established relationships between genes.

## Discussion and conclusion

The PhenoGen website consolidates many data analysis and interpretation tools in an easy point-and-click command format, and it can also facilitate the sharing of data between investigators across the globe. We have extensive and up-to-date transcriptome databases for whole brain gene expression for BXD RI and inbred strains of mice and RI rats that can be used in an "*in-silico*" analyses of correlation with phenotypes arising from the functions of the central nervous system, and to identify "candidate genes" using behavioral and expression QTL data. In addition to "QTL Tools" we provide a number of tools for promoter/upstream sequence analysis, literature search, and tools to obtain annotation for a given list of genes. Although there are a number of other web-based tools available to carry out many of these analyses individually, PhenoGen provides one-stop access to most of these tools and to gene expression databases necessary to identify "candidate genes" for complex traits. Though at present the majority of the data available at PhenoGen is related to gene expression, the tools on the website can be adapted to handle other types of high throughput data, such as data derived from proteomic analysis. Another advantage of using the PhenoGen database, and associated analytical tools, is that users do not need expensive computational hardware and do not require extensive knowledge of programming languages. Websites such as PhenoGen are becoming indispensable for the life sciences research community so that scientists can arrive at biologically relevant interpretations of results of high-throughput studies[24].

## Availability and requirements

The PhenoGen database and associated tools are available to the scientific community at . Users need to register in order to carry out any "*in-silico*" experiments using the data available in the open access component on the site or with their own data. Users can also conduct "*in-silico*" experiments using the data deposited in the semi-public component of our database by obtaining permission to do so from the investigators who deposited such data. Protocols and means for obtaining such permission are available on site.

## Authors' contributions

SVB generated data from gene expression studies, assisted with e-QTL analysis, assisted in writing User Manual and this manuscript. CH performed coding and programming for all aspects of the PhenoGen website. TLP participated in organization of the website, and performed coding and testing. LS performed coding, statistical analysis testing, and helped in writing this manuscript. RL performed coding and testing, particularly in the e-QTL arena. KK performed coding and testing, particularly in transcription factor analysis tools. JG performed gene array analysis and site testing and assisted with the User Manual. DM performed coding for p-QTL analysis. AD performed coding for literature search modules. SL helped coding for statistical analysis modules in R. BS supervised and performed gene expression analysis and tested site elements. AE wrote User Manual. EE performed coding for integrating the PhenoGen with various public databases. KJ performed coding and assisted in programming for all aspects of the PhenoGen. JM developed of tools for e-QTL analyses (e-QTL Explorer). JKB introduced and helped program the QTL analysis in R. RWW introduced and assisted integration between WebQTL and PhenoGen. LEH supervised and directed coding and obtained funding.

PLH supervised collection of gene expression data, interpretation and testing the PhenoGen and helped with writing and editing this manuscript. BT conceived the underlying premises and structure for PhenoGen, organized the efforts of staff, secured funding and helped write and edit this manuscript

## Supplementary Material

Additional file 1 Example of use of PhenoGen website. This additional file provides an example of the use of the website to identify "candidate genes" for the contextual fear conditioning response in BXD RI mice. Click here for file

Additional file 2 PhenoGen user manual. This additional file is a user manual for the PhenoGen website. Click here for file
