# Supplementary material for: The PhenoGen Informatics website: tools for analyses of complex traits
Source: BMC Genet. 2007 Aug 30;8:59. doi: 10.1186/1471-2156-8-59 (PMC2034588; doi:10.1186/1471-2156-8-59)
Supplement: Additional file 1 — Example of use of PhenoGen website. This additional file provides an example of the use of the website to identify "candidate genes" for the contextual fear conditioning response in BXD RI mice. [file 1471-2156-8-59-S1.pdf]

Additional file 1

The PhenoGen Informatics Website: Demonstration of Tools to Identify “Candidate genes” for Fear Conditioning in BXD RI Strains of Mice.

We present an "*in-silico*" case study for the purpose of demonstrating the functions of the various tools available at the PhenoGen website. This case study illustrates how the PhenoGen website can be used for performing "candidate gene" searches even with data derived from the literature, which is melded with the data available on the PhenoGen website. For this case study we have utilized the phenotypic data published by Owen et al. [1] on "fear conditioning" in BXD RI mice. In recent years a number of different approaches, based on differential gene expression patterns, have been utilized to identify "candidate genes" for various complex traits ([2]). We will illustrate one such approach which starts by analyzing the correlation between gene expression profiles and a given complex trait (or phenotype) in a panel of mice or rats (or any other experimental model) that demonstrate a significant range of values for a particular phenotype. Such analyses generally result in a list of hundreds of genes that show significant correlation with the given phenotype. We, and others, have developed "filters" to further narrow down this list of correlated "candidate genes" [3-5]. We have used information about the genomic regions associated with the given phenotype, i.e., information obtained using quantitative trait locus (QTL) analysis, as a filter for narrowing the list of "candidate genes". In addition, we have used information, available on PhenoGen, about the QTLs that determine the expression levels of genes in the brain (e-QTLs). This e-QTL data provides another filter, using the genomic site of control of mRNA expression, and allows for determination of whether the genes are cis- or trans-regulated. We illustrate the use of the information about the phenotypic QTLs (p-QTLs) and e-QTLs to narrow down the list of "candidate genes". ONLY those correlated genes which have a significant e-QTL located within a p-QTL for a given phenotype would be considered as "candidate genes" [5-7] in this illustration.

We will demonstrate how users of PhenoGen can carry out an "*in-silico*" experiment: up-load the phenotype data, select a proper gene expression data set, perform quality control and normalization of the microarray data to increase the power of analyses, run analyses of correlation, filter gene lists and save the list of correlated, filtered transcripts. After narrowing down the list of "candidate genes" by using the overlap of p- and e-QTLs, we will demonstrate how users can obtain information about the transcription factors that may be involved in the regulation of expression of these "candidate genes", using additional tools available on the PhenoGen website. We will also demonstrate how some of the tools, such as "Literature Search", "Promoter

Analysis" and links to various databases, including the Allen Brain Atlas, available on PhenoGen, can expedite a search for information on biological interactions and biologic/physiologic relevance of “candidate genes”.

Initially users need to register on the Phenogen website in order to use the microarray data and various tools available. Users can register by clicking on the “Register” link on the Home Page (see pages 6 – 8 in the User Guide). Once the registration process is completed, users will be notified via e-mail within 24 hr that they can start using the PhenoGen website. After the registration process is complete, every time he/she enters the site, the user can fill out the required fields (username and password) on the Home Page of the PhenoGen website (http://phenogen.uchsc.edu) and then, by clicking on the “Login”, users will have access to the microarray data and analysis tools. Since we were using the “Open Access” microarray data for this example, we did not have to seek permission from any of the “curators” (Principal Investigators) of the microarray data for access to microarray data.

Log-on:

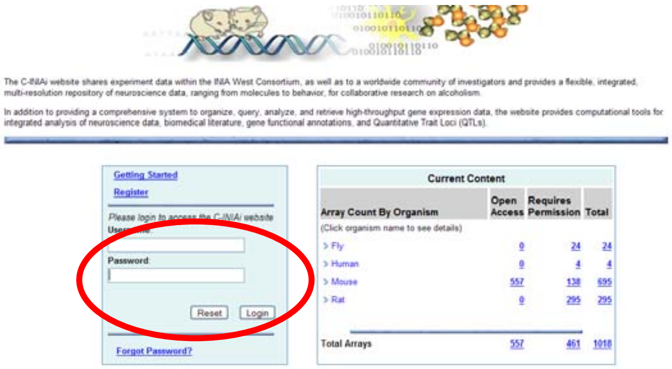

Create New Experiment:

The analysis of the correlation of the whole brain gene expression profiles with the “fear conditioning” phenotype in the BXD RI panel was initiated by clicking on “Experiments” to reveal the drop-down menu and then selecting “Create New Experiment” and “Do Correlation Analysis” from the drop-down menu. This leads to the page where users can upload the phenotype data and select the proper microarray data set (from stored data) for the correlation analysis. For this “*in-silico*” experiment, we obtained the phenotype data for BXD RI strains from Table 1 of the paper by Owen et al. (1). The data, labeled “BXD Phenotype Database”, are also available at:

http://www.nervenet.org/main/databases.html). At present, users have an option to carry out analysis of correlation with whole brain gene expression profiles obtained from panels of BXD RI mice, inbred mice, or a panel of HXB/BXH RI rats, with any behavioral/physiologic phenotypes available for these animals and related to brain function.

Users can also Create New Experiment:

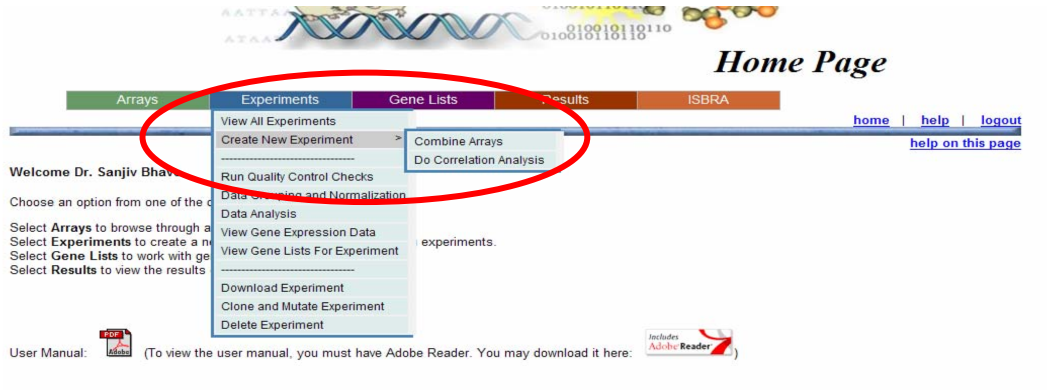

Phenotype data file upload:

The illustrative "*in-silico*" experiment was named "Correlation with fear conditioning" by us (but any other name would work) and a brief description of what we wished to do was added in the "Experiment Description" box. The phenotype data (strain mean values as a ".txt") file can be created and saved off-line using any of the available spreadsheet programs (in our case, Microsoft Excel). The ".txt" file contained two columns. The first column of the spreadsheet had the strain name and the second column had the phenotype data (see the example below). By clicking on the "Browse" link, one opens a file browser to select the saved ".txt" file containing the phenotype data and import it into the "Experiment". After selecting the ".txt" file with the phenotype data, users then select a proper microarray dataset (in our case a BXD RI panel with microarray data normalized using the RMA method) by clicking on the radio button next to the dataset (see below – and pages 28 – 30 in the User Guide). The columns in the phenotype ".txt" file should NOT have any heading AND the strain names have to match the strain names in the microarray datasets to proceed.

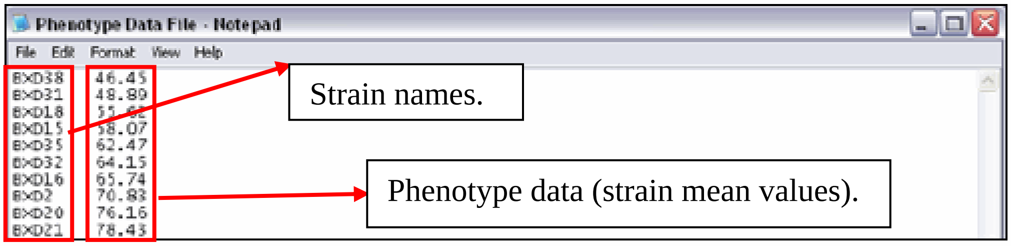

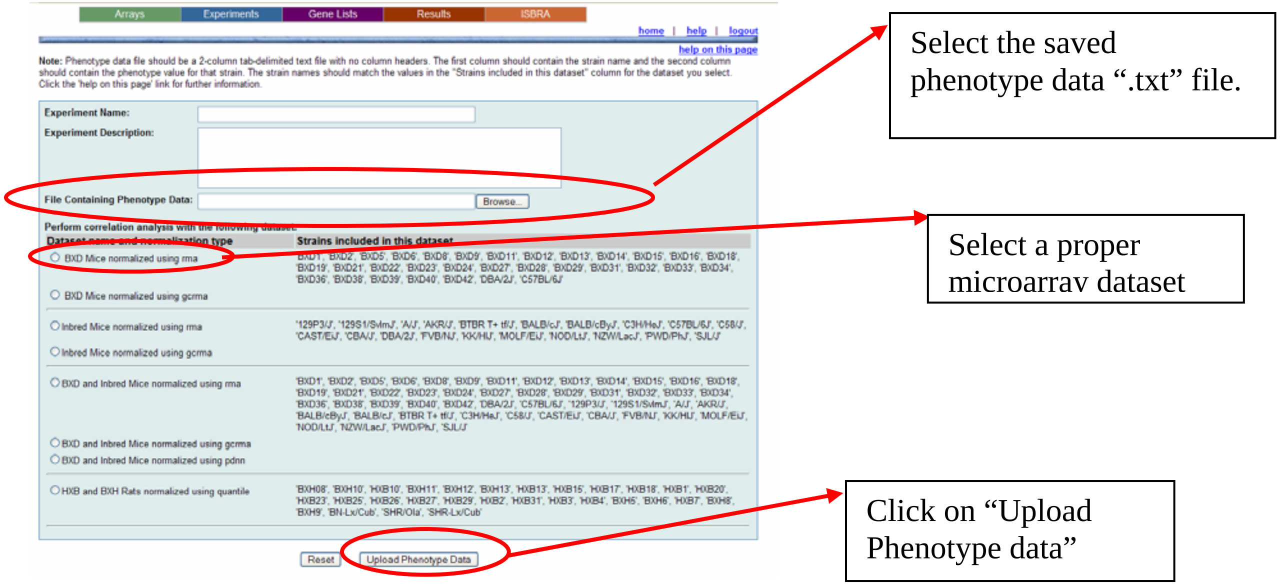

Once the microarray dataset (i.e., either from the BXD RI mice, or inbred mice or HXB/BXH rats) which matches the phenotype data is selected, the tools at the PhenoGen website will automatically pull out the gene expression data for the corresponding strains from the whole brain gene expression datasets by matching the strain names in the phenotype file with strain names in the microarray data files. For example, when we up-loaded the phenotype (fear conditioning) data for 19 BXD RI and two parental strains, the gene expression data for those corresponding 19 BXD RI and two parental strains were "automatically" selected and used in the correlation analysis (see pages 28 – 30 in the User Guide). At this point, the user can continue the analysis or save the experiment for future analysis.

At PhenoGen, users have access to whole brain gene expression profile data for 30 BXD RI strains and the two parental strains of mice i.e., DBA/2J and C57BL/6J, 20 inbred strains of mice and 26 RI HXB/BXH strains of rats. In the "BXD RI" gene expression dataset, each strain has 4 to 7 biological replicates for a total of 172 individual arrays. In this dataset, whole brain mRNA for each naive 10-12 week old male mouse was

hybridized to a separate array, (Affymetrix Whole Genome), i.e. no pooling of samples. In the "inbred mice" gene expression dataset, each strain has 4 to 7 biological replicates for a total of 90 individual Affymetrix Whole Genome arrays. Again, the whole brain mRNA for each naive 10-12 week old male mouse was hybridized to a separate array, i.e. no pooling of samples.   In the "HXB/BXH RI "gene expression dataset, each strain has 4 to 7 biological replicates for a total of 139 individual arrays. The whole brain mRNA for each naive 10-12 week old male rat was hybridized to a separate CodeLink Whole Genome array, i.e. no pooling of samples.

Data Analysis:

The data analysis for a stored experiment is carried out by returning to the Home Page, clicking on the “Experiment” drop-down menu and selecting “Data Analysis” (see below and page 55 in the User Guide). At this point users are asked to select the experiment they wish to analyze (see below).

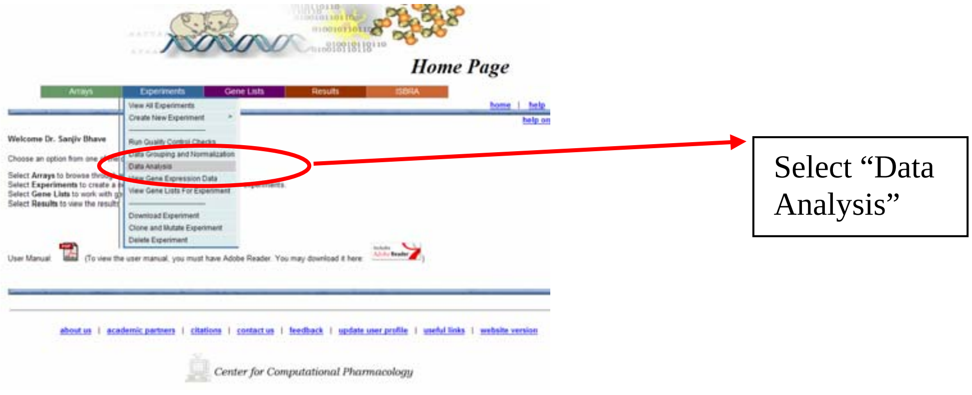

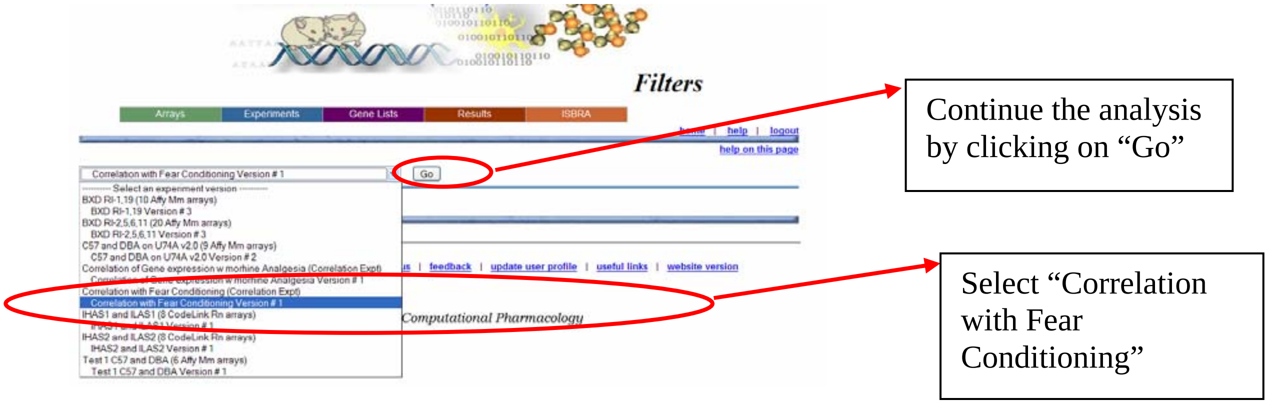

The experiment created earlier, which we called “Correlation with Fear Conditioning”, was selected from the dropdown list of experiments available to the user. At the PhenoGen website, such “*in-silico*” experiments

created by users are visible ONLY to those who created such experiments. Analysis options are then presented to the user by his/her clicking on the “Go” button.

Data Filtering:

At this stage we chose to remove the data for the “Affymetrix Control Probe sets” present on each array by choosing the “Affy Control Genes Filter” from the drop-down menu. This action removed the data for the Affymetrix control probe sets from all of the arrays in the experiment. We also removed from consideration the data for probe sets with expression intensity less than the “negative control probe sets” by choosing the option to remove “Probes with # samples below detection limit = 100%” from the dropdown menu (see below and page 51 in the User Guide).

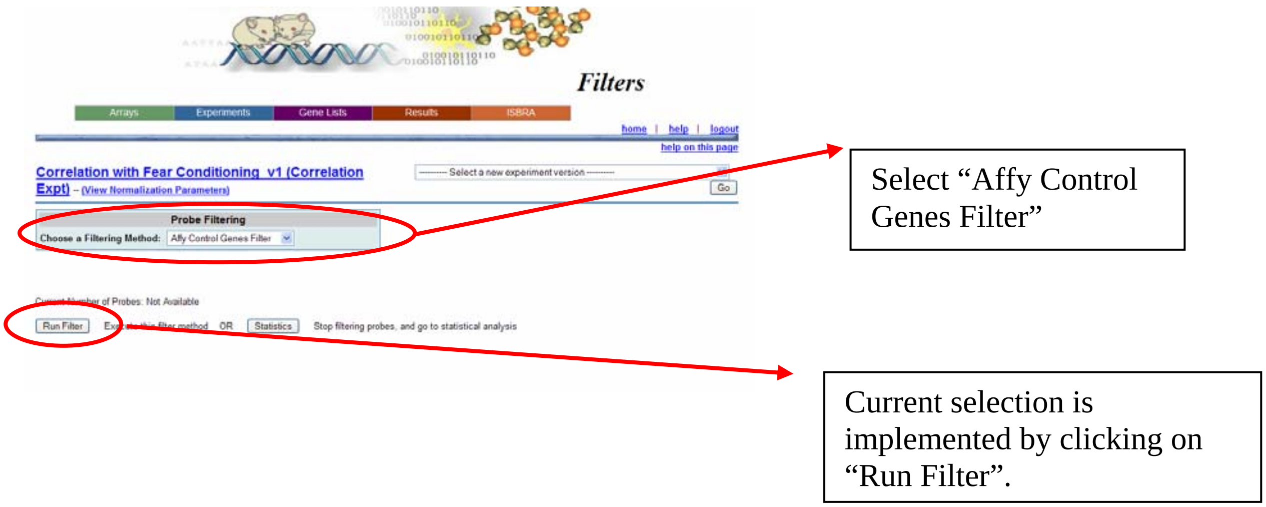

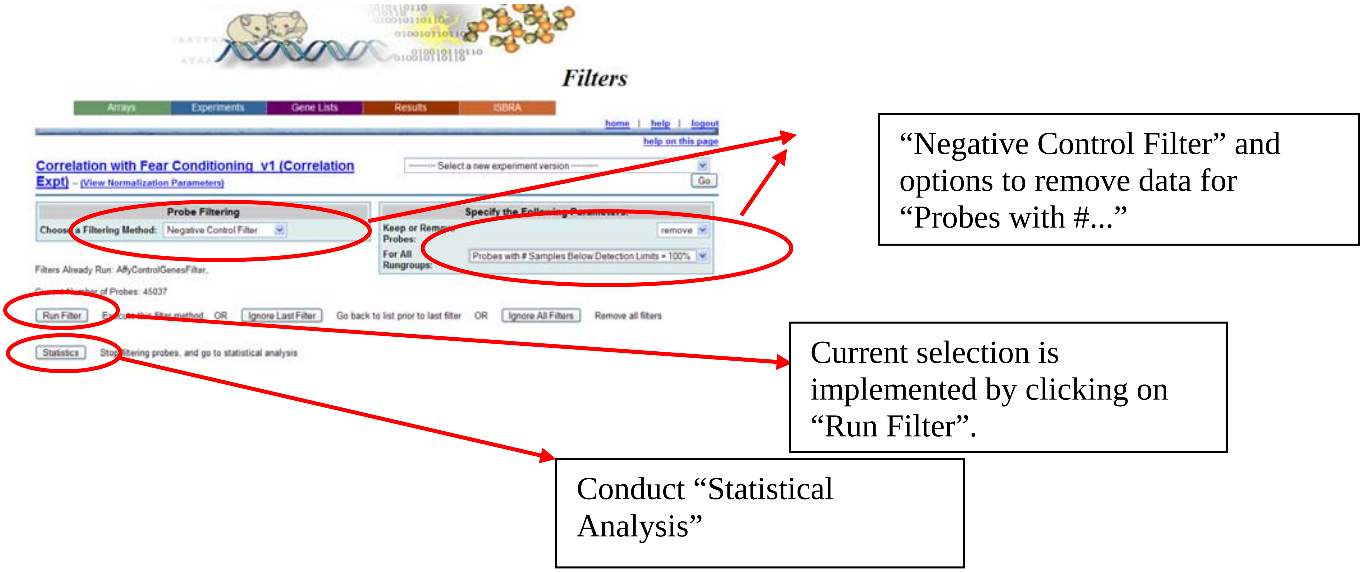

Correlation of gene expression with fear conditioning:

The statistical analysis of the correlation of the phenotype with the filtered whole brain gene expression profiles in a total 19 BXD RI and 2 parental strains was carried out by clicking on “Statistics” (see Screen Shot above). This presents users with options to select the type of correlation analysis (parameteric or non-parametric), type of multiple testing corrections, and the alpha level threshold to be used. A number of other statistical tools, in addition to the analysis of correlation, are available for different types of comparisons (see pages 53 – 59 in the User Guide). In the present example, we used carried out the analyses of correlation of gene expression intensity with the phenotypic data (strain mean) using a Spearman correlation test (a non-parametric test) without any correction for multiple testing (see below and page 55 – 56 in the User Guide).

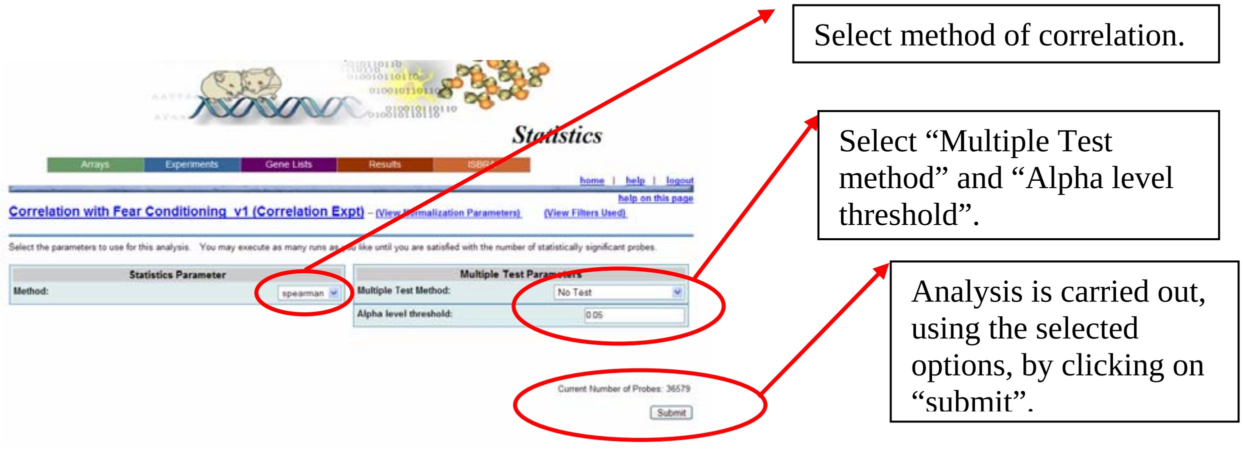

The list of correlated genes (405 probesets, p<0.05) thus obtained was saved for further analysis (see below and pages 68 – 70 in the User Guide).

Save Gene List:

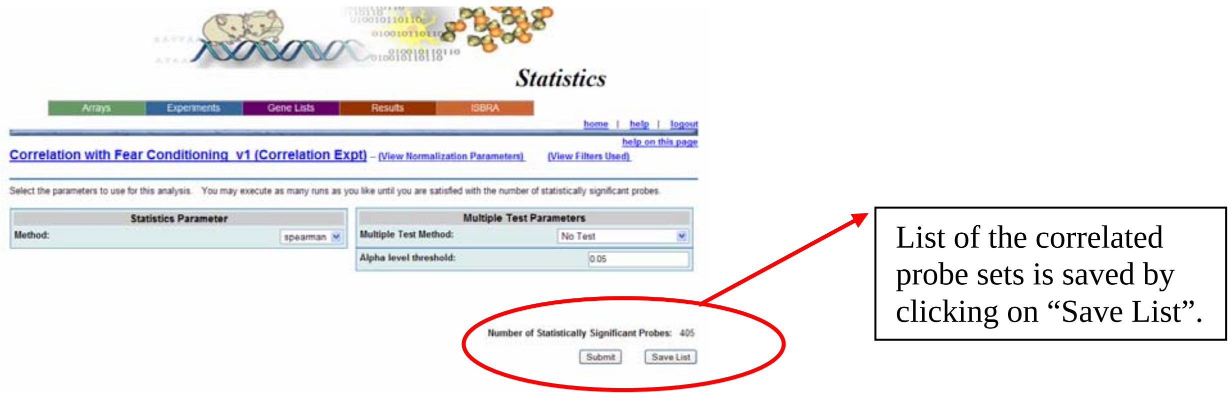

Save Gene List:

By clicking on the "Save List", users are presented with options to name the list, save a brief description (e.g., the description of the parameters used in the analysis) and to share, if desired, the list with other users. After entering the descriptive information the gene list is finally saved, along with the relevant information, by clicking on "Save Gene List" (see below and pages 68 – 70 in the User Guide).

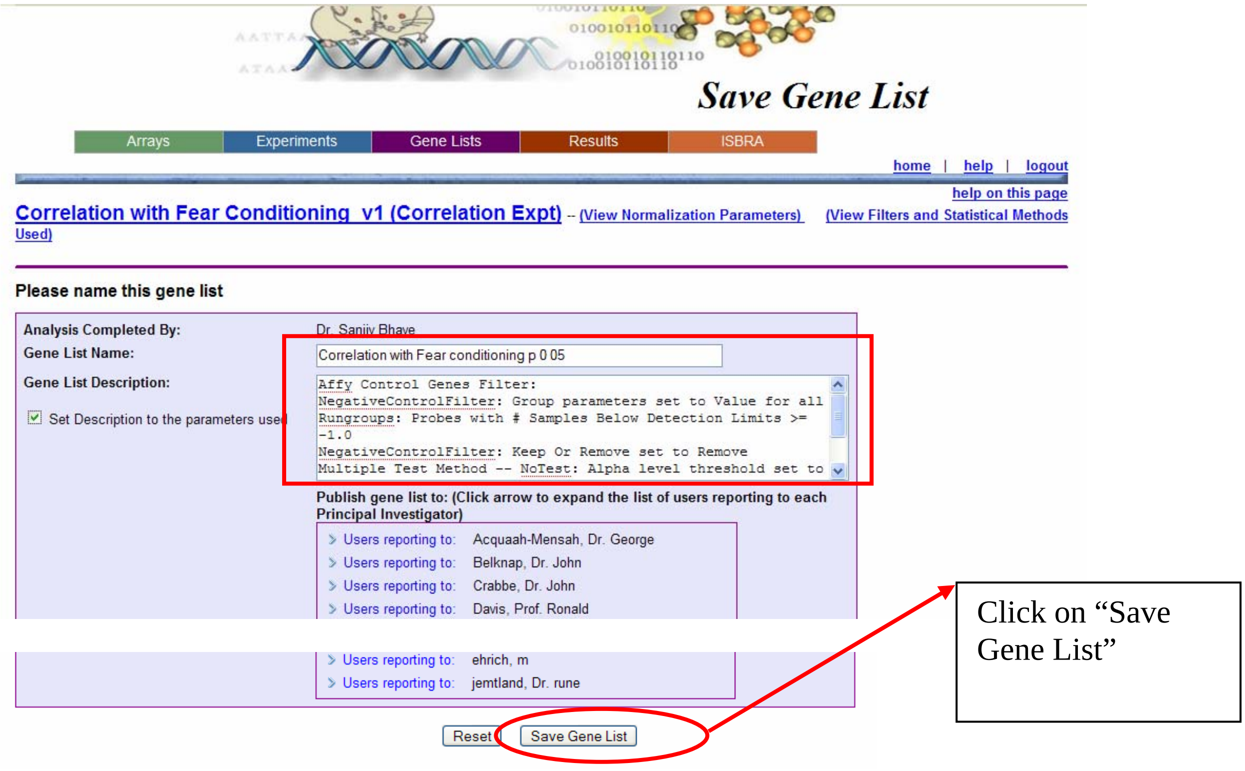

Users have access to a multitude of tools available to obtain annotations, chromosomal locations, homolog information or information about SNPs in the listed elements in these "gene lists" (see pages 73 – 79 in the User Guide).

QTL Tools:

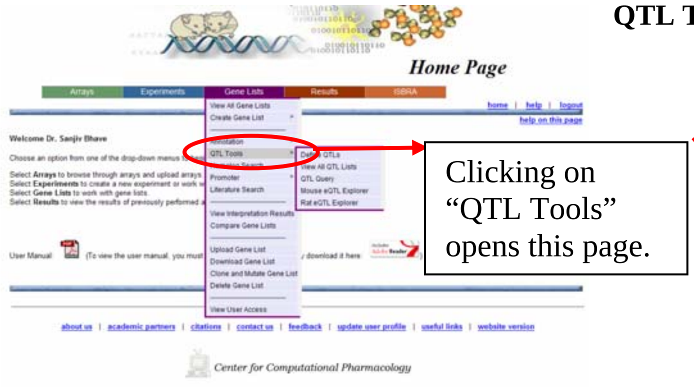

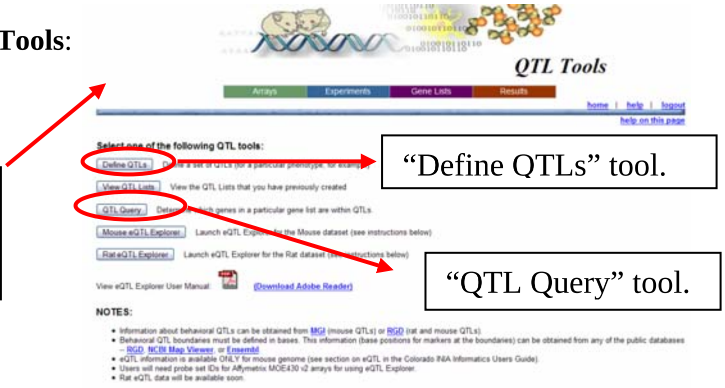

A number of tools are also available to use p-QTL related information, available in the public domain and at PhenoGen, to further narrow (filter) the list of "candidate genes" for a given phenotype. Users can use the information about genomic boundaries, generally available either on PubMed or MGI, and "define" the p-QTL regions for a given phenotype. The information about the p-QTLs for "fear conditioning" was obtained from the study by Owen et al. [1]. We used this information to identify which genes, in the list of correlated genes for the given phenotype, were located in the p-QTL regions (and which genes had overlapping e-QTLs and p-QTLs, see below).

Define QTLs:

The p-QTL regions (boundaries) for the "fear conditioning" phenotype [1] were entered using "Define QTLs" tool (see page 81 of the User Guide). Users can access "Define QTLs" by clicking on the "Gene List" drop-down menu in the tool-bar on top of the Home Page and selecting "QTL Tools". This action opens the "QTL Tools" page. The p-QTL boundaries entered by us in this "*in-silico*" experiment were chr1: 85 to 102 Mb; chr12: 2 to 20 Mb and chr17: 49 to 65 Mb [1].

On the "Define QTL" page the user can enter a name for the p-QTL list they wish to create and save, they can select the organism (mice, rats or humans) from which the p-QTLs were obtained and also enter the relevant information for the p-QTL i.e., chromosome number and start and end base-pair limits. In our case, we entered "Fear Conditioning" as the QTL List name, and selected "*Mus musculus*" from the drop-down menu for selecting "Organism". Then we entered "FC1" as the "QTL identifier", entered "1" for the "chromosome number" for that p-QTL and entered "85000000" and "102000000" in the "Start bp" and "End bp" fields respectively. Then the information for the p-QTL on chromosome 12 was entered by clicking on "Add New QTL" which opens additional fields for "QTL Identifier", "Chromosome", Start bp" and "End bp". We entered the information about the p-QTL on chromosome 12 in these fields and clicked on "Add New QTL" to enter relevant information about the p-QTL on chromosome 17. After all of the information had been entered we clicked on "Save QTL List" which saved all of the p-QTL information entered in these fields.

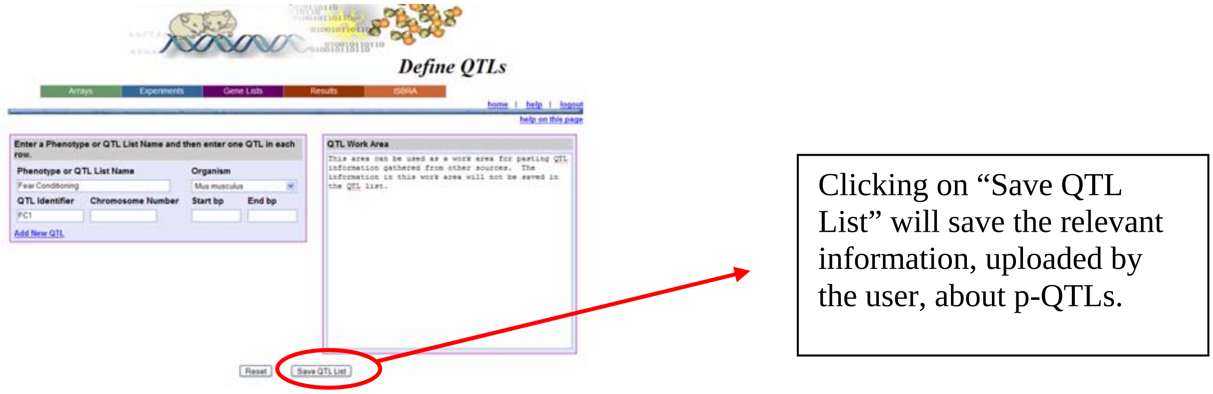

QTL query:

By using the "QTL query" tool, users can simultaneously obtain a list of genes (probesets) that are located within the given p-QTL of interest and a list of genes (probesets) that have a significant e-QTL overlapping with the given p-QTL. The "QTL query" tool is available on the "QTL Tools" page (see above). By clicking on "QTL query" users are asked to select the gene list that they wish to "filter" (see below and pages 83 – 84 in the User Guide). In this case we selected "Correlated genes with Fear Conditioning in BXDs".

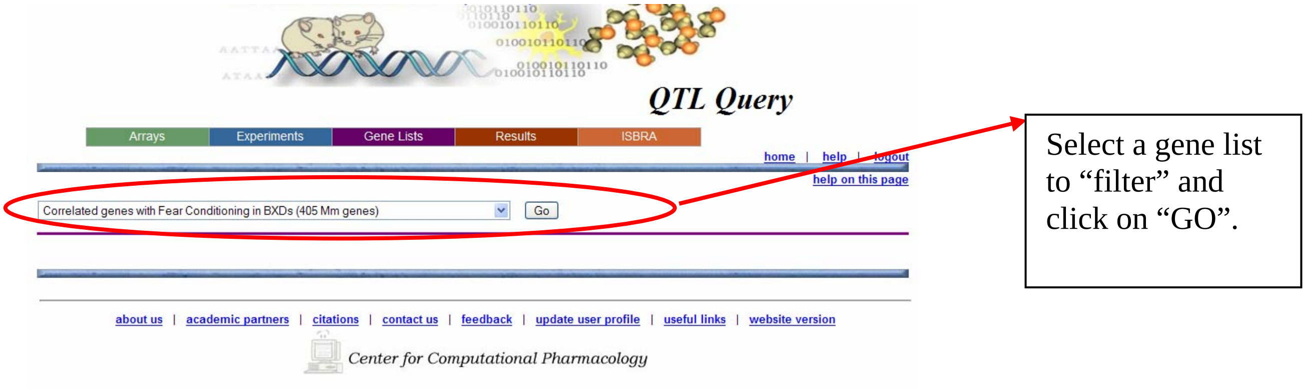

Next, users are asked to select a phenotypic QTL list that they wish to use. We selected the QTL list for "Fear Conditioning" that we had saved using the "Define QTL" tool. As noted above, using the "QTL Query" tool, one can also obtain the list of genes (probesets) that have a significant e-QTL overlapping with a p-QTL. At the PhenoGen website, users can access e-QTL data that were obtained using whole brain gene expression data (Affymetrix Mouse Whole Genome Array - MOE430 v2 for a panel of 30 BXD RI strains). For the e-QTL analysis, probe set intensity values were normalized using RMA. Mean expression levels within strains were used as phenotypic values in a QTL analysis implemented in the R/qtl program running in R (for details about

analysis see pages 73 – 74 in the User Guide and [5]). These e-QTL data are available in the “Basic Annotation” tables (see page 76 in the User Guide) as well as via e-QTL Explorer (see pages 154 – 165 in the User Guide). Within the "QTL query" tool, users are given an option to choose the threshold for the e-QTL (MaxLOD score) p value from the drop-down menu (see below and page 83 in the User Guide).

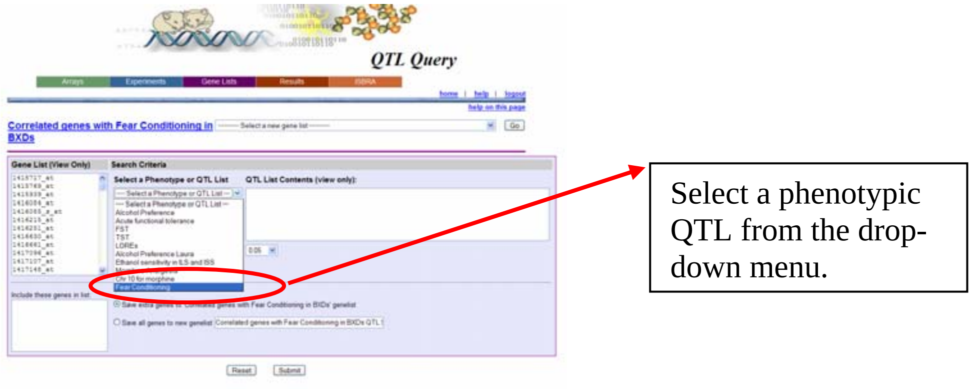

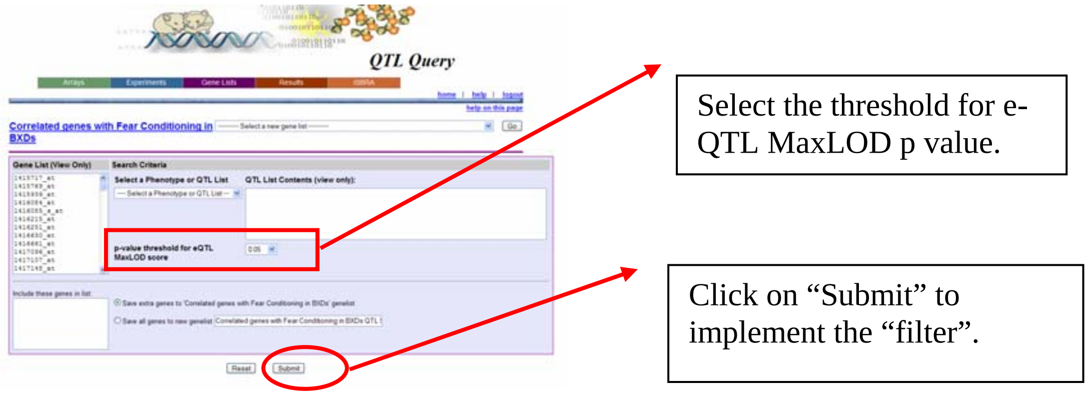

The output from the use of “QTL query” tool, when used as described above, is two gene lists: a list of correlated probesets, the genomic location of which overlaps with p-QTLs; and a list of correlated probesets with overlapping p-QTLs and e-QTLs (that later gene list, for our example, is shown in Table 1). The output of the QTL query shows the p-QTLs (1st column), the genomic boundaries used as “filters” (2nd and 3rd columns), genes (probe sets) physically located in these p-QTLs (4th column) and genes with a significant e-QTL overlapping with a p-QTL (last column (see below)). Both *cis*-regulated genes (genes that have a significant e-QTL located within a p-QTL, AND are also physically located within the same p-QTL) and *trans*-regulated genes (genes that have

significant e-QTL located within a p-QTL, but are located OUTSIDE of the p-QTL region) can be identified using this tool.

QTL Query Results:

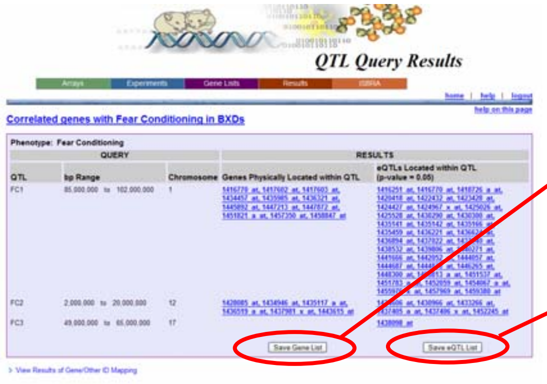

Click to save "Gene List" of probesets physically located in p-QTL

Click to "Save eQTL List" – a list of probesets with overlapping p- and e-QTLs

The list of correlated genes (probesets) that are located within the p-QTLs for fear conditioning (QTL query output in the 4th column) and the list of genes (probesets) that have significant e-QTLs overlapping with p-QTLs (QTL query output in the 5th column) were saved by clicking on the "Save Gene List" link provided in the respective columns (see User Guide, page 85). Both of these gene lists are currently available in our "Demo" section on PhenoGen. Further information regarding the eQTL and the location of the gene of interest within the eQTL can be obtained simply by clicking on the probeset ID (which is linked to the relevant information about e-QTL) in the "QTL query Results" table (see below).

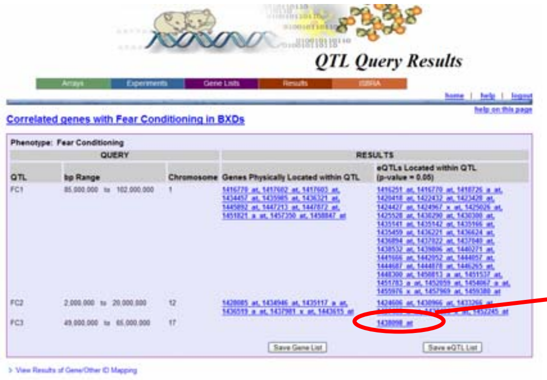

Clicking on the probe set ID opens a new window (or a tab) in the browser to display relevant e-QTL information associated with that particular probe set (see below).

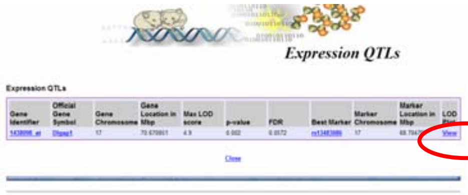

Users can view "LOD plots" for that probe set by clicking on "View" (see below).

The “Logarithm of odds” (LOD) plot associated with that particular gene (probeset) can be viewed by clicking on “View”. LOD plots show the “linkage disequilibrium scores” across the whole genome and the peak (Maximum LOD score) value which is associated with the region of the genome (eQTL) that may play a role in the regulation of expression of the mRNA for that particular gene.

LOD Plots:

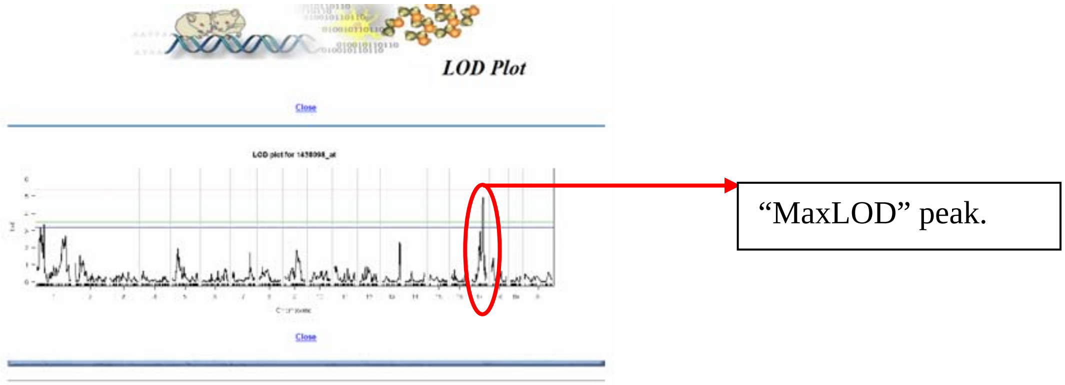

Advanced Annotation Example:

One of the major tools developed at PhenoGen is “i-Decoder”. iDecoder is an annotation tool that translates gene “identifiers” into many different nomenclatures, including gene symbols, RefSeq IDs, and probe names from both Affymetrix and CodeLink arrays. Because of this tool, users have an option to upload “gene lists” with any of these identifiers (including a list with a mix of these identifiers) and obtain any other annotation for the given identifier. Most of the microarray platforms have multiple probes (probesets) for a given gene. iDecoder can translate these multiple IDs for a gene and retrieve that information for the users (see below and information about “Advanced Annotation” on pages 77 – 79 in the User Guide). It is evident from the example that a given “identifier” can be associated with a multitude of other “identifiers” (and annotations) from various databases.

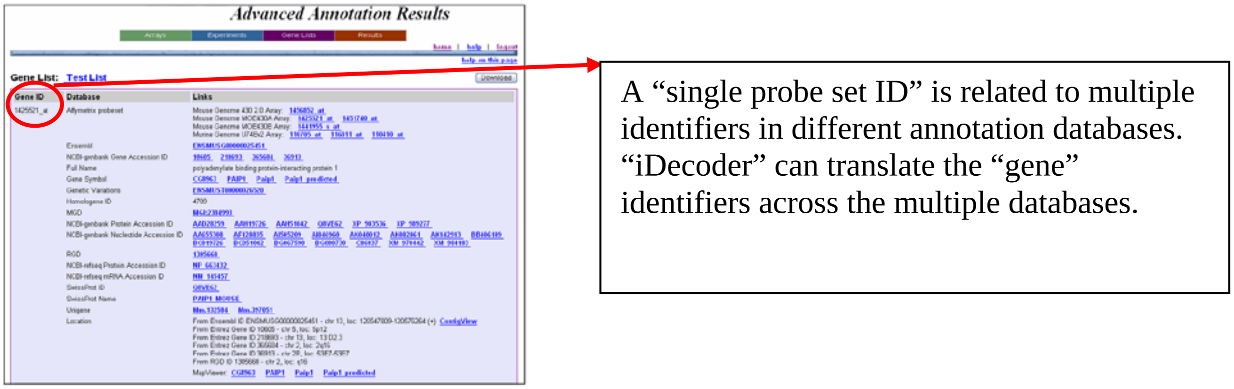

One of the issues that arises with iDecoder is that when the user applies the “QTL query” filter to a gene list the output can contain duplications generated by probesets with similar annotation. However, a simple solution to this problem is to compare the original gene list and the result of the “QTL query” to determine the “intersect” of these two lists (see below and on pages 64 – 65 in the User Guide). The resulting gene list can be saved (see the “Demo” and page 101 in the User Guide).

Compare Gene Lists:

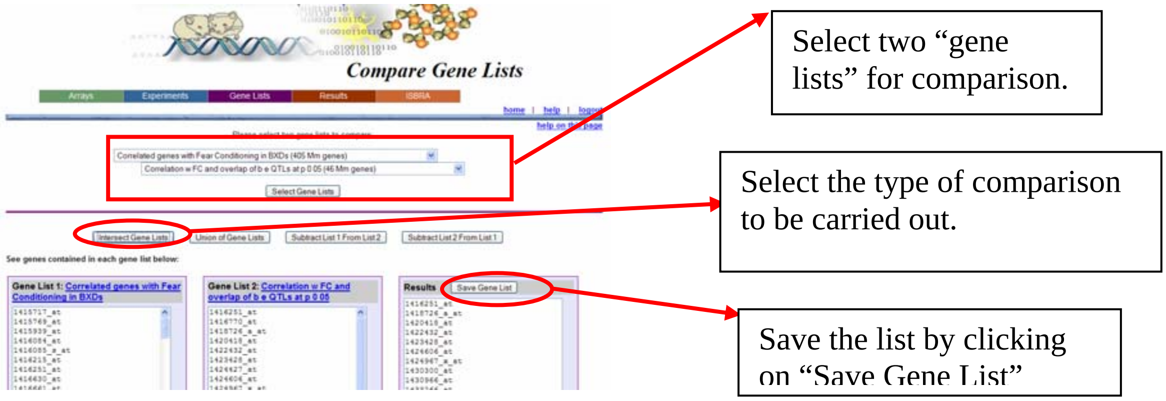

Promoter Analysis:

Another tool available at PhenoGen to understand biological relevance of gene lists is “Promoter Analysis”. Transcription factor binding sites (TFBS) are short repeating degenerate (genomic) sequences that are involved in protein-DNA interactions, usually within the promoter (upstream) regions of genes, essential for the regulation of transcription. Experimental methods for characterizing TFBS, such as footprinting assays, are too time-consuming and laborious to be used on a large scale [8]. Several dozen computational tools have been developed for the identification of degenerate sequences called ‘motifs’ [see reviews 9-11]. The goal of these

tools is to identify short sequences that are statistically overrepresented in the "genes" (sequences) of interest. Depending on the strategies used for the identification of TFBS, these tools can be classified into several categories. For example, tools may be based on searching for matches to known transcription factor binding site motifs, or alternatively, tools may be based on searches for novel motifs that may represent binding sites that have not been characterized. Another distinction is whether the computational tool uses information from a single species or takes advantage of conservation across multiple genomes in the promoter region to reduce the search space.

The PhenoGen website provides the ability to perform promoter analyses by applying several different computational tools. For our example, using the tool "Promoter Analysis", a search for known motifs was executed by oPOSSUM [12], which is a system for determining the over-representation of TFBS within a set of co-expressed genes, as compared with a pre-compiled background set (see pages 80 to 82 in the User Guide). Users can access tools for "Promoter Analysis" by clicking on the "Gene List" drop-down menu of the tool-bar on the Home Page and selecting "Promoter Analysis – oPOSSUM".

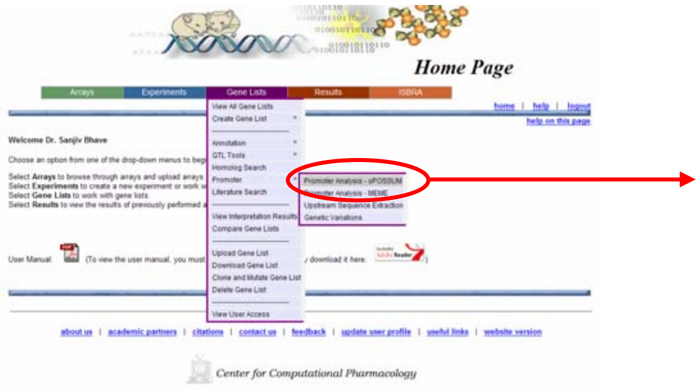

Click on "Promoter Analysis – oPOSSUM".

Clicking on the "Promoter Analysis – oPOSSUM" link opens a page where users can select different parameters for the analysis of transcription factor binding sites. Initially, users are asked to select a saved "gene list" from the drop-down menu (see below) for the analysis.

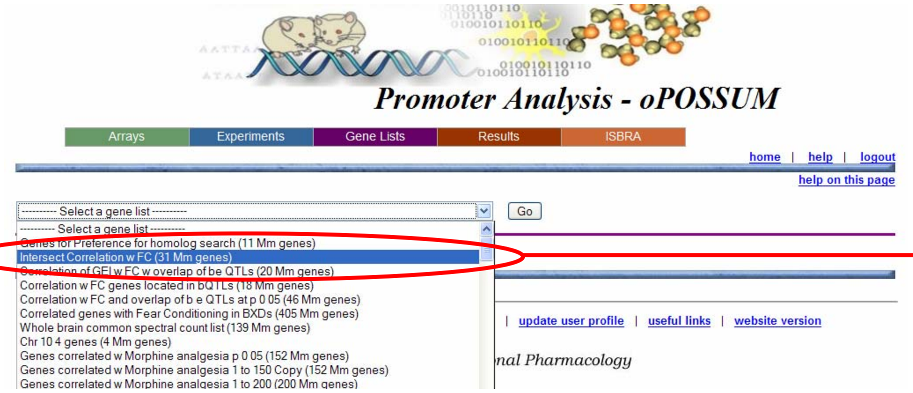

Select a gene list for the analysis and click on “Go”.

Promoter Analysis oPOSSUM:

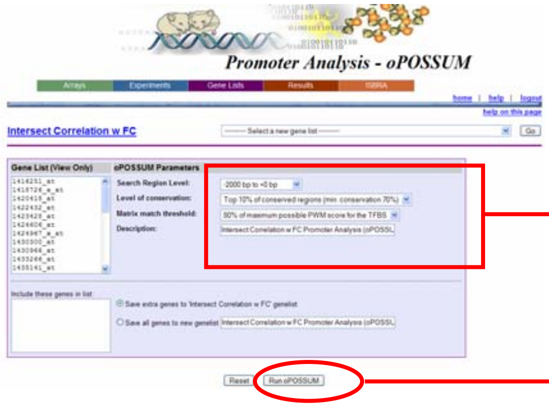

Users have options to choose bp region to be searched, level of conservation and the matrix match threshold to be used in the search.

Click on “Run oPOSSUM”.

Clicking on “Go” opens a page where users can select different parameters for the analysis of transcription factor binding sites, such as the length of the “search region”, “level of conservation” of the sequence, and “Matrix match threshold” (see [12]). oPOSSUM relies on the Jaspar database ([13], http://jaspar.genereg.net) of transcription factor motifs. In order to limit spurious TFBS sites, the sequences are filtered for conservation with the aligned orthologous mouse sequence, such that only sites that fall within these conserved regions are kept. The oPOSSUM run takes some time (generally more than a few minutes – depending on the number of genes in the list) and therefore users are notified that they will be informed via e-mail about the completion of the oPOSSUM run. The results of the oPOSSUM search can be accessed from the “Results” dropdown menu of the tool-bar on the Home Page (see below and pages 115 – 118 in the User Guide).

“Results”

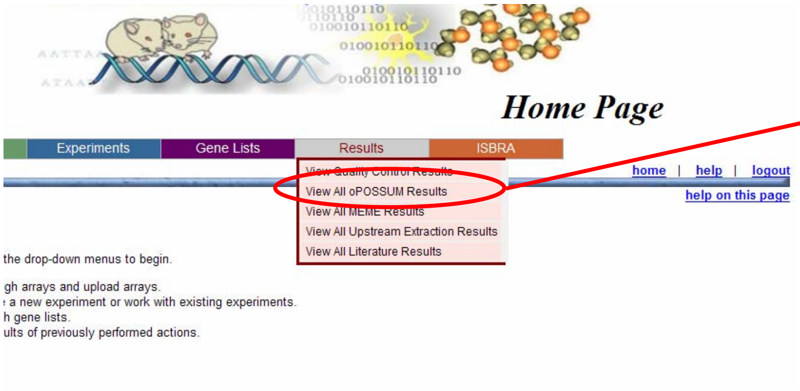

Click on “View All oPOSSUM Results” to access results of oPOSSUM analysis.

Clicking on the “View All oPOSSUM Results” link will open a page displaying results for all of the “oPOSSUM” analyses carried out by the user (see below – and pages 115 – 118 in the User Guide).

View All oPOSSUM Results:

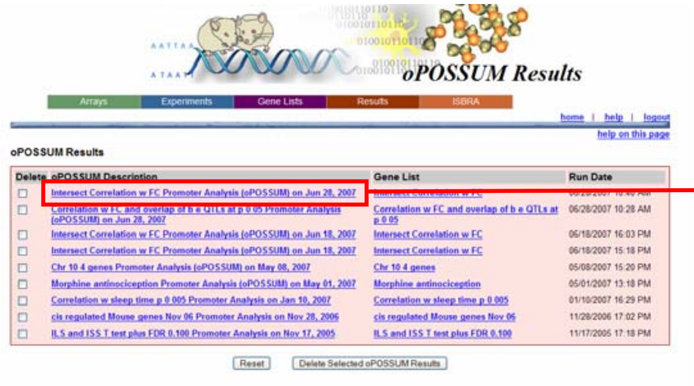

Clicking on the link will open the results table.

oPOSSUM Results:

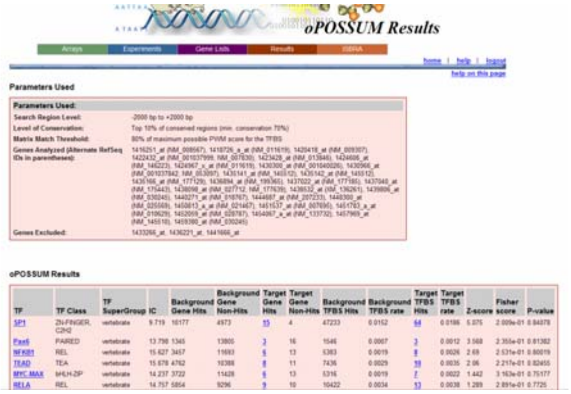

The oPOSSUM results table lists the top transcription factor motif occurrences (or hits) in the list of “candidate genes”. The columns indicate the transcription factor (TF), the number of hits and hit rates in the

background sequences and the 2KB upstream region (chosen by us) of the 25 (candidate) genes, and the Z-score, which uses a simple binomial distribution model to compare the rate of occurrence of a TFBS in the target set of genes to the expected rate estimated from the pre-computed background set. Statistical significance of the results of oPOSSUM analysis, based on the Fisher's Chi square test, is also provided to the users.

For our demonstration experiment, one transcription factor, Pbx1, which shows a significant increase in target binding rate, is located on chromosome 1 (at 169.95 Mb) very close to the e-QTL locus (at 167.91 Mb) involved in regulation of 8 out of 21 transcripts that have a significant e-QTL on chromosome 1. Another transcription factor that demonstrates a significant increase in target binding rate in our analysis, Foxd2, is a factor belonging to a Forkhead family. Though physically located on chr 4 at 99.14 Mb, Foxd2 has been observed to interact with the Sox family of transcription factors [14]. Sox9, Sox5 and Sox17 all showed a higher rate of representation, though not statistically significant, in the upstream regions of several of our "candidate genes". These results suggest that genes regulated by transcription factors Pbx1, Foxd2 and the Sox family may play a role in the fear conditioning response.

The oPOSSUM search is based on searches for known motifs. To explore the occurrence of previously uncharacterized motifs, there are many software options. Although these have not been directly incorporated within the PhenoGen website as with oPOSSUM, they can easily be accessed by using other publicly available webservers. The work of Tompa et al., [13] provides a comprehensive approach to promoter analysis based on curated test sets of many of the leading motif finding methods using single species data [11]. Tompa et al [13] found that the software MEME (**M**ultiple **E**m for **M**otif **E**licitation [15]) was one of the best performing algorithms on mouse data. At the PhenoGen website we have provided a link to MEME (see pages 92 to 93 in the User Guide). Users also have the option to download the upstream sequence, for a list of genes, using the "Upstream Sequence Extraction" tool so that they can use any tools for evaluating the transcription factor binding sites.

Literature Search:

A further tool available at the PhenoGen web site to understand biological interactions among "candidate genes" is the "Literature Search" option. Users can carry out a literature search for a given "gene list" by clicking

on “Literature Search” on the “Gene List” drop-down menu of the tool-bar on the Home Page. This opens a page (see below) where users are asked to select a “gene list” from the saved “gene lists”.

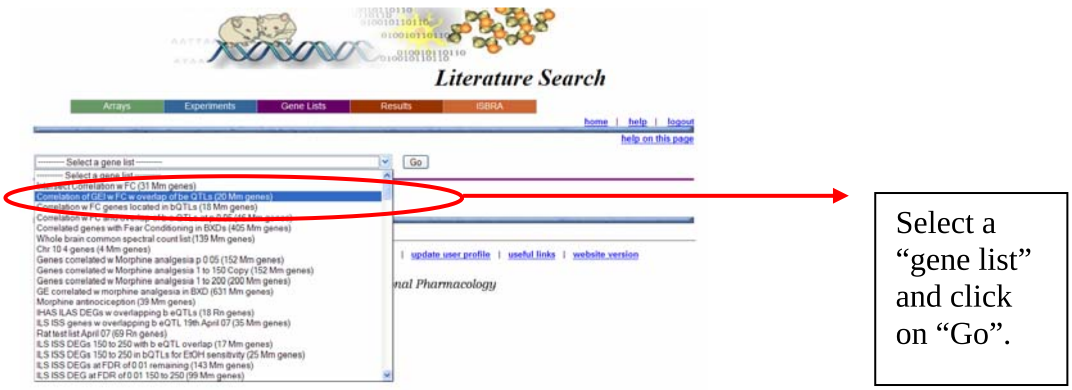

By clicking on “Go” one opens a page where users are given options to select the “categories” (such as “Behavior”, “Genetics”, “Biochemistry” etc.,) and enter certain “key words” (or phrases) in the boxes provided to narrow the literature search. The “Literature Search” tool uses the gene names (symbols and synonyms) for every object in the gene list and searches the PubMed database for their co-occurrences with any of the “key words” as defined by the user (see pages 77 to 78 in the User Guide). This tool also looks for “co-citations” i.e. citation of two or more of the objects, in the gene list, in the same abstract (and/or full text).

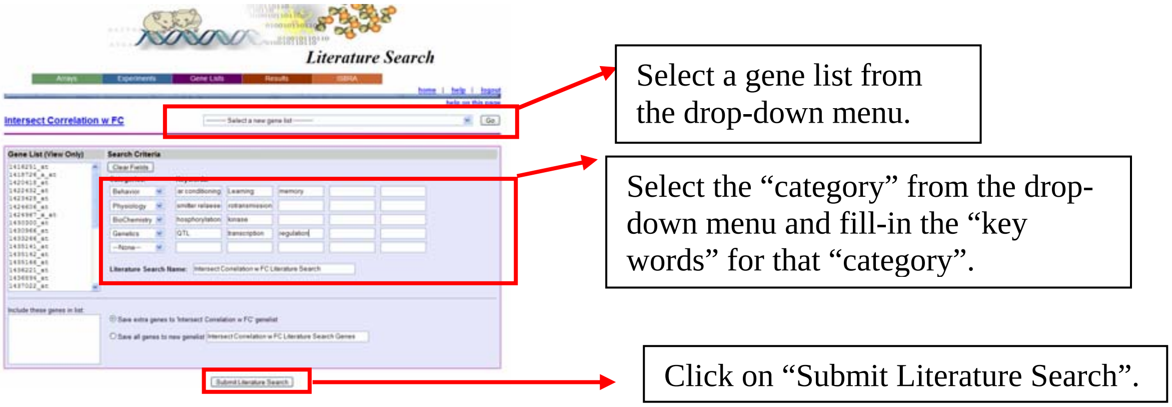

PhenoGen uses a local copy of PubMed database (updated once a month) to carry out the “Literature Search” (see pages 98 - 99 in the User Guide). Since the PubMed database search may take some minutes (or more depending on the number of genes in the gene list) users are notified via e-mail once the search is complete. Similar to the output of oPOSSUM (and MEME), the results of the search can be accessed by clicking on the link

“View All Literature Results” provided under the “Results” dropdown menu (see above and pages 124 – 126 in the User Guide).

Literature Search Results:

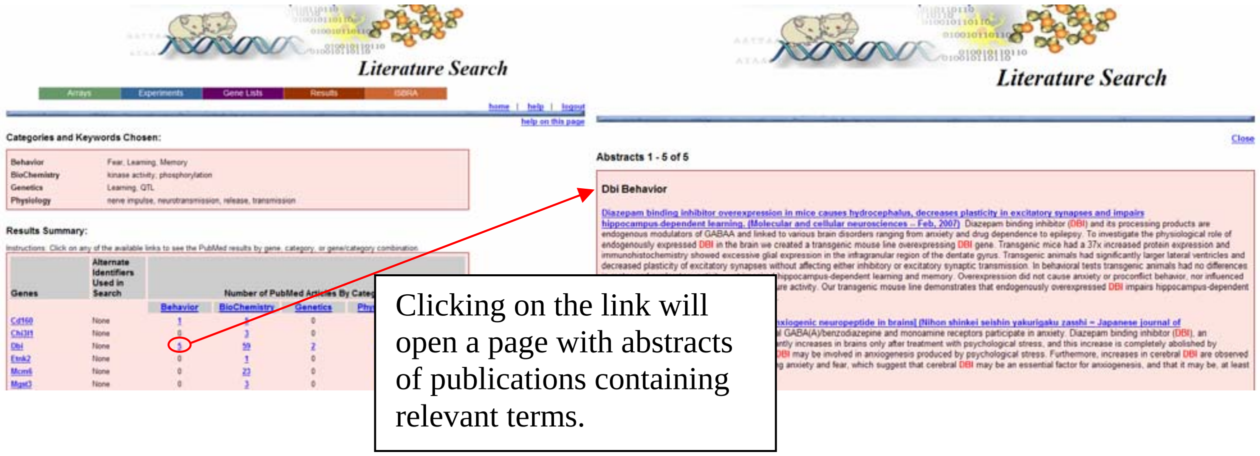

The use of tools such as “Literature Search” is extremely helpful when one is dealing with a large number of genes (usually the case in most of the “omic” studies). Such automated “text mining” tools, though still in the early developmental stages, can be very efficient and time saving tools available to the research community [16]. In our demonstration, the role of one of the correlated genes, which came through all the filters, Dbi (diazepam binding inhibitor), in learning (hippocampus-dependent learning) was evident from the PubMed database search (see the “Literature Search Results” in the Demo). A number of other “candidate genes”, such as Synaptotagmin II (Syt2) play a role in neurotransmission, while recent studies have implicated Chitinase 3-like 1 (Chi3l1) as a potential schizophrenia-susceptibility gene (see the “Literature Search Results” in the Demo). One of the problems associated with natural language processing (NLP), a technique used in the “Literature Search”, becomes evident by clicking on the link for the CD160 gene, under the “Behavior” column. The automated text mining tools generally will consider any abstract (or full text article) with both of the query terms, “CD160” and “behavior” in this instance. The output will display the abstract irrespective of the actual relevance to the user's investigation. Therefore, the user needs to screen the literature results to filter out any irrelevant references.

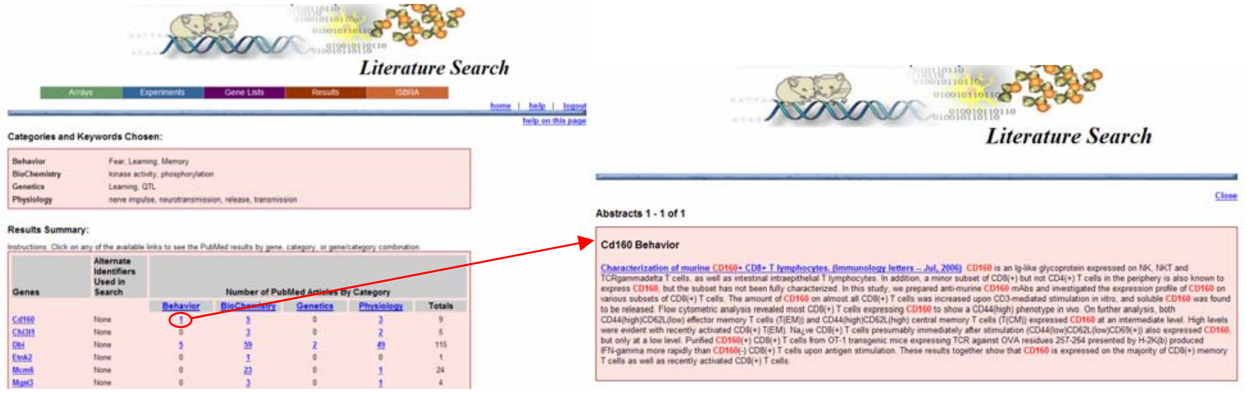

Conclusions:

The high-throughput computation tools available at the PhenoGen website make data storage, sharing, analysis, and interpretation easier than assessing single tools separately. In addition, the tools, such as “QTL Tools”, “Promoter Analysis” and “Literature Analysis”, available for use with large lists of genes, make the overall process of identifying “candidate genes” for complex traits readily accessible to investigators.

References:

1. Owen EH, Christensen SC, Paylor R, Wehner JM: **Identification of quantitative trait loci involved in contextual and auditory-cued fear conditioning in BXD recombinant inbred strains**. *Behav Neurosci* 1997, **111**(2):292-300.
2. Nadler JJ, Zou F, Huang H, Moy SS, Lauder J, Crawley JN, Threadgill DW, Wright FA, Magnuson TR: **Large-scale gene expression differences across brain regions and inbred strains correlate with a behavioral phenotype**. *Genetics* 2006, **174**(3):1229-1236.
3. Drake TA, Schadt EE, Lusis AJ: **Integrating genetic and gene expression data: application to cardiovascular and metabolic traits in mice**. *Mamm Genome* 2006, **17**(6):466-479.
4. Tabakoff B, Bhave SV, Hoffman PL: **Selective breeding, quantitative trait locus analysis, and gene arrays identify candidate genes for complex drug-related behaviors**. *J Neurosci* 2003, **23**(11):4491-4498.
5. Saba L, Bhave SV, Grahame N, Bice P, Lapadat R, Belknap J, Hoffman PL, Tabakoff B: **Candidate genes and their regulatory elements: alcohol preference and tolerance**. *Mamm Genome* 2006, **17**(6):669-688.
6. Schadt EE: **Novel integrative genomics strategies to identify genes for complex traits**. *Anim Genet* 2006, **37 Suppl 1**:18-23.
7. Bao L, Wei L, Peirce JL, Homayouni R, Li H, Zhou M, Chen H, Lu L, Williams RW, Pfeffer LM *et al*: **Combining gene expression QTL mapping and phenotypic spectrum analysis to uncover gene regulatory relationships**. *Mamm Genome* 2006, **17**(6):575-583.
8. Latchman DS: **Eukaryotic transcription factors**, 4th edn. San Diego: Elsevier Academic Press; 2004.
9. Wasserman WW, Sandelin A: **Applied bioinformatics for the identification of regulatory elements**. *Nat Rev Genet* 2004, **5**(4):276-287.
10. Elnitski L, Jin VX, Farnham PJ, Jones SJ: **Locating mammalian transcription factor binding sites: A survey of computational and experimental techniques**. *Genome Res* 2006, **16**(12):1455-1464.
11. Tompa M, Li N, Bailey TL, Church GM, De Moor B, Eskin E, Favorov AV, Frith MC, Fu Y, Kent WJ *et al*: **Assessing computational tools for the discovery of transcription factor binding sites**. *Nat Biotechnol* 2005, **23**(1):137-144.
12. Ho Sui SJ, Mortimer JR, Arenillas DJ, Brumm J, Walsh CJ, Kennedy BP, Wasserman WW: **oPOSSUM: identification of over-represented transcription factor binding sites in co-expressed genes**. *Nucleic Acids Res* 2005, **33**(10):3154-3164.
13. Vlieghe D, Sandelin A, De Bleser PJ, Vleminckx K, Wasserman WW, van Roy F, Lenhard B: **A new generation of JASPAR, the open-access repository for transcription factor binding site profiles**. *Nucleic Acids Res* 2006, **34**(Database issue):D95-97.
14. Yan YL, Willoughby J, Liu D, Crump JG, Wilson C, Miller CT, Singer A, Kimmel C, Westerfield M, Postlethwait JH: **A pair of Sox: distinct and overlapping functions of zebrafish sox9 co-orthologs in craniofacial and pectoral fin development**. *Development* 2005, **132**(5):1069-1083.
15. Bailey TL, Elkan C: **The value of prior knowledge in discovering motifs with MEME**. *Proc Int Conf Intell Syst Mol Biol* 1995, **3**:21-29.
16. Hunter L, Cohen KB: **Biomedical language processing: what's beyond PubMed?** *Mol Cell* 2006, **21**(5):589-594.

Table 1: Correlation of Gene Expression Intensity with Fear Conditioning and Overlap of e- and p-QTLs:

Full Name	Gene Symbol	Gene Location: Chr (Mb)	Correlation Coefficient	Raw P-value	MaxLOD	MaxLOD Location: Chr (Mb)	MaxLOD p value
diazepam binding inhibitor	Dbi	1 (121.94)	-0.4840	0.0262	7.7612	1 (119.81)	0.000
solute carrier family 35, member F5	Slc35f5	1 (127.38)	-0.5541	0.0091	8.1447	1 (126.78)	0.000
troponin I, skeletal, slow 1	Tnni1	1 (137.61)	-0.4859	0.0255	6.3863	1 (139.07)	0.000
complement component 1, q subcomponent-like 2	C1ql2	1 (122.16)	-0.4387	0.0466	4.9961	1 (139.07)	0.000
contactin 2	Cntn2	1 (134.33)	-0.5358	0.0123	3.7458	1 (139.07)	0.059
minichromosome maintenance deficient 6	Mcm6	1 (130.15)	-0.4525	0.0394	10.1265	1 (139.07)	0.000
SCO cytochrome oxidase deficient homolog 1 (yeast)	Sco1	11 (66.86)	0.4787	0.0281	3.7304	1 (139.07)	0.043
troponin T2, cardiac	Tnnt2	1 (137.65)	-0.5731	0.0066	8.9317	1 (139.07)	0.000
chitinase 3-like 1	Chi3l1	1 (135.99)	0.4426	0.0445	7.3842	1 (142.84)	0.001
kinesin-associated protein 3	Kifap3	1 (165.61)	-0.4426	0.0445	5.3835	1 (166.16)	0.007
SFT2 domain containing 2	Sft2d2	1 (167.01)	-0.4564	0.0376	4.8731	1 (166.16)	0.004
DNA segment, Chr 1, ERATO Doi 471, expressed	5330438I03Rik	1 (168.14)	0.5017	0.0205	7.2918	1 (167.91)	0.001
CD160 antigen	Cd160	3 (96.88)	0.4938	0.0229	4.1059	1 (167.91)	0.017
RIKEN cDNA D130059P03 gene	D130059P03Rik	6 (38.42)	0.4335	0.0496	3.4905	1 (167.91)	0.035
transcriptional adaptor 1 (HFI1 homolog, yeast) like	Tada1l	1 (168.21)	-0.6171	0.0029	5.7770	1 (167.91)	0.002
DNA segment, Chr 1, ERATO Doi 471, expressed	D1Ertd471e	1 (168.15)	-0.4925	0.0233	6.7813	1 (167.91)	0.000
hemicentin 1	Hmcn1	1 (152.32)	0.4400	0.0459	5.7898	1 (167.91)	0.001
microsomal glutathione S-transferase 3	Mgst3	1 (169.20)	0.5082	0.0187	5.9646	1 (167.91)	0.000
synaptotagmin II	Syt2	1 (136.52)	-0.5272	0.0140	5.5733	1 (167.91)	0.002
receptor tyrosine kinase-like orphan receptor 2	Ror2	13 (53.12)	-0.4787	0.0281	5.5965	1 (73.27)	0.001
ethanolamine kinase 2	Etnk2	1 (135.19)	-0.4918	0.0235	4.5544	1 (76.68)	0.013
camello-like 3	Cml3	6 (85.72)	-0.4545	0.0385	5.5566	12 (21.64)	0.003
discs, large (Drosophila) homolog-associated protein 1	Dlgap1	17 (70.42)	-0.5246	0.0146	4.5729	17 (68.52)	0.009
