# Supplementary material for: The PhenoGen Informatics website: tools for analyses of complex traits
Source: BMC Genet. 2007 Aug 30;8:59. doi: 10.1186/1471-2156-8-59 (PMC2034588; doi:10.1186/1471-2156-8-59)
Supplement: Additional file 2 — PhenoGen user manual. This additional file is a user manual for the PhenoGen website. [file 1471-2156-8-59-S2.pdf]

PhenoGen

User Guide

Disclaimer

PhenoGen Informatics hopes that the tools made available will be useful to INIA (and other) investigators in advancing the knowledge about addictions and other disorders. However, since all of these tools rely on the information uploaded by various investigators and from various public databases (such as MGI, Ensembl, NCBI), PhenoGen cannot guarantee the reliability of the data. Similarly, if any of these databases are not functioning properly, such malfunction is expected to affect the results of queries carried out on the PhenoGen website. In the past few years, availability of computational tools based on "Natural Language Processing" has considerably decreased the time needed for high-throughput literature searches. However, users should check the results of the "Literature Search" tool due to various caveats associated with extracting information out of biomedical literature using such computational tools (Hunter and Cohen, 2006, Molecular Cell, 21:589). The PhenoGen website tools use gene symbols and synonyms along with the user-defined keywords to search the PubMed database. For example, gene symbol "Cap1" (which could be either an official gene symbol for "adenylyl cyclase-associated protein 1" – MGI ID: 88262, or a synonym for "protease, serine, 8" – MGI ID: 1923810, or a short-form for "contraception-associated protein 1" – PubMed ID: 11105923) may pull abstracts related to any of these proteins and a gene symbol "Wars" (for tryptophanyl-tRNA synthetase) may get abstracts related to wars (fighting) rather than the actual gene. So please use the tools provided on the PhenoGen website with care and proper reflection and review of the output

Citation for the PhenoGen Website:

PhenoGen Website [Internet]. Aurora (CO): University of Colorado at Denver and Health Sciences Center. PhenoGen Informatics, 2005 - [cited (insert date of access)]. Available from: http://phenogen.uchsc.edu.

Table of Contents

PhenoGen **1**
Overview 1
Getting Started **2**
PhenoGen Website Overview 2
Website Process Flow *3*
Website Start Page 5
Users 6
Basic User *6*
Principal Investigator *6*
Registration 6
Updating Your Information 7
Logging Into the Website 8
Logging Out of the Website 8
Arrays **9**
Arrays Overview 9
Arrays Menu 10
Browsing Arrays 10
Searching Arrays 10
Searching for Arrays *11*
Browsing Arrays (Logged In) *11*
Browsing Arrays (Not Logged In) *13*
Viewing MIAMExpress Experiment Details 14
Viewing MIAMExpress Array Details 14
Uploading Arrays 16
Uploading an Array *16*
Experiments **21**
Experiments Overview 21
Experiment Process Flow *21*
Experiments Menu 23
Viewing an Experiment 24
Working with Experiments and Versions *24*
Viewing Experiment Details *25*
Creating a New Experiment 26
Combine Arrays *26*

Correlation Analysis ... *28*
Quality Control Checks Overview ... 31
MIAMExpress Data Comparison (Step 1) ... *31*
Array Integrity (Step 2) ... *31*
Guidelines for Assessing the Quality of Affymetrix Data ... *32*
Guidelines for Assessing the Quality of CodeLink Data ... *40*
Running a Quality Control Check ... 42
Data Grouping and Normalization ... 43
Normalization Overview ... *43*
Normalization Methods ... *43*
Run Groups Overview ... *47*
Creating Run Groups and Normalizing Data ... 48
Data Analysis ... 50
Filtering ... *50*
Affymetrix Gene Filtering Procedures ... *50*
CodeLink Gene Filtering Procedures ... *51*
Statistical Analysis Overview ... *53*
Applying Filters and Performing Statistical Analysis ... *57*
Viewing Gene Expression Data ... 60
Viewing Gene Lists for Experiments ... 61
Downloading an Experiment ... 61
Gene Lists ... 63
Gene Lists Overview ... 63
Gene Lists Menu ... 64
Viewing a Gene List ... 66
Working with Gene Lists ... *66*
Viewing Gene List Details ... *66*
Creating a Gene List ... 68
Creating a Gene List from an Experiment ... *68*
Manually Entering a Gene List ... *71*
Annotating Gene Lists ... 73
Expression QTL (eQTL) ... *73*
Allen Brain Atlas ... *75*
Basic Annotation Example ... *76*
Advanced Annotation Example ... *77*
Performing Basic Annotation ... *78*
Performing Advanced Annotation ... *79*

Using QTL Tools 80

QTL Tools 80

Defining QTLs 81

Viewing All QTL Lists 82

QTL Query 83

eQTL Explorer 86

Performing Homolog Search 87

Homolog Search Overview 87

Performing Homolog Search 87

Using Promoter Options 89

Promoter Analysis - oPOSSUM Overview 89

Running oPOSSUM 90

Promoter Analysis - MEME Overview 92

Running MEME 92

Upstream Sequence Extraction Overview 94

Running Upstream Sequence Extraction 94

Genetic Variations Overview 96

Retrieving Genetic Variation Data 97

Performing Literature Search 98

Literature Search Overview 98

Co-reference Analysis 98

Performing a Literature Search 98

Viewing Interpretation Results by Gene List 100

Comparing Gene Lists 101

Uploading a Gene List 102

Downloading a Gene List 103

Cloning and Mutating a Gene List 104

Deleting a Gene List 106

Viewing User Access to a Gene List 107

Results 109

Viewing Interpretation Results Overview 109

Results Menu 109

Viewing Quality Control Results 110

Quality Control Results 110

Deleting an Array During Quality Control 110

Quality Control Results Affymetrix Example 111

Quality Control Results CodeLink Example 113

Viewing oPOSSUM Results ... 115
oPOSSUM Results ... *116*
Deleting an oPOSSUM Analysis ... *118*
Viewing MEME Results ... 119
MEME Description Results ... *120*
Deleting a MEME Analysis ... *121*
Viewing Upstream Sequence Extraction Results ... 122
Upstream Sequence Extraction Results ... *122*
Deleting an Upstream Sequence Extraction ... *123*
Viewing Literature Search Results ... 124
Literature Search Name Results ... *125*
Deleting a Literature Search ... *126*
Principal Investigator ... 127
Principal Investigator Overview ... 127
Principal Investigator Menu ... 128
Approving Array Requests ... 129
Approving Registration Requests ... 130
Approving or Denying a Registration Request ... *130*
Granting Array Access ... 131
Granting Individual Access ... *131*
Granting Open Access ... *132*
Publishing an Experiment ... 133
Creating a MAGE-ML File ... *133*
Submitting to ArrayExpress ... *134*
Security ... 135
Security Overview ... 135
Accessing Data ... 135
Arrays ... *135*
Experiments ... *135*
Gene Lists ... *136*
Supplementary Information ... 137
Reporting Problems, Suggestions and Comments ... 137
Creating a New Issue in JIRA ... *137*
Attaching Files or Screenshots in JIRA ... *140*
Additional Quality Control Sources ... 141
All About R ... 142
Viewing the R Project Homepage ... *142*

Viewing R Manuals *142*

MIAME Overview 143

Promoter Extraction Tools 144

Glossary 149

Appendix A – eQTL Explorer Software User Guide 151

1. Logging In 154

Menu Bar *154*

2. Main Page Tab 154

Fetching (Retrieving) Data *155*

Changing max eQTL size *155*

3. Advanced Options Tab 156

Probeset Options *156*

pQTL Options *156*

4. Warnings Tab 157

5. Export Tab 158

6. The Genome Panel 159

7. Chromosome Panel 160

Visualizing eQTL Information in Table Format *160*

Viewing eQTL and pQTL Annotations *161*

8. Troubleshooting 165

9. Additional Information 165

Contact Us *165*

System Requirements *165*

Licence *165*

Disclaimer *165*

Index 167

PhenoGen

Overview

The PhenoGen Informatics website (http://phenogen.uchsc.edu) is a comprehensive toolbox for storing, analyzing, and integrating microarray data and related genotype and phenotype data. The site is particularly suited for combining QTL and microarray data to search for "candidate" genes contributing to complex traits. In addition, the site allows, if desired by the investigators, storage and sharing of data. Investigators can conduct "*in-silico*" microarray experiments using their own and/or "shared" data.

The PhenoGen toolbox was originally created to facilitate interactions within the INIA consortium of investigators. In brief, the goals and purpose of the **INIA** (**I**ntegrative **N**euroscience **I**nitiative on **A**lcoholism, http://www.scripps.edu/np/inia/index.html) consortium is to identify the molecular, cellular, and behavioral neuroadaptions that occur in the brain reward circuits associated with the extended amygdala and its connections as a result of exposure to ethanol. Although PhenoGen web tools were initially created for the consortium members, the integrated tools described here can be accessed by the global scientific community.

Getting Started

Before you can use the PhenoGen website, you must **register** and set up a **user profile**. Your user profile provides details such as your name, email address, and the Principal Investigator you are working with. Your user profile can be modified at any time when you are logged into the website. After your registration is complete, you can use the website to create and analyze experiments. The website is available at **http://phenogen.uchsc.edu/**

See the topics *Registration*, *Logging Into the Website*, and *Logging Out of the Website* for details.

PhenoGen Website Overview

The PhenoGen website shares experimental data within the INIA West Consortium, as well as a worldwide community of investigators and provides a flexible, integrated, multi-resolution repository of neuroscience data, ranging from molecules to behavior, for collaborative research on alcoholism.

In addition to providing a comprehensive system to organize, query, analyze, and retrieve high-throughput gene expression data, the website provides the Consortium members with computational tools for integrated analysis of neuroscience data, biomedical literature, gene functional annotations, and Quantitative Trait Loci (QTLs).

The PhenoGen website allows you to:

- Upload microarray raw data using the PhenoGen copy of MIAMExpress, a web-based tool for gene expression data submissions to ArrayExpress.
- Upload gene lists.
- Share data with other INIA investigators.
- Search literature.
- Translate gene identifiers to and from multiple databases.
- Access databases containing behavioral QTL data.
- Match physical location of genes of interest and their eQTL to behavioral QTLs.
- Correlate gene expression with a phenotype using multiple data sets.

You can also perform:

- Microarray data quality control analysis and normalization.
- Data filtering (noise filtering).
- Statistical analyses, including most common statistical tests and permutations.
- Promoter analysis (Transcription factors).
- Obtain information about genetic variations (e.g. SNPs or polymorphisms) in the transcripts of interest.

Website Process Flow

After you have registered and logged into the PhenoGen website, there are two process flows: Combine Arrays and Correlation Analysis

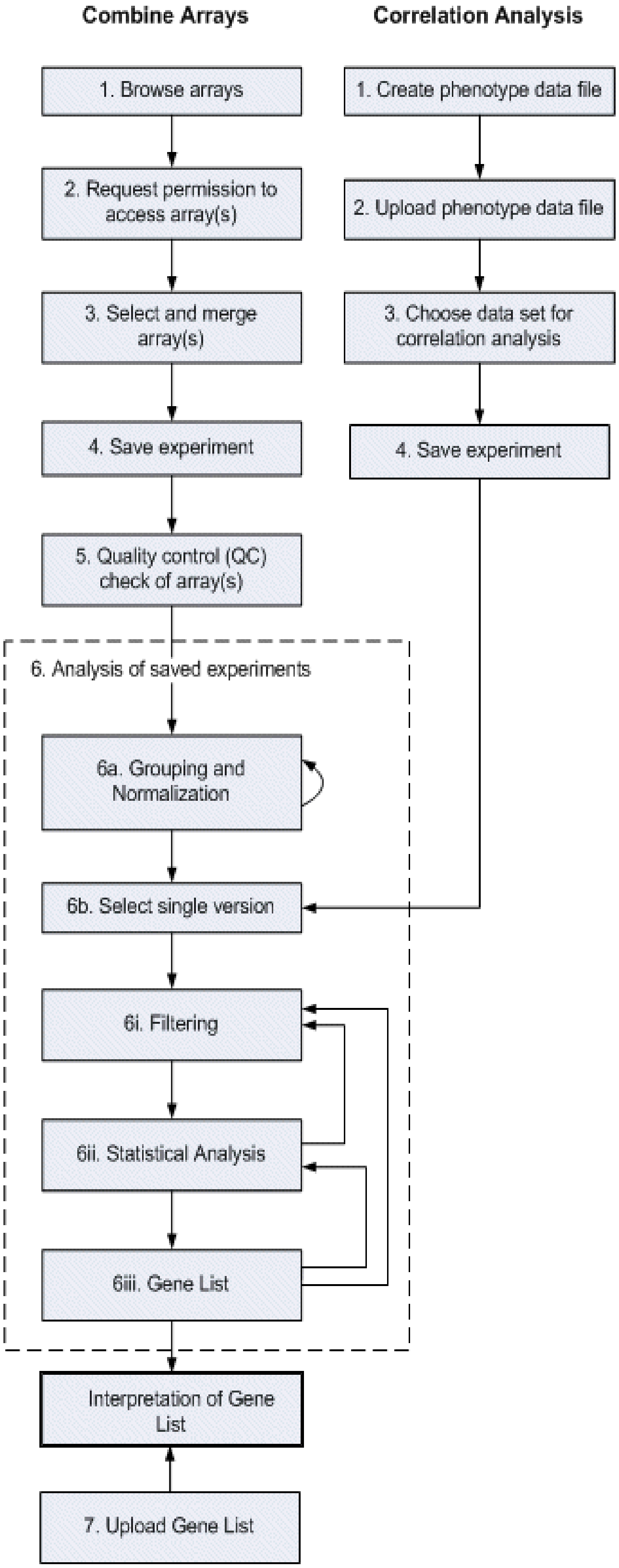

Combine Arrays

Working with Arrays

Upload microarray data using MIAMExpress. If you have an existing experiment, you can upload it using MIAMExpress. See the glossary for explanations of the difference between MIAMExpress experiments and PhenoGen website experiments.

5. Browse arrays.
6. Request permission to access arrays.

Working with Experiments

7. Select and merge arrays.

   Note: You can only select and merge data you have been given permission to access. See *Browsing All Arrays (Logged In)*.

8. Save the experiment.
9. Apply quality control measures to the merged arrays.

Working with Experiment Versions

10. Analyze saved experiments:
    a. Group and normalize, including naming and saving the normalized grouping (creating versions).
    b. Select a single version.
        i. Filter genes.
        ii. Perform statistical analysis.
        iii. Save gene lists.
11. Upload a gene list. If you have an existing gene list to interpret, you can upload it to the PhenoGen website.

Interpretation of Gene Lists

After saving or uploading gene lists, you can interpret them. At certain points in the process, you can return to a previous page to redo an action or repeat an action to further narrow the ultimate gene list results. At any point in the flow, you can abandon your current course of action and return to the beginning to browse arrays.

Note: If you choose to return to the browse arrays step and you have not saved a gene list (step 6iii), any actions performed after grouping and normalization (step 6a) are lost.

After you have grouped your experiment arrays and performed normalization, there are opportunities within Filtering, Statistical Analysis, and Gene Lists to return to a previous step and further narrow your criteria.

Correlation Analysis

Creating and Uploading Phenotype Data Files

You can create phenotype data files to correlate with gene expression data that is currently in the PhenoGen website.

1. Create behavior phenotype data file (a text file created off line).
2. Upload behavior phenotype data file.

Working with Experiments

3. Choose data set for correlation analysis.
4. Save the experiment.

Working with Experiment Versions

When you are performing Correlation Analysis, you do not have to apply quality control measures (step 5) or group and normalize (step 6a). After you save the experiment, begin at step 6b, select a single version.

Website Start Page

The PhenoGen website *Start* page provides you with the opportunity to:

- Register to use the website.
- Login to the website.
- Retrieve a forgotten password.

The **Current Content** box displays the existing number of arrays available at the PhenoGen website, as well as the array count by organism. You can click the numbers next to each option to open the *Browse All Arrays page (not logged in)* where you can view details about the selected organism.

Click the > to the left of the organism name (e.g., > **Mouse**) to display a drop-down list that shows the categories of the organism that are in the database, and the number of arrays in each category.

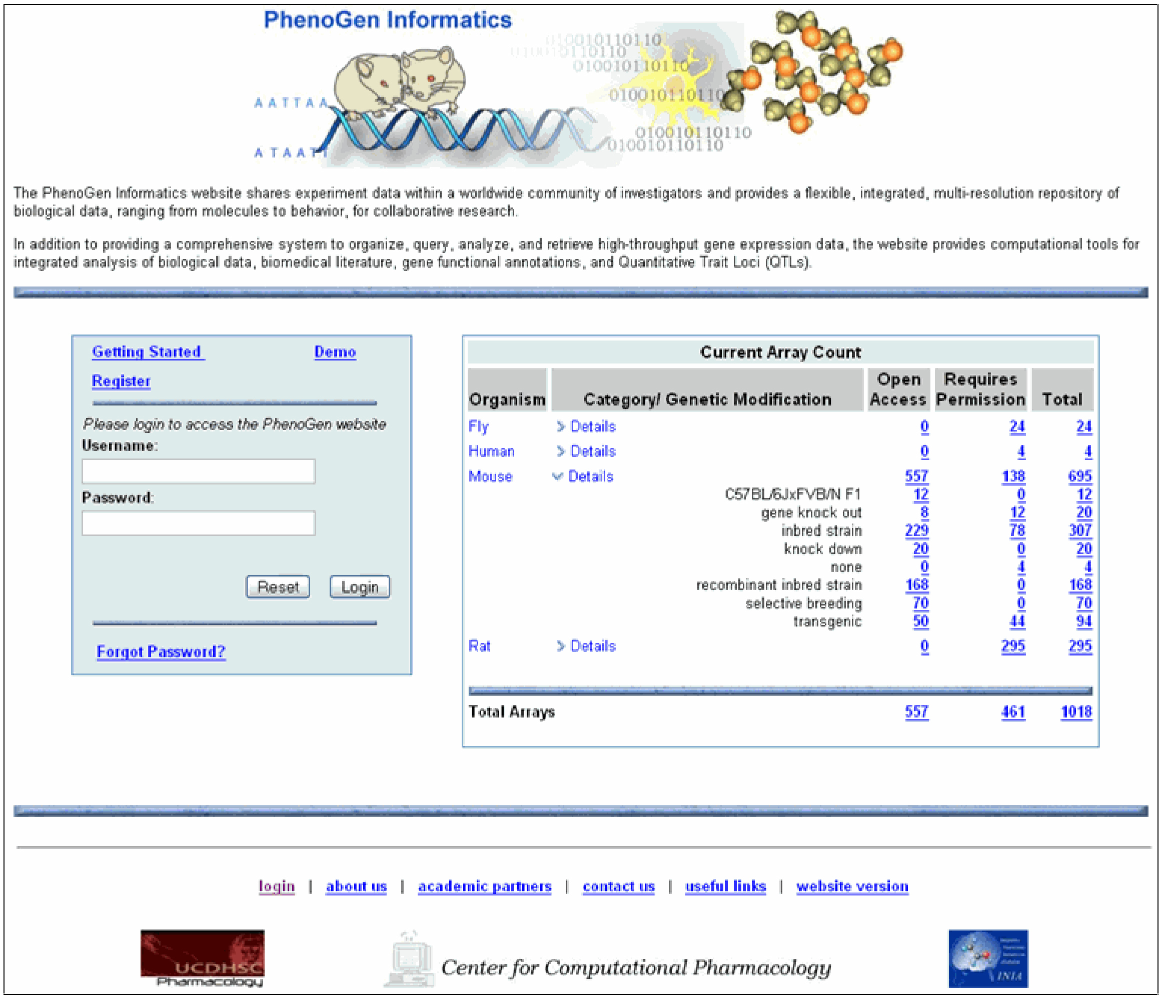

Users

There are two types of users in the PhenoGen website:

- Basic User (default)
- Principal Investigator (PI)

See *Registration* for details.

Basic User

Most users are basic users. These users, upon approval from a PI, can:

- Upload experiment arrays.
- View arrays and experiments.
- Create experiments.
- Download experiments.
- Access all of the available data analysis tools.

Principal Investigator

The Principal Investigator is often the head of a lab and is responsible for granting permission to other users to view the arrays. A user who is a Principal Investigator sees a Principal Investigator button on the Main page. This button provides administrative access for the PI. In addition to all of the functions available to basic users, the PI can:

- Approve array requests.
- Approve registration requests.
- Grant array access to an individual.
- Grant open access to array data.
- Create MAGE-ML files.
- Publish MAGE-ML files to ArrayExpress.

Registration

The Registration page must be completed before you can log into the PhenoGen website.

1. Click **Register** on the *Start* page. The *Register* page displays.

   Note: Required fields have an asterisk *.

2. Select your **Title** and enter your **First Name**, **Middle Initial**, and **Last Name**.
3. Enter a **Username**. If the username you enter is the same as an existing name, an error message displays when you try to register.

4. Enter a **Password**, then re-enter the password. Your password must be at least six but not more than 16 characters and contain numbers, letters, and special characters (~!@#$%^&*()+).

   Note: Your Username and Password are used to log in to the website.

5. Enter your contact **Email**, **Phone**, and **Fax**.
6. Enter the **Name** of your institution, your **Department**, and your **Box** number.
7. Enter your **Address**.
8. Click **Register**. A pop-up dialogue displays, with the terms of the PhenoGen website. If you agree to the terms, click **OK** to send your request for registration. Click **Cancel** to cancel your registration if you do not agree to the terms.

If your registration request is successful, a page displays that informs you that your submission was successful and the submission will be reviewed within 24 hours.

See the topic *Updating Your Information* for instructions on changing your registration details. The only detail you cannot change is your username.

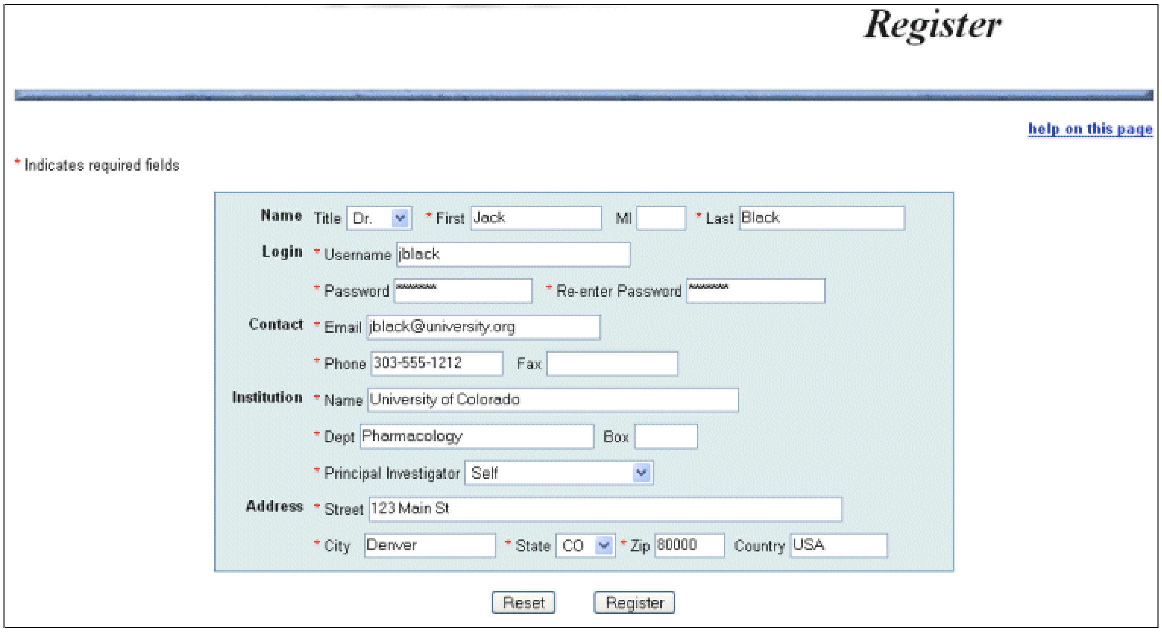

Updating Your Information

If you need to update the information you provided in the *Registration* page:

1. Click **Update User Profile** in the bottom menu. The *User Update* page displays.

   Note: Required fields have an asterisk *.

2. Update your information as required. You cannot change your username.
3. Click **Update**. Your information is updated. You can also click **Reset** to return all the fields to their original values.

Logging Into the Website

Before you can log into the PhenoGen website, you must register and receive approval for your registration. After your registration is approved:

1. Open your internet browser.
2. Type **http://phenogen.uchsc.edu** in the address field. The PhenoGen website displays.
3. Enter your **Username**.
4. Enter your **Password**.
5. Click the **Login** button below the Password field to log in. The *Home Page* displays.

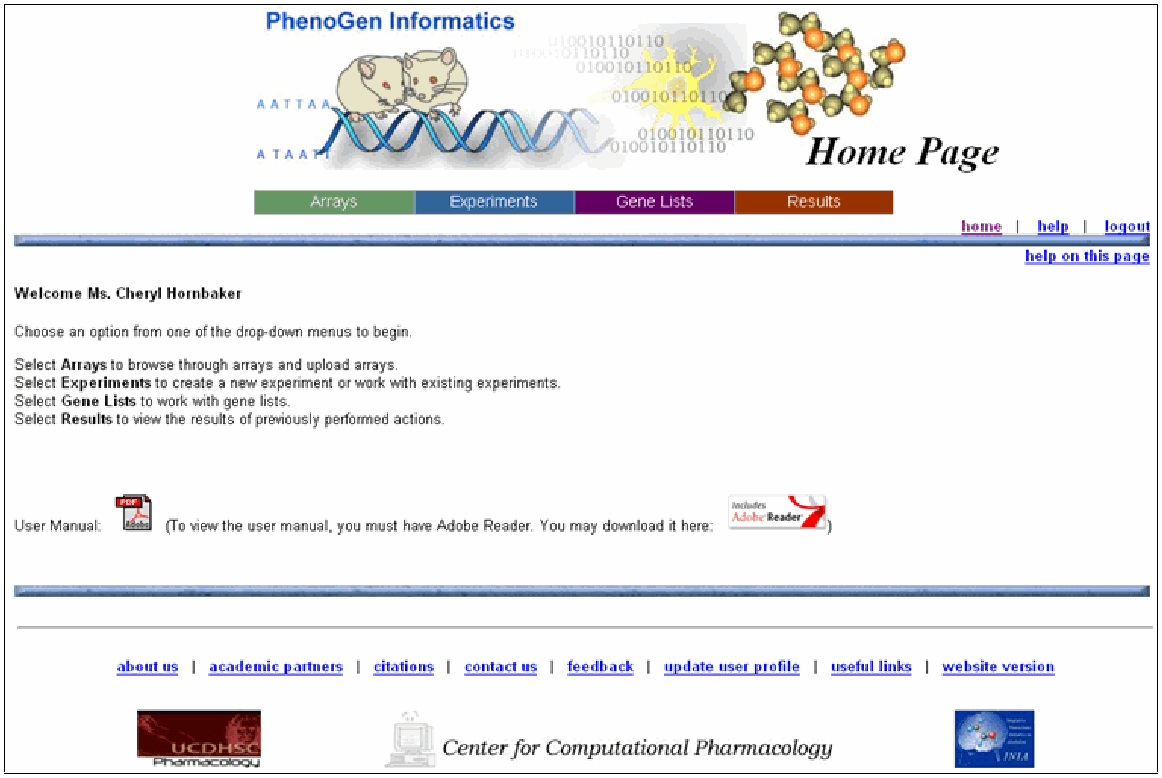

Logging Out of the Website

When you are done entering data, creating experiments, and analyzing data, you should log out of the website. Logging out lets the website know that you are finished working and closes your connection to the website. You can log in again at any time.

- Click **Logout** at the top right corner of the page to log out of the PhenoGen website.

Arrays

Arrays Overview

Arrays in the PhenoGen website can be viewed and combined, for an "in-silico" experiment, in numerous ways. The PhenoGen website allows you to take arrays from multiple MIAMExpress experiments and combine them into different experiments for statistical analysis.

Note: The PhenoGen website uses a local copy of MIAMExpress, and data entered is only available on the PhenoGen website.

The definition of an experiment differs between MIAMExpress and the PhenoGen website.

Experiment Type	Description
MIAMExpress Experiment	A group of arrays that are assayed at the same time. Information about the experimental protocol is entered using MIAMExpress. Each array in the experiment represents a hybridization and is linked to a sample.
PhenoGen Experiment	An "in-silico" experiment created by a user using a collection of arrays from one or more MIAMExpress experiments OR a behavioral phenotype list to be correlated with the gene expression of a PhenoGen data set.

In the PhenoGen website, each **array** is annotated by a MIAMExpress annotation document; the *Array Details* page. The annotation document provides details such as species, gender, and array type. The PhenoGen website allows you to look at single arrays or collect arrays into groups that you define. These groups can then be used in an experiment where you can use the tools provided by the website to make comparisons between the groups you created. Such an analysis results in a gene list of differentially expressed genes.

In the PhenoGen website, arrays to be viewed can be filtered within a species by:

- Category (e.g., inbred)
- Sex
- Tissue (e.g., brain)
- Treatment (e.g., naive)
- PI (including an option for all of the public data)

Filtering is not required, but you can filter on one, or multiple, attributes. Specific arrays can also be searched for by the name or part of the name in the **Array Name Contains** field.

Arrays Menu

The *Arrays* menu displays when you move your mouse over the **Arrays** button.

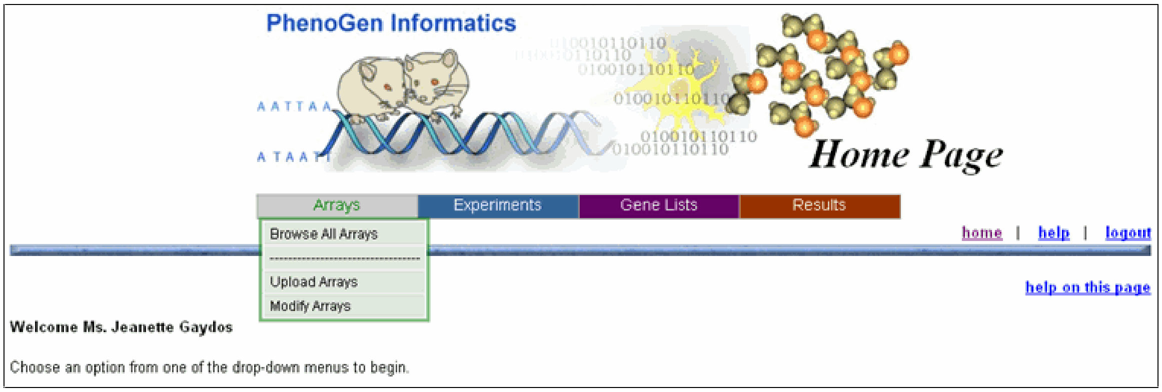

Drop-down Menu Option	Description
Browse All Arrays	Allows you to browse all arrays that exist in the MIAMExpress application. When you are logged into the website you can use this page to select arrays that you want permission to use. A request for access is sent to the PI who is responsible for the data.
Upload Arrays	Launches the MIAMExpress website for you to upload an array.
Modify Arrays	Allows you to select the MIAMExpress experiment that you want to modify. When Modify is selected, preprocessing occurs (the experiment status is changed from curated to pending) and the MIAMExpress website is launched. **You can only modify experiments that you uploaded.**

Browsing Arrays

You can browse all arrays from the *Start* page without logging in. Or you can browse arrays after you are logged in by selecting **Arrays menu** > **Browse All Arrays**. To proceed beyond browsing the arrays, you must log into the PhenoGen website. After you have logged into the website, you can browse the arrays AND:

- Request permission to access arrays.
- Combine arrays to which you have access into your own experiment.

Note: Array data is the responsibility of the Principal Investigator (PI). You must request permission from the PI to access an array which is not part of the open access data.

Searching Arrays

Whether you open the *Browse All Arrays* page when you are logged into the PhenoGen website or when you are not logged in, you can use search functionality to narrow your search results. You can search by Organism, Category, Sex, Tissue, Treatment and PI. Each selection limits the number of results that are returned. Specific arrays can also be searched for by typing the name or part of the name in the **Array Name Contains** field.

Searching for Arrays

Your choices in the Search drop-down lists are based on the experiments that have been uploaded into MIAMExpress.

Notes:

- You can leave all search terms set as **All** to return all the arrays in the database.
- You can narrow your criteria using the drop-downs.
- You can set the **PI** drop-down menu to **Public Data** to return publicly available arrays.
- You can search for specific arrays by typing the array name or part of the array name in the **Array Name Contains** field.

1. Select the **Organism**.
2. Select the **Category**.
3. Select the **Sex**.
4. Select the **Tissue**.
5. Select the **Treatment**
6. Select the **PI**.
7. *OPTIONAL* - Type in the whole or partial array name in the **Array Name Contains** field.
8. Click **Find Arrays**. Any arrays that match the criteria you specified are displayed. If you require access to an array, see *Browse All Arrays*.

Browsing Arrays (Logged In)

After you log in, the *Browse All Arrays* page includes a **Request Access** column that allows you to request permission to use specific arrays. Open access arrays do not require permission to use and say *Public* in the **Request Access** column. When requesting access, you can select an array or a subset of arrays. When you submit a request for permission to use an array (or multiple arrays), an email that requests access permission is sent to the PI who is responsible for the data.

1. Select **Arrays menu** > **Browse All Arrays**. The *Browse All Arrays* page displays.
2. Use the **Search** drop-down lists to narrow the array list. See the topic *Searching Arrays* for details.
3. Click **Find Arrays**. The arrays that match your search criteria display.

   Notes:

   - You can change the number of arrays that display. Enter the number of arrays you want to display in the **Display [number] arrays per page** section, then click **Go**.
   - You can sort the arrays by clicking on the column headings in blue.

4. *OPTIONAL* - Click the **Array Name** to open the *Array Details* page, to view details about the MIAMExpress array.
5. *OPTIONAL* - Click the **MIAMExpress Experiment Name** to open the *Experiment Details* page and view the MIAMExpress experiment.

6. Click the checkbox(es), in the **Request Access** column, beside the array(s) for which you would like to request access permission.

Notes:

- Open access data says *Public* in the **Request Access** column.
- If you have access to an array, the **Request Access** column contains an X.
- If you have requested access and have not yet been granted access, the column says *Pending*.
- If you requested access and access was denied, the column says *Denied*.

7. Click **Request Array Access**. An email is sent to the Principal Investigator who is responsible for the array. When you receive permission to use the array, it displays in the *Create New Experiment* page. See the topic *Creating a New Experiment* for details.

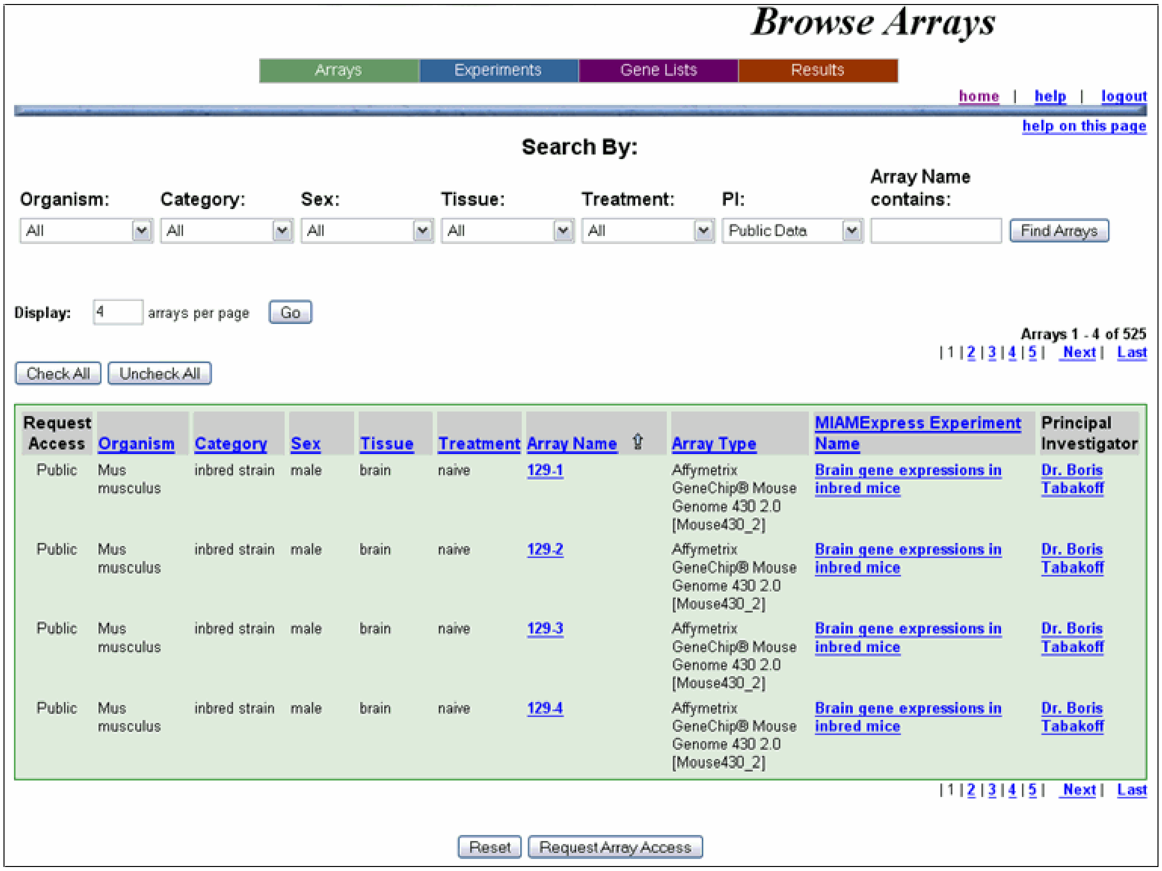

Browsing Arrays (Not Logged In)

From the *Start* page:

1. Click on the array number in the Current Content box beside the organism for which you want to view arrays. The *Browse Arrays* page displays.
2. Use the **Search** drop-down lists to narrow the array list.

After determining that there are arrays in the database that you want to use to create an experiment, you can log in, browse all arrays, and request access to arrays you want to use. See the topic *Browsing Arrays (Logged In)* for more information.

Viewing MIAMExpress Experiment Details

The MIAMExpress Experiment Details page allows you to view details about the MIAMExpress experiment, such as name, description, arrays, platform, etc. that are part of the experiment.

1. Select **Arrays menu** > **Browse All Arrays**. The *Browse All Arrays* page displays.
2. Use the **Search** drop-down lists or type in array name search criteria to narrow the array list. See the topic *Searching Arrays* for details.
3. Click **Find Arrays**. The arrays that match your search criteria display.
4. Click a **MIAMExpress Experiment Name** to open the *Experiment Details* page, to view details about the experiment.

See the topic *Uploading Arrays in MIAMExpress* for details on uploading an experiment into MIAMExpress.

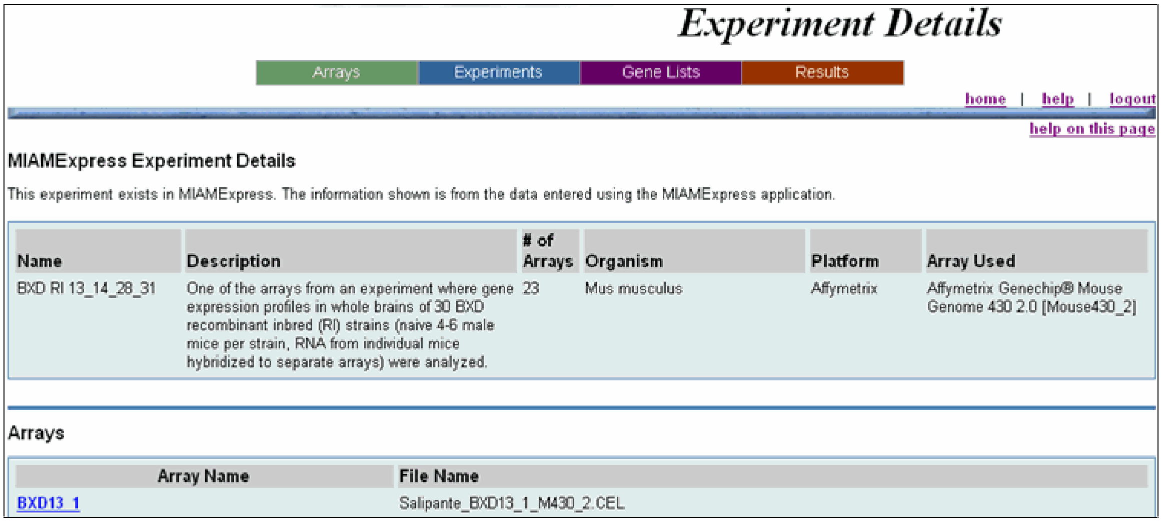

Viewing MIAMExpress Array Details

The MIAMExpress Array Details page allows you to view details about the MIAMExpress sample (array).

1. Select **Arrays menu** > **Browse All Arrays**. The *Browse All Arrays* page displays.
2. Use the **Search** drop-down lists or type in array name search criteria to narrow the array list. See the topic *Searching Arrays* for details.
3. Click **Find Arrays**. The arrays that match your search criteria display.
4. Click the **Array Name** to open the *MIAMExpress Array Details* page, to view details about the sample and array.

Array Details

Arrays | Experiments | Gene Lists | Results

home | help | logout

help on this page

Sample Details

**Sample Name:**	BXD31_1
**Organism:**	Mus musculus
**Gender:**	male
**Sample Provider:**	Jackson Laboratory
**Development Stage:**	adult **Age:** specified **Min:** 10 **Max:** 12
**Unit:**	weeks
**Initial Time Point:**	not applicable
**Organism Part:**	brain
**Genetic Modification:**	recombinant inbred strain
**Individual Identifier:**	null
**Individual Genotype:**	null
**Disease State:**	null
**Separation Technique:**	not applicable
**Target Cell Type:**	null
**Cell Line:**	BXD31/TyJ
**Strain:**	BXD31/TyJ
**Additional Clinical Information:**	null
**Treatment:**	naive
**Platform:**	Affymetrix
**Array Used:**	Affymetrix Genechip® Mouse Genome 430 2.0 [Mouse430_2]
**Growth Conditions Protocol:**	null
**Growth Conditions Protocol Description:**	null
**Sample Treatment Protocol:**	RI_Sample_treatment
**Sample Treatment Protocol Description:**	Naive male mice, 10 to 12 weeks of age, were sacrificed whole brain was removed and stored at -80 C. Total RNA was extacted using the protocol described in "Sanjiv3"

Experiment Details

**Submission Description:**	BXD RI 13_14_28_31
**Experiment Description:**	One of the arrays from an experiment where gene expression profiles in whole brains of 30 BXD recombinant inbred (RI) strains (naive 4-6 male mice per strain, RNA from individual mice hybridized to separate arrays) were analyzed.
**Submitter:**	bhaves

Extract Details

**Extract Name:**	BXD31_1
**Extract Protocol:**	Sanjiv3
**Extract Protocol Description:**	RNA Extractions for GeneChip Assay using Rneasy Lipid Tissue kit RNA extraction using QIAzol: 1. A 4 ml of QIAzol reagent. Do not allow tissue to thaw before addition of QIAzol reagent. 2. Homogenize with Polytron for 60 seconds at setting 4. 3. Let samples sit at RT for 5 minutes. 4. Add 1.0 ml chloroform per sample. 5. Cap tubes securely and shake vigorously by hand for 15 seconds. 6. Let sit at RT 3 minutes. 7. Centrifuge @ 5,000 x g for 15 minutes at 4oC. 8. Transfer the aqueous phase to an eppendorf tube (1 ml/tube). 9. Add 1 volume of 70% ethnol (about 3 ml) mix thoroughly by vortexing. Continue without delay with Rneasy Midi columns. B. RNeasy Midi Kit 1. Add 4 ml of the sample onto midi Spin column. Close the tube and centrifuge at 5000 x g for 5 min at room temp. Discard the flowthrough. 2. Repeat the step 1 with remainder of the sample. Discard the flowthrough. 3. Add 4.0 ml of Buffer RW1 onto the spin column and centrifuge at 5000 x g for 5 min to wash the column. Discard the flowthrough. 4. Add 2.5 ml of RPE Buffer to the spin column. Close the tube and centrifuge at 5000 x g for 2 min. Discard the flowthrough. 5. Add another 2.5 ml of RPE Buffer to the spin column. Close the tube and centrifuge at 5000 x g for 5 min. Discard the flowthrough. 6. Place column in fresh tube. Add 150 il of Rnase free water to elute total RNA. Let the column and the tubes stand for 10 min at room temp and then centrifuge at 5000 x g for 5 min. 7. Repeat the elution with the flow through (150 il). 8. Take Ods at 260 and 280 nm on the scanning spectrophotometer in AFFY core lab .

Labeled Extract Details

**Label Extract Name:**	BXD31_1
**Label Extract Description:**	null
**Label Extract Protocol:**	HAP and LAP
**Label Extract Protocol Description:**	www.affymetrix.com

Hybridization Details

**Hybridization Name:**	BXD31_1
**Hybridization Description:**	null
**Hybridization Protocol:**	Affy MOE430v2
**Hybridization Protocol Description:**	WWW.AFFYMETRIX.COM
**Scanning Protocol:**	Affymetrix 430v2
**Scanning Protocol Description:**	www.Affymetrix.com

File Details

**File Name:**	Salipante_BXD31_1_M430_2.CEL

Uploading Arrays

All arrays available in the Browse page are pulled from experiments entered in MIAMExpress.

When you are logged into the PhenoGen website, you can upload microarray data (arrays). Each time an array is uploaded, it is automatically assigned to the Principal Investigator associated with the user who uploads the data.

Uploading an Array

1. Select **Arrays menu** > **Upload Arrays**. The *Upload Arrays* page displays.
2. Click the **MIAMExpress** link to launch the MIAMExpress application. MIAMExpress launches in a new browser window.

   Notes:

   - The PhenoGen website uses a local copy of MIAMExpress, and data entered is only available on the PhenoGen website.
   - The PhenoGen website remains open in its own browser window.

3. Enter your **PhenoGen Username**.
4. Enter your **PhenoGen Password**.
5. Click **Login** to log into MIAMExpress.

   Note: Click HELP in the MIAMExpress application for help entering data in MIAMExpress..

Uploading an Experiment in MIAMExpress

These instructions provide an overview of how to upload an experiment in MIAMExpress. **They do not provide complete step-by-step instructions.** For complete details, review the help provided in the MIAMExpress application.

Submitting an Experiment

You do not have to enter all details for an experiment at one time, since MIAMExpress allows you to enter experiments over a period of time. Until you choose to complete your submission, an experiment retains a *Pending* status and is editable. When you complete the submission, the experiment status is changed to curated or submitted and you can no longer edit it.

6. Click **Experiment Submission**. You can create a new submission. Or, you can choose a Pending submission and click **View/Edit** to enter more information for that submission.
7. Click **Create**. The *Experiment Design* page displays.
8. You must completely fill in the top section of the *Experiment Design* page, since each field is required.
9. In addition to your experimental factors, you must choose **Other** in the **Experimental factors** field and type **Treatment** in the space provided. You can do this by holding down **CTRL** and

selecting the experimental factors. Treatment becomes a field name in subsequent pages. In the *Sample Description* page, you can enter naive or treated in the *Treatment* field.

Notes:

- You should enter quality control information for any microarrays that failed quality control in the **Experiment Description** field.
- The **Experiment design type** and **Experimental factors** fields allow you to select multiple options. Hold down the **CTRL** or **Shift** key, and then click with the left mouse button to select.

10. Click **Submit** to submit details in the top section. Many sections contain a *Submit* button so you can save data as you enter it.
11. Click **Publication** to enter details about publications that might be associated with your experiment. This field is mandatory.
12. Enter the **Publication Status**.
13. Click **Submit**.

Creating Samples for an Experiment

Tip: Once you have named your sample, MIAMExpress will automatically name any extracts bound to the sample as E_Sample Name, any labeled extracts bound to it as L_Sample Name, and any hybridizations bound to it as H_Sample Name. This can be changed, however leaving these names makes it easier to see associations.

14. Select the number of samples that are in your experiment. If the number is more than ten, select **10+** and enter the number.
15. Click **Create**. The appropriate number of samples are created with generic names.
16. Select a sample then click **View/Edit**. The *Sample Description* page displays.
17. Edit the sample name and details, filling in as much information as you can. Data must be entered in the following fields in MIAMExpress to ensure arrays display correctly in the PhenoGen website:

- **Sample Name**
- **Organism**
- **Gender**
- **Sample Type**
- **Organism Part**
- **Genetic Modification -** If you do not find the appropriate modification, select **Other**, and then enter details. Note that the drop-down list provides options that become searchable in the PhenoGen website *Browsing arrays* page.
- **Cell line** - Please enter the full name of the strain or line (e.g., C57BL\6).
- **Strain -** Please enter the full name of the strain or line (e.g., C57BL\6).
- **Treatment -** Please enter either **treated** or **naive**, then enter **n/a** in the **Unit** field. If there has been a treatment, please create a **Treatment Protocol** detailing the treatment, and bind the sample to it. Additional information can also be placed in the **Additional Clinical**

Information field. Note that the *Treatment* field provides options that become searchable in the PhenoGen *Browsing arrays* page.

18. *OPTIONAL* - If your samples are all similar, click **Paste this sample description across all samples**. All the data you entered, except the sample name, strain, and treatment is pasted into the *Sample Description* page for each sample. You can edit each sample to change the name and minor details, such as strain or cultivar.
19. Create protocols for the samples. You can create **Growth Condition** and **Sample Treatment** protocols.
20. Click **Submit**.

Creating Extracts for the Samples

21. Click **Extracts (create/view/edit)** to create extracts for the samples. The *Extract* page displays, where you can enter all the extracts for your experiment.
22. Select the number of extracts that are in your experiment. If the number is more than ten, select **10+** and enter the number.
23. Click **Create**. The appropriate number of extracts is created with generic names.
24. Link each extract to a sample or multiple samples. MIAMExpress will create a new name for each extract, referencing the sample it is bound to.
25. Click **Submit**.
26. Select an extract then click **View/Edit** to change the extract name, if desired, and link the extract to a protocol.
27. *OPTIONAL* - Click **Create new extraction protocol** or **Create new pooling protocol** to create new protocols. Fill in the required information on the page that displays, then click **Submit**.
28. Click **Submit**.
29. Select the number of labeled extracts that you want to add to the current extract. If the number is more than ten, select **10+** and enter the number.

    Note: Labeled extracts are ONLY available from the *Extracts* page.
30. Click **Create**. The appropriate number of labeled extracts is created with names referencing the sample it is linked to.

    Note: You cannot move on to hybridization unless you create labeled extracts.
31. Select a labeled extract then click **View/Edit** to change the name and link the labeled extract to a protocol.
32. Click **Submit**.
33. *OPTIONAL* - Click **Create new labeling protocol** to create a labeling protocol.

Tip: Use the Navigation bar at the top of the page to return to previous screens!

Creating Hybridizations

After you have completed the annotation of all samples, extracts, and labels, you can proceed to Hybridization.

34. Click **To Hybridizations** on the *Labeled Extract* page. The *Create Hybridizations* page displays.
35. Select the number of hybridizations that you want to create. If the number is more than ten, select **10+** and enter the number.
36. Click **Create**. The appropriate number of hybridizations is created with generic names.
37. Select the **Array design name**, enter the **Serial number**, then select the **Labeled extract name** for each hybridization. If you are using an array that is not listed in the Array design name drop-down, please contract the system administrator.
38. Click **Submit**.
39. The hybridizations will be renamed to reflect the sample it is linked to.
40. Select a hybridization, and then click **Upload data files** to change the name and link the hybridization to a raw data file and to hybridization and scanning protocols.

    Notes:

    - Modify the Hybridization Name to reflect failure if the quality control for the microarray failed.
    - When you analyze arrays in the CodeLink software, make sure you save as GENESPRING to generate a .txt file. This creates a raw data file you can link to in the Hybridization page. The brackets in the file names must be removed before uploading the .txt data files
    - Make sure you enter your quality control method in the Scanning protocol list. Use the Create new scanning protocol button.
    - If uploading an Affymetrix .CHP file, a normalization protocol must be selected.

41. Click **Submit**.
42. Return to the *Create Hybridizations* page.

Completing the Submission

When you are sure you have entered all pertinent details for your experiment:

41. Click **Continue** under *Final Step* on the *Create Hybridizations* page. This opens the *Complete Submission* page.
42. Click **Complete submission**. The experiment status is changed from **Pending** to **In curation/Submitted**.
43. Close MIAMExpress

See the topic *MIAME Overview* in the *Supplementary Information* section for details about the Minimum Information About a Microarray Experiment.

Experiments

Experiments Overview

An *Experiment* in the PhenoGen website is an "in-silico" experiment created by a user using a collection of arrays from one or more MIAMExpress experiments or a phenotype list to be correlated with the gene expression of a PhenoGen data set. You can create experiments using arrays in the PhenoGen database that are public or that you have been granted access to use. You can alternately create an experiment by creating a behavioral phenotype list and uploading it to be correlated to the whole brain gene expression profiles of one of the PhenoGen data sets.

Experiment Process Flow

The experiment process flow begins after browsing arrays and requesting permission to access arrays (if needed) or after creating a phenotype list. It is comprised of the actions required to create an experiment, the actions required to create an experiment version, and the subsequent extraction of a gene list from the experiment version.

Create an Experiment

Combine Arrays

1. Select and merge arrays.

   Note: You can only select and merge data that is public or that you have been given permission to access. See the topic *Browsing All Arrays (Logged In)*.

2. Save the experiment.
3. Apply quality control measures to the merged arrays.
4. Group and normalize, including naming and saving the normalized grouping (creating versions).

Correlation Analysis

1. Create a behavioral phenotype data file (off line).
2. Upload the phenotype data file. This includes choosing the data set (such as the whole brain gene expression profile of the BXD RI panel of mice) you wish to correlate the behavioral phenotype list to and naming and saving the experiment (creating versions).

Analyze an Experiment

5. Select a single version.
6. Filter genes.
7. Perform statistical analysis.
8. Save gene lists.

Interpret Gene Lists

9. Upload a gene list. If you have an existing gene list to interpret, you can upload it to the PhenoGen website.
10. Interpret the gene lists.

Experiments Menu

The *Experiments* menu displays when you move your mouse over the **Experiments** button.

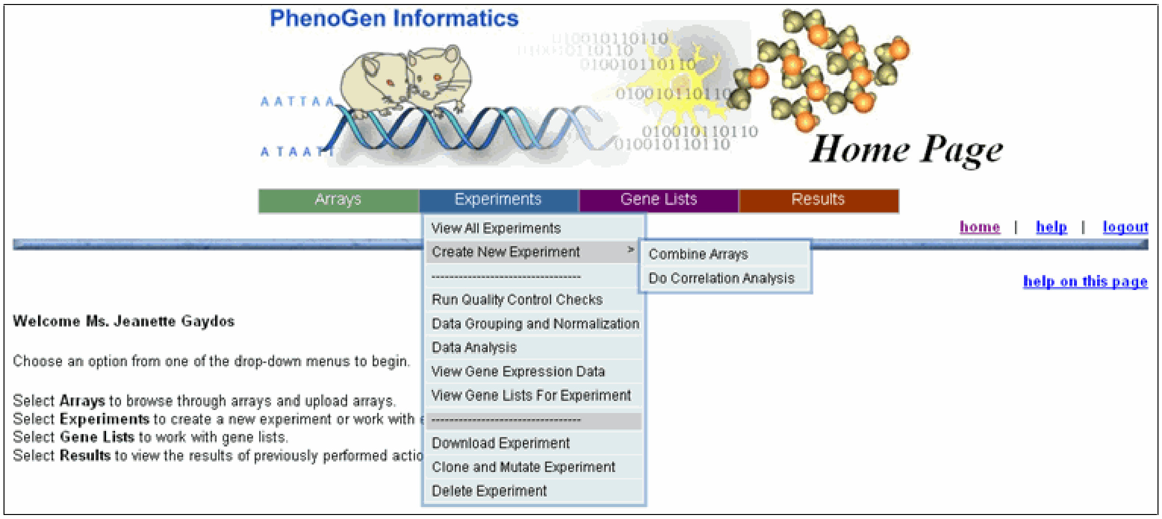

Drop-down Menu Option	Description
View All Experiments	Allows you to view all the experiments that you created in the PhenoGen database.
Create New Experiment	Opens a sub-menu where you can select:<br>• *Combine Arrays* - Allows you to browse arrays for which you have permission, select arrays, and create an experiment.<br>• *Do Correlation Analysis* - Allows you to upload a phenotype list to be correlated with the gene expression of a PhenoGen data set.
Run Quality Control Checks	Allows you to run quality control checks on the arrays you selected for the experiment.
Data Grouping and Normalization	Allows you to create run-groups to be compared statistically and normalize the data for a saved experiment.
Data Analysis	Allows you to run data analysis, including filtering and statistical analysis, on a version (a normalized run-group) of an experiment.
View Gene Expression Data	Allows you to obtain gene intensity expression data for a gene or gene list from a given experiment.
View Gene Lists for Experiments	Allows you to view gene lists for an experiment.
Download Experiment	Allows you to download a saved experiment or a version to your computer.
Clone and Mutate Experiment	Allows you to copy an existing experiment, modify the arrays if necessary, and save the experiment with a new name.
Delete Experiment	Allows you to delete an experiment, which deletes all versions of that experiment, or an experiment version.<br>**You can only delete experiments and experiment versions created by you.**

Viewing an Experiment

Select **Experiments menu** > **View All Experiments** to view your saved experiments and experiment versions.

Working with Experiments and Versions

The *View All Experiments* page allows you to work with normalized versions of the experiment.

1. Select a normalized version.
2. Select an action from the drop-down list:

    - **Data Analysis -** Allows you to perform filtering and statistical analysis.
    - **Gene Lists for Experiment -** Allows you to view gene lists associated with the selected version.
    - **Download** - Allows you to download an experiment version.
    - **Clone and Mutate -** Allows you to copy an experiment version.
    - **Delete -** Allows you to delete an experiment version.

3. Click **Go**. The page that pertains to your selection displays.

The *View All Experiments* page also provides links that allow you to:

- **Run Quality Control** on combined array experiments that have not previously had quality control checks run.
- **Normalize** a combined array experiment that has not previously been normalized or use different parameters to re-normalize.

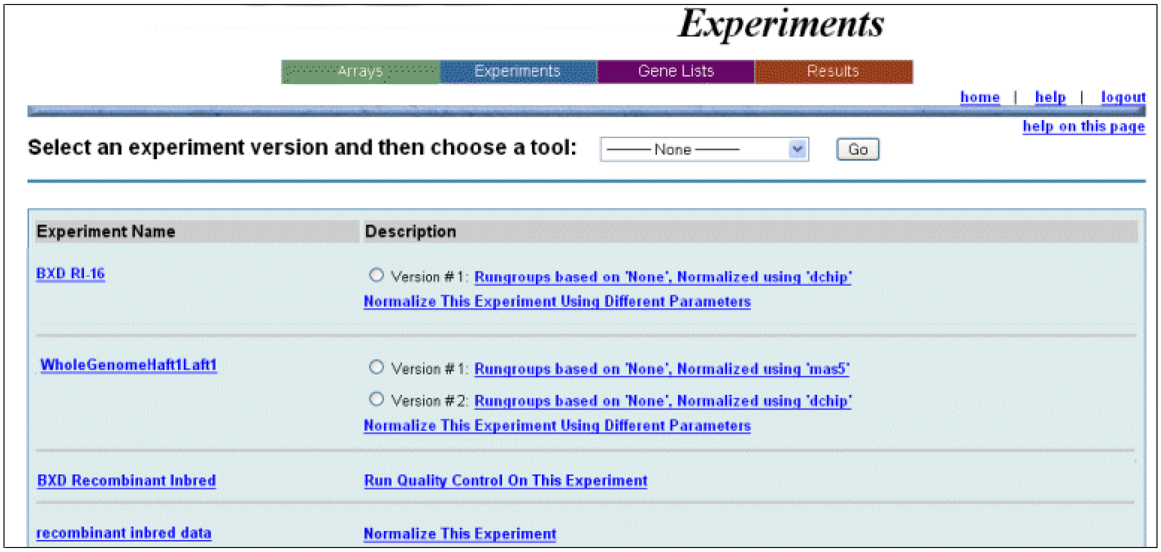

Viewing Experiment Details

To view details for an experiment:

- Click the **experiment name** on the *View All Experiments* page.
- Click the **experiment name** on any page where it displays.

The resulting *Experiment Details* page provides experiment details such as name, description, creator, date created, the number of arrays, organism, and probe type. It also allows you to view the normalizations and normalization parameters for the experiment, and each array in the experiment, if applicable.

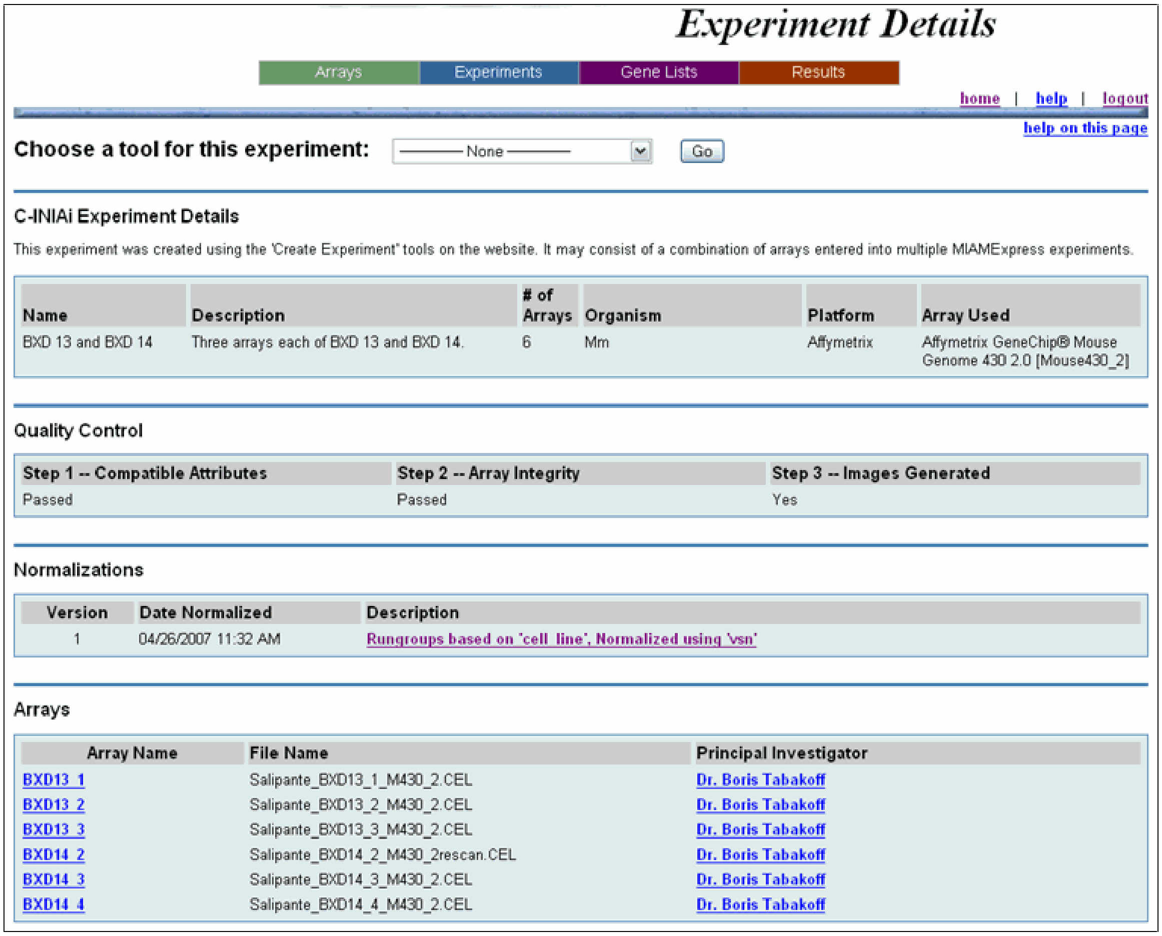

Creating a New Experiment

The PhenoGen website allows you to create experiments in two ways. You can combine arrays from the public data and arrays that you have permission to use, or you can upload a phenotype list and do correlation analysis.

Combine Arrays

The PhenoGen website provides infinite combinability for compatible arrays. You can select a full set or subset of arrays from a MIAMExpress experiment to add to an "in-silico" experiment. You can also select multiple arrays from different MIAMExpress experiments. You can only see the arrays that are public or that you have permission to access.

After you have selected all the arrays needed for your experiment, you must run a quality control (QC) check on the selected and merged arrays. See *Quality Control Checks* for details about the QC procedures that are used.

The *Create Experiment* page displays the arrays to which you have access. If you do not find an applicable array in those you have permission to use, you can upload your own data (see *Uploading Arrays*) or return to the *Browse All Arrays* page, select appropriate arrays, and request permission to access those arrays. See *Browse All Arrays (Logged In)*.

Creating an Experiment and Adding Arrays to an Experiment

Initial Steps

1. Select **Experiments menu > Create New Experiment**. The *Create Experiment* page displays.
2. Use the **Search** drop-down lists to narrow the array list. See *Searching Arrays* for details.
3. Click **Find Arrays**. The arrays that match your search criteria display.
4. Click the checkbox(es), in the **Select** column, beside the array(s) that you want as part of your experiment. Arrays can only be selected from the current page.

   Note: If you do not see all the arrays you want, return to the *Browse All Arrays* page to request access to more arrays.

   You cannot choose arrays from different organisms, different platforms (e.g., CodeLink or Affymetrix), or different types of arrays.

Creating a New Combined Arrays Experiment

After completing the *Initial Steps*:

1. Enter the **Experiment Name**.
2. Enter a **Description** of the experiment.
3. Click **Create Experiment**. The experiment is created.

Note: You can view the experiment on the *View All Experiments* page.

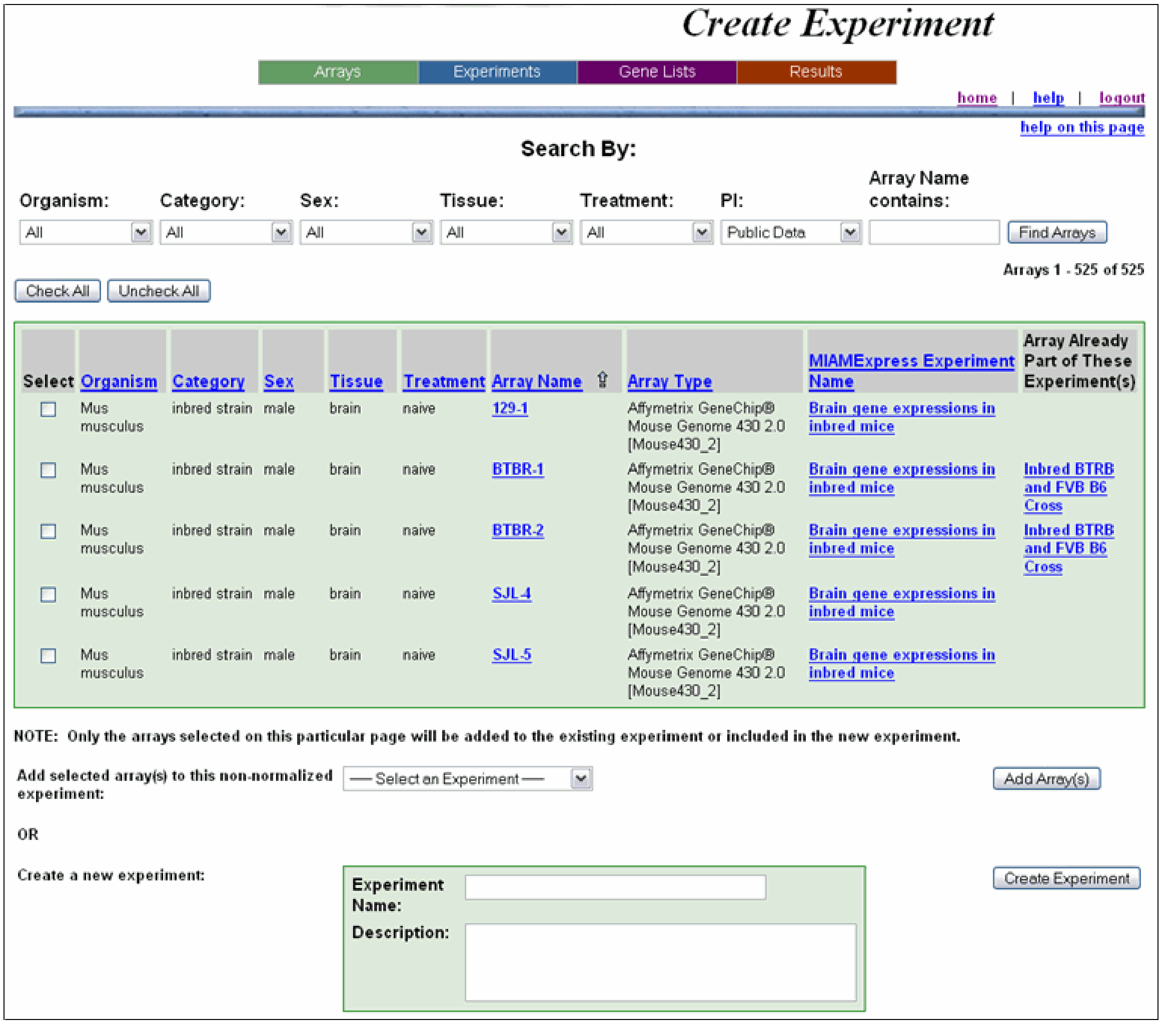

Adding Arrays to an Existing Non-Normalized Experiment

After completing the *Initial Steps*:

1. Select a non-normalized experiment from the **Add selected array(s) to this non-normalized experiment** drop-down list to add the arrays you selected to an existing non-normalized experiment.
2. Click **Add Array(s)** to add the selected arrays to the selected non-normalized experiment.

Notes:

- You can view the experiment on the *View All Experiments* page.
- You can click **Run Quality Control** to run the quality control check on your experiment.

Correlation Analysis

A correlation analysis is used to determine if two variables are associated with each other. The Correlation Analysis tool on the PhenoGen website searches for genes whose whole brain expression correlates with a phenotype using one of the data sets contained within the website (see below). The phenotype is uploaded by the user and can be any continuous measurement. For example, you may be interested in genes that are correlated with alcohol preference in mice. Alcohol preference would need to be measured in several of the inbred or recombinant inbred strains within the PhenoGen data sets. A positive correlation between alcohol preference and the expression value of a transcript would indicate that in strains where the expression for that transcript is high the mice also prefer more alcohol than mice whose expression for that transcript is low. A negative correlation would indicate an inverse relationship where a high transcript expression value is associated with a lower alcohol preference. The use of inbred and recombinant inbred strains allows for valid correlation analyses when the phenotype and the expression data are measured on different populations of mice in different laboratories.

Data Sets

BXD Recombinant Inbred Mice

The whole brain gene expression profile data for the BXD recombinant inbred mice includes 30 recombinant inbred strains and the two parental strains (C57BL/6J and DBA/2J). Each strain has 4 to 7 biological replicates for a total of 172 individual arrays. The whole brain mRNA for each naive 10-12 week old male mouse was hybridized to a separate array, i.e. no pooling of samples. The user has the option of using two different data sets. The different data sets represent the same raw data normalized two different ways.

Inbred Mice

The whole brain gene expression profile data for the inbred mouse strains includes 20 inbred strains. Each strain has 4 to 7 biological replicates for a total of 90 individual arrays. The whole brain mRNA for each naive 10-12 week old male mouse was hybridized to a separate array, i.e. no pooling of samples. The user has the option of using two different data sets. The different data sets represent the same raw data normalized two different ways.

BXD Recombinant Inbred and Inbred Mice

This expression data set represents a combination of the two data sets previously mentioned. In this data set there are a total of 50 strains (C57BL/6J and DBA/2J are in both of the previous sets) and 253 individual arrays. The user has the option of three normalization procedures for this data set.

HXB/BXH Recombinant Inbred Rats

The whole brain gene expression profile data for the HXB/BXH recombinant inbred rats includes data from 26 recombinant inbred strains, the two parental strains (SHR/Ola and BN-Lx/Cub), and the SHR-Lx/Cub strain. The whole brain mRNA of 4 to 7 naive 12-14 week old male rats from each strain were hybridized to separate CodeLink Whole Genome rat arrays (one rat per array) for a total of 139 arrays. The normalization procedure for this data set includes first adding 10 to each normalized expression value from the CodeLink software. This is done so in the next step the expression values can be transformed using a log base 2. The transformed values are then quantile normalized.

Creating a Correlation Analysis Experiment

Initial Steps

In order to perform correlation analysis, a phenotype data file needs to be created from a continuous measurement provided by the user. The phenotype data file should be a two column text file with no column headers. The first column should contain the strain name and the second column should contain the phenotype value for that strain. The strain name must exactly match the strain name in the provided data sets. The strain names for the data sets are shown when a data set is selected on the *Upload Phenotype Data* page.

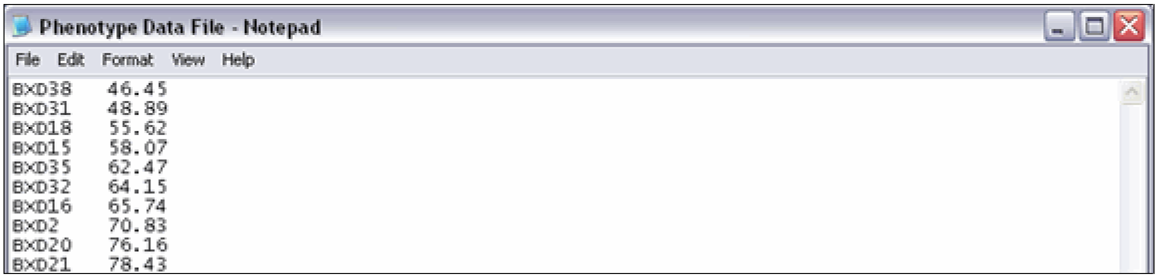
Phenotype Data File - Notepad
File Edit Format View Help
BXD38 46.45
BXD31 48.89
BXD18 55.62
BXD15 58.07
BXD35 62.47
BXD32 64.15
BXD16 65.74
BXD2 70.83
BXD20 76.16
BXD21 78.43

Uploading Phenotype Data

After completing the *Initial Steps*:

1. Select **Experiments menu > Create New Experiment > Do Correlation Analysis**. The *Upload Phenotype Data* page displays.
2. Enter the **Experiment Name**.
3. Enter a **Description** of the experiment.
4. Select a phenotype data file to upload by clicking on the **Browse** button.
5. Select a **Data Set** from the drop down menu. The strains included in this selection will display in the box to the right.
6. Click **Upload Phenotype Data**. A *Success* page will display with the number of matching strains uploaded.

Notes:

- The strain names in your phenotype data file must match the strain names in the selected data set exactly.
- Once the phenotype data is uploaded, the experiment is ready for Data Analysis.
- You can view the experiment on the *View All Experiments* page.

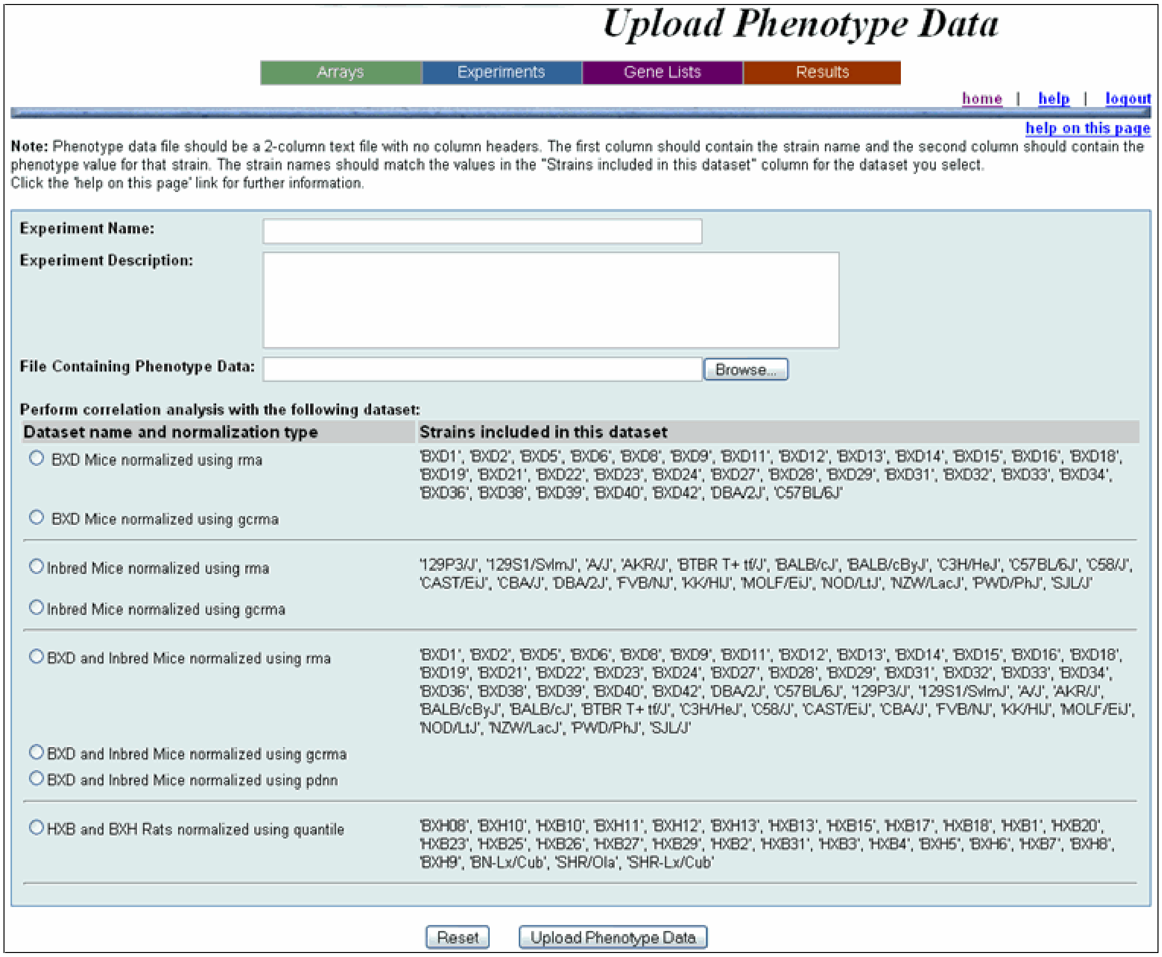
Upload Phenotype Data
Arrays
Experiments
Gene Lists
Results
home | help | logout
help on this page
Note: Phenotype data file should be a 2-column text file with no column headers. The first column should contain the strain name and the second column should contain the phenotype value for that strain. The strain names should match the values in the "Strains included in this dataset" column for the dataset you select.
Click the 'help on this page' link for further information.
Experiment Name:
Experiment Description:
File Containing Phenotype Data:
Browse...
Perform correlation analysis with the following dataset:
Dataset name and normalization type
Strains included in this dataset
BXD Mice normalized using rma
'BXD1', 'BXD2', 'BXD5', 'BXD6', 'BXD8', 'BXD9', 'BXD11', 'BXD12', 'BXD13', 'BXD14', 'BXD15', 'BXD16', 'BXD18', 'BXD19', 'BXD21', 'BXD22', 'BXD23', 'BXD24', 'BXD27', 'BXD28', 'BXD29', 'BXD31', 'BXD32', 'BXD33', 'BXD34', 'BXD36', 'BXD38', 'BXD39', 'BXD40', 'BXD42', 'DBA/2J', 'C57BL/6J'
BXD Mice normalized using gcrma
Inbred Mice normalized using rma
'129P3/J', '129S1/SvImJ', 'A/J', 'AKR/J', 'BTBR T+ tf/J', 'BALB/cJ', 'BALB/cByJ', 'C3H/HeJ', 'C57BL/6J', 'C58/J', 'CAST/EiJ', 'CBA/J', 'DBA/2J', 'FVB/NJ', 'KK/HIJ', 'MOLF/EiJ', 'NOD/LtJ', 'NZW/LacJ', 'PWD/PhJ', 'SJL/J'
Inbred Mice normalized using gcrma
BXD and Inbred Mice normalized using rma
'BXD1', 'BXD2', 'BXD5', 'BXD6', 'BXD8', 'BXD9', 'BXD11', 'BXD12', 'BXD13', 'BXD14', 'BXD15', 'BXD16', 'BXD18', 'BXD19', 'BXD21', 'BXD22', 'BXD23', 'BXD24', 'BXD27', 'BXD28', 'BXD29', 'BXD31', 'BXD32', 'BXD33', 'BXD34', 'BXD36', 'BXD38', 'BXD39', 'BXD40', 'BXD42', 'DBA/2J', 'C57BL/6J', '129P3/J', '129S1/SvImJ', 'A/J', 'AKR/J', 'BALB/cByJ', 'BALB/cJ', 'BTBR T+ tf/J', 'C3H/HeJ', 'C58/J', 'CAST/EiJ', 'CBA/J', 'FVB/NJ', 'KK/HIJ', 'MOLF/EiJ', 'NOD/LtJ', 'NZW/LacJ', 'PWD/PhJ', 'SJL/J'
BXD and Inbred Mice normalized using gcrma
BXD and Inbred Mice normalized using pdnn
HXB and BXH Rats normalized using quantile
'BXH08', 'BXH10', 'HXB10', 'BXH11', 'BXH12', 'BXH13', 'HXB13', 'HXB15', 'HXB17', 'HXB18', 'HXB1', 'HXB20', 'HXB23', 'HXB25', 'HXB26', 'HXB27', 'HXB29', 'HXB2', 'HXB31', 'HXB3', 'HXB4', 'BXH5', 'BXH6', 'HXB7', 'BXH8', 'BXH9', 'BN-Lx/Cub', 'SHR/Ola', 'SHR-Lx/Cub'
Reset
Upload Phenotype Data

Quality Control Checks Overview

Quality control is an essential process when combining arrays from multiple MIAMExpress experiments. This step is not necessary for correlation analysis experiments, since it has already been performed on the expression data sets. There are two quality control checks that ensure that the arrays you want to combine are compatible. They are:

- MIAMExpress data comparison
- Array integrity

When you run quality control, a quality control check of the arrays in the selected experiment is performed. Arrays that are identified as questionable at any or all of the steps should be considered for deletion. However, some of the small imperfections and minor concerns can be alleviated by an appropriate normalization method. See *Data Grouping and Normalization*.

For more details about the QC procedures for commonly used microarrays, see *Additional Quality Control Sources*.

MIAMExpress Data Comparison (Step 1)

The MIAMExpress information for the arrays is compared and discrepancies are listed for the user. A table displays that shows the warnings in yellow text. A warning can be generated by differences in the following attributes:

• Sex	• Individual Identifier
• Sample type	• Individual genetic trait or genotype
• Development Stage	• Disease state
• Age	• Separation technique
• Initial Time Point	• Cell type or target cell type
• Organism Part	• Cell line
• Genetic Modification	• Strain

Array Integrity (Step 2)

The quality control process looks specifically at each array. There are two steps involved in checking the array integrity:

1. Each array image is individually checked using measurements outlined by Affymetrix or CodeLink. See the topics *Guidelines for Assessing the Quality of Affymetrix Data* and *Guidelines for Assessing the Quality of CodeLink Data*.
2. Arrays within an experiment are compared. See the topic *Compare Arrays Within an Experiment*.

Note: Neither step indicates definitively whether an array is “bad”. Instead, you must balance considerations for quality of data and quantity of data with respect to the analysis at hand.

The output of this quality control check is a page that displays all the graphs for comparing arrays within an experiment.

Guidelines for Assessing the Quality of Affymetrix Data

Within-Array Quality Control Checks

The within-array quality control checks are examined using the Bioconductor package Simpleaffy. There are five checks that are examined; average background, hybridization controls, internal controls, percent present, and scaling factors.

Average Background Average background is examined to determine if it is consistent across arrays. Affymetrix has indicated that typical background averages range from 20 to 100, but there is no statistically relevant range for these values to fall within.

Hybridization Controls There are four hybridization controls that are added to each sample to monitor the hybridization efficiency. These four controls represent genes that should not be present in mRNA. They are added to the sample before hybridization in controlled, escalating quantities. BioB is added at the lowest concentration at 1.5 pM, right at the threshold of assay sensitivity. Affymetrix recommends that bioB be present at least 50% of the time. The other three genes, bioC, bioD, and cre, should be considered “present” all of the time and should exhibit increasing intensities.

Internal Controls There are two internal house-keeping genes (β-actin and GAPDH) that are used to evaluate the RNA and assay quality. Three probe sets have been designed per control. The first probe set measures the intensity of the 3’ end of the gene, the second probe set measures the intensity of the 5’ end of the gene, and the third probe set measures the intensity in the middle of the gene. The ratio of the intensity from the 3’ end to the 5’ end should theoretically be around 1. According to Wilson, et al. (2004), ratios above 1.25 for GAPDH should be considered outliers and ratios over 3 for β-actin should be considered outliers.

Percent Present Affymetrix recommends the use of a normalization and summary method call Microarray Suite 5.0 (MAS5). Within this normalization procedure, each probe set gives a Present, Marginal, or Absent call. The percent of present probe sets out of all probe sets on the array is also used as a quality control measure. Although the percent of present probe sets measure is highly dependent on each specific experiment with respect to the number of genes you expect to be expressed, an extremely low value raises suspicion about the quality of an array. Also, it is expected that duplicate arrays have similar percent missing levels.

Scaling Factors As part of the quality control procedure, intensities are normalized using the MAS5 procedure. Within the process of normalization, each array is adjusted by a scaling factor to get the trimmed mean of all arrays to equal a target signal. This scaling factor indicates how much RNA was hybridized onto the array. A wide variation of scaling factors across arrays can be a cause for concern. Affymetrix defines a wide variation as a three-fold or greater difference.

The Simpleaffy package from Bioconductor calculates the five within-array quality control check measures from a group of CEL files. Four out of the five are displayed on a single graph (next page).

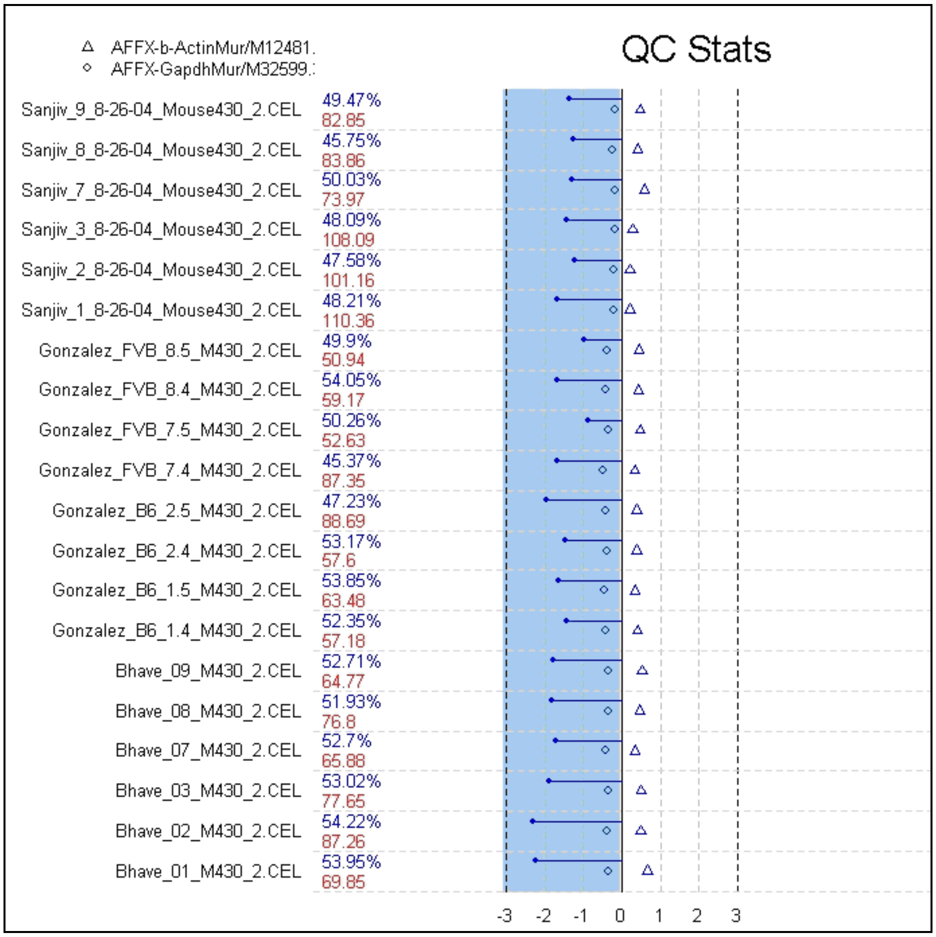

Along the left side of the graph are the names of the CEL files that were included in this analysis. The next column has two numbers per CEL file. The top number is the percent present and the bottom number is the average background for that CEL file. It is expected that the average background measures across arrays should be similar and, ideally, below 100. The percent present values should also be similar across arrays.

When the average background measures display in red, it indicates that the values across arrays show a “considerable amount of variation”. When the percent present values display in red, it indicates that there is a spread greater than 10% between the lowest and highest percentage.

The solid dots that are attached to a horizontal bar originating from the zero line represent the scale factors for each array. The blue shading that starts at approximately -3 and ends approximately at 0 is the region that spans three-fold below and three-fold above the average scale factor. In the example graph above, all of the scale factors fall into this range. However, if one scale factor did not fall within the range, the dot and horizontal line for that scale factor would display in red.

The open triangles represent the 3’ to 5’ ratio for β-actin. Affymetrix indicates that a value above 3 is cause for concern. However, the values that are plotted appear to be a difference in log signals as opposed to a ratio of signals. Therefore, in the graph above, a value of 0 for the ratio would be ideal and a value above 1.6 would be a cause for concern. None of the ratios in the graph are greater than 1.6.

Similarly, the open diamonds on the graph represent the log base 2 of the 3’ to 5’ ratio of GAPDH. This ratio should be below 0.32 (1.25 for the ratio). Again, none of the ratios are above the threshold.

The percent of samples with a present call for bioB and the log intensities should be examined for bioB, bioC, bioD, and cre to look for an increasing trend.

Compare Arrays Within an Experiment

Another package in Bioconductor looks at a model-based quality control assessment. There are three assessments that are examined at this stage; relative log expressions (RLE), normalized unscaled standard errors (NUSE), and array pseudo-images. To calculate all three assessments, a probe level model must first be fit to the data.

$$\log_2 PM_{kij} = \beta_{kj} + \alpha_{ki} + \varepsilon_{kij}$$

k = 1 …K probe sets

i = 1 … I_k probes

j = 1 … J arrays

In this default model, β_{kj} is the array effect, α_{ki} is the probe effect, and ε_{kij} is the residual error term. The model can be adjusted to include other effects, but the default model is used for quality control purposes.

Relative log expressions (RLE) The relative log expressions for each probe represent that particular probe's deviation from the median value of that probe across arrays. This quality assessment is dependent on the assumption that most of the genes measured are expressed at similar levels across the arrays. The relative logs are displayed as box plots. The expectation is that the relative log expressions should be evenly distributed around zero within each array, i.e., one array does not always have a higher intensity than all the other arrays. Also, if one or more arrays have box plots that are much larger than the other arrays, then these arrays tend to have more outliers than the other arrays.

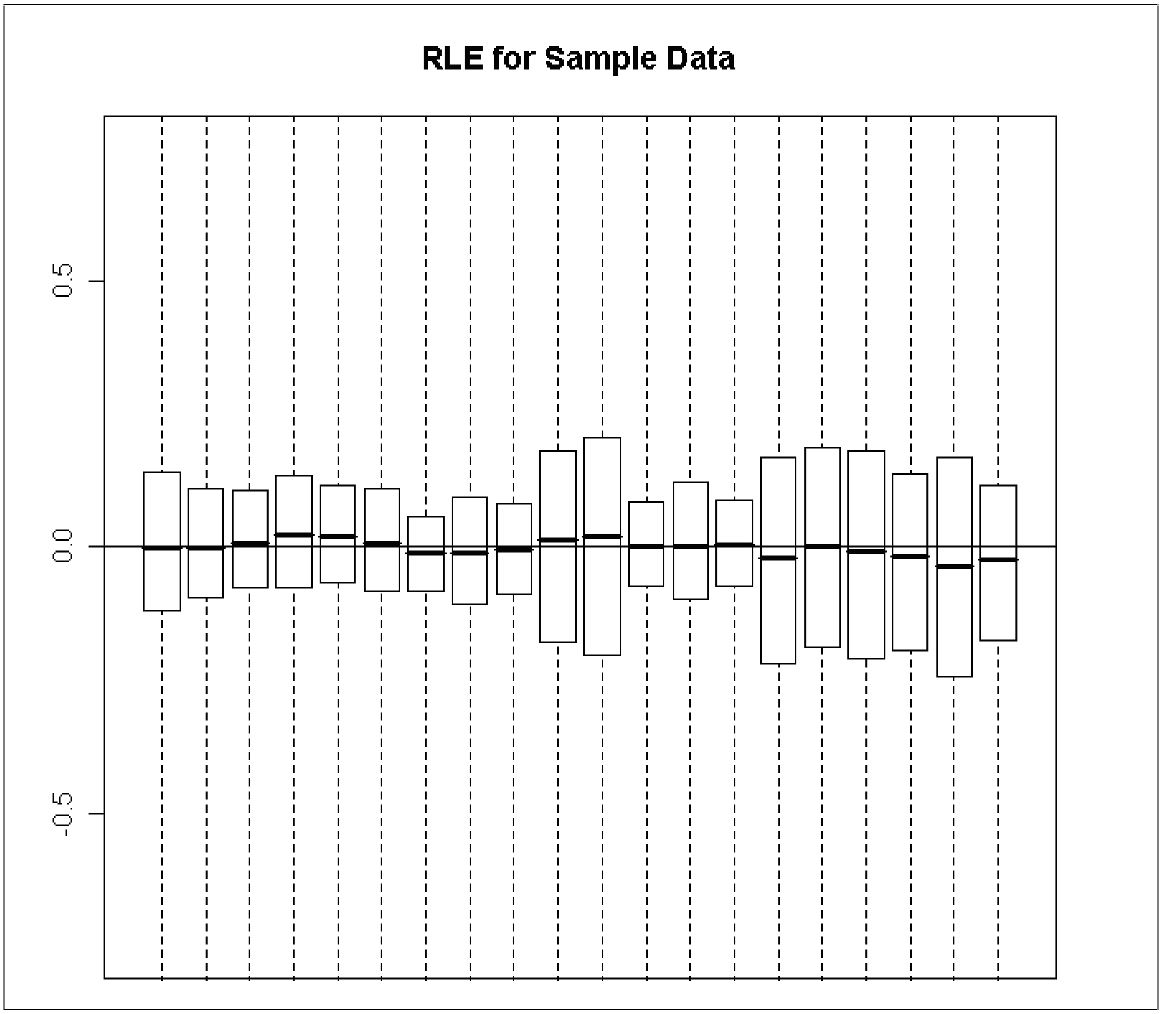

The RLE graph above displays a variety of box lengths and several boxes that are not centered around 0. This raises concerns about the distribution of the RLEs within arrays, but these minor issues could be resolved with normalization.

Normalized Unscaled Standard Errors (NUSE) The normalized unscaled standard errors represent the standard error between probe intensities within a probe set on a specific array. These errors are normalized by dividing all values of a particular probe set by the median standard error for that probe set across arrays. The expected distribution of NUSEs within an array is centered around zero, like the RLEs. A higher value indicates that the array has more variance for that probe set than the other arrays.

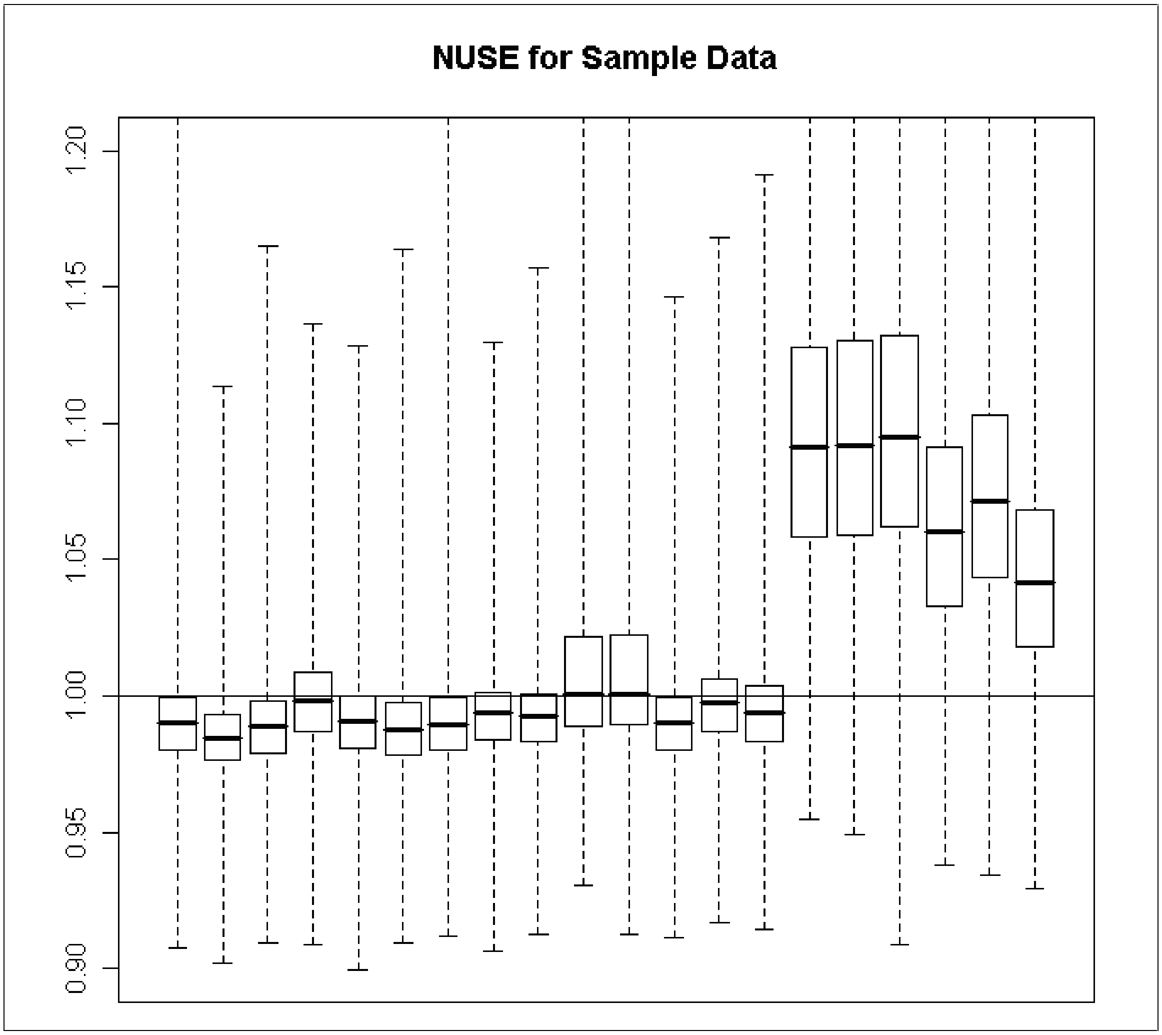

The plot above is a concern because the six samples on the right side have much more variation than the other 14 samples. The extent of the variation indicates that the samples could be of poorer quality.

Optional Images

There are two types of images that you may opt to create in the Quality Control Checks. These are pseudo-images and MA plots. There is a prompt when Quality Control is run, allowing you to generate these images, if desired. Images can be generated later by re-running the Quality Control.

Pseudo-images There are several different pseudo-images that you can inspect for artifacts that are not visible from the raw images. The default model is fit using the robust M-estimation method. In this method, weights are calculated for each probe. In some sense, weights can be considered a "standardized" residual. They range from 0 to 1 where 1 is a small residual and 0 is a large residual.

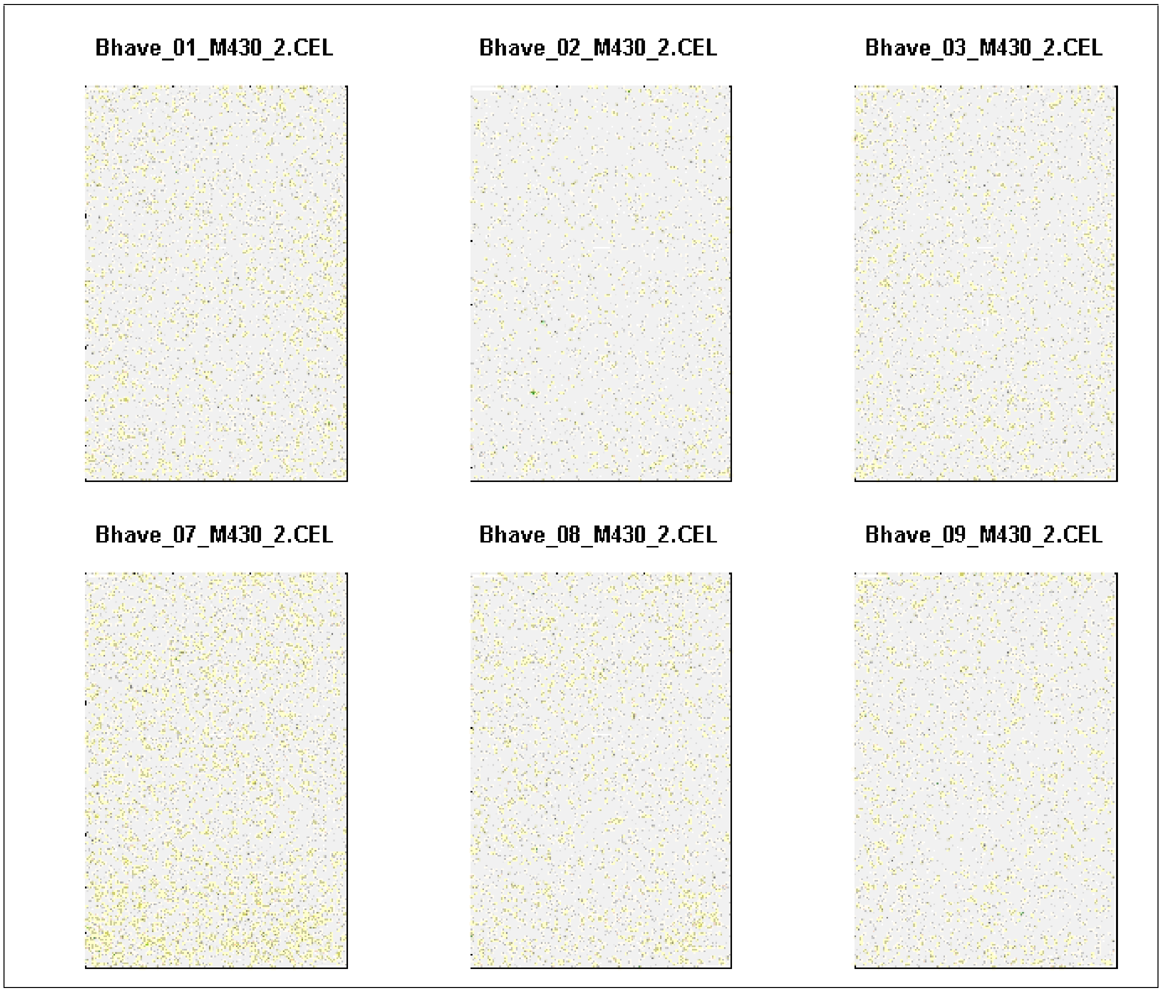

The pseudo-images (previous page) look good because the spots appear to be randomly scattered around the array, rather than being concentrated in any one area.

You can also look at the raw residuals using a pseudo-image, which represents the ε_{kij} value from the default model equation. As opposed to the weights, you want to see values in these images that are close to zero, which indicates that the model is a good fit. There are also several options for looking at the residuals; viewing both the positive and negative residuals on one array or separating them onto two different arrays. You can view a pseudo-image that represents just the sign of the residual, not the magnitude. Following is an example of each choice for the same array.

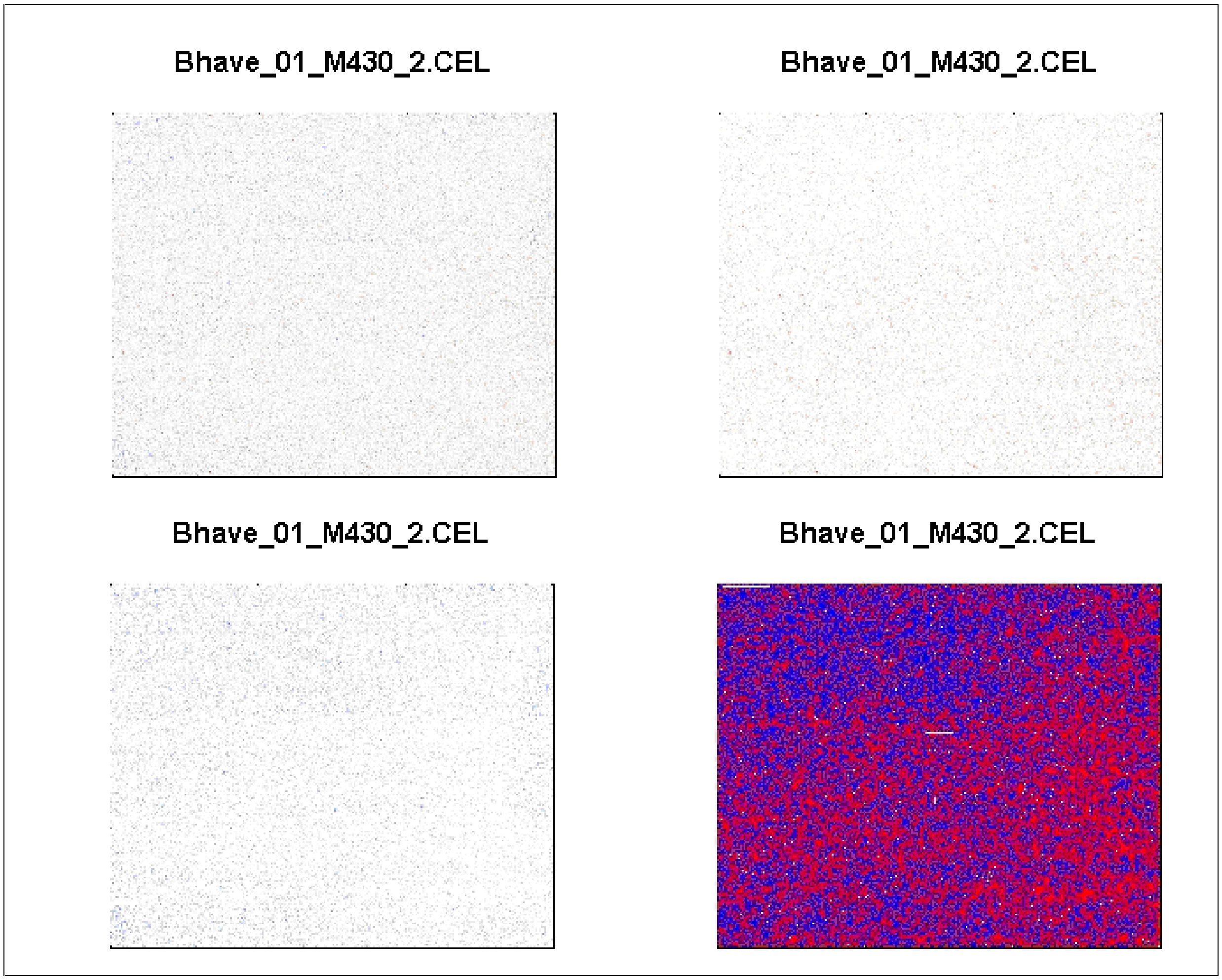

The image in the top left has both negative (blue) and positive (red) residuals shown. The intensity of each color represents the magnitude of the residual. The image in the top right shows only positive residuals. The image in the bottom left shows only negative residuals. Finally, the image in the bottom right represents the signs of the residuals only. In general, the plots show random distribution of color and intensity, indicating no major artifacts of concern for this array.

MA Plots An MA plot is a scatter plot used to compare two arrays. The y-axis is the log-fold change and the x-axis is the average log intensity between the two arrays. The example data uses 20 arrays, so instead of looking at each pair-wise comparison, a “reference” array is used. The reference array in the following graphs is the median intensities across all arrays. Again, the expectation is a random scatter plot, centered about the zero horizontal line. Below are MA plots for six of the arrays.

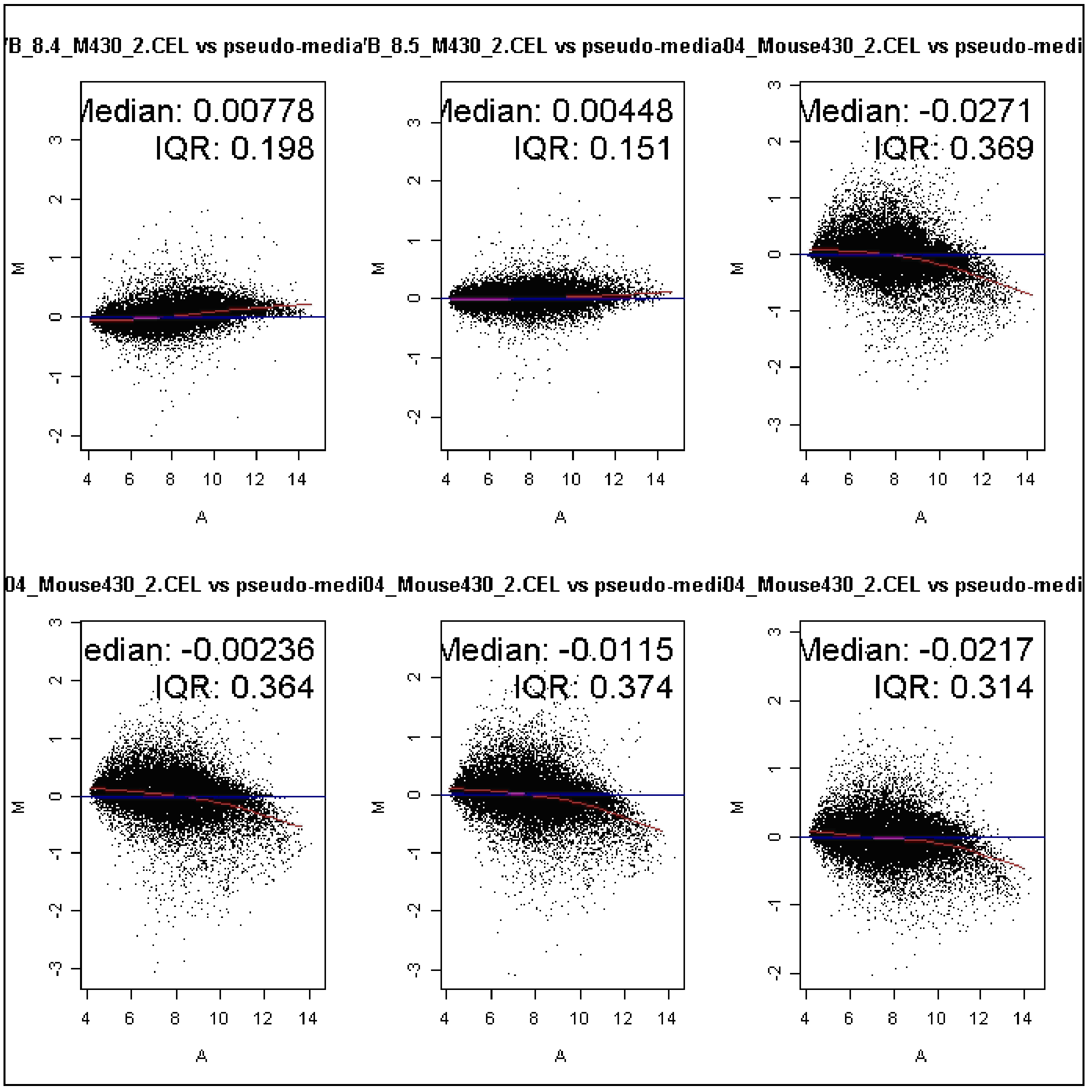

The blue line in the graph is the zero reference line. The red line is the loess curve, fit to the actual data. The top middle graph shows a “good” MA plot because the points are scattered evenly about the zero reference line and the loess line is close to the zero reference line. The normalized data for the three bottom MA plots shows some biases. Each of the loess curves has a downward slope at the higher average intensities.

The inter-quartile range (IQR) and median are also reported on the *Quality Control Results* page and on each graph. A compact IQR indicates that few genes are different, and there is less variation between arrays. A larger inter-quartile range can indicate that many genes are differentially expressed or that there is more variation between arrays. The IQR for the three bottom graphs is much larger than the IQR for the top center graph. The four graphs with the downward sloping loess curve (bottom three and top right) are arrays that were elevated in the NUSE graph. See the topic *Compare Arrays Within an Experiment* in the *Array Integrity* section.

Guidelines for Assessing the Quality of CodeLink Data

Quality control for CodeLink arrays is measured using Relative Log Expressions (RLE), Coefficient of Variation, and a table that displays flags set by the proprietary CodeLink software.

Relative log expressions (RLE) The relative log expressions for each probe represent that particular probe's deviation from the median value of that probe across arrays. This quality assessment is dependent on the assumption that most of the genes measured are expressed at similar levels across the arrays. The relative logs are displayed as box plots. The expectation is that the relative log expressions should be evenly distributed around zero within each array, i.e., one array does not always have a higher intensity than all the other arrays. Also, if one or more arrays have box plots that are much larger than the other arrays, then these arrays tend to have more outliers than the other arrays.

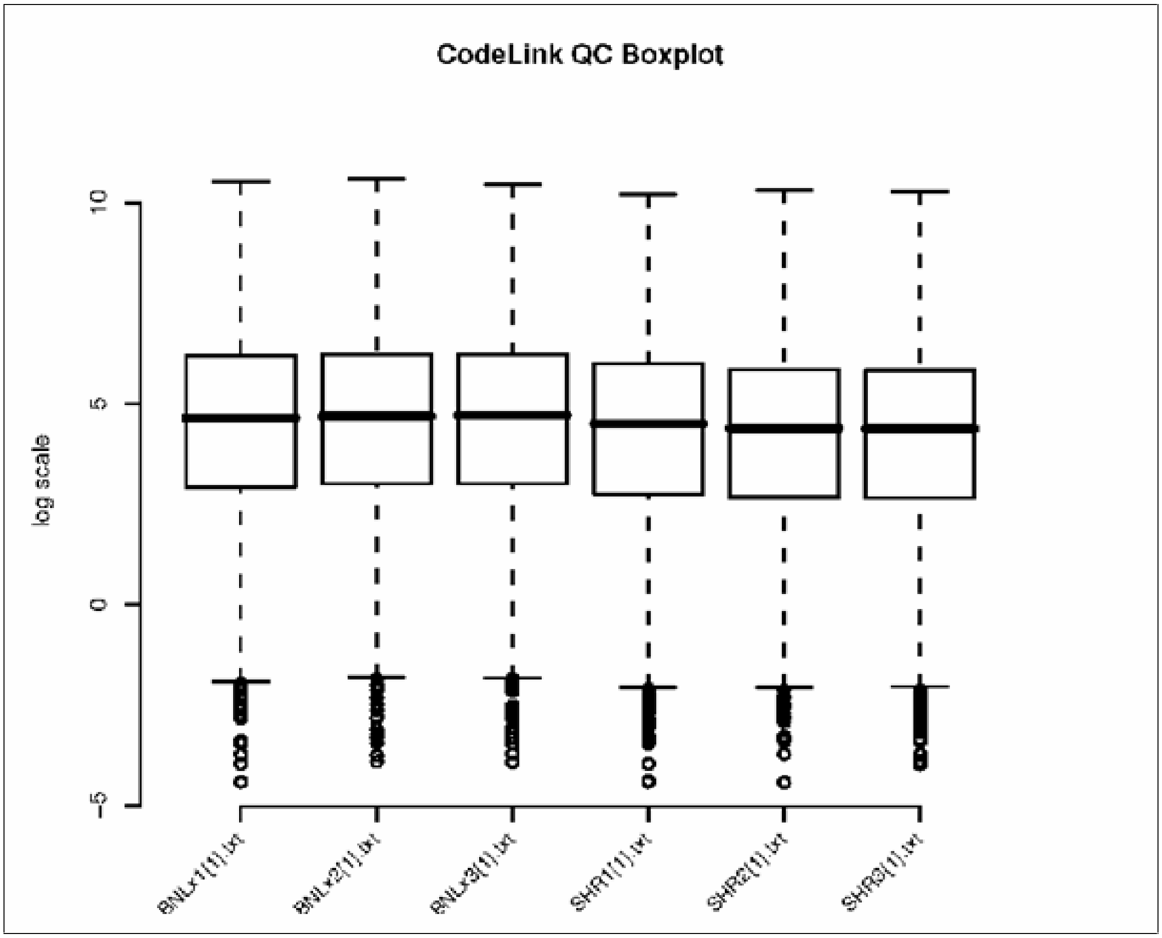

Coefficient of Variation (CV) Coefficient of Variation (CV) is a statistical measure of the dispersion of data points in a data series around the mean. It is calculated as follows:

CV = standard deviation / mean

The coefficient of variation represents the ratio of the standard deviation to the mean and it is a useful statistic for comparing the degree of variation from one data series to another, even if the means are drastically different from each other. The CV values are represented on the following graph where a vertical line is dropped down from each CV point to link to its array originality.

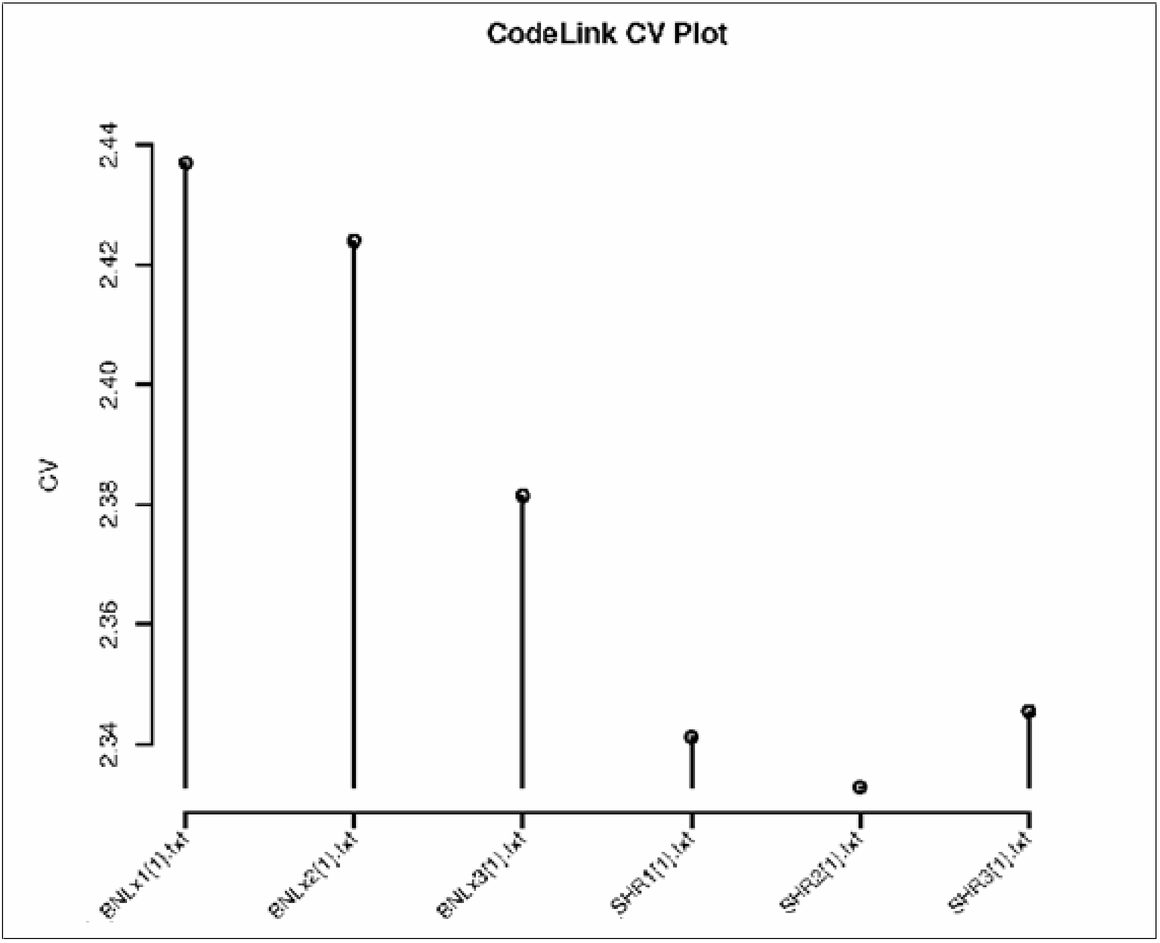

CodeLink Software CodeLink proprietary software produces quality control flags that show the integrity of each array. These flags serve as an indication of inferior arrays. The table lists some summary statistics for each array, as well as the number of spots labeled with perspective CodeLink Calls values. For example, arrays having high number of CodeLink Calls other than G (Good) can be considered for elimination. The **View** link in the last column shows a heatmap image of the background intensity for each array. A background value is useful for detecting artifacts, such as scratches and uneven concentration of sample distribution. See the topic *CodeLink Call Filter* in the *Filtering* section for details.

Note: Image generation is optional in the Quality Control process. If images are not created during Quality Control, the **View** link is not hyperlinked.

		Background Values			Number of Probes									
Delete	Array Name	Mean	Max	Min	G	L	C	CL	I	M	S	IS	CI	Image
☐	BNLx 1	53	75	45	24125	10119	4	2	68	25	2	0	0	[View Image]
☐	BNLx 2	52	148	44	24439	9679	69	28	85	42	2	0	1	[View Image]
☐	BNLx 3	53	444	45	24482	9745	4	2	84	25	3	0	0	[View Image]
☐	SHR 1	52	79	46	23500	10698	3	2	126	15	1	0	0	[View Image]
☐	SHR 2	52	871	45	23124	11067	2	4	126	20	1	0	1	[View Image]
☐	SHR 3	52	578	45	23110	11073	5	3	129	23	1	0	1	[View Image]

Running a Quality Control Check

After you create a combined array experiment, you must run a quality control check on the experiment. The *Quality Control* page can be accessed in two ways:

- Select **Experiments menu > Run Quality Control Checks**.
- Select **Experiments menu > View All Experiments** then click **Run Quality Control on this Experiment**. This option only displays next to experiments that have not yet had the quality control check run.

On the *Quality Control* page:

1. Select the experiment from the drop-down list for which you want to run the quality control check.
2. Click **Go**. The experiment name displays along with a prompt about generating images.
3. Select **Yes** or **No**.
4. Click **Run Quality Control Checks**.

The quality control checks take time. When they are completed, an email is sent to the address you provided in the *Registration* page to inform you that you can view your Quality Control Results. If the quality control process encounters errors, you must revise your array selection and rerun the quality control process. See *Viewing Quality Control Results* for instructions on deleting arrays.

Data Grouping and Normalization

Data grouping allows you to group the arrays and normalize data across groups. It is then in a state where statistical analysis can be performed. The *Data Grouping and Normalization* page displays all your combined array experiments. It also displays all saved groupings and normalizations with applicable arrays. To begin, you must first choose an experiment. You can then create run-groups, choose a normalization method, and name the version accordingly. See the topic *Creating a Run Group and Normalizing Data* for details.

Normalization Overview

The purpose of normalization and background correction is to remove systematic noise and reduce technical variation. To normalize in the context of DNA microarrays means to standardize your data to be able to differentiate between real (biological) variations in gene expression levels and variations due to the measurement process. Normalizing also scales your data so that you can compare relative gene expression levels. In general, the normalization process is subdivided into four sequential steps:

1. Background correction.
2. Data normalization.
3. Adjustment for non-specific binding (Affymetrix arrays only).
4. Data summary methods (Affymetrix arrays only).

The options for normalizing data are based on array type:

Affymetrix Arrays	CodeLink Arrays
• MAS5.0<br>• dChip<br>• RMA<br>• VSN<br>• GCRMA	• None<br>• Loess<br>• VSN<br>• LIMMA

Normalization Methods

MAS 5.0

Micro Array Suite Version 5.0 (MAS5) is implemented in both the MAS 5.0 software package from Affymetrix and in the Affy package in R. The PhenoGen website application uses the “mas5” function in R. Signal calculation using MAS 5.0 consists of five main steps:

The first step adjusts the raw intensities for a global background by organizing the array into zones. Within each zone, the lowest 2% of intensities are used as an estimate background for that zone. The transition between zones is smoothed by taking a weighted estimate of background for each point where the weights are based on distances from zone centers. The second step calculates an “ideal” mismatch (IM) intensity to adjust the perfect match intensity with the goal of eliminating background cross-hybridization and stray signal. The IM value is used instead of the mismatch (MM) value to ensure that the resulting signal (PM-IM) is positive. The third step transforms the intensity values with a log base 2

transformation. The fourth step combines probe values within a probe set using the one-step Tukey's biweight algorithm, which "weights" the data to reduce the influence of outliers. The fifth and final step scales all probe sets after conversion back to the original intensity scale, i.e., not log base 2 transformed, to a target probe set intensity (500 by default). After the MAS 5.0 procedure has been completed, all intensity values are transformed using a log base 2 in preparation for statistical analyses.

References

1. Affymetrix (2001). Statistical algorithms reference guide. Technical report Affymetrix.
2. Hubbell E, Liu W-M, Mei R (2002). Robust estimators of expression analysis. Bioinformatics 18(12):1585-1592.
3. Liu W-M, Mei R, Di X, Ryder TB, Hubbell E, Dee S, Webster TA, Harrington CA, Ho M-H, Baid J, Smeekens SP (2002). Analysis of high density expression microarrays with signed-rank call algorithms. Bioinformatics 18(12):1593-1599.

dChip (Perfect Match Probes Only)

DNA-chip Analyzer (dChip) is actually a software package that implements the model proposed by Cheng Li and Wing Hung Wong. This model is also referred to as the Li Wong method and the resulting values are considered model-based expression indexes (MBEI). The PM-only model fits the following model to each gene $PM_{ij} = v_j + \theta_i \phi'_j + \varepsilon_{ij}$, where PM_{ij} is the intensity for perfect match probe of probe pair *j* on the *i*th array, v_j is the baseline response of the *j*th probe due to nonspecific binding, θ_i is the expression index of the gene in the ith array, ϕ'_j is the sensitivity of the PM probe of the probe pair *j*, and ε_{ij} is the random error. Parameter estimates are determined through iteration. The R code used for normalization on the PhenoGen website is not identical to the code used in Li and Wong's stand alone program *dChip*. Therefore, normalization on the website might differ slightly from normalization derived from the *dChip* program. After the dChip method is completed, all intensities are transformed using a log bas 2 in preparation for statistical analyses.

References

1. Li C, Wong WH (2001). Model-based analysis of oligonucleotide arrays: expression index computation and outlier detection. Proc Natl Acad Sci U S A 98(1):31-6. (uses both PM and MM probes)
2. Li C, Wong WH (2001). Model-based analysis of oligonucleotide arrays: model validation, design issues, and standard error application. Genome Biology 2(8):1-11. (uses only PM probes)

RMA

Robust Multi-Chip Average/Robust Multi-Array Analysis (RMA) RMA preprocessing consists of three main steps: background correction, normalization, and summarization of probe level intensities in probe sets. RMA uses a background correction method to account for optical noise and non-specific binding using the perfect match probes only. The background-corrected probe intensities are then log base 2 transformed and normalized using quantile normalization. Finally, the probe intensities are combined using a median polish to get one intensity for each probe set. The log base 2 transformation of intensity values occurs within the RMA procedure, so a separate transformation is not needed.

References

1. Bolstad BM, Irizarry R A, Astrand M, Speed TP (2003). A Comparison of Normalization Methods for High Density Oligonucleotide Array Data Based on Bias and Variance. Bioinformatics 19(2):185-193.
2. Irizarry RA, Benjamin M, Bolstad FC, Cope LM, Hobbs B, Speed TP (2003). Summaries of Affymetrix GeneChip probe level data. Nucleic Acids Research 31(4):e15.
3. Irizarry RA, Hobbs B, Collin F, Beazer-Barclay YD, Antonellis KJ, Scherf U, Speed TP (2003). Exploration, Normalization, and Summaries of High Density Oligonucleotide Array Probe Level Data. Biostatistics 4(2): 249-264.

VSN

Variance stabilization (VSN) The goal of variance stabilization is to transform the data in such a way as to eliminate the dependence of the variance on the mean. The VSN accounts for both background correction and normalization by scaling and shifting the original intensity and then using the inverse of the hyperbolic sine as a variance stabilizing transformation. For Affymetrix data, this normalization method is implemented in the Affy expresso() procedure. The VSN transformation is done at the probe level on the PM probes only and then the probe level intensities are combined into a probe set intensity using a median polish. For CodeLink data, this normalization method is implemented in the VSN procedure in R using the VSN option on the website and in the *Normalize Between Arrays* procedure using the LIMMA option on the website. Affymetrix data is transformed to log base 2 within the VSN procedure. Likewise, when the LIMMA option is used for VSN of CodeLink data, values are on the log base 2 scale. When the VSN option is chosen without LIMMA, the values are on the natural log scale. For CodeLink, data values are background corrected before VSN is implemented. When using the VSN option for normalization of CodeLink data, you are given the option of which quantile you want to use in the least-trimmed sum of squared (LTS) regression for the estimation of parameters. Possible values range from 0.5 to 1, where 1 offers no protection to outliers and 0.5 is the most robust procedure. In the two other implementations of VSN (Affymetrix and CodeLink via LIMMA) a quantile of 0.5 is used.

References

1. Huber W, von Heydebreck A, Sultmann H, Poustka A, Vingron M (2002). Variance stabilization applied to microarray data calibration and to the quantification of differential expression. Bioinformatics 18(S1):S96-S104.
2. Huber W, von Heydebreck A, Sultmann H, Poustka A, Vingron M (2003). Parameter estimation for the calibration and variance stabilization of microarray data. Statistical Applications in Genetics and Molecular Biology 2(1) Article 3. http://www.bepress.com/sagmb/vol2/iss1/art3

GCRMA

G/C Robust Multi-Array Average (GCRMA). The GCRMA method is related to the RMA method described previously, in that GCRMA also uses quantile normalization on the log base 2 prove values and a median polish to summarize probes into a probe set. However, the difference lies in the background adjustment. The original RMA method does not account for probe affinity when calculating background. For GCRMA, the estimate for non-specific binding is related to the base composition of the nucleic acid molecules, i.e., the proportion of G and C bases present in the probe sequence.

References

1. Wu Z, Irizarry RA, Gentleman R, Martinez Murllio F, Spencer F (2004). A model based background adjustment for oligonucleotide expression arrays. Johns Hopkins University, Dept. of Biostatistics Working Papers, Paper 1. http://www.bepress.com/jhubiostat/paper1

LOESS

Locally Weighted Scatterplot Smoothing (LOWESS/LOESS). For application to normalization of CodeLink arrays, this method is also referred to as cyclic loess. This method is an iterative approach that is based on the MA plot. For each distinct pair of arrays, the data is plotted using an MA plot (difference in log base 2 values versus the average of log base 2 values). A loess curve using a one-degree polynomial is fit to each graph. This loess curve is used to estimate an adjustment for each value. The average adjustment for all pairwise comparisons for a particular probe is used to obtain starting values for that probe and array in the next iteration. This method is implemented using the **normalize.loess** function in R. Initially, expression values from CodeLink are adjusted for background, i.e., spot mean–background median. Because this can result in negative values, expression values less than 1 are re-coded to 1. Expression values are transformed using a log base 2 before LOESS normalization. You have the option to fit the loess curve using a weighted least –squares approach (family.loess=gaussian) or a re-descending M estimator with the Tukey's biweight function (family.loess=”symmetric”).

References

1. Bolstad BM, Irizarry R A, Astrand M, Speed TP (2003). A Comparison of Normalization Methods for High Density Oligonucleotide Array Data Based on Bias and Variance. Bioinformatics 19(2):185-193.

LIMMA

Linear Models for MicroArrays (LIMMA) LIMMA refers to the package that is used in R. Four normalization methods are available under this method; **none**, **scale**, **quantile**, and **vsn**.

- *None* - Data is background corrected, values less than 1 are re-coded to 1, and all values are log base 2 transformed, but no normalization is done.
- *Scale* - Data is background corrected, values less than 1 are re-coded to 1, and all values are log base 2 transformed. Values are then scaled so that the median absolute deviations (MADs) are the same across arrays.
- *Quantile* - Data is background corrected, values less than 1 are re-coded to 1, and all values are log base 2 transformed. Values are then adjusted so that each array has the exact same distribution of intensities. This gives the same results as the **normalize.quantiles** function in R.
- *VSN* - Employs the same methods as the other VSN option. However, the output for the other method is on the natural log scale and the output from this function for VSN is on the log base 2 scale. Data is background corrected before the VSN procedure is applied.

References

1. Bolstad BM, Irizarry R A, Astrand M, Speed TP (2003). A Comparison of Normalization Methods for High Density Oligonucleotide Array Data Based on Bias and Variance. Bioinformatics 19(2):185-193.
2. Smyth GK and Speed TP (2003). Normalization of cDNA microarray data. Methods 31(4): 265-273,

Run Groups Overview

The term run group is used to indicate an analysis group. For example, if you are analyzing data for differential expression between males and females, run group 1 could be all the samples that are from female mice and run group 2 could be all the samples that are from male mice. For inbred strains, replicate samples within a group can be considered biological replicates, because even though the samples are two different animals, it is assumed that the gene expression is similar (if not exactly the same) between the two samples.

Run-groups can be chosen based on:

- MIAMExpress attribute.
- Previous user run-group and normalization combinations that are saved.
- Category created by the user.

Creating Run Groups and Normalizing Data

The *Grouping & Normalization* page can be accessed in two ways:

- Select **Experiments menu > Data Grouping and Normalization**.
- Select **Experiments menu > View All Experiments**, then click one of the following links beside the experiment you want to normalize:
  - **Normalize this Experiment**
  - **Normalize this Experiment Using Different Parameters**

In the *Normalization* page:

1. Select an experiment from the drop-down list.
2. Click **Go**. The experiment displays.
3. Select the **Run Groups** by:

   - Choosing from the drop-down list and clicking **Select Criterion** to apply the choice you selected. The samples are sorted into run groups automatically. You have the option to exclude any sample(s) in the list.
   - Manually adding samples to the **Run Groups** or **Exclude** columns.
   - Creating new run groups by clicking **Create Rungroup** and naming them.

4. Select a **Normalization Method** from the drop-down list. See the topic *Normalization Methods* in the *Data Grouping and Normalization Overview* section.

   #### Normalizing Affymetrix Arrays

   If you have an experiment with Affymetrix arrays, choose:

   - **MAS5** (method implemented in Affymetrix GeneChip 5 software) **-** Proceed to step 5.
   - **dChip** (DNA chip analyzer) **-** Proceed to step 5.
   - **rma** (robust multi-array average method) **-** Proceed to step 5.
   - **vsn** (Rocke's variance stabilization normalization method) **-** Proceed to step 5.
   - **gcrma** (G/C robust multi-array average) **-** Proceed to step 5.

   #### Normalizing CodeLink Arrays

   If you have an experiment with CodeLink arrays, choose:

   - **None -** Proceed to step 5.
   - **Loess** (Locally Weighted Scatterplot Smoothing) **-** Select **gaussian** or **symmetric** from the drop-down list that displays, then proceed to step 5.
   - **vsn** (Rocke's variance stabilization normalization method) **-** Select **Lts.quantile** and **Number of iterations** from the drop-down lists that display, then proceed to step 5.
   - **LIMMA** (Linear Models for MicroArrays) **-** Select the **Limma method** from the drop-down list that displays, then proceed to step 5.

5. Change the default **Normalization version name** for the data grouping and normalization, if appropriate.
6. Click **Normalize Data**. If the normalization is successful, the *Success* page displays.

Note: You can group and normalize the same experiment multiple times.

After the normalization process is complete, you can open the *View All Experiments* page to see the experiment with the version listed below. See the topic *Viewing an Experiment*.

Data Analysis

Data analysis consists of:

1. Filtering out noise in the data by applying optional filters which allow the user to eliminate certain probe sets from further analysis.
2. Identifying statistically significant genes using statistical analysis.
3. Saving the resultant data.

Data analysis tools can be accessed from:

- **Gene List menu > Create Gene List > From Experiment**
- **Experiments menu > Data Analysis**
- ***Experiment Details* page**

Filtering

A typical microarray consists of thousands of probe sets (10,000 – 45,000). As a result, the introduction of meaningless noise is inevitable. Removing this noise increases the chances of finding significant genes. The PhenoGen website provides various gene filtering methods for:

- Affymetrix
- CodeLink

The website also provides the ability to filter a saved experiment multiple times after normalization.

Note: The PhenoGen website does not provide custom array filtering.

Affymetrix Gene Filtering Procedures

Control Gene Filter

Affymetrix technology uses house-keeping genes' intensity values as a means for quality control. A pre-determined concentration of these control genes were spiked into the cRNA target mixture prior to application onto the microarrays. The measured intensity values are used for internal quality control algorithm. Since these control genes are typically from different species, they have little importance to the analysis. Therefore, Control Gene Filter is used to remove these control genes.

MAS5 Absolute Call Filter

The MAS5 algorithm uses probe pair intensities to generate a Detection p-value and assign a Present, Marginal, or Absent call. Each probe pair in a probe set is considered as having a potential vote in determining whether the measured transcript is detected (Present) or not detected (Absent). The vote is described by a value called the Discrimination score [R]. The score is calculated for each probe pair and is compared to a predefined threshold Tau. Probe pairs with scores higher than Tau vote for the presence of the transcript. Probe pairs with scores lower than Tau vote for the absence of the transcript. The voting result is summarized as a p-value. The greater the number of discrimination scores calculated for a given

probe set that are above Tau, the smaller the p-value and the more likely the given transcript is truly Present in the sample. The p-value associated with this test reflects the confidence of the Detection call.

The Detection p-value cut-offs, Alpha 1 ($\alpha 1$) and Alpha 2 (α 2), provide boundaries for defining Present, Marginal, or Absent calls. At the default settings determined for probe sets with 16–20 probe pairs (defaults α 1 = 0.04 and α 2 = 0.06), any p-value that falls below α 1 is assigned a Present call, and above α 2 is assigned an Absent call. Marginal calls are given to probe sets which have p-values between α 1 and α 2. The p-value cut-offs can be adjusted to increase or decrease sensitivity and specificity.

Negative Control Filter

The *Affymetrix Negative Control Probe Filter* takes advantage of several control probe sets on the Affymetrix array. There are four transcripts represented by three probe sets that are labeled Lys, Phe, Thr, or Dap. In each set of three probe sets there is a probe set with probes at the 3' end, the 5' end, and the middle of the gene. According to Affymetrix standards, these three transcripts should be added to the mRNA mixture as a quality control measure before it is hybridized to the array. However, to save costs, most arrays are run without this spike. All arrays run at the Colorado facility do not have these three transcripts present. Therefore, these three transcripts are used as negative controls. An array's detection limit is equal to the mean of the 3 probe sets representing the transcript out of the four negative controls that has the higher intensity plus two standard deviations.

Note: User must know if the Affymetrix control probes were spiked into the mRNA mixture or not before using this filter.

CodeLink Gene Filtering Procedures

Control Gene Filter

There are three basic types of CodeLink probes that are used in the Control Gene Filter:

- **(D) Discovery**: The probes corresponding to the genes of interest for a particular species.
- **(P) Positive controls**: The bacterial probes corresponding to bacterial transcripts that are spiked in at the total RNA level and are used to evaluate the sensitivity and dynamic range of the platform.
- **(N) Negative controls**: The bacterial probes used for evaluating the degree of nonspecific assay background and negative control threshold.

Upon completing this filter, both P and N are removed.

CodeLink Call Filter

The CodeLink Call filter is based on the quality control flag results measured from the imaging software; *CodeLink Expression v4.1 algorithm*. The flags are:

Flag	Description
G	The spot has passed all quality control measures and is defined as good.
M	The spot is identified in the MSR (Manufacturing Spot Removed) File and no intensity data is provided. The probe was masked after printing because it represented a suboptimal probe. Data from these spots is disregarded.
C	The spot has a high level of background contamination. Its background is above the global background population.
I	The spot has an irregular shape.
L	The spot has a near background signal.
S	The spot has a high number of saturated pixels, typically above 60,000 units.

CodeLink uses the quality control flags listed above to compute the two types of Calls:

- **(P) Present Call -** The spot receives a P call if it gets a G flag.
- **(A) Absent Call -** The spot receives an A call if it gets one or more of the following flags: M, C, I, L and S.

Median Filter

The first filter identifies genes with a moderate variance over the entire experiment; genes whose expression does not vary at all seem unlikely to be involved in these phenotypes. Since it seems likely that most genes are not involved, the gene with the median variance is a reasonable model of null variation; i.e., the variation due to other factors. The variance is calculated as s2 for each gene. The null hypothesis is that these variances represent random and normally distributed noise. The system computes the statistic W = (N-1)s2/median(s2), where N is the number of observations of the gene, which is approximately chi-square distributed with N-1 degree of freedom [(Hogg & Craig, 1978), p175]. The system calculates a p-value for rejecting the null hypothesis and performs the False Discovery Rate (FDR) multiple testing correction (Benjamini & Hochberg 1995), setting the FDR to 10%. The result is a list of genes with significantly greater variation than the median variation gene with, at most, 10% of that list including genes that have true variation less than, or equal to, median variation.

Coefficient Variation Filter

The Coefficient Variation filter measures the consistency of the probes across all samples. The coefficient of variation (CV) of each probe is calculated as standard deviation divided by mean. A high CV value reflects inconsistency among the samples within the group. For a two-group comparison study, the CV of each group is calculated independently. You can predefine a cutoff value, where genes with CVs above the cutoff value are removed. You can also define different cutoff thresholds to control the consistency levels of the genes.

Negative Control Probe Filter

The negative control probe filter takes advantage of the negative controls on the CodeLink arrays to create thresholds for detection. Detection limits are calculated for each array individually based on the negative controls on that array only. The detection limit is equal to the mean plus two standard deviations of the set of negative probes with the highest mean. Both the mean and standard deviation are calculated based on a 5% trimmed data set.

Statistical Analysis Overview

The statistical procedure for microarray analysis comprises:

- Statistics for differential expression in two run groups
- Statistics for differential expression using one-way ANOVA
- Statistics for differential expression using two-way ANOVA
- Statistics for differential expression with replicate lines of selectively bred animals
- Gene expression and phenotype correlation analysis
- Multiple comparison correction testing

The type of statistical tools available to the user is dependent on the type of experiment and the number of run groups specified during the Data Grouping and Normalization process. If more complicated analyses are needed, you can download the raw CEL files or the normalized data for analysis in the statistical package of your choice.

Statistics for Differential Expression for Two Run Groups

If two run groups are identified, the user has the choice of two types of statistical analyses:

- Parametric
- Non-parametric

Parametric Analysis

The parametric analysis is a two-sample t-test assuming equal variances executed using the R function “t.test”. This test is considered parametric because it assumes that the distribution of expression values is normal within each group. However, expression data is often not distributed normally and there are often not enough observations to assume that the central limit theorem applies.

Non-Parametric Analysis

A non-parametric test does not depend on the distribution of the expression data. The Wilcoxon rank sum test is invoked when you specify a non-parametric test. This analysis is done in R using the function “wilcox_test" from the coin package. When the sample size is under 50 the exact p-value is used, otherwise a normal approximation is calculated. The parametric t-test is more powerful when the data are truly normally distributed, but the non-parametric test is robust for data that is not normally distributed.

Statistics for Differential Expression Using One-Way ANOVA

The PhenoGen website allows the user to do statistical analyses with more than two run groups using a one-way ANOVA model, which compares the within group variance and the between group variance. When there are three or four groups, the user can choose his/her hypothesis of interest from any of the possible pair-wise contrast (e.g. Group1 vs. Group 2) or the overall effect of group (factor effect) on the model. When there are more than four groups, the user can only use the factor effect as the hypothesis of interest. For the pair-wise contrasts, a moderated t-statistic is used to test significance, and for factor effects, a moderated F-statistic is used. For the moderated t-statistic and F-statistic, the standard deviation of the ordinary test is “shrunk” to reflect information that is borrowed across genes (Smyth 2004).

Reference

Smyth GK (2004). Linear Models and Empirical Bayes Methods for Assessing Differential Expression in Microarray Experiments. Statistical Applications in Genetics and Molecular Biology 3(1), Article 3.

Statistics for Differential Expression Using Two-Way ANOVA

A user can choose to input factors or use factors from the MIAMExpress files in a two-way analysis of variance. The user can choose from four different effects to test for; main effect of factor 1, main effect of factor 2, the interaction effect between factor 1 and factor 2, or the overall model effect. To test for the interaction effect or the overall model effect a regression model is used that contains the main effects for both factor 1 and factor 2 plus the interaction effect, a true two-way ANOVA. To test for main effects, however, the interaction effect is not included in the model. F-statistics are reported in all cases. Factors that are uploaded as character variables are treated as categorical and factors that are uploaded as numerical variables are treated as continuous.

Statistics for Differential Expression with Replicate Lines of Selectively Bred Animals

A t-test with noise distribution for replicate lines can be implemented when the user would like to determine a list of genes that are differentially expressed between two selected lines and the user has a replicate experiment, i.e. “high” selected line from replicate 1, “low” selected line from replicate 1, “high” selected line from replicate 2, and “low” selected line from replicate 2. This method was first introduced by Eaves et al in 2002.

This method involves first calculating a modified t-statistic for each probe/probe set for each replicate separately where the traditional sample variances are replaced with a “pooled” variance. The pooled variance is calculated for each group using a weighted mean between the observed variance and a mean local variance. The weights are 2 to 1 where the larger of the two variances is given the larger weight. To calculate the mean local variance, the data first is sorted by mean expression for each probe/probe set, the mean local variance is then calculated as the mean of variances the 250 probes/probe sets immediately below the probe/probe set of interest and the 250 probes/probe sets immediately above the probe/probe set of interest.

Once t-statistics are calculated for each probe/probe set and each replicate, the probes/probe sets are separated into two groups. Probes/probe sets are placed in the null distribution if their t-statistics show opposite signs in the two replicate experiments. The t-statistics for these experiments are used to generate a null distribution of t-statistics for p-values to be based on. Instead of individual p-values being calculated for each probe/probe set, an initial p-value threshold is set and it is determined whether or not probes/probe sets meet this criteria. Using this method, the type I error rate (p-value) is determined by product of the probability of a t-statistic greater than the one observed given the gene is not differential expressed in replicate experiment 1(i.e. in null distribution), the probability of a t-statistic greater than the

one observed given the gene is not differential expressed in replicate experiment 2(i.e. in null distribution), and the probability of having the two t-statistics showing the same direction of differential expression (i.e. 0.5). Therefore, the percentiles from the null distribution used to determine 'significance' can be calculated for a specific error rate by taking the square root of the fraction, the probability of having the two t-statistics showing the same direction of differential expression divided by the specified error rate. Therefore, a gene is considered significant if the observed t-statistic for each replicate is larger than the threshold t-statistic determined from the null distribution for that replicate. Exact p-values are not reported for this statistical method, therefore, multiple testing corrections cannot be implemented.

Reference

Eaves IA, Wicker LS, Ghandour G, Lyons PA, Peterson LB, Todd JA, Glynne RJ (2002). Combining mouse congenic strain and microarray gene expression analyses to study a complex trait: The NOD model of type1 diabetes. Genome Research 12:232-243.

Gene Expression and Phenotype Correlation Analysis

A correlation analysis is used to determine if two variables are associated with each other. The Correlation Analysis tool on the PhenoGen website searches for genes whose expression is associated with a phenotype using one of the inbred or recombinant inbred data sets contained within the website. The phenotype is uploaded by the user and can be any continuous measurement. For example, you may be interested in genes that are correlated with alcohol preference in mice. Alcohol preference would need to be measured in several of the inbred or recombinant inbred strains within the PhenoGen data sets. A positive correlation between alcohol preference and the expression value of a transcript would indicate that in strains where the expression for that transcript is high the mice also prefer more alcohol than mice whose expression for that transcript is low. A negative correlation would indicate an inverse relationship where a high transcript expression value is associated with a lower alcohol preference. The use of inbred and recombinant inbred strains allows for valid correlation analyses when the phenotype and the expression data are measured on different populations of mice in different laboratories.

For the correlation analysis, mean expression values are calculated within strain. These mean values are correlated with the strain phenotypic measures that are uploaded by the user. The user has the choice of either using a Pearson Correlation Coefficient or a Spearman Rank Correlation Coefficient. The Pearson correlation coefficient is a parametric test for a linear relationship between two variables, where as a Spearman rank correlation is a non-parametric test that looks at the correlation of the ranks of the values rather than the actual values. A non-parametric test is necessary when the distribution of either of the variables is not normal. A parametric test will be more powerful when the two variables involved are truly normally distributed, but a non-parametric test is more accurate when this normality assumption is not met.

Multiple Comparison Correction Testing

There are eleven options for multiple comparison p-value adjustment that are split into four categories.

False Discovery Rate

1. Benjamini and Hochberg (BH)
2. Benjamini and Yekutieli (BY)
3. Storey

References

Benjamini Y and Hochberg Y (1995). Controlling the false discovery rate: a practical and powerful approach to multiple testing. JR Statist Soc B 57:289-300.

Benjamini Y and Yekutieli D (2001). The control of the false discovery rate in multiple hypothesis testing under dependency. Annals of Statistics 29(4):1165-1188.

General

4. Bonferroni
5. Holm
6. Hochberg
7. Sidak Single Step (SidakSS)
8. Sidak Step-Down (SidakSD)

References

Dudoit S, Shaffer JP, Boldrick JC (2002). Multiple hypothesis testing in microarray experiments. UC Berkeley Division of Biostatistics Working Paper Series, paper 110. http://www.bepress.com/ucbbiostat/paper110.

Permutation

9. minP with permutation (only available for two run groups)
10. maxT with permutation (only available for two rungroups)

References

Westfall PH and Young SS (1993). *Resampling-based multiple testing: Examples and methods for p-value adjustment*, John Wiley & Sons.

None

11. No Test

All multiple comparison adjustments are conducted in R. Adjustments 1, 2 and 4 through 8 are done using the R function “mt.rawp2adjp”. Adjustment 9 uses “mt.maxT” and adjustment 10 uses “mt.minP”.

For several methods, the only parameter that needs to be specified to complete the analysis is the threshold value for α. However with the two methods of adjustment that require permutation (9 and 10),

two more parameters must be set. These two methods require that the statistical analysis be done for each permutation. One of the additional parameters that needs to be set for the permutation options is the type of test to be conducted. The choice here overrides the results from the statistical analysis specified previously. The other parameter that needs to be specified is the number of permutation to use in estimating the p-value.

Note: The last method, 'No Test', does not contain an adjustment. This provides the option of not using any of the multiple comparison adjustments.

Applying Filters and Performing Statistical Analysis

After an experiment is normalized, you can apply filters to it and perform statistical analysis. The *Filter* page can be accessed in three ways:

- Select **Experiments menu > Data Analysis**.
- Select **Experiments menu > View All Experiments**, choose an experiment version, select **Data Analysis** from the Experiment Tools drop-down, and click **Go**.
- Select **Gene Lists menu > Create Gene List > From Experiment.**

On the *Filters* page:

1. Select an **Experiment Version** from the drop-down list.
2. Click **Go**.

   Notes:

   - You can select **View Normalization Parameters** to view the normalization parameters.
   - For correlation analysis experiments, the filters apply to the arrays contained in the PhenoGen data set chosen.

Filtering a Normalized Experiment

3. Choose a gene filtering method.

   #### Filtering Affymetrix Arrays

   If your experiment uses Affymetrix arrays, choose:

   - **Affy Control Genes -** Proceed to step 4.
   - **MAS5 Absolute Call Filter -** Specify whether to **Keep** or **Remove** genes and the values for run groups, then proceed to step 4.
   - **Negative Control Filter -** Specify whether to **Keep** or **Remove** genes and the values for run groups, then proceed to step 4

Filtering CodeLink Arrays

If your experiment uses CodeLink arrays, choose:

- **CodeLink Control Genes Filter -** Proceed to step 4.
- **CodeLink Call Filter -** Specify whether to **Keep** or **Remove** genes and the values for run groups, then proceed to step 4.
- **GeneSpring Call Filter -** Specify whether to **Keep** or **Remove** genes and the values for run groups, then proceed to step 4.
- **Median Filter -** Specify the **Filter** threshold.
- **Coefficient Variation Filter -** Specify **and** or **or** and the value for run groups, then proceed to step 4.
- **Negative Control Probe Filter -** Specify trim percentage, then proceed to step 4.

4. Click **Run Filter** to run the filtering criteria.
5. After the filter runs, you have the option to refine your filtering criteria.

- Enter new filter criteria and click **Run Filter** to re-run filtering.
- Click **Ignore Last Filter** to revert back to the gene list that existed prior to the last filter.
- Click **Ignore All Filters** to revert back to the gene list that existed before any filtering occurred.

After you are satisfied with the filtered gene list, proceed with statistical analysis to select differentially expressed genes.

Statistically Analyze a Normalized Experiment

6. Click **Statistics** to proceed to the *Statistics* page. You can run statistics for a saved experiment version multiple times after filtering.

Notes:

- Statistical analysis tools can also be accessed by selecting **Gene List menu > Create Gene List > From Experiment**.
- You can select **View Normalization Parameters** to view the normalization parameters.
- You can select **View Filters Used** to view the filter parameters.

7. Select the **Statistics Parameter Method**. Based on the number of run groups you have you may need to:

    - Select **parametric** or **non-parametric**
    - Select **pearson** or **spearman**
    - Select **1-Way ANOVA**, then select the **1-Way ANOVA Parameter**.
    - Select **2-Way ANOVA**, then select the **P-value of interest** and choose the **2-Way ANOVA Factors**.

8. Select the **Multiple Test Parameters**, if applicable. See the topic *Statistical Analysis Overview: Multiple Comparison Correction Testing* for details.

   FDR

   Benjamini and Hochberg

   Benjamini and Yekutieli

   Storey

   General

   Bonferroni

   Holm

   Hochberg

   SidakSS

   SidakSD

   Permutation (only available for two run groups)

   minP

   maxT

   None

   No Test

9. Based on your selection in step 8, you may need to:

   - Enter an **alpha level threshold**.
   - Select an **alpha or multiple correction threshold** from a drop-down list.
   - Enter an **alpha level threshold**, select the **type of test for permutation**, and select the **number of permutations**.

10. Click **Submit**. The statistical analysis runs.
11. Click **Save Gene List**. The *Save Gene List* page displays.

    Note: To optimize computational time and speed required for further analysis (such as Advanced Annotation, Promoter Analysis, and Literature Search) the number of differentially expressed genes should be less than 200.

12. Enter a **name** for the gene list.
13. Enter a **description** for the gene list. *OPTIONAL* - Click the **Set description to the parameters used** checkbox to automatically populate the description field with the filtering and statistical analysis parameters you used.
14. Click **Save Gene List**. The gene list is saved. You can view the gene list on the *View All Gene Lists* page.

Viewing Gene Expression Data

The *View Gene Expression* page allows you to obtain gene expression intensity data for a gene or gene list from a given experiment. In order to select the desired array expression data, a specific experiment is chosen. Specific genes may be typed in using any identifier, or a pre-created gene list can be used. The results table displays gene identifiers, probe IDs, group means, and group standard error.

1. Select **Experiments menu > View Gene Expression Data**. The *View Gene Expression Data* page displays.
2. Select an experiment version from the drop-down list and click **Go**.
3. Type in gene identifiers OR select a gene list.
4. Click **View**. The gene expression data displays.
5. Click **Download** at the bottom of the page to save the information.
6. Follow the instructions that display as you open or save the files. These instructions vary depending on your Internet browser (e.g., Internet Explorer, Firefox, etc.).

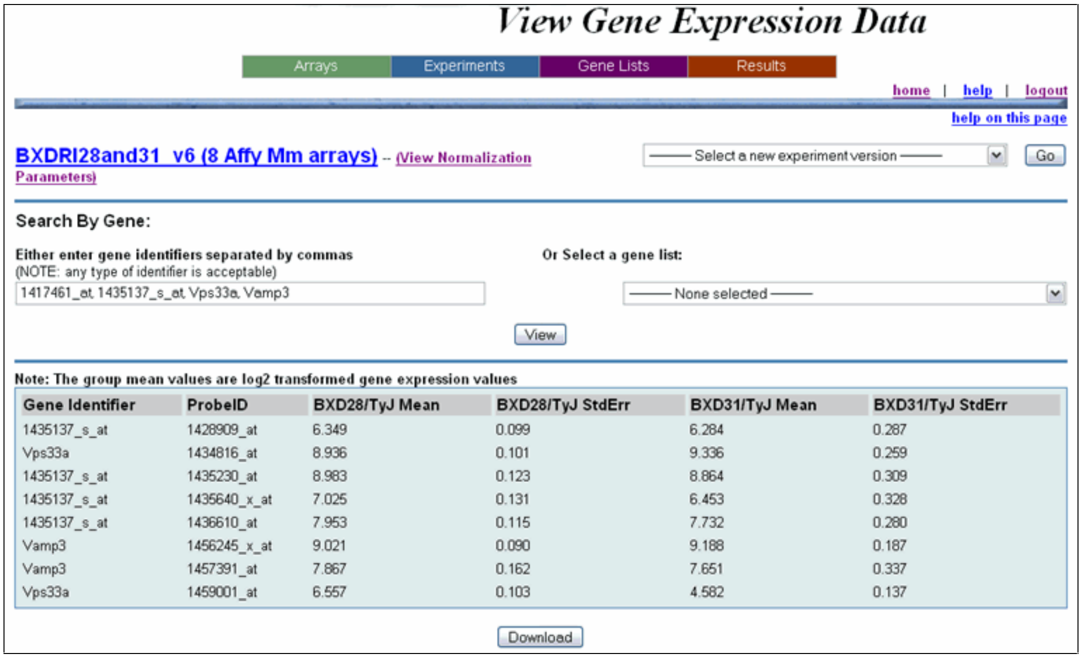

Viewing Gene Lists for Experiments

You can view the gene lists associated with your experiment versions.

1. Select **Experiments menu > View Gene Lists for Experiment**. The *Gene Lists for Experiments* page displays.
2. Select an **Experiment Version** from the drop-down list.
3. Click **Go**. The gene lists for that experiment version display. See the topic *View All Gene Lists* for details.

Downloading an Experiment

You can download raw data files that pertain to a combined array experiment or download grouped and normalized versions of the combined array experiment.

1. Select **Experiments menu > Download Experiment**. The *Download Experiment* page displays.
2. Select an experiment version from the drop-down list.
3. Click **Go**. The normalized data files for each version of the experiment and the raw data files associated with that experiment version display.

   Note: If there are no normalized data files, only raw data files display.
4. Click the checkbox(es) next to the data files you want to download. You have the option to **Check All** or **Uncheck All**.
5. Click **Download**. You have the option to open or save the files.
6. Follow the instructions that display as you open or save the files. These instructions vary, depending on your Internet browser (e.g., Internet Explorer, Firefox, etc.).

Gene Lists

Gene Lists Overview

Gene lists can be created, copied and modified, deleted, uploaded, and downloaded. When you have a gene list in the application, you can interpret the gene list using Basic and Advanced Annotation, Quantitative Trait Loci (QTL) tools, Literature Search, Promoter Analysis, Upstream Sequence Extraction, and Genetic Variation Data Retrieval.

Gene lists can be created from:

- Experimental Analysis
- Existing gene lists

Gene lists can also be created through:

- Manual entry
- Uploading

Gene lists can be published to other users if you are the owner of the gene list.

See the topics *Viewing User Access to a Gene List* for instructions on publishing gene lists and *Creating a Gene List* for instructions on all methods of creating gene lists.

Gene Lists Menu

The *Gene Lists* menu displays when you move your mouse over the **Gene Lists** button.

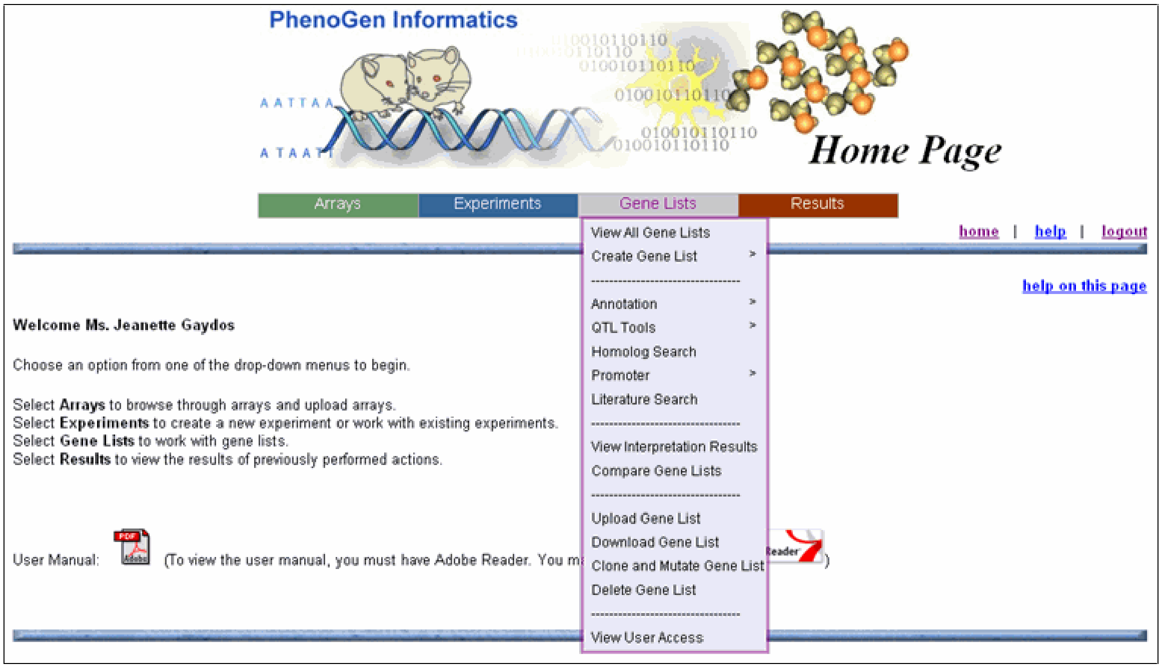

Drop-down Menu Option	Description
View All Gene Lists	Allows you to view all gene lists to which you have access.
Create Gene List	Opens a sub-menu where you can select:<br>• *From Experiment* - Allows you to select an experiment version and perform filtering and statistical analysis.<br>• *From Existing Gene List* - Allows you to copy an existing gene list.<br>• *From Uploaded File* - Allows you to upload a gene list.<br>• *Manually Enter Gene List* - Allows you to enter a gene list manually.
Annotation	Opens a sub-menu where you can select:<br>• *Basic Annotation* - Allows you to perform basic annotation on a gene list.<br>• *Advanced Annotation* - Allows you to perform advanced annotation on a gene list.
QTL Tools	Opens a page where you can select:<br>• *Define QTLs* - Allows you to define the number of QTLs you would like for a Phenotype or for a QTL list.<br>• *View QTL Lists* - Allows you to look at or delete the QTL Lists created in *Define QTLs.*<br>• *QTL Query* - Allows you to match the genetic location of the differentially expressed genes using a tool that can match these locations to chromosomal regions associated with certain phenotypes (bQTL).<br>• *Mouse eQTL Explorer* - Allows you to run eQTL Explorer using the PhenoGen mouse eQTL database for a gene list.<br>• *Rat eQTL Explorer* - Allows you to run eQTL Explorer using the PhenoGen rat eQTL database for a gene list.

Homolog Search	Allows you to obtain information regarding chromosomal location, for genes in a given gene list, in other genomes.
Promoter	Opens a sub-menu where you can select:<br>• *Promoter Analysis oPOSSUM* - Allows you to run oPOSSUM, which is a tool for determining the over-representation of Transcription Factor Binding Sites (TFBS) within a set of (co-expressed) genes as compared with a pre-compiled background set.<br>• *Promoter Analysis MEME* - Allows you to discover motifs in groups of related DNA or protein sequences.<br>• *Upstream Sequence Extraction* - Allows you to extract upstream sequences for the genes in a gene list.<br>• *Genetic Variations* - Allows you to obtain information about genetic variations (e.g. SNPs and polymorphisms) in the transcripts of interest.
Literature Search	Allows you to use an automated literature search tool that is tailored to your area(s) of interest when you select a set of query terms.
View Interpretation Results	Allows you to view literature search and promoter analysis results for a selected gene list.
Compare Gene Lists	Allows you to compare gene lists side-by-side, with the option to create unions, intersection, A-B, and B-A
Upload Gene List	Allows you to upload a gene list.
Download Gene List	Allows you to download a gene list.
Clone and Mutate Gene List	Allows you to copy and modify an existing gene list.
Delete Gene List	Allows you to delete gene lists that you own.
View User Access	Allows you to view the users who have access to your gene lists.

Viewing a Gene List

Click **Gene Lists menu > View All Gene Lists** to open the *View All Gene Lists* page.

Working with Gene Lists

The *View All Gene Lists* page allows you to work with gene lists.

1. Select a gene list.
2. Select an action from the drop-down list:

   - **Basic Annotation -** Allows you to perform basic annotation on a gene list.
   - **Advanced Annotation -** Allows you to perform advanced annotation on a gene list.
   - **QTL Query -** Allows you to perform a QTL Query.
   - **Homolog Search -** Allows you to obtain information regarding chromosomal location in other genomes.
   - **Promoter Analysis oPOSSUM -** Allows you to run promoter analysis using oPOSSUM on a gene list.
   - **Promoter Analysis MEME -** Allows you to discover motifs in groups of related DNA or protein sequences.
   - **Upstream Sequence Extraction -** Allows you to extract upstream sequences for the genes in a gene list.
   - **Genetic Variations -** Allows you to obtain information about genetic variations in the transcripts of interest.
   - **Literature Search -** Allows you to perform a literature search for a gene list.
   - **User Access -** Allows you to view users who have access to a gene list.
   - **Download -** Allows you to download a gene list.
   - **Clone and Mutate -** Allows you to copy and modify a gene list.
   - **Delete Gene List -** Allows you to delete a gene list.

3. Click **Go**. The page that pertains to your selection displays.

Note: You can only view gene lists for which you have permission. See the topic *Viewing User Access to a Gene List*.

Viewing Gene List Details

To view details for a gene list:

- Select the gene list from the *View All Gene Lists* page.
- Click the gene list name on any page where it displays.

The resulting *Gene List Details* page provides basic information about a gene list such as ownership, description, number of genes, organism, and source. It also provides links to the filters, normalization parameters, and statistical analysis that were used to create the gene list. Links to view oPOSSUM results, MEME results, and literature searches are also provided. In addition, it displays gene identifiers, gene symbols, the raw p-values and the adjusted p-values. Depending on the number

of run groups, it may also display group 1 and group 2 means, average of all arrays, F-statistic, mean intensity, correlation coefficient, difference in log base 2 intensity, t-statistic, or parameter estimates.

Note: If the gene list was uploaded, compared, or cloned and mutated, it will only display gene identifiers.

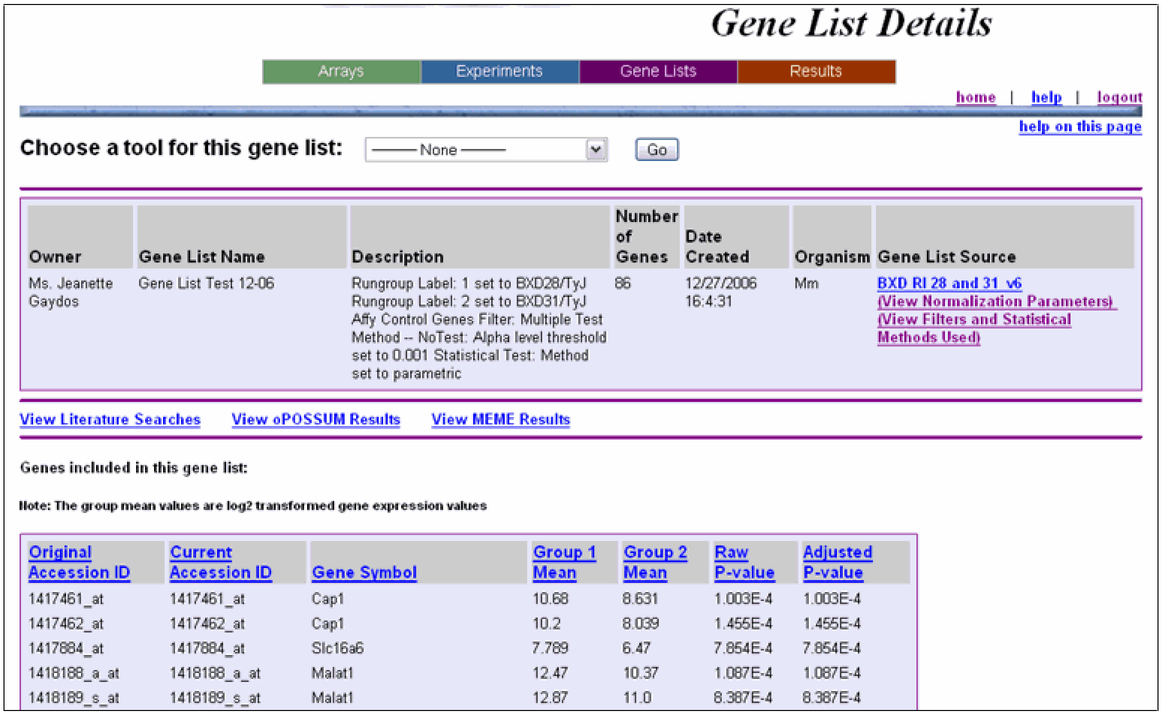

Gene List Details

Arrays | Experiments | Gene Lists | Results

home | help | logout

help on this page

Choose a tool for this gene list: —— None —— Go

Owner	Gene List Name	Description	Number of Genes	Date Created	Organism	Gene List Source
Ms. Jeanette Gaydos	Gene List Test 12-06	Rungroup Label: 1 set to BXD28/TyJ Rungroup Label: 2 set to BXD31/TyJ Affy Control Genes Filter: Multiple Test Method -- NoTest: Alpha level threshold set to 0.001 Statistical Test: Method set to parametric	86	12/27/2006 16:4:31	Mm	BXD RI 28 and 31_v6 (View Normalization Parameters) (View Filters and Statistical Methods Used)

View Literature Searches   View oPOSSUM Results   View MEME Results

Genes included in this gene list:

Note: The group mean values are log2 transformed gene expression values

Original Accession ID	Current Accession ID	Gene Symbol	Group 1 Mean	Group 2 Mean	Raw P-value	Adjusted P-value
1417461_at	1417461_at	Cap1	10.68	8.631	1.003E-4	1.003E-4
1417462_at	1417462_at	Cap1	10.2	8.039	1.455E-4	1.455E-4
1417884_at	1417884_at	Slc16a6	7.789	6.47	7.854E-4	7.854E-4
1418188_a_at	1418188_a_at	Malat1	12.47	10.37	1.087E-4	1.087E-4
1418189_s_at	1418189_s_at	Malat1	12.87	11.0	8.387E-4	8.387E-4

Creating a Gene List

Gene lists can be created:

- From an experiment (see the topic *Creating a Gene List from an Experiment*).
- From an existing gene list (see the topic *Cloning and Mutating a Gene List*).

Gene lists can also be entered manually or uploaded if you have an existing gene list that you want to analyze. See the topics *Manually Entering a Gene List* and *Uploading a Gene List*.

Creating a Gene List from an Experiment

You can create a gene list from an experiment version.

1. Select Gene Lists menu > Create Gene List > From Experiment. The *Filters* page displays.
2. Select an **Experiment Version** from the drop-down list.
3. Click **Go**. The experiment version displays.
4. Choose a gene filtering method.

   Filtering Affymetrix Arrays

   If your experiment uses Affymetrix arrays, choose:

   - **Affy Control Genes -** Proceed to step 5.
   - **MAS5 Absolute Call Filter -** Specify whether to **Keep** or **Remove** genes and the values for run groups, then proceed to step 5.
   - **Negative Control Filter -** Specify whether to **Keep** or **Remove** genes and the values for run groups, then proceed to step 5.

   Filtering CodeLink Arrays

   If your experiment uses CodeLink arrays, choose:

   - **CodeLink Control Genes Filter -** Proceed to step 5.
   - **CodeLink Call Filter -** Specify whether to **Keep** or **Remove** genes and the values for run groups, then proceed to step 5.
   - **GeneSpring Call Filter -** Specify whether to **Keep** or **Remove** genes and the values for run groups, then proceed to step 5.
   - **Median Filter -** Specify the **Filter** threshold.
   - **Coefficient Variation Filter -** Specify **and** or **or** and the value for run groups, then proceed to step 5.
   - **Negative Control Probe Filter -** Specify trim percentage, then proceed to step 5.

5. Click **Run Filter** to run the filtering criteria.
6. After the filter runs, you have the option to refine your filtering criteria.

   - Enter new filter criteria and click **Run Filter** to re-run filtering.
   - Click **Ignore Last Filter** to revert back to the gene list that existed prior to the last filter.

- Click **Ignore All Filters** to revert back to the gene list that existed before any filtering occurred.

After you are satisfied with the filtered gene list, proceed with statistical analysis to select differentially expressed genes.

7. Click **Statistics** to proceed to the *Statistics* page. You can run statistics for a saved experiment version multiple times after filtering.
8. Select the **Statistics Parameter Method**. Based on the number of run groups used, you may need to:
    - Select **parametric** or **non-parametric.**
    - Select **pearson** or **spearman.**
    - Select **1-Way ANOVA**, then select the **1-Way ANOVA Parameter**.
    - Select **2-Way ANOVA**, then select the **P-value of interest** and choose the **2-Way ANOVA Factors**.
9. Select the Multiple Test Parameters. See the topic *Statistical Analysis Overview: Multiple Comparison Correction Testing* for details.

   FDR

   Benjamini and Hochberg

   Benjamini and Yekutieli

   Storey

   General

   Bonferroni

   Holm

   Hochberg

   SidakSS

   SidakSD

   Permutation (available only for two run groups)

   minP

   maxT

   None

   No Test
10. Based on your selection in step 9, you may need to:

    - Enter an **alpha level threshold**.
    - Select an **alpha or multiple correction threshold** from a drop-down list.
    - Enter an **alpha level threshold**, select the **type of test for permutation**, and select the **number of permutations**.

11. Click **Submit**. The statistical analysis runs.
12. Click **Save Gene List**. The *Save Gene List* page displays.

13. Enter a **name** for the gene list.
14. Enter a **description** for the gene list. *OPTIONAL* **-** Click the **Set description to the parameters used** checkbox to automatically populate the description field with the filtering and statistical analysis parameters you used.
15. *OPTIONAL* - Click the checkbox beside each user who you want to view the gene list in the **Publish gene list to these users** section.
16. Click **Save Gene List**. The gene list is saved. You can view the gene list on the *View All Gene Lists* page.

Manually Entering a Gene List

1. Select **Gene Lists menu > Create Gene List > Manually Enter Gene List**. The *Create Gene List* page displays.

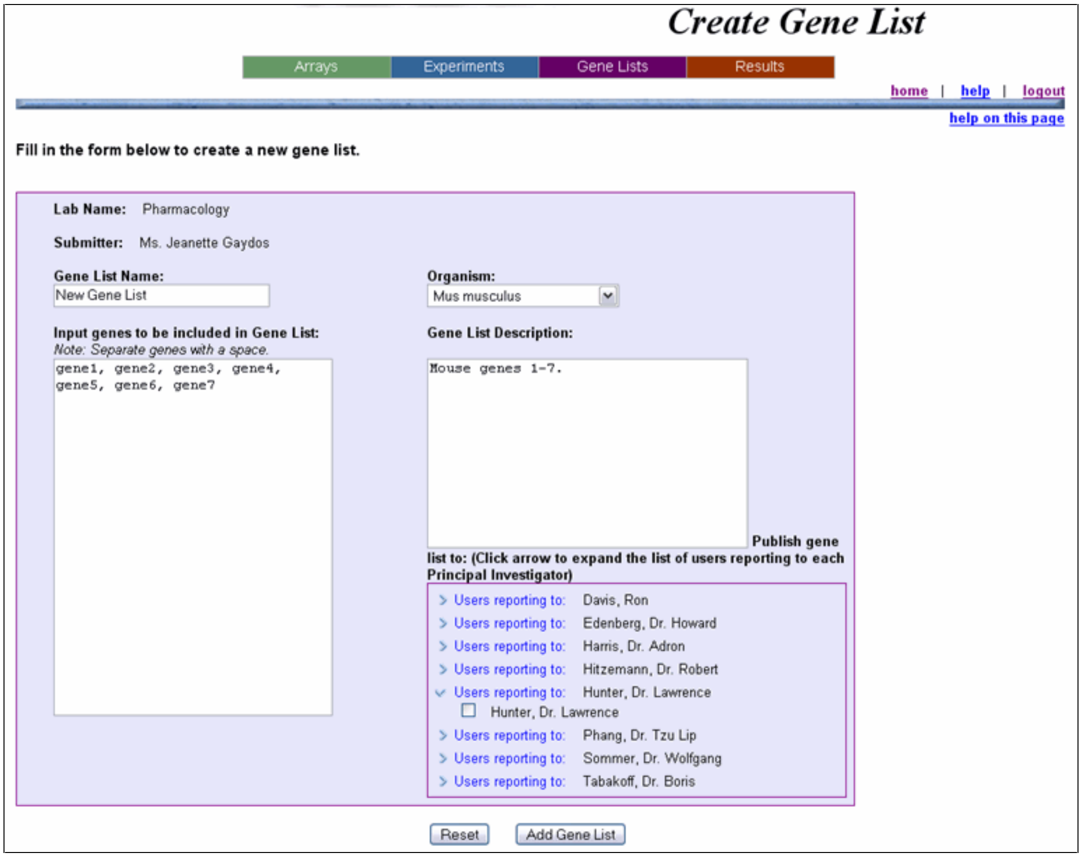

2. Enter the **Gene List Name** for the new gene list.
3. Select the **Organism** for the gene list from the drop-down list.
4. Type in the genes to include in the gene list. You must separate the genes with a space or by pressing **Enter** on the keyboard, or they cannot be processed correctly. Genes can be entered in the following formats:

Gene IDs	Example	Description
Affymetrix ID	1416283_at	
CodeLink ID	NM_009775_Probe1<br>GE34729	
Ensembl ID	ENSMUSG00000022962	Begins with ENS, followed by the three-letter organism, followed by G, followed by a number.
Entrez Gene ID	14450	Contains three or more numbers.
FlyBase Gene ID	FBgn0013277	All IDs are preceded by FBgn and have numbers following.
Gene Symbols	Grin1	Official gene symbols from MGI.
MGI ID	MGI:95654	
NCBI/EMBL RNA accession number	AK146355	Begins with one or two letters, followed by multiple numbers.
RefSeq RNA accession number	NM_010256	Begins with two capital letters, followed by an underscore, followed by numbers.
RGD ID	2203	Contains four or more numbers.
UniGene ID	Mm.4505	Contains an organism prefix (Dm, Hs, Rn, or Mm), followed by a period, followed by numbers.

Genes not entered in the formats above may not be recognized by the PhenoGen website when you perform analysis on a gene list, such as promoter analysis or basic annotation.

5. Enter the **Gene List Description**.
6. *OPTIONAL* - Click the checkbox beside each user who you want to view the gene list in the **Publish gene list to these users** section.
7. Click **Add Gene List**. The gene list is added.

Note: You can view the gene list on the *View All Gene Lists* page.

Annotating Gene Lists

Gene lists on the PhenoGen website can contain identifiers from any source, and can be translated using a tool called iDecoder. This tool translates identifiers to and from the following identifier types:

- AFFYMETRIX PROBESET ID
- CODELINK PROBESET ID
- ENSEMBL ID
- ENTREZ GENE ID
- FLYBASE ID
- GENE SYMBOL
- MGI ID
- REFSEQ PROTEIN ID
- REFSEQ GENE ID
- RGD ID
- SWISSPROT ID
- UNIGENE ID

The data used to translate these values comes from information downloaded from the following organizations:

- Affymetrix
- CodeLink (GE Healthcare)
- Ensembl
- FlyBase
- MGI
- NCBI
- RGD
- SwissProt

You can choose the type of official gene symbol that is sent to the databases then use Basic Annotation to create links between databases for that gene symbol. Note that you do not have to specify the ID type. In fact, the uploaded gene list can be a mixture of many ID types. For example, you can upload a gene list that contains a probe set ID, an official gene symbol, an Entrez Gene ID, and a RefSeq Gene ID, and iDecoder will generate a list of the most probable gene identifiers.

In addition to general annotation, the Basic Annotation function also provides information on expression QTL (eQTL) when the gene list was created based on mouse or rat data. A QTL column will appear in the *Basic Annotation* table and an INIA West eQTL link will appear when the gene list entry matches either a probe ID or gene symbol in the eQTL data. Mouse gene lists will also display links to the Allen Institute for Brain Science's Allen Brain Atlas. A column in the *Basic Annotation* table will display the Allen Brain Atlas links.

Expression QTL (eQTL)

The purpose of expression QTLs is to determine the location in the genome that controls the transcription level of a gene. eQTLs are calculated using traditional QTL techniques where the quantitative trait of interest is the expression level of a gene as measured by microarray analysis.

INIA West eQTLs have been calculated for both mouse and rat data. When either of these species is used to create a gene list, the eQTLs of the matching species are reported and genes are matched based on gene symbol. For mice, whole-brain gene expression data was obtained for a panel of 30 BXD RI strains plus the two parental strains on the MOE430v2 array from Affymetrix. Probe set intensities were normalized and summarized using RMA. For rats, whole-brain gene expression data was obtained for a panel of 25 HXB/BXH RI strains plus the two parental strains on the CodeLink Rat Whole Genome Array. The rat data was quantile-normalized after values were adjusted for local background and transformed using a log base 2. Individual values were eliminated if the value showed an irregular shape, contamination, or saturation.

For the mice, an original set of 3,795 markers and their genotypes for 89 BXD RI strains was downloaded from http://www.genenetwork.org/genotypes/BXD.geno. The location of these markers is based on Mouse Build 36-mm8 (updated January 2007). However, the set of markers used for the eQTL analyses was reduced from the original set to eliminate markers that had markers on either side of them with identical strain distributions (with respect to the 32 strains used in our analysis). This final set contained 943 informative markers. Genotype information for rats was downloaded from ftp://atlas.utmem.edu/public/HXB_Rat_UT_Aug06.xls. This set had 922 markers for 36 RI strains on 21 chromosomes (20 autosomal and 1 sex-linked). Unsupportable double recombinants were recorded and values were simulated for missing genotypes. Of the 922, 560 markers were used for eQTL calculations.

For both mice and rats, mean expression levels within strains were used as phenotypic values in a QTL analysis implemented in QTL Reaper, which is written in C and compiled as a Python module. QTL Reaper is downloadable from the following website, http://qtlreaper.sourceforge.net/ . A weighted marker regression analysis was used within this program to calculate LRS scores for each marker. LRS scores were transformed to LOD scores for convenience by dividing by 4.61. The regression is weighted to account for the different number of arrays within strains used to calculate strain means. The weight is based on the repeatability of the transcript intensity and the number of arrays used to calculate the strain mean (Carlborg et al. 2005). The empirical p-value with respect to the maximum LOD score was calculated for each transcript by permutation (Churchill and Doerge, 1994). The empirical p-value adjusts for the multiple comparisons due to the multiple markers per transcript, but not for the multiple comparisons due to the many transcripts. To adjust for the multiple comparisons due to many transcripts, false discovery rates were calculated according to Benjamini and Hochberg (1995).

In the *Expression QTL* table, the physical location of the gene represented by the probe set ID is shown, along with the location of the marker that represents the maximum LOD score for that transcript. The location of the marker with the maximum LOD score indicates the region of transcriptional control. For this analysis, if the physical location of a gene is near the location of transcriptional control, the gene is considered to be cis-regulated. Otherwise, the gene is considered trans-regulated. The physical location of mouse genes was determined from the Affymetrix annotation file released on November 15, 2006, which is based on the February 2006 version of NCBI Build 36. Physical locations for rat genes were determined by "BLAST"ing the probe sequence using NCBI, based on RCSC 3.4.

References

1. Carlborg O, De Koning DJ, Manly KF, Chesler E, Williams RW, and Haley, CS (2005). Methodological aspects of the genetic dissection of gene expression. Bioinfomatics 21(10), 2383-2393.
2. Churchill GA, Doerge RW (1994). Empirical threshold values for quantitative trait mapping. Genetics 138:963-971.
3. Benjamini Y and Hochberg Y (1995). Controlling the false discovery rate: a practical and powerful approach to multiple testing. J R Statist Soc B 57:289-300.

Allen Brain Atlas

PhenoGen users can obtain the regional expression pattern, in C57BL/6J mice, of a given gene in the gene list by simply clicking on the link provided in the **Allen Brain Atlas** column in the *Basic Annotation* table. There links are available ONLY for the mouse brain gene expression data. The Allen Brain Atlas (Lein et al., 2007) is an open-access database of gene expression in the C57Bl/6J brain tissue. This database was created by the Allen Institute for Brain Science (Seattle, Washington). The Atlas contains data for genome wide RNA expression obtained using high-throughput in-situ hybridization technique. In addition to the expression data, the Atlas also has a number of tools available for analyzing and visualizing the in-situ images. Comprehensive *Help* documentation is available online (http://www.brain-map.org).

References

1. Allen Brain Atlas [Internet]. Seattle (WA): Allen Institute for Brain Science. © 2004-2007. Available from: http://www.brain-map.org .
2. Lein ES et al. (2007). Genome-wide atlas of gene expression in the adult mouse brain. Nature 445:168-176, doi:10.1038/nature05453.
3. Markram H (2007). Industrializing neuroscience. Nature 445:160-161.

Basic Annotation Example

You can use iDecoder to map gene identifiers between databases. For instance, if database 1 contains entry A and database 2 contains entry B, and both A and B refer to entry C in database 3, but not to each other, iDecoder will identify that A and B are related. The method is very efficient in unearthing previously unknown equivalent IDs. iDecoder can be used to resolve ambiguities by replacing the **Current Identifier** with any of the equivalent IDs when you select the radio button next to the ID of choice. You can run iDecoder as often as needed and use the Current Identifier as input for each successive run.

The following image shows the result of an iDecoder run. The Original Identifiers were uploaded and the equivalent IDs for these genes from six specified resources were returned; Official Gene Symbol, NCBI's RefSeq, Jackson Laboratory's Mouse Genome Informatics (MGI), SwissProt's UniProt, University of California Santa Cruz, and Ensembl's TranscriptSNPView. These corresponding IDs were hyperlinked to their original sources. When you click a link, further annotation is displayed in a new window. In addition to these links, if there are any genetically modified mice available for a particular gene, information from the database of genetically modified mice (maintained by MGI) is provided. Similarly, a link to INIA West eQTL data for a particular gene is also provided, if available. For mouse gene lists, a link to the Allen Brain Atlas may also be displayed for a specific gene. The data in the *Basic Annotation* table may be sorted by **Currently Selected Identifier**, **Original Identifier**, **Genetically Modified Animal Available**, and **QTLs** by clicking on the appropriate column heading. Basic Annotation information can be downloaded by the user.

Note: Gene lists over 200 genes will be re-directed to Advanced Annotation, where the information can be downloaded in order to optimize computational time and speed.

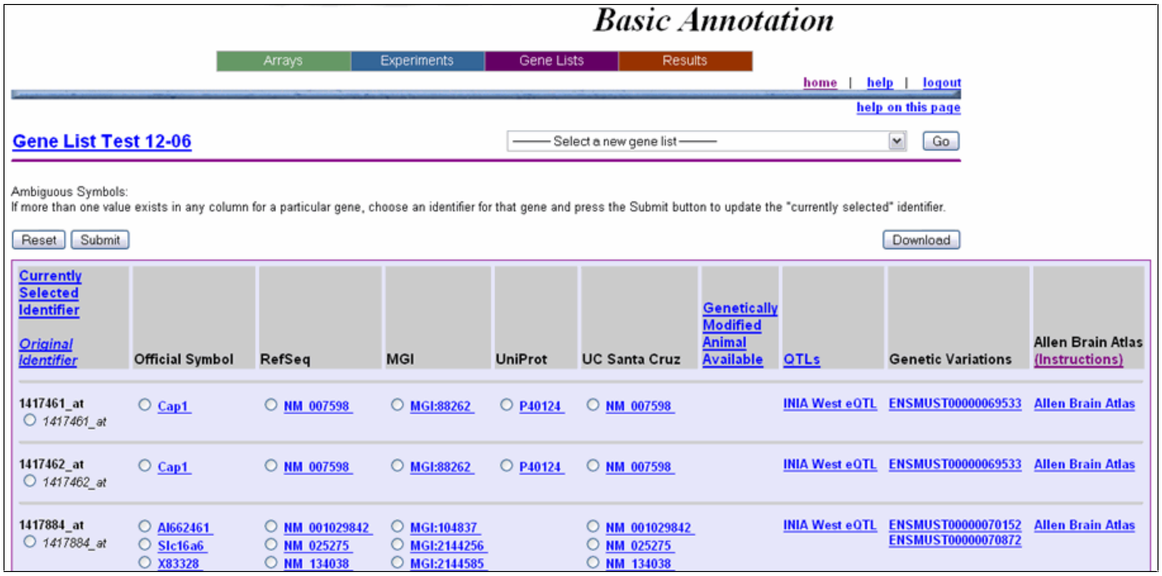

Advanced Annotation Example

In comparison, Advanced Annotation allows you to select “databases of interest” to populate the annotation for a given gene list. After you select a gene list, you can perform Advanced Annotation and select any OR all of the different databases to obtain further annotation. In addition to the information from these databases, users can also obtain synonyms (if any), the genomic location of the genes in the gene list, and genetic variation data. This information can be downloaded by the user.

Note: Gene lists over 200 genes can only be downloaded in order to optimize computational time and speed.

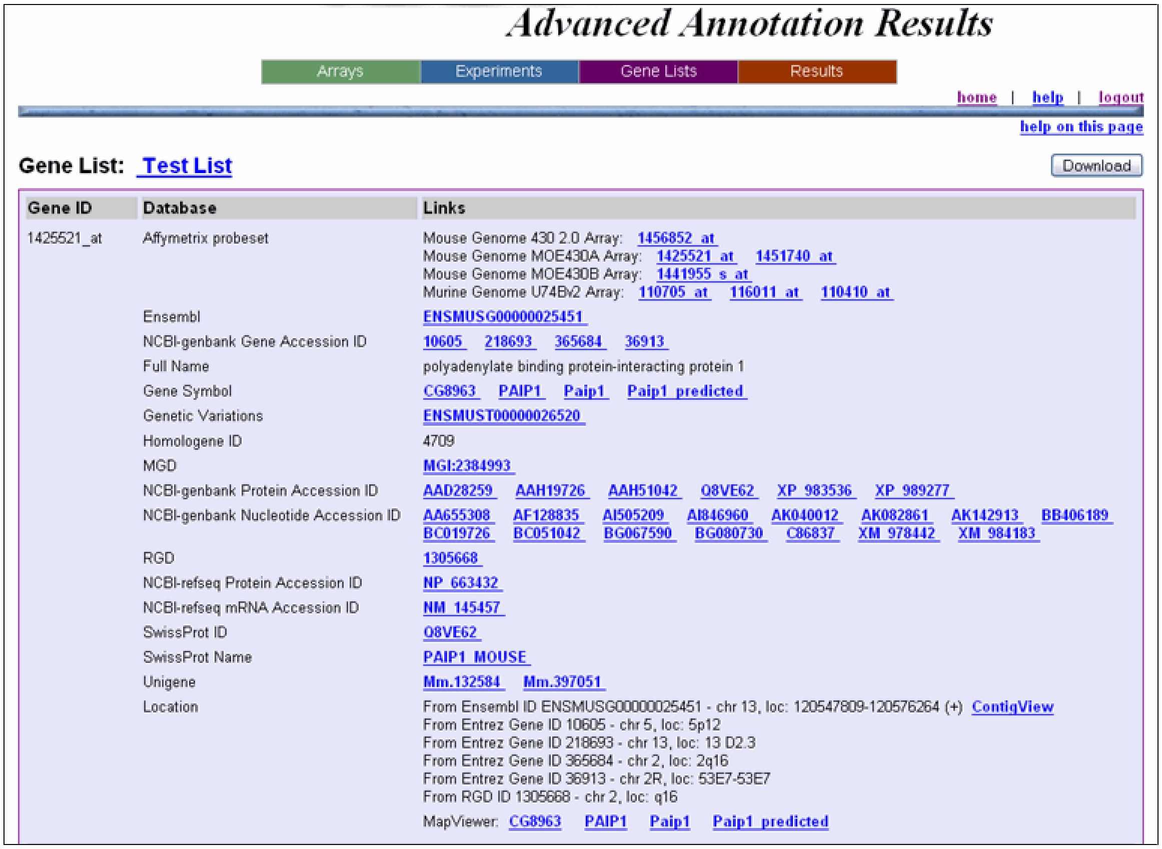
Advanced Annotation Results

Arrays | Experiments | Gene Lists | Results

home | help | logout

help on this page

Gene List: Test List

Download

Gene ID	Database	Links
1425521_at	Affymetrix probeset	Mouse Genome 430 2.0 Array: 1456852_at<br>Mouse Genome MOE430A Array: 1425521_at 1451740_at<br>Mouse Genome MOE430B Array: 1441955_s_at<br>Murine Genome U74Bv2 Array: 110705_at 116011_at 110410_at
	Ensembl	ENSMUSG00000025451
	NCBI-genbank Gene Accession ID	10605 218693 365684 36913
	Full Name	polyadenylate binding protein-interacting protein 1
	Gene Symbol	CG8963 PAIP1 Paip1 Paip1_predicted
	Genetic Variations	ENSMUST00000026520
	Homologene ID	4709
	MGD	MGI:2384993
	NCBI-genbank Protein Accession ID	AAD28259 AAH19726 AAH51042 Q8VE62 XP_983536 XP_989277
	NCBI-genbank Nucleotide Accession ID	AA655308 AF128835 AI505209 AI846960 AK040012 AK082861 AK142913 BB406189<br>BC019726 BC051042 BG067590 BG080730 C86837 XM_978442 XM_984183
	RGD	1305668
	NCBI-refseq Protein Accession ID	NP_663432
	NCBI-refseq mRNA Accession ID	NM_145457
	SwissProt ID	Q8VE62
	SwissProt Name	PAIP1_MOUSE
	Unigene	Mm.132584 Mm.397051
	Location	From Ensembl ID ENSMUSG00000025451 - chr 13, loc: 120547809-120576264 (+) ContigView<br>From Entrez Gene ID 10605 - chr 5, loc: 5p12<br>From Entrez Gene ID 218693 - chr 13, loc: 13 D2.3<br>From Entrez Gene ID 365684 - chr 2, loc: 2q16<br>From Entrez Gene ID 36913 - chr 2R, loc: 53E7-53E7<br>From RGD ID 1305668 - chr 2, loc: q16<br>MapViewer: CG8963 PAIP1 Paip1 Paip1_predicted

Performing Basic Annotation

The *Annotation* page can be accessed in two ways:

- Select **Gene Lists menu > Basic Annotation**.
- Select **Gene Lists menu > View All Gene Lists**, choose a gene list, select **Basic Annotation** from the Gene List Tools drop-down, and click **Go**.

1. Choose a gene list from the drop-down list.
2. Click **Go**. The *Basic Annotation* page displays.

   The basic annotation table lists the equivalent ID from five different ID types:

   - Official Symbol
   - RefSeq
   - MGI (Mouse Genome Informatics), RGD (Rat Genome Database), or FlyBase
   - UniProt (Swiss Prot)
   - UC Santa Cruz (University of California, Santa Cruz)

   It also lists:

   - Genetically Modified Animal Available
   - QTLs
   - Genetic Variations
   - Allen Brain Atlas (if applicable)

3. Click any of the different links to open the website or information page for the link you selected.

iDecoder in Basic Annotation is an ambiguity resolver, which allows you to identify a more appropriate representation of the genes. For example, after some investigation you decide one gene identifier is a better representation than the original one; you can replace it by selecting the preferred ID and clicking the **Submit** button. This replaces the old ID with the new in the **Currently Selected Identifier** field. Note that the Original Identifier does not change. The Original Identifier is always in *ITALICS* and below the Current Identifier. If the Original Identifier has not been changed, the Current and Original Identifier are the same.

Performing Advanced Annotation

Advanced Annotation allows you to select the gene list you want to annotate, and then select the links you want iDecoder to search for. The *Advanced Annotation* page can be accessed in two ways:

- Select **Gene Lists menu > Annotation > Advanced Annotation**.
- Select **Gene Lists menu > View All Gene Lists**, choose a gene list, select **Advanced Annotation** from the Gene List Tools drop-down, and click **Go**.

On the *Advanced Annotation* page:

1. Choose a gene list from the drop-down list.
2. Click **Go**. The *Advanced Annotation* page displays.
3. Select one or more target database links and array name(s), if desired.

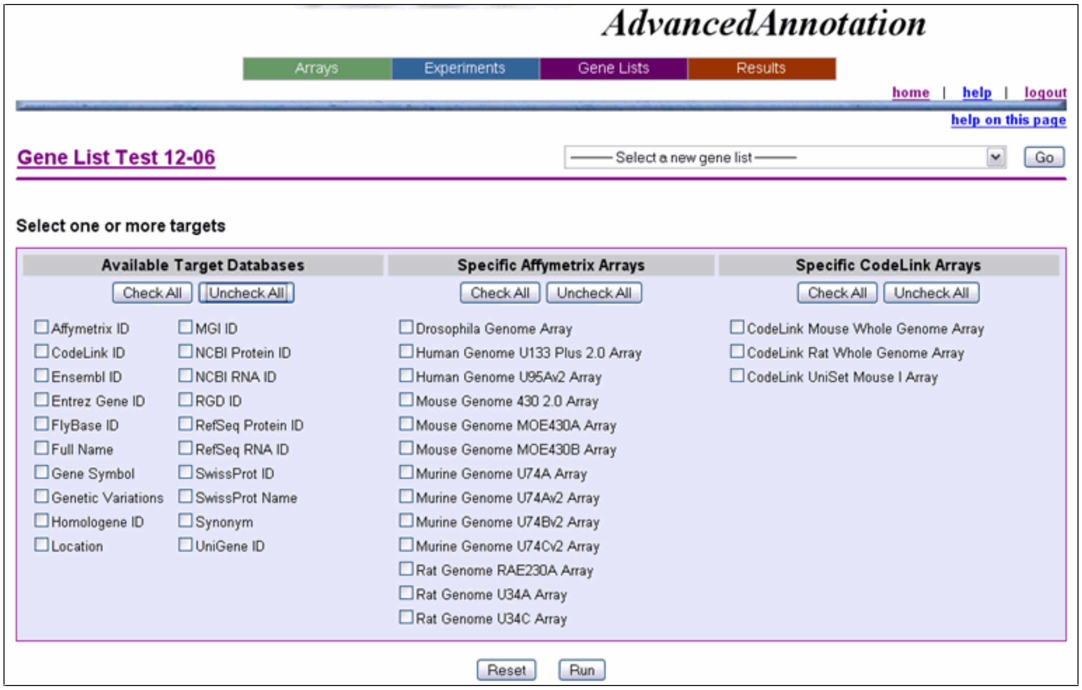

4. Click **Run**. The *Advanced Annotation Results* page displays. It shows you the Gene ID, the databases searched, and the links found in each database.
5. Click any of the different links to open the website for the link you selected.

Using QTL Tools

Quantitative Trait Loci (QTL) Tools can use the QTLs from the Mouse Genome Informatics (MGI) and the RGD databases and can be supplemented with additional QTLs that have been generated by literature review. QTL Tools uses a centralized QTL page to find the genetic location of the differentially regulated genes and a tool that matches these locations to QTL-based specifications of chromosomal regions.

QTL Tools

The website has tools that allow you to assess whether the genomic location of any of the differentially expressed genes (i.e., list of genes obtained using data analysis tools on the website or uploaded to the website) fall within the QTL regions for the behaviors of your choice. Information about the behavioral QTLs can be obtained from MGI (mouse QTLs) or RGD (rat and mouse QTLs).

You can define the QTL regions for a given behavior using the **Define QTL** tool.

You can use these defined QTLs to assess the overlap of these QTL regions with genomic locations of genes in any of the gene lists to which you have access using the **QTL Query** tool. The **QTL Query** tool also determines if an expression QTL for any of the genes in the list falls within the behavioral QTLs you defined. At present, eQTL information is available ONLY for the mouse genome.

eQTL Explorer is a Java-based tool for managing and visualizing “expression” and “behavioral” QTL data. You need the Sun Microsystems JRE, version 1.4.2 or greater, installed on your desktop. This software can be obtained from www.sun.com.

See the topic *Expression QTL (eQTL)* under *Annotating Gene Lists* for details on calculations of eQTLs.

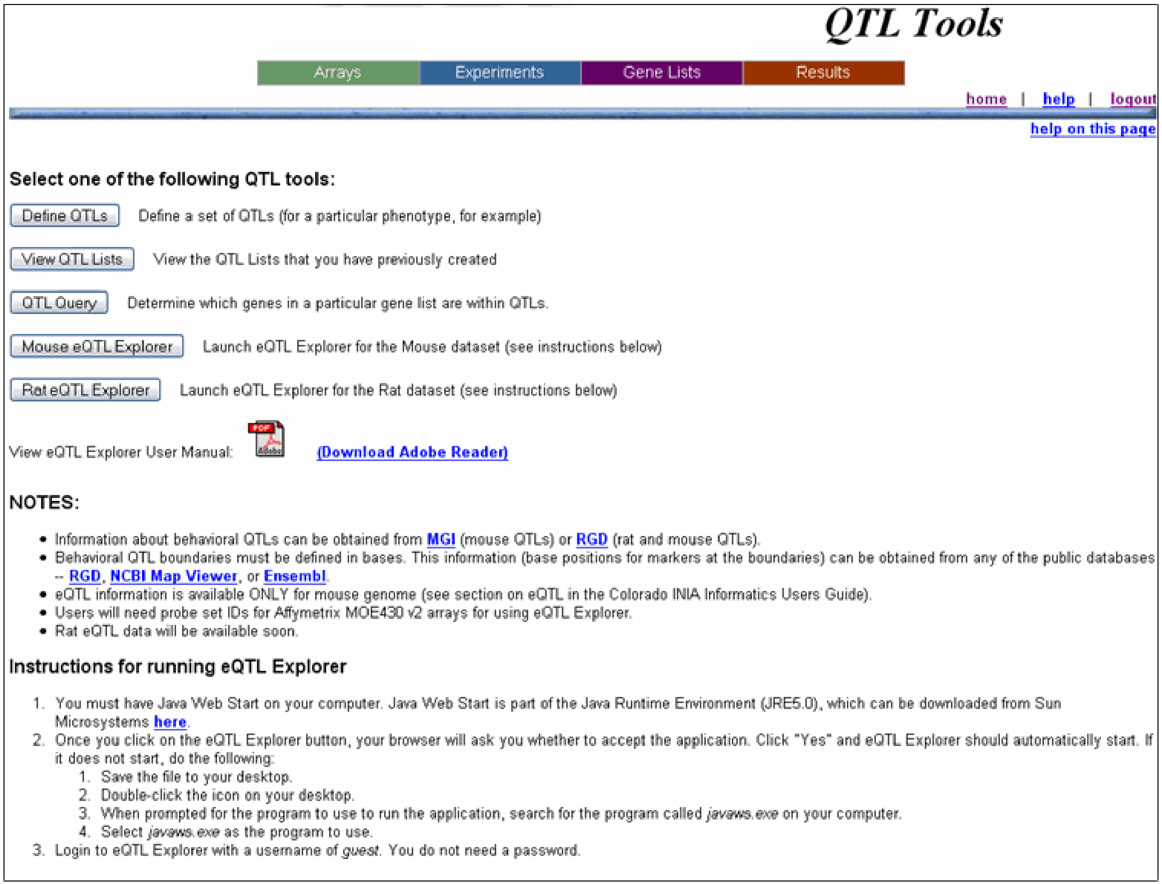

Defining QTLs

The *Define QTLs* page allows you to create a list of QTLs for a phenotype. For instance, you could define a number of QTLs from an alcohol phenotype.

1. Select **Gene Lists menu > QTL Tools**. The *QTL Tools* page displays.
2. Click **Define QTLs**. The *Define QTLs* page displays.
3. Click **Go**.
4. Enter the **Phenotype** or **QTL List Name**. This field is referenced on the *QTL Query* page.
5. Select the organism to which this list pertains.
6. Enter the **QTL** Identifier, **Chromosome**, and **Start** and **End** base pairs (bp).
7. To add another QTL, click on **Add New QTL** and repeat step
8. Click **Save QTL List**.

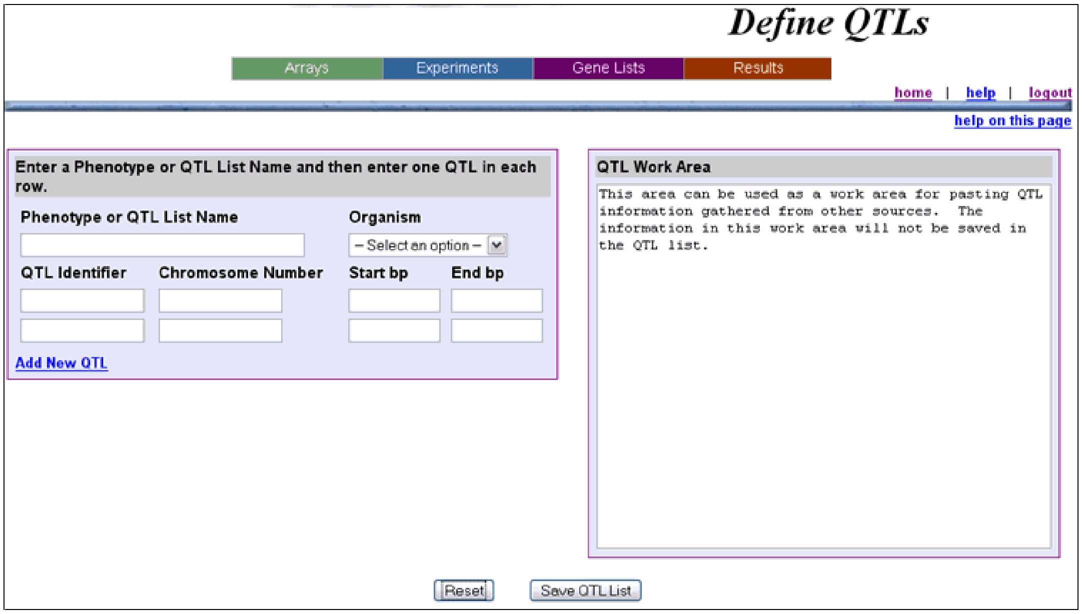

Viewing All QTL Lists

The *View All QTL Lists* page allows you to see all of the QTL lists that you have created for any organism. It also allows you to delete any of these lists.

- Select **Gene Lists menu > QTL Tools.** The *QTL Tools page* displays.
- Click **View QTL Lists**. The *QTL Lists* page displays.

The *QTL Lists* page displays the QTL lists that were created using the **Define QTLs** tool. By clicking on the **QTL List Name**, it will bring up the **QTL Lists Contents** for you to view. To delete any list, click **Delete**. You will then be prompted to confirm the deletion

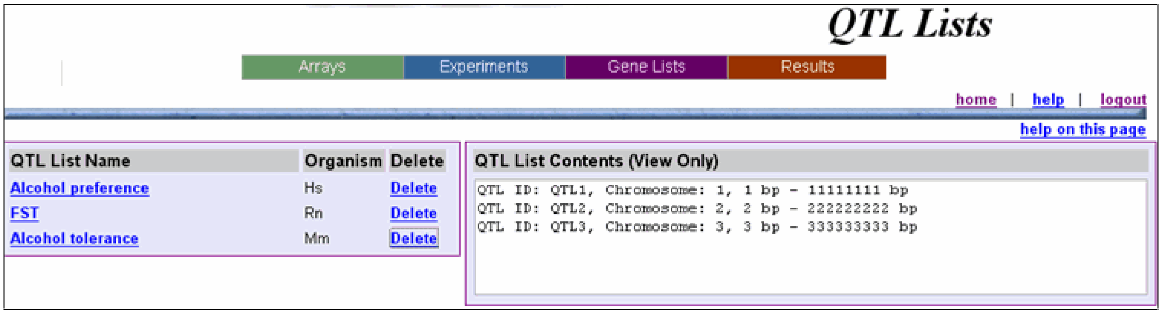

QTL Query

The QTL Query page allows you to use QTL Lists you previously defined to run a QTL query on a gene list. The *QTL Query* page can be accessed in two ways:

- Select **Gene Lists menu > QTL Tools**. The *QTL Tools* page displays. Click **QTL Query**.
- Select **Gene Lists menu > View All Gene Lists**, choose a gene list, select **QTL Query** from the Gene List Tools drop-down, and click **Go**.

Note: You must define QTLs before you can use this page. See the topic *Defining QTLs* for details.

On the *QTL Query* page:

1. Select a gene list from the drop-down list.
2. Click **Go**.
3. Select a **Phenotype** or **QTL List**. The drop-down is populated with items you created in the *Define QTLs* page. The QTL List Contents displays.
4. Select a p-value cut off for MaxLOD scores for eQTL data.
5. *OPTIONAL* – Type additional genes into the **Include these genes in list** field. Separate the genes with a space or press the **Enter** key on your keyboard between entries or the entries will not be processed correctly. Genes can be entered in the following formats:

Gene IDs	Example	Description
Affymetrix ID	1416283_at	
CodeLink ID	NM_009775_Probe1<br>GE34729	
Ensembl ID	ENSMUSG00000022962	Begins with ENS, followed by the three-letter organism, followed by G, followed by a number.
Entrez Gene ID	14450	Contains three or more numbers.
FlyBase Gene ID	FBgn0013277	All IDs are preceded by FBgn and have numbers following.
Gene Symbol	Grin1	Official gene symbols from MGI.
MGI ID	MGI:95654	
NCBI/EMBL RNA accession number	AK146355	Begins with one or two letters, followed by multiple numbers.
RefSeq RNA accession number	NM_010256	Begins with two capital letters, followed by an underscore, followed by numbers.
RGD ID	2203	Contains four or more numbers.
UniGene ID	Mm.4505	Contains an organism prefix (Dm, Hs, Rn, or Mm), followed by a period, followed by numbers.

5. OPTIONAL - Enter a new name for the gene list to save the gene list as a new list when you click submit.
6. Click **Submit**.

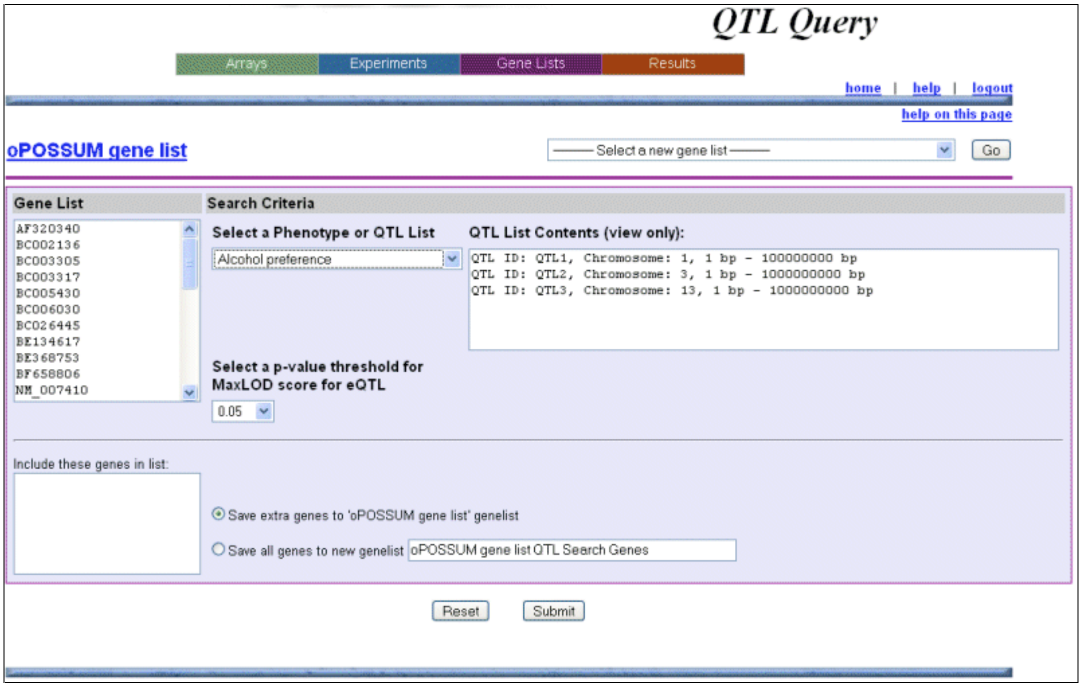
QTL Query
Arrays
Experiments
Gene Lists
Results
home | help | logout
help on this page
oPOSSUM gene list
Select a new gene list
Go
Gene List
Search Criteria
AF320340
BC002136
BC003305
BC003317
BC005430
BC006030
BC026445
BE134617
BE368753
BF658806
NM_007410
Select a Phenotype or QTL List
Alcohol preference
QTL List Contents (view only):
QTL ID: QTL1, Chromosome: 1, 1 bp - 100000000 bp
QTL ID: QTL2, Chromosome: 3, 1 bp - 1000000000 bp
QTL ID: QTL3, Chromosome: 13, 1 bp - 1000000000 bp
Select a p-value threshold for MaxLOD score for eQTL
0.05
Include these genes in list:
Save extra genes to 'oPOSSUM gene list' genelist
Save all genes to new genelist
oPOSSUM gene list QTL Search Genes
Reset
Submit

QTL Query Results

The first column under **Results** in the *QTL Query Results* page displays the genes from the gene list that were physically located in the QTLs you entered. The second column in the **Results** shows the genes where the 95% confidence intervals for location of their expression QTLs overlaps QTL. The confidence intervals are derived using the bootstrap method (Visscher et al 1996). The genes displayed in both **Results** columns are hyperlinked to the *Expression QTLs* page. See the topic *Expression QTL (eQTL)* for more information.

Visscher PM, Thompson R, Haley CS (1996). Confidence intervals in QTL mapping by bootstrapping. Genetics 143:1013-1020.

- Click **View Results of Gene/Other ID Mapping** to view the IDs and alternates found by iDecoder for the original gene list.

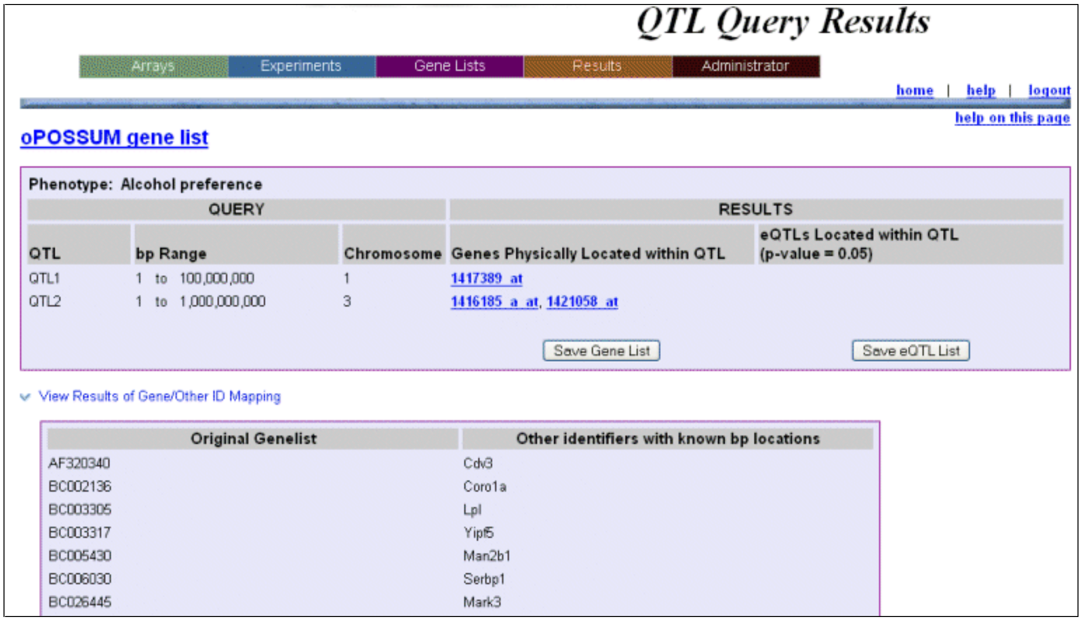

eQTL Explorer

eQTL Explorer is a tool that stores expression profiles, linkage data, and information from external sources in a relational database and enables simultaneous visualization and intuitive interpretation of the combined data via a Java graphical interface. eQTL Explorer provides a new and powerful tool to interrogate these very large and complex datasets. The PhenoGen website utilizes a local copy of eQTL Explorer that references two PhenoGen created databases for both mouse and rat.

1. Select **Gene Lists menu > QTL Tools**. The *QTL Tools* page displays.
2. Click either **Mouse eQTL Explorer** or **Rat eQTL Explorer**, depending on your chosen organism. You are asked if you want to open *eqtlexplorer_mm5.jnlp* or save it to disk.

   Note: You must have Sun Microsystem's JRE version 1.4.2 or greater installed on your computer, and the Java Webstart plug-in must be properly configured for eQTL Explorer to open.

3. Click **OK** to open eQTL Explorer. You may have to accept a digital certificate. The Log On window displays.
4. Enter the User name **guest** and leave the password field blank.
5. Click **Connect**. eQTL Explorer opens. The PhenoGen website remains open in the background.
6. Close eQTL Explorer when you are finished.

Performing Homolog Search

Homolog Search Overview

Homologous genes demonstrate high sequence similarity and can demonstrate similarity in functions. Homologous sequences (genes) can be divided into two groups: Orthologs and Paralogs. Homologous sequences (genes) in two different species originating from a common ancestor are known as Orthologs. Duplication of a homologous sequence in a given species results in Paralogous sequences with a different chromosomal location. Using the Homolog Search tool in the PhenoGen website, users can obtain information regarding chromosomal locations, for genes in a given gene list, in other genomes. For example, for a list of differentially genes between two selected lines of mice, users can obtain the chromosomal location for these genes in the mouse genome as well as the chromosomal location for the known homologous genes in the rat and human genomes.

Performing Homolog Search

Homolog Search allows you to obtain information regarding chromosomal locations, for genes in a given gene list, in other genomes. Homolog Search can be accessed in two ways:

- Select **Gene Lists menu > Homolog Search**.
- Select **Gene Lists menu > View All Gene Lists**, choose a gene list, select **Homolog Search** from the Gene List Tools drop-down, and click **Go**.

On the *Homologous Genes* page:

1. Choose a gene list from the drop-down list.
2. Click **Go**. The *Homologous Genes* page displays. The table on the page displays:
    - Gene Identifier
    - HomoloGene ID (NCBI)
    - Gene Symbol
    - Homolog Species
    - Homolog Identifier
    - Homolog Chromosome Location
3. Click on **HomoloGene ID** or **Homolog Species** and **Identifier** to open the website for that selection.
4. Click on **Download** to download the information.

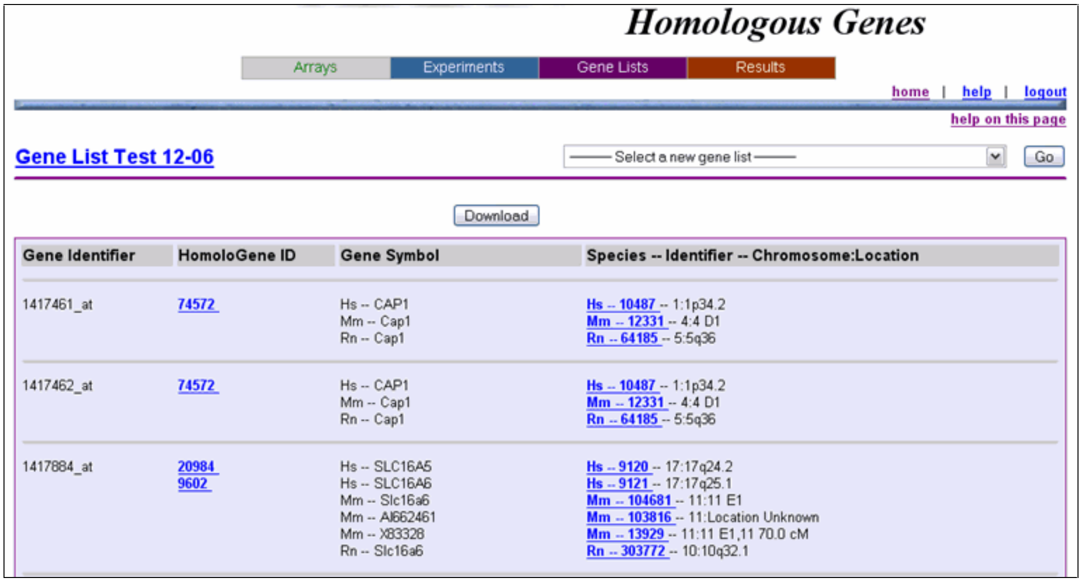

Gene Identifier	HomoloGene ID	Gene Symbol	Species -- Identifier -- Chromosome:Location
1417461_at	74572	Hs -- CAP1<br>Mm -- Cap1<br>Rn -- Cap1	Hs -- 10487 -- 1:1p34.2<br>Mm -- 12331 -- 4:4 D1<br>Rn -- 64185 -- 5:5q36
1417462_at	74572	Hs -- CAP1<br>Mm -- Cap1<br>Rn -- Cap1	Hs -- 10487 -- 1:1p34.2<br>Mm -- 12331 -- 4:4 D1<br>Rn -- 64185 -- 5:5q36
1417884_at	20984<br>9602	Hs -- SLC16A5<br>Hs -- SLC16A6<br>Mm -- Slc16a6<br>Mm -- AI662461<br>Mm -- X83328<br>Rn -- Slc16a6	Hs -- 9120 -- 17:17q24.2<br>Hs -- 9121 -- 17:17q25.1<br>Mm -- 104681 -- 11:11 E1<br>Mm -- 103816 -- 11:Location Unknown<br>Mm -- 13929 -- 11:11 E1,11 70.0 cM<br>Rn -- 303772 -- 10:10q32.1

Using Promoter Options

Promoter Analysis - oPOSSUM Overview

Note: This is a customized version of oPOSSUM featuring a set of input parameters.

oPOSSUM is a tool for determining the over-representation of transcription factor binding sites (TFBS) within a set of (co-expressed) genes as compared with a pre-compiled background set (Ho Sui et al., 2005, Nucleic Acids Res, 33(10):3154-64). The input is a set of gene identifiers and analysis parameters. The system compares the number of hits for each selected TFBS on the target gene set against the background set. Two different measures of statistical significance are applied to determine which TFBS sites are over-represented in the target set. The results of the analysis are displayed in a tabular form.

Selection Criteria

Selection of transcription factor binding site matrices

All matrices in the oPOSSUM database with a given minimum specificity are selected. These matrices are obtained from the JASPAR database.

Selection of TFBS analysis parameters

Search Regional Level

This refers to the size of the region around the transcription start site (TSS) which was analyzed for TFBS sites. The background set was computed using a region extending a maximum of 5000 bp upstream and 5000 bp downstream of the TSS. During the background computation the upstream region was truncated to less than 5000 bp if it overlapped an upstream exon from another gene.

Conservation Level

In order to limit spurious TFBS sites, conservation with the aligned orthologous mouse sequence was used as a filter such that only sites which fell within these non-coding conserved regions were kept. A conserved region was defined as a span of some minimum length L within the human sequence which had a percent identity with the aligned mouse sequence of some minimum value X. The background set was pre-computed with three levels of conservation filter. Level 1 corresponds to the top 10 percentile of non-coding conserved regions with an absolute minimum percent identity of 70%. Level 2 corresponds to the top 20 percentile with a minimum percent identity of 65% and level 3 corresponds to the top 30 percentile with a minimum percent identity of 60%.

Matrix Match Threshold

TFBS sites are scanned by sliding the corresponding position weight matrix (PWM) along the sequence and scoring it at each position. The threshold is the minimum relative score used to report the position as a putative binding site. The background set was computed using a threshold of 70%.

Statistical measure for over-representation

Two measures of statistical over-representation are available: a one-tailed Fisher exact probability and a Z-score.

One-tailed Fisher Exact Probability

The one-tailed Fisher exact probability compares the proportion of co-expressed genes containing a particular TFBS to the proportion of the background set that contains the site to determine the probability of a non-random association between the co-expressed gene set and the TFBS of interest. It is calculated using the hypergeometric probability distribution that describes sampling without replacement from a finite population consisting of two types of elements. Therefore, the number of times a TFBS occurs in the promoter of an individual gene is disregarded, and instead, the TFBS is considered as either present or absent.

Z-score

The Z-score uses a simple binomial distribution model to compare the rate of occurrence of a TFBS in the target set of genes to the expected rate estimated from the pre-computed background set.

For a given TFBS, let the random variable x denote the number of predicted binding site nucleotides in the conserved non-coding regions of the target gene set. Let B be the number of predicted binding site nucleotides in the conserved non-coding regions of the background gene set. Using a binomial model with n events, where n is the total number of nucleotides examined (i.e. the total number of nucleotides in the conserved non-coding regions) from the co-expressed genes, and N is the total number of nucleotides examined from the background genes, then the expected value of x is u = B * C, where C = n / N (i.e. C is the ratio of sample sizes). Then taking p = B / N as the probability of success, the standard deviation is given by s = sqrt(n * p * (1 - p)).

Let *x* be the observed number of binding site nucleotides in the conserved non-coding regions of the co-expressed genes. By applying the Central Limit Theorem and using the normal approximation to the binomial distribution with a continuity correction, the z-score is calculated as $z = (x - u - 0.5) / s$. Then, the probability of observing *x* or more binding site nucleotides in the conserved non-coding regions of the target genes, given the TFBS is not truly over-represented in the target genes, is the p-value associated with $Pr(Z >= z)$.

Running oPOSSUM

The *PromoterAnalysis - oPOSSUM* page can be accessed in two ways:

- Select **Gene Lists menu > Promoter > Promoter Analysis - oPOSSUM**.
- Select **Gene Lists menu > View All Gene Lists**, choose a gene list, select **Promoter Analysis - oPOSSUM** from the Gene List Tools drop-down, and click **Go**.

On the *Promoter Analysis - oPOSSUM* page:

1. Select a gene list from the drop-down list.
2. Click **Go**. The table displays genes from the selected list in the **Gene List** field and default parameters.

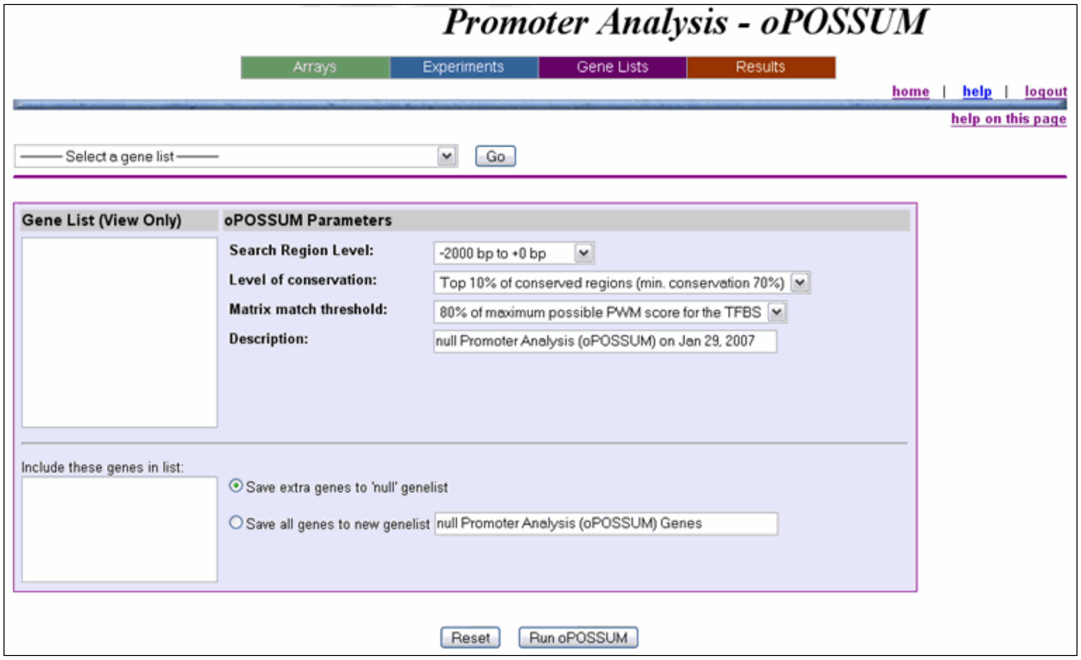

3. Change the parameters as necessary, using the drop-down lists.

   - Set the **Search Region Level**.
   - Set the **Level of Conservation**.
   - Set the **Matrix Match Threshold**.

4. Change the **Description**, if appropriate.
5. Use the **Include these genes in list** field to enter additional genes that are not part of the current list.
6. Select **Save extra genes to "current" gene list** or **Save all genes to new gene list**. If you choose to save all genes to a new list, enter the new gene list name.
7. Click **Run oPOSSUM**. Running oPOSSUM takes time. When your results are available, an email is sent to the address you provided in the Registration page to inform you that you can view those results.

Promoter Analysis - MEME Overview

The oPOSSUM search is based on occurrences of known motifs (transcription factor binding sites). There are many software options available to explore the occurrence of previously uncharacterized motifs. Although these have not been directly incorporated within the PhenoGen website as with oPOSSUM, they can easily be applied using other publicly available web servers. A recent comprehensive review (Tompa et al., 2005, Nature Biotechnology, 23:137) of such softwares found that MEME (Bailey and Elkan, 1995, Proc. Int Conf Intell Syst Mol Biol, 3:21), which stands for Multiple EM for Motif Extraction, was one of the best performing algorithms on mouse data. Methods like MEME are optimal for analyzing sequences less than 2KB and it is not recommended to use longer lengths for such tools. Furthermore, many motif software webservers restrict the input data size. MEME is available at PhenoGen and can be accessed by clicking on *Promoter* under the *Gene Lists* drop-down menu. There are a number of options users need to select prior to submitting the search to MEME. MEME can also be accessed at http://meme.sdsc.edu/meme/meme.html.

Running MEME

MEME (Multiple EM for Motif Extraction) allows you to discover motifs in groups of related DNA or protein sequences. MEME can be accessed in two ways:

- Select **Gene Lists menu > Promoter > Promoter Analysis - MEME**.
- Select **Gene Lists menu > View All Gene Lists**, choose a gene list, select **Promoter Analysis - MEME**.

On the *Promoter Analysis - MEME* page:

1. Select a gene list from the drop-down list.
2. Click **Go**. The table displays genes from the selected list in the *Gene List* field and default parameters.

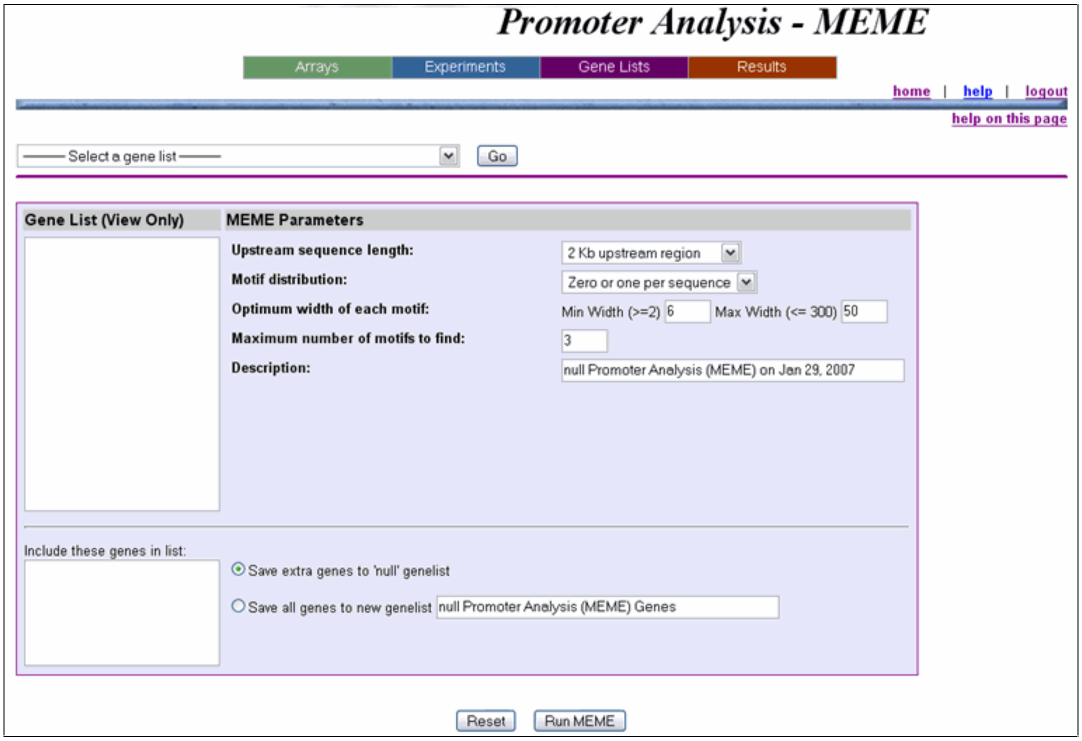

3. Select the **Upstream sequence length**.
4. Choose the **Motif distribution**.
5. Specify the **Minimum Width** and **Maximum Width**.
6. Specify the **Maximum number of motifs to find**.
7. Change the **Description**, if appropriate.
8. Use the **Include these genes in list** field to enter additional genes that are not part of the current list.
9. Select **Save extra genes to 'current gene list'** or **Save all gene to new gene list**. If you choose to save all genes to a new list, enter the new gene list name.
10. Click **Run MEME**. MEME will take some time to complete. When your results are available, an email is sent to the address provided in the Registration page to inform you that the results are ready for viewing.

Upstream Sequence Extraction Overview

An important step in understanding the mechanisms that regulate the expression of genes is represented by the ability to identify regulatory elements, i.e., the binding sites in DNA for transcription factors. Transcription factors are DNA binding proteins, typically upstream from, and close to, the transcription start site (TSS) of a gene, that modulates the expression of the gene by activating or repressing the transcription machinery.

Because there is a limited amount of information regarding the majority of the transcription factors and especially about their target binding sites (even in well-characterized organisms) you could focus on computational tools designed for the discovery of novel regulatory elements, where nothing is known a priori of the transcription factor or its preferred binding sites. If you provide a collection of sequences that correspond to the regulatory regions of genes that are believed to be coregulated, the computational tool identifies short DNA sequence 'motifs' that are statistically over- or under-represented in these regulatory regions. Accurate identification of these motifs is very difficult because they are short signals (typically about 10 bp long) in the midst of a great amount of statistical noise (a typical input being one regulatory region of length 1,000 bp upstream of each gene). Also, there is marked sequence variability among the consensus binding sites of a given transcription factor, and the nature of the variability itself is not well understood.

There are numerous tools available for this task of motif prediction. They differ from each other mainly in their definition of what represents a motif and what would be an acceptable model for statistical over-representation of a motif. A comprehensive list of tools that could be used (table adapted from Tompa et al., Nature Vol. 23, No.1, Jan 2005, p137-144) is presented. This sequence information can be used to carry out TFBS analysis, off the PhenoGen website, using any of these tools. See the topic *Promoter Extraction Tools* in the *Supplementary Information* section.

Running Upstream Sequence Extraction

The upstream sequence extraction tool is used to extract the upstream genome sequence of a particular gene. The *Upstream Sequence Extraction* page can be accessed in two ways:

- Select **Gene Lists menu > Promoter > Promoter Analysis**.
- Select **Gene Lists menu > View All Gene Lists**, choose a gene list, select **Promoter Analysis** from the Gene List Tools drop-down, and click **Go**.

On the *Upstream Sequence Extraction* page:

1. Select a gene list from the drop-down list.
2. Click **Go**. The table displays genes from the selected list in the **Gene List** field and default extraction parameters.

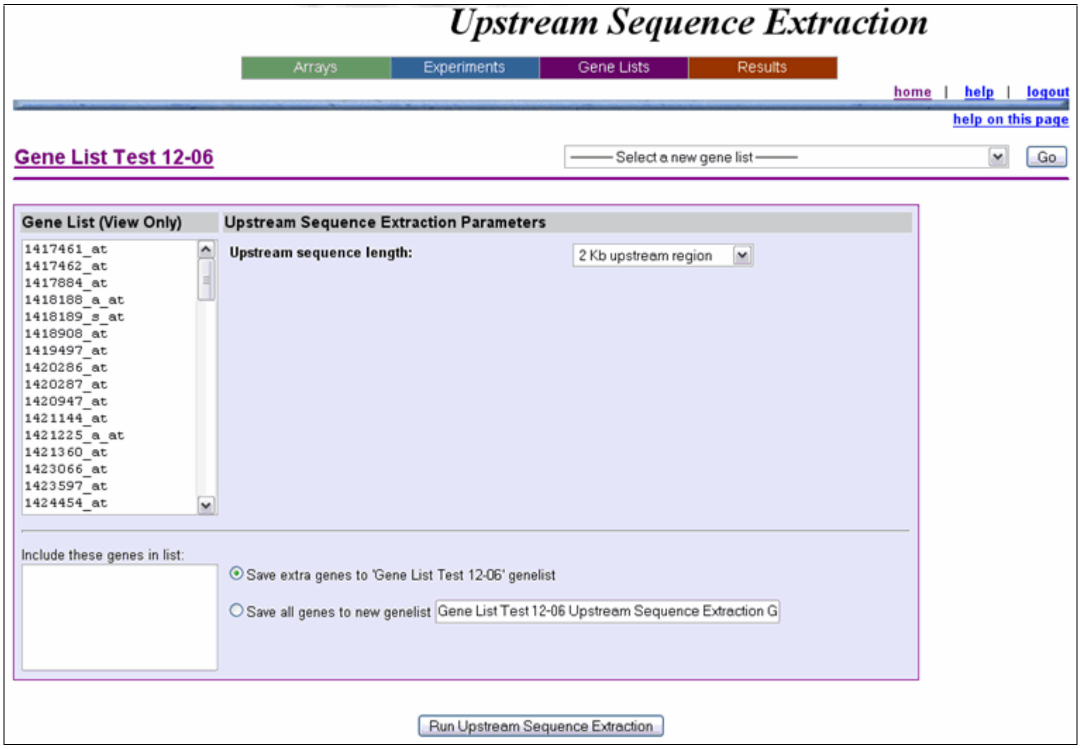

3. Select the **Upstream sequence length**.
4. Use the **Include these genes in list** field to enter additional genes that are not part of the current list.
5. Select **Save extra genes to "current" gene list** or **Save all genes to new gene list**. If you choose to save all genes to a new list, enter the new gene list name.
6. Click **Run Upstream Sequence Extraction**. The upstream sequence extraction will take some time. An email will be sent to the address you provided in the registration page once it is finished and ready for viewing.

Genetic Variations Overview

Users can access genetic variations using *TranscriptSNPView* at Ensembl to identify coding variations for the genes of interest. Data for genetic variations is available for mice, rats, and human. *TranscriptSNPView* uses genetic variation information obtained from dbSNP, the National Institute of Environmental Health Sciences (NIEHS), and the Sanger Institute. A help menu describing the details of the information provided and various options available to obtain genetic variations is available at Ensemble. Users have several options, including selecting the source of the data (Sanger and/or dbSNP), selecting the form of variation (deletion/insertion polymorphism, SNP, etc.), selecting the type of SNP (synonymous or non-synonymous SNPs, SNPs in the upstream region, SNPs in regulatory regions, SNPs in 5' UTR, etc.), selecting inbred strains to display, and selecting the length of the sequence around exons and UTRs to be displayed. Users may also choose to export or download the data. Genetic Variation Data Retrieval can be accessed either from the Gene Lists menu or from Basic and Advanced Annotation.

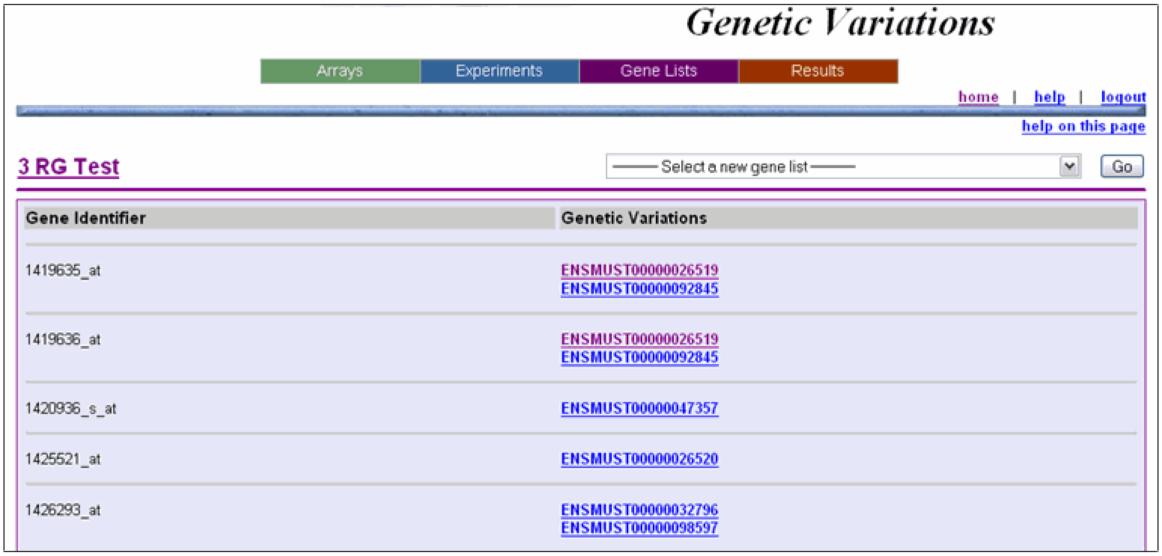

Retrieving Genetic Variation Data

The *Genetic Variations* page can be accessed in three ways:

- Select **Gene Lists menu** > **Annotation** > **Basic Annotation**
- Select **Gene Lists menu** > **Annotation** > **Advanced Annotation**
- Select **Gene Lists menu** > **Promoter** > **Genetic Variations**

Genetic Variations is included in the Basic Annotation table and is an option in Advanced Annotation. To use the third option:

1. Select a gene list from the drop-down menu.
2. Click on **Go**.
3. The screen then displays the chosen gene list name with an option to 'select a new gene list'.
4. Once confirmed, click **Get Data**.

The Genetic Variations page now displays. The Gene Identifier is displayed along with the Genetic Variations, which are hyperlinked.

Performing Literature Search

Literature Search Overview

The Literature Search option is an automated literature search that you can tailor to your area(s) of interest by selecting a set of query terms. Query terms can be further organized into categories. The automated literature search then looks for articles that contain the gene symbol, plus one or more of the query terms. The results of the search are organized by the user-defined categories and by gene name. The return page provides the title, abstract, and PubMed link for each of the documents. The gene identifiers and keywords from the search are also highlighted, in order to help you sort, read, and work through what is likely to be a large amount of text.

Co-reference Analysis

An additional feature of the literature search on the PhenoGen website is that publications are flagged if more than one of the genes within the gene list are mentioned in a single article. This allows the user to easily identify genes that have previously shown a documented relationship.

Performing a Literature Search

The Literature Search on the PhenoGen website allows you to perform a Literature Search for a gene list as well as the co-references for each gene in the list. See the topic *Viewing Literature Search Results* for more details.

The *Literature Search* page can be accessed in two ways:

- Select **Gene Lists menu > Literature Search**.
- Select **Gene Lists menu > View All Gene Lists**, choose a gene list, select **Literature Search** from the Gene List Tools drop-down, and click **Go**.

On the *Literature Search* page:

1. Choose a gene list from the drop-down list.
2. Click **Go**. The table displays genes from the selected list in the **Gene List** field.

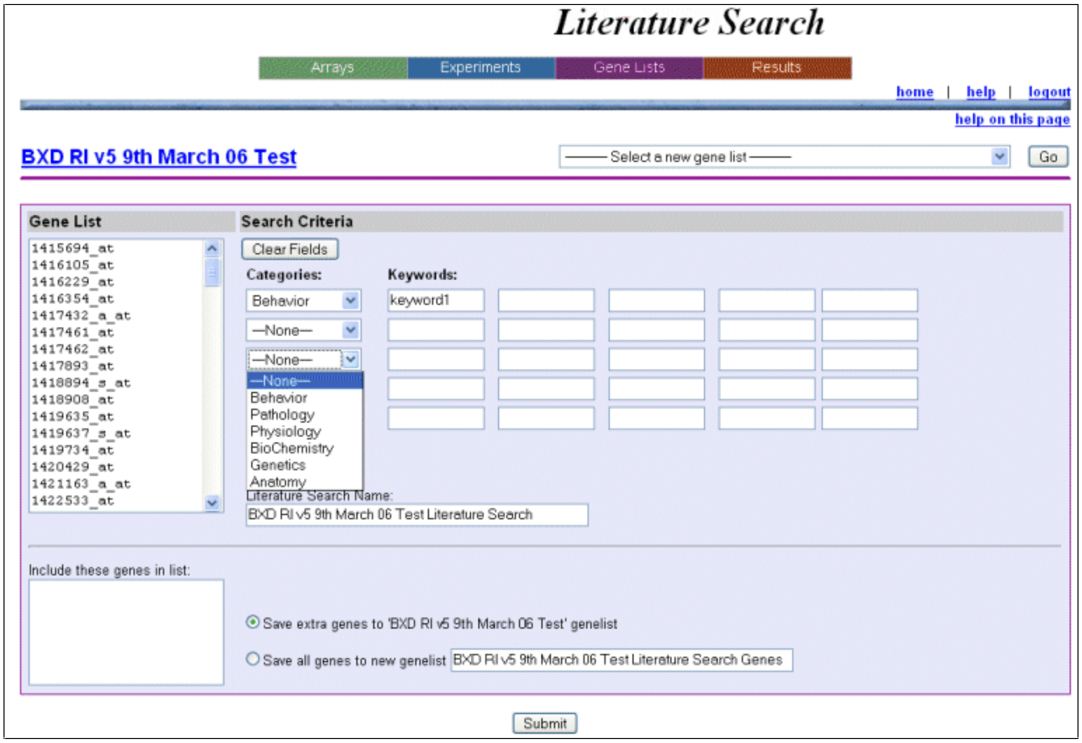

3. Select a value from the drop-down list in the **Category** field. The category field provides you with a way to categorize your keywords.

Note: The Category name is NOT used in the search, but is a *REQUIRED* field. ONLY values in the keyword fields are used in the search.

4. Enter a **keyword** or keywords for the category you selected.
5. *OPTIONAL* **-** Click **Clear Fields** to clear all the categories and keywords fields.
6. Change the **Literature Search Name**, if appropriate.
7. Use the **Include these genes in list** field to enter additional genes that are not part of the current list.
8. Select **Save extra genes to "current" gene list** or **Save all genes to new gene list**. If you choose to save all genes to a new list, enter the new gene list name.
9. Click **Submit**. Literature searches take time. When the search is complete, an email is sent to the address you provided in the *Registration* page to inform you that you can view your literature search results.

Viewing Interpretation Results by Gene List

Interpretation results, such as oPOSSUM results, MEME results, upstream sequence extraction results, and literature searches, can be viewed for a selected gene list.

1. Select **Gene Lists menu > View Interpretation Results.**
2. Select a gene list from the drop-down menu.
3. Click **Go**. The *Results* page displays.

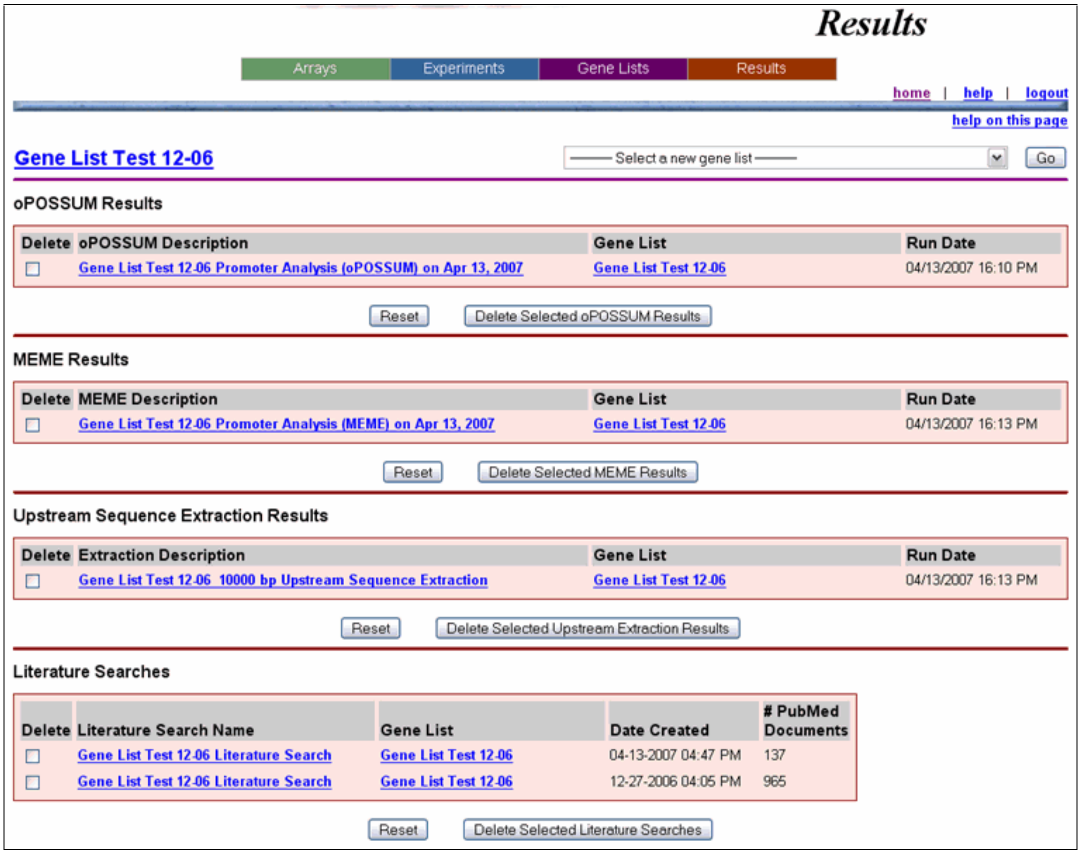

You can:

- Click on the **Results Description** or **Literature Search Name** to view the results.
- Click the gene list in the **Gene List** column to view the *Gene List Details* page for that gene list.
- Select the **Delete** checkbox next to a result and the **Delete** button for that result to delete an existing result.

Comparing Gene Lists

The *Compare Gene Lists* page allows you to compare two gene lists, side-by-side.

1. Select **Gene List menu > Compare Gene Lists**. The *Compare Gene Lists* page displays.
2. Select a gene list from each of the drop-down lists.
3. Click **Select Gene Lists**. The gene lists display.

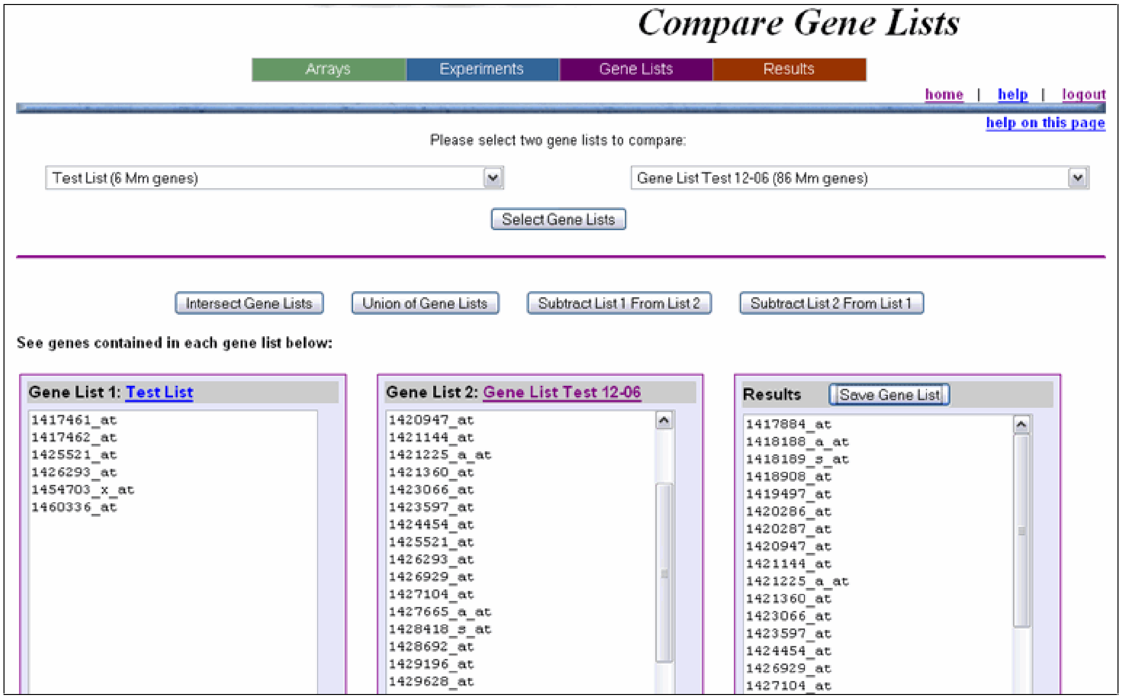

4. Do one of the following:

- Click Intersect Gene Lists to view a list that displays the genes that are in both selected lists.
- Click Union of Gene Lists to combine the two gene lists into one gene list
- Click Subtract List 1 from List 2 to remove the genes in list 1 from list 2. The resultant gene list displays the genes that are only in list 2.
- Click Subtract List 2 from List 1 to remove the genes in list 2 from list 1. The resultant gene list displays the genes that are only in list 1.

5. Click **Back to Gene List Comparison** to return to the *Compare Gene Lists* page.
6. Select another option from step 4, if you choose.

Uploading a Gene List

You can upload an existing gene list to the PhenoGen website for analysis. Your gene list must be a text file and contain only one gene identifier per line or it will not upload correctly.

The *Upload Gene List* page can be accessed in two ways:

- Select **Gene Lists menu > Upload Gene List**.
- Select **Gene Lists menu > Create Gene List > Upload Gene List**.

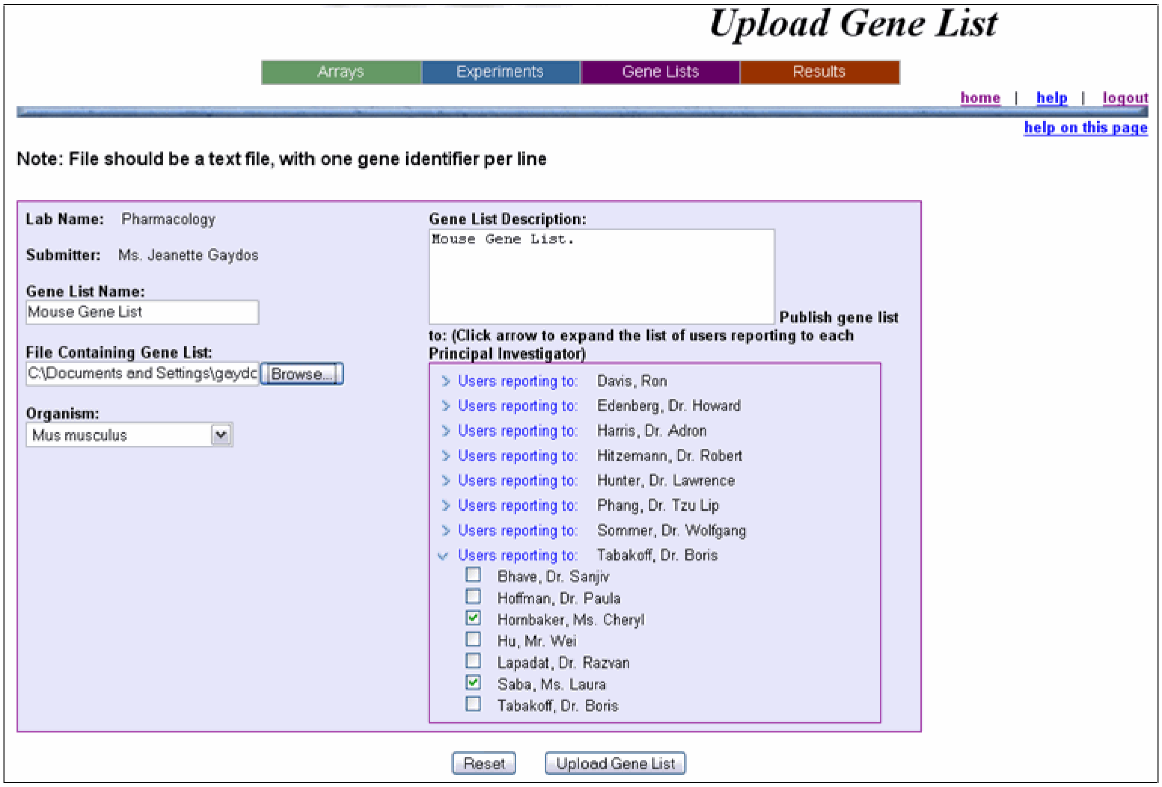

On the *Upload Gene List* page:

1. Enter the Gene List Name.
2. Enter the **Gene List Description**.
3. Click **Browse** and follow the instructions to select the gene list you want to upload.
4. Select the **Organism** from the drop-down list to define the organism for your gene list.
5. *OPTIONAL* – Click the checkbox beside each user who you want to view the gene list in the **Publish gene list to these users** section.
6. Click **Upload Gene List**.

Downloading a Gene List

You can download a gene list that you create using the PhenoGen website to analyze it using your own tools, or to distribute it to other users. The *Download Gene List* page can be accessed in two ways:

- Select **Gene Lists menu > Download Gene List**.
- Select **Gene Lists menu > View All Gene Lists**, choose a gene list, select **Download** from the Gene List Tools drop-down, and click **Go**.

On the *Download Gene List* page:

1. Select a gene list from the drop-down list.
2. Click **Go**. The gene list displays.

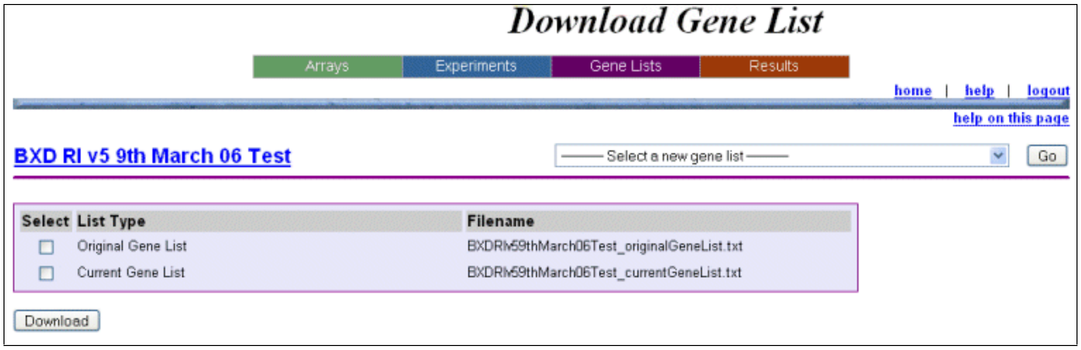

3. Click the checkbox next to the gene list you want to download (original or current). You can choose both, if appropriate.
4. Click **Download**. You have the option to open or save the files.
5. Follow the instructions that display as you open or save the files. These instructions vary, depending on your Internet browser (e.g., Internet Explorer, Firefox, etc.).

Cloning and Mutating a Gene List

The *Clone and Mutate Gene List* page allows you to create a new gene list from an existing gene list by copying the original gene list. The *Clone and Mutate Gene List* page can be accessed in three ways:

- Select **Gene Lists menu > Clone and Mutate**.
- Select **Gene Lists menu > Create Gene List > From Existing Gene List**.
- Select **Gene Lists menu > View All Gene Lists**, choose a gene list, select **Clone and Mutate** from the Gene List Tools drop-down, and click **Go**.

On the *Clone and Mutate Gene List* page:

1. Choose the gene list that you want to copy.
2. Click **Go**. The original gene list populates the page. The Gene List Name field is populated with the original gene list name and "Copy" appended after the name. For example, a gene list named *Test* becomes *Test Copy* when you copy it.

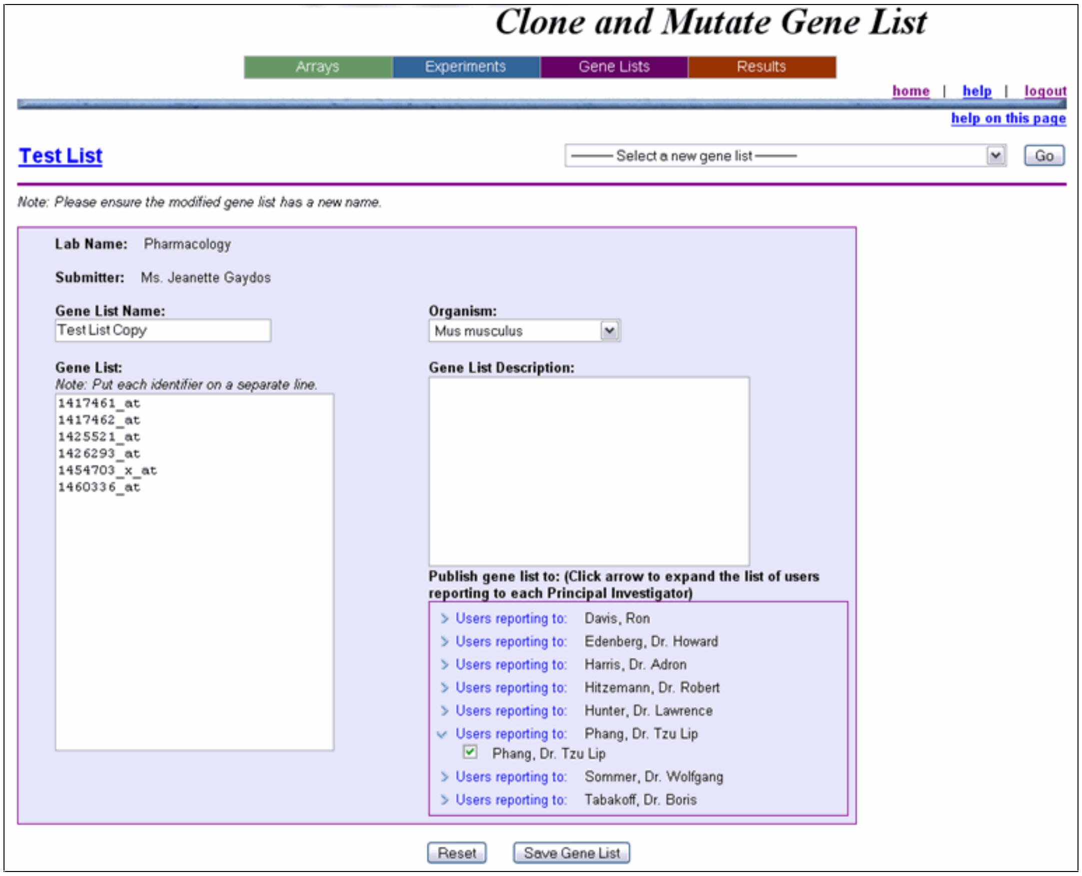

3. Change the **Gene List Name** for the new gene list, as necessary.
4. Change the **Organism**, if appropriate.
5. Revise the **Gene List Description**, if appropriate.

6. *OPTIONAL* - Type additional genes into the **Gene List** field. Separate the genes with a space or press the **Enter** key on your keyboard between entries or the entries will not be processed correctly. Genes can be entered in the following formats:

Gene IDs	Example	Description
Affymetrix ID	1416283_at	
CodeLink ID	NM_009775_Probe1<br>GE34729	
Ensembl ID	ENSMUSG00000022962	Begins with ENS, followed by the three-letter organism, followed by G, followed by a number.
Entrez Gene ID	14450	Contains three or more numbers.
FlyBase Gene ID	FBgn0013277	All IDs are preceded by FBgn and have numbers following.
Gene Symbol	Grin1	Official gene symbols from MGI.
MGI ID	MGI:95654	
NCBI/EMBL RNA accession number	AK146355	Begins with one or two letters, followed by multiple numbers.
RefSeq RNA accession number	NM_010256	Begins with two capital letters, followed by an underscore, followed by numbers.
RGD ID	2203	Contains four or more numbers.
UniGene ID	Mm.4505	Contains an organism prefix (Dm, Hs, Rn, or Mm), followed by a period, followed by numbers.

7. *OPTIONAL* - Highlight genes in the **Gene List** field, then press the **Delete** button on your keyboard to remove the highlighted genes from the list.
8. *OPTIONAL* - Click the checkbox beside each user who you want to view the gene list in the **Publish gene list to these users** section.
9. Click **Save Gene List**. The gene list is copied and saved with the new name and new or revised data. Click **Reset** to return all the fields to their original values.

Note: You can view the gene list on the *View All Gene Lists* page.

Deleting a Gene List

If you no longer need a gene list, you can delete it.

Important! Any references to the gene list, such as a literature searches, are deleted when the gene list is deleted.

The *Delete Gene List* page can be accessed in two ways:

- Select **Gene Lists menu > Delete Gene List**.
- Select **Gene Lists menu > View All Gene Lists**, choose a gene list, select **Delete** from the Gene List Tools drop-down, and click **Go**.

On the *Delete Gene List* page:

1. Select the gene list from the drop-down list that you want to delete.

   Note: You can only delete gene lists that you own.

2. Click **Go**. A link to the gene list displays.
3. *OPTIONAL* - Click the gene list link to view details for the gene list.

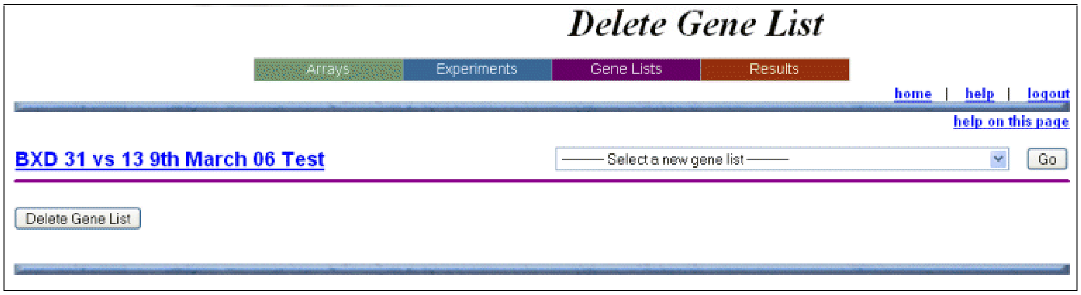

4. Click **Delete Gene List**. A pop-up dialogue asks you to confirm the delete.
5. Click **OK** to delete your selection. Click **Cancel** to return to the *Delete Gene List* page.

Viewing User Access to a Gene List

You can view the users who have access to your gene lists. You can grant users access to a gene list that you created from an experiment you own. You cannot publish a gene list that you do not own. The Gene List Users page can be accessed in two ways:

- Select **Gene Lists menu** > **View User Access**.
- Select **Gene Lists menu** > **View All Gene Lists**, choose a gene list, select **User Access** from the Gene List Tools drop-down, and click **Go**.

On the *Gene List Users* page:

1. Select a gene list from the drop-down list.
2. Click **Go**. A list of users who can view the selected gene list displays.

   Note: If you are the owner of the gene list, you can allow other users to view the gene list. If you are not the owner, the **Check All**, **Uncheck All**, **Reset**, and **Update Gene List Users** buttons do not display.

   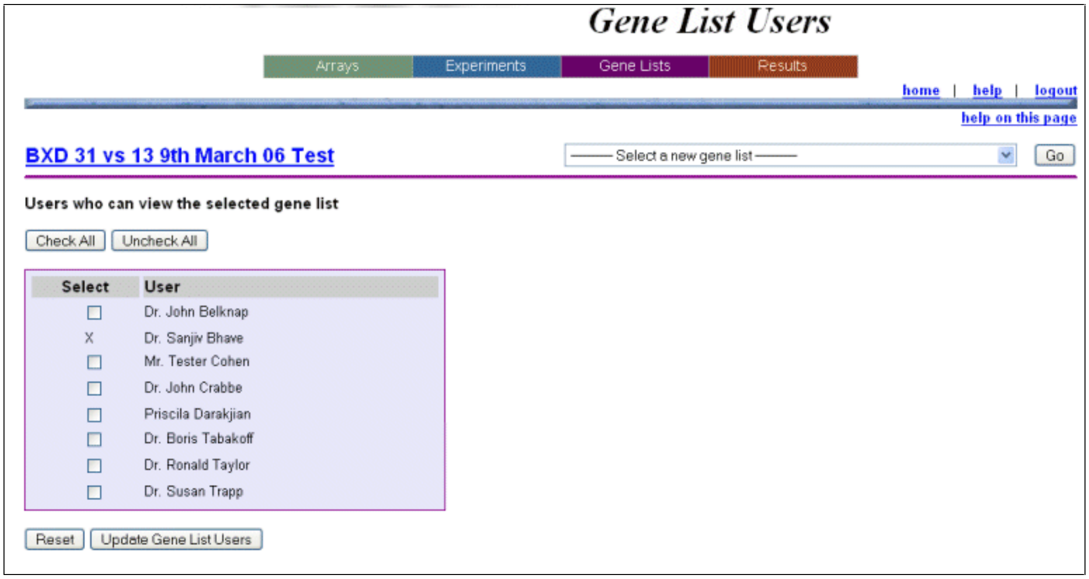

3. Select the users to whom you want to grant view permissions for your gene list.
4. Click **Update Gene List Users**.

Results

Viewing Interpretation Results Overview

You can view interpretation results for quality control checks that you have performed on experiment, promoter analysis, and literature searches that you have performed on specific gene lists. See the topics *Running a Quality Control Check*, *Running oPOSSUM*, *Running MEME*, *Running Upstream Sequence Extraction*, and *Performing a Literature Search* for details.

The *Quality Control Results* page displays the results for all quality control checks you have performed.

The *oPOSSUM Results*, *MEME Results*, *Upstream Sequence Extraction Results*, and *Literature Search Results* pages display results for all the searches or analyses you have performed. You can also delete these searches and results.

Results Menu

The *Results* menu displays when you move your mouse over the **Results** button.

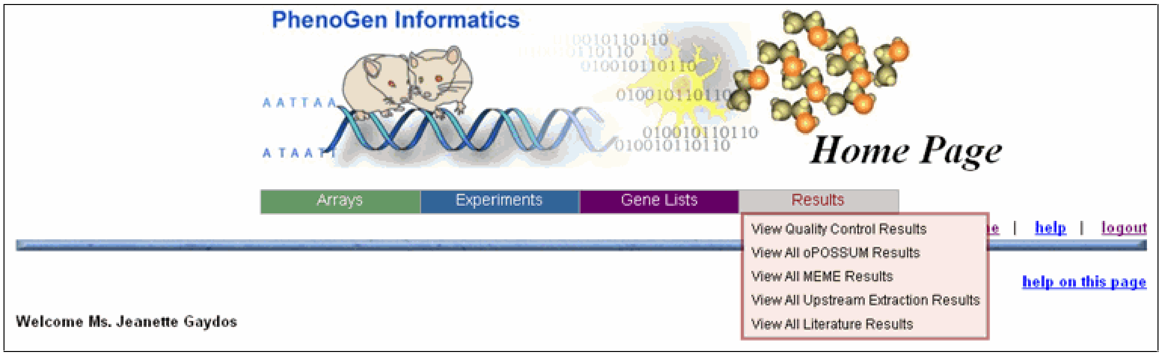

Drop-down Menu Option	Description
View Quality Control Results	Allows you to view the quality control results for a selected experiment.
View All oPOSSUM Results	Allows you to view all the oPOSSUM results for a selected gene list.
View All MEME Results	Allows you to view the MEME results for a selected gene list.
View All Upstream Extraction Results	Allows you to view the upstream genome sequences for a selected gene list.
View All Literature Results	Allows you to view all interpretation results for literature analysis.

Viewing Quality Control Results

The *Quality Control Results* page displays the results of the quality control checks you have run. You can click the sample name in any of the tables to view MIAMExpress experiment details for that sample.

1. Select **Results menu** > **View Quality Control Results**. The *Quality Control Results* page displays.
2. Select an experiment from the drop-down list.
3. Click **Go**. The Quality Control Results for that experiment display.

Quality Control Results

Each quality control step has its own section in the results page. See the topics *MIAMExpress Data Comparison (Step 1)*, and *Array Integrity (Step 2)* for details about each step.

Step and Results
Step 1, MIAMExpress Data Comparison, ensures that the attributes entered for each array are compatible. If warnings are generated, attributes where more than one value was found are shown in yellow. You have the option to delete arrays, if necessary. See the *Quality Control Results Example*.
Step 2, Array Integrity, displays information to help you determine the integrity of the arrays in your experiment. It shows graphs for QC Stats, Relative Log Expressions, and Normalized Unscaled Standard Errors.<br><br>The QC results may or may not have images, depending on the option chosen when running *Quality Control Checks*. If images are generated, each array provides you with the option to click the **[View Images]** and **[View MAplot]** links to review residual images and MAplot images of the array in a separate window. You have the option to delete arrays from the results pages, if necessary. See the topic *Compare Arrays Within an Experiment* in the *Array Integrity* section for details about **[View Images]**. See the topic *MA Plots* in the *Graphical Representation of Normalized Data* section for details about **[View MAplot]**.

Deleting an Array During Quality Control

1. Select the checkbox beside any array(s) you want to delete.
2. Click **Delete Array** at the bottom of the page to remove the selected arrays.

Quality Control Results Affymetrix Example

The following two images show all the details that display on the *Quality Control Results* page.

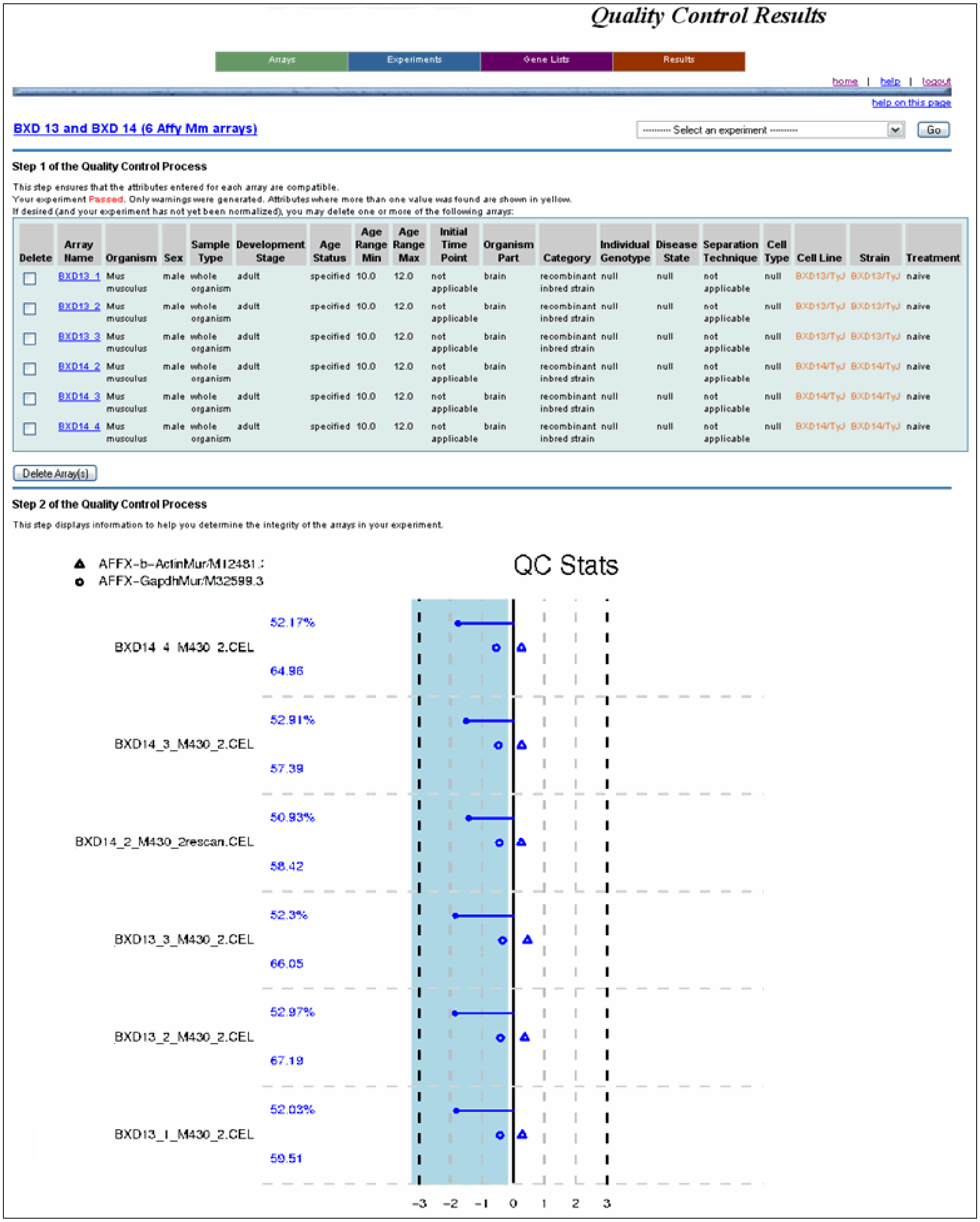

Quality Control Results

Arrays | Experiments | Gene Lists | Results

home | help | logout

help on this page

BXD 13 and BXD 14 (6 Affy Mm arrays)

------- Select an experiment ------- Go

Step 1 of the Quality Control Process

This step ensures that the attributes entered for each array are compatible.
Your experiment Passed. Only warnings were generated. Attributes where more than one value was found are shown in yellow.
If desired (and your experiment has not yet been normalized), you may delete one or more of the following arrays:

Delete	Array Name	Organism	Sex	Sample Type	Development Stage	Age Status	Age Range Min	Age Range Max	Initial Time Point	Organism Part	Category	Individual Genotype	Disease State	Separation Technique	Cell Type	Cell Line	Strain	Treatment
☐	BXD13_1	Mus musculus	male	whole organism	adult	specified	10.0	12.0	not applicable	brain	recombinant inbred strain	null	null	not applicable	null	BXD13/TyJ	BXD13/TyJ	naive
☐	BXD13_2	Mus musculus	male	whole organism	adult	specified	10.0	12.0	not applicable	brain	recombinant inbred strain	null	null	not applicable	null	BXD13/TyJ	BXD13/TyJ	naive
☐	BXD13_3	Mus musculus	male	whole organism	adult	specified	10.0	12.0	not applicable	brain	recombinant inbred strain	null	null	not applicable	null	BXD13/TyJ	BXD13/TyJ	naive
☐	BXD14_2	Mus musculus	male	whole organism	adult	specified	10.0	12.0	not applicable	brain	recombinant inbred strain	null	null	not applicable	null	BXD14/TyJ	BXD14/TyJ	naive
☐	BXD14_3	Mus musculus	male	whole organism	adult	specified	10.0	12.0	not applicable	brain	recombinant inbred strain	null	null	not applicable	null	BXD14/TyJ	BXD14/TyJ	naive
☐	BXD14_4	Mus musculus	male	whole organism	adult	specified	10.0	12.0	not applicable	brain	recombinant inbred strain	null	null	not applicable	null	BXD14/TyJ	BXD14/TyJ	naive

Delete Array(s)

Step 2 of the Quality Control Process

This step displays information to help you determine the integrity of the arrays in your experiment.

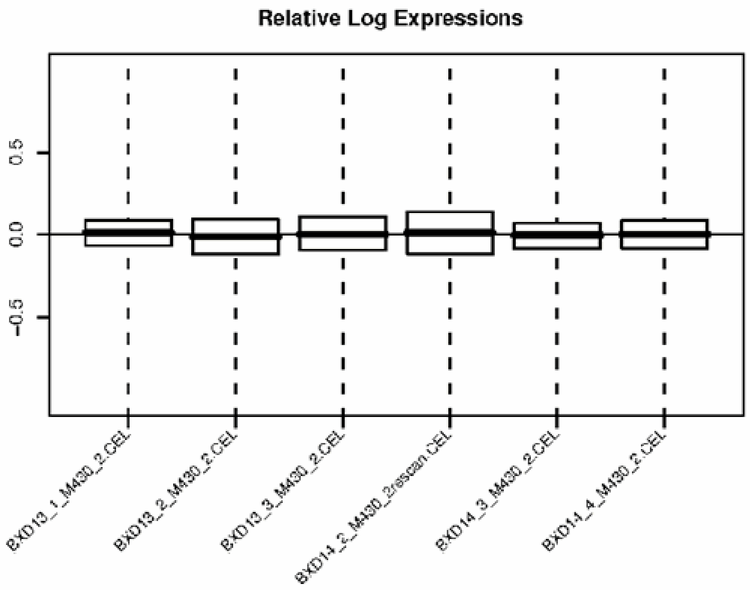

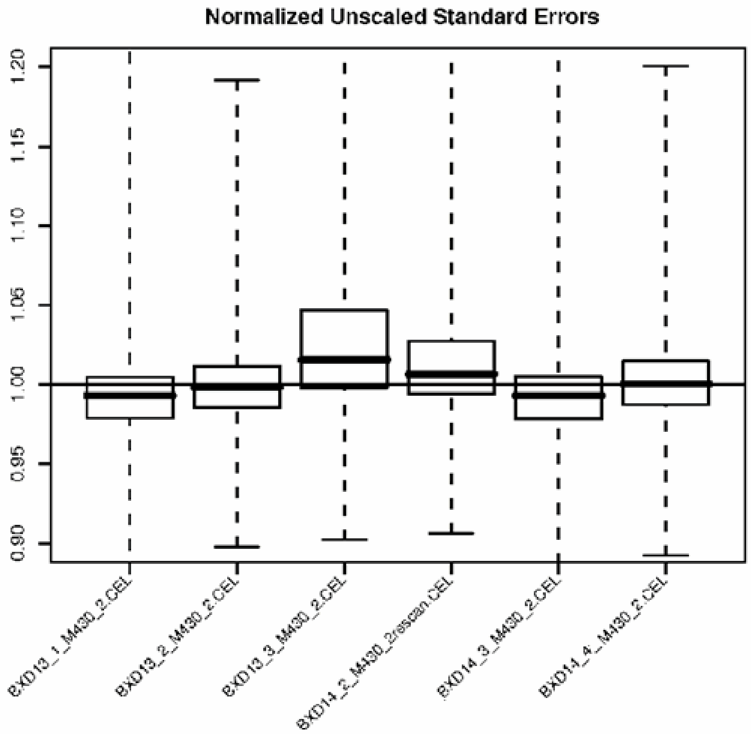

Step 3 of the Quality Control Process

This step displays the pseudo-images and MA plots generated as part of the quality control process.

If you generated images during the quality control process, you may click on the [View ...] links to see images of the array in a separate window. It may take a few minutes to display these images, so please be patient. If desired, after viewing the images, you may delete one or more of the following arrays:

Delete	Array Name	Image Links		MA Plot Statistics	
				Median	IQR
☐	BXD13_1	[View Images]	[View MA Plot]	0.00280863	0.2476
☐	BXD13_2	[View Images]	[View MA Plot]	-0.0183394	0.2768
☐	BXD13_3	[View Images]	[View MA Plot]	-0.0138910	0.3157
☐	BXD14_2	[View Images]	[View MA Plot]	0.0213354	0.3188
☐	BXD14_3	[View Images]	[View MA Plot]	0.000264144	0.2503
☐	BXD14_4	[View Images]	[View MA Plot]	0.00823552	0.2643

Delete Array(s)

Quality Control Results CodeLink Example

The following two images show all the details that display on the *Quality Control Results* page.

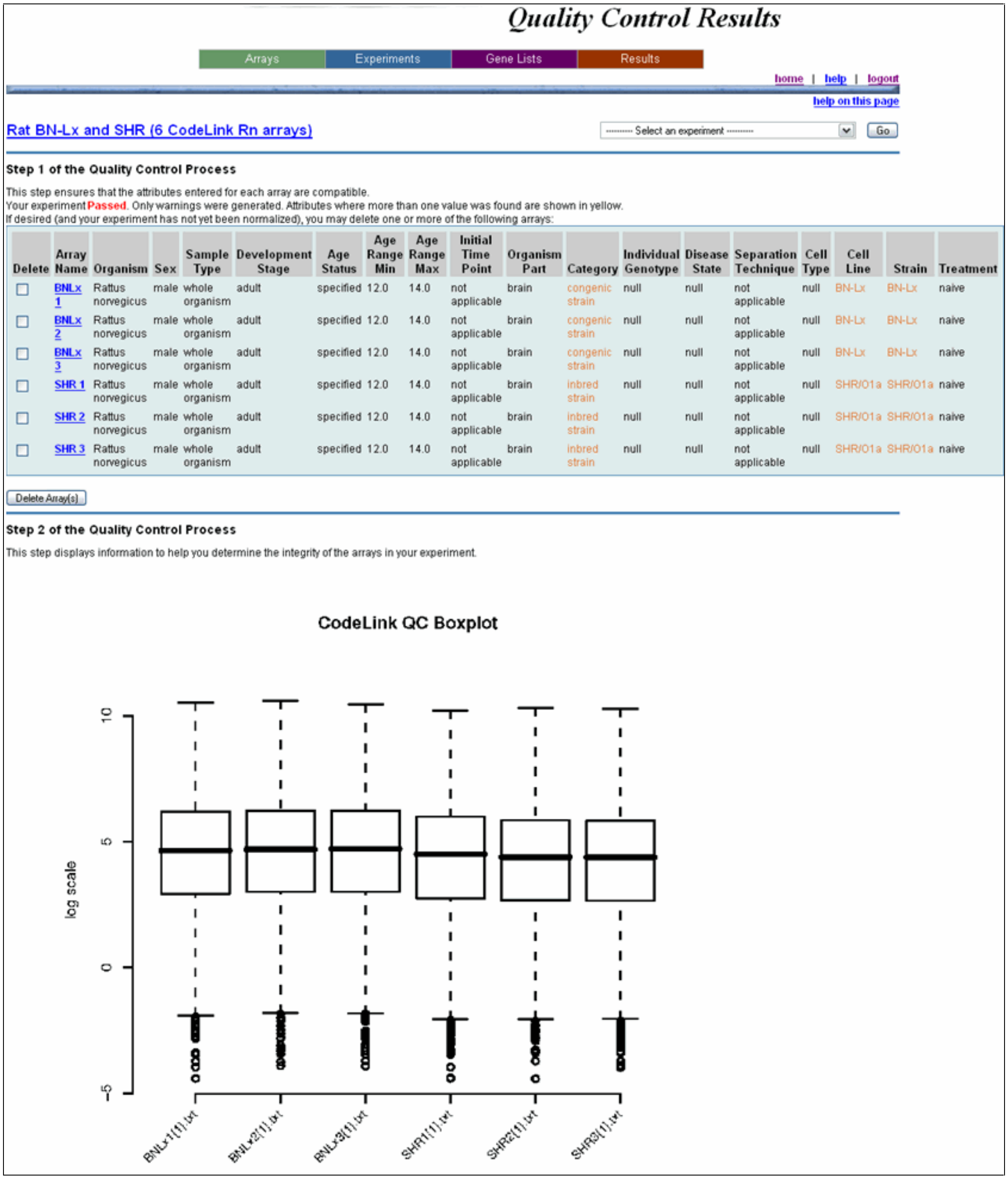

Quality Control Results

Arrays | Experiments | Gene Lists | Results

home | help | logout

help on this page

Rat BN-Lx and SHR (6 CodeLink Rn arrays)

---------- Select an experiment ---------- Go

Step 1 of the Quality Control Process

This step ensures that the attributes entered for each array are compatible.
Your experiment Passed. Only warnings were generated. Attributes where more than one value was found are shown in yellow.
If desired (and your experiment has not yet been normalized), you may delete one or more of the following arrays:

Delete	Array Name	Organism	Sex	Sample Type	Development Stage	Age Status	Age Range Min	Age Range Max	Initial Time Point	Organism Part	Category	Individual Genotype	Disease State	Separation Technique	Cell Type	Cell Line	Strain	Treatment
☐	BNLx 1	Rattus norvegicus	male	whole organism	adult	specified	12.0	14.0	not applicable	brain	congenic strain	null	null	not applicable	null	BN-Lx	BN-Lx	naive
☐	BNLx 2	Rattus norvegicus	male	whole organism	adult	specified	12.0	14.0	not applicable	brain	congenic strain	null	null	not applicable	null	BN-Lx	BN-Lx	naive
☐	BNLx 3	Rattus norvegicus	male	whole organism	adult	specified	12.0	14.0	not applicable	brain	congenic strain	null	null	not applicable	null	BN-Lx	BN-Lx	naive
☐	SHR 1	Rattus norvegicus	male	whole organism	adult	specified	12.0	14.0	not applicable	brain	inbred strain	null	null	not applicable	null	SHR/O1a	SHR/O1a	naive
☐	SHR 2	Rattus norvegicus	male	whole organism	adult	specified	12.0	14.0	not applicable	brain	inbred strain	null	null	not applicable	null	SHR/O1a	SHR/O1a	naive
☐	SHR 3	Rattus norvegicus	male	whole organism	adult	specified	12.0	14.0	not applicable	brain	inbred strain	null	null	not applicable	null	SHR/O1a	SHR/O1a	naive

Delete Array(s)

Step 2 of the Quality Control Process

This step displays information to help you determine the integrity of the arrays in your experiment.

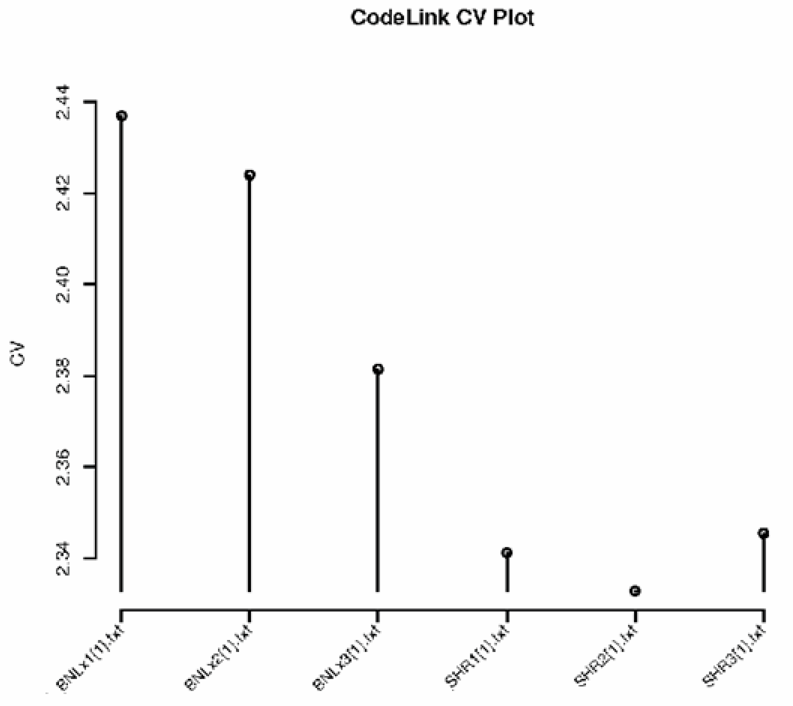

Step 3 of the Quality Control Process

This step displays the pseudo-images and MA plots generated as part of the quality control process.

If you generated images during the quality control process, you may click on the [View ...] links to see images of the array in a separate window. It may take a few minutes to display these images, so please be patient. If desired, after viewing the images, you may delete one or more of the following arrays:

Key: G=Good, L=Near bg signal, C=Contamination, CL=Contamination & Near bg signal, I=Irregular shape, M=Masked, S=Saturated, IS=Irregular shape & Saturated, CI=Contamination & Irregular shape

		Background Values			Number of Probes									
Delete	Array Name	Mean	Max	Min	G	L	C	CL	I	M	S	IS	CI	Image
☐	BNLx 1	53	75	45	24125	10119	4	2	68	25	2	0	0	[View Image]
☐	BNLx 2	52	148	44	24439	9679	69	28	85	42	2	0	1	[View Image]
☐	BNLx 3	53	444	45	24482	9745	4	2	84	25	3	0	0	[View Image]
☐	SHR 1	52	79	46	23500	10698	3	2	126	15	1	0	0	[View Image]
☐	SHR 2	52	871	45	23124	11067	2	4	126	20	1	0	1	[View Image]
☐	SHR 3	52	578	45	23110	11073	5	3	129	23	1	0	1	[View Image]

Delete Array(s)

Viewing oPOSSUM Results

You can view oPOSSUM results for all promoter analyses you have performed using oPOSSUM.

1. Select **Results menu > View All oPOSSUM Results**. The *oPOSSUM Results* page displays.

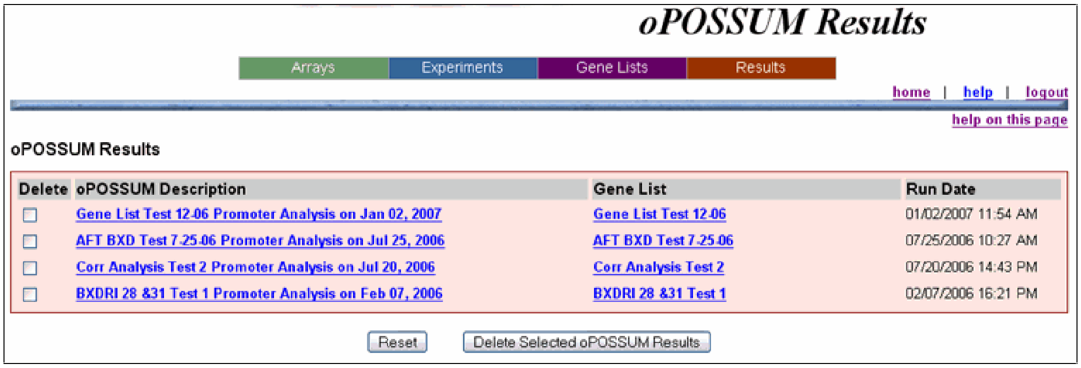

Delete	oPOSSUM Description	Gene List	Run Date
☐	Gene List Test 12-06 Promoter Analysis on Jan 02, 2007	Gene List Test 12-06	01/02/2007 11:54 AM
☐	AFT BXD Test 7-25-06 Promoter Analysis on Jul 25, 2006	AFT BXD Test 7-25-06	07/25/2006 10:27 AM
☐	Corr Analysis Test 2 Promoter Analysis on Jul 20, 2006	Corr Analysis Test 2	07/20/2006 14:43 PM
☐	BXDRI 28 &31 Test 1 Promoter Analysis on Feb 07, 2006	BXDRI 28 &31 Test 1	02/07/2006 16:21 PM

You can:

- Select the **Delete** checkbox and click **Delete Selected oPOSSUM Results** to delete existing oPOSSUM results.
- Click a gene list in the **Gene List** column to view the *Gene List Details* page for that gene list.
- Click a promoter description in the **oPOSSUM Description** column to view the results of that oPOSSUM analysis.

oPOSSUM Results

The *oPOSSUM Results* page for the specific gene list displays the parameters used in the oPOSSUM analysis. It also displays transcription factors based on the One-Tailed Fisher Extract Probability Analysis and the Z-Score Analysis.

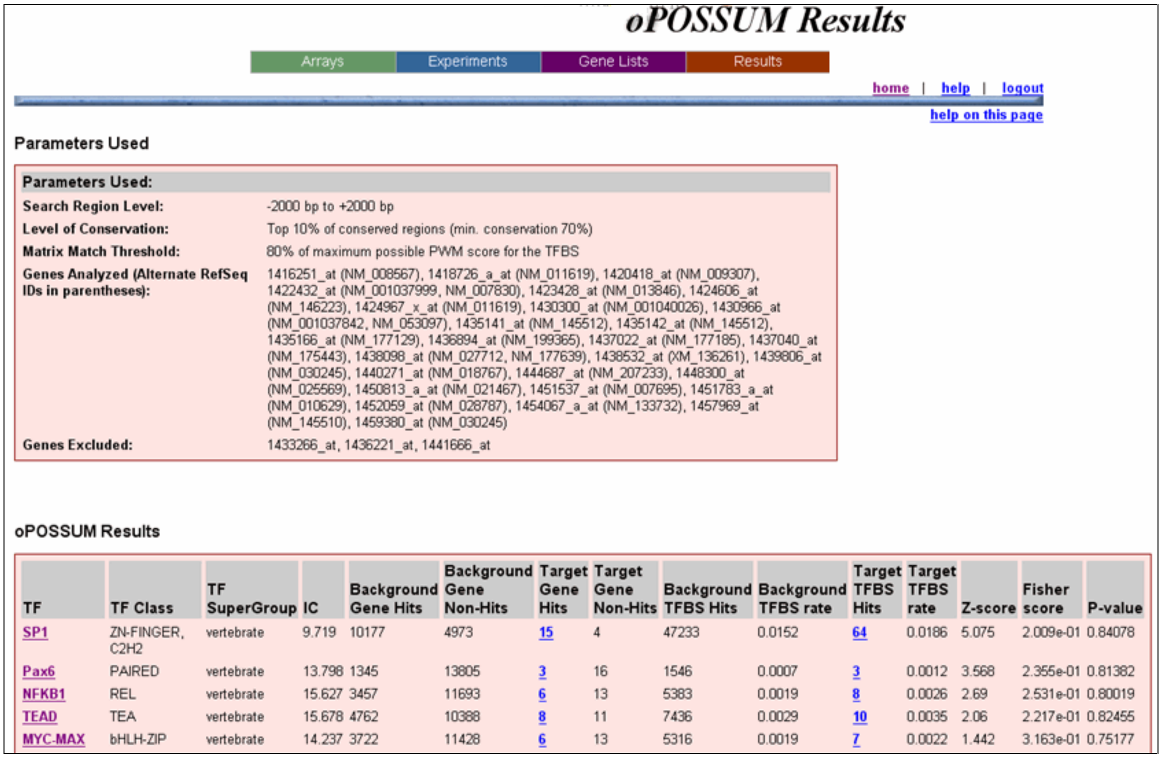

oPOSSUM Results

Arrays | Experiments | Gene Lists | Results

home | help | logout

help on this page

Parameters Used

Parameters Used:	
**Search Region Level:**	-2000 bp to +2000 bp
**Level of Conservation:**	Top 10% of conserved regions (min. conservation 70%)
**Matrix Match Threshold:**	80% of maximum possible PWM score for the TFBS
**Genes Analyzed (Alternate RefSeq IDs in parentheses):**	1416251_at (NM_008567), 1418726_a_at (NM_011619), 1420418_at (NM_009307), 1422432_at (NM_001037999, NM_007830), 1423428_at (NM_013846), 1424606_at (NM_146223), 1424967_x_at (NM_011619), 1430300_at (NM_001040026), 1430966_at (NM_001037842, NM_053097), 1435141_at (NM_145512), 1435142_at (NM_145512), 1435166_at (NM_177129), 1436894_at (NM_199365), 1437022_at (NM_177185), 1437040_at (NM_175443), 1438098_at (NM_027712, NM_177639), 1438532_at (XM_136261), 1439806_at (NM_030245), 1440271_at (NM_018767), 1444687_at (NM_207233), 1448300_at (NM_025569), 1450813_a_at (NM_021467), 1451537_at (NM_007695), 1451783_a_at (NM_010629), 1452059_at (NM_028787), 1454067_a_at (NM_133732), 1457969_at (NM_145510), 1459380_at (NM_030245)
**Genes Excluded:**	1433266_at, 1436221_at, 1441666_at

oPOSSUM Results

TF	TF Class	TF SuperGroup	IC	Background Gene Hits	Background Gene Non-Hits	Target Gene Hits	Target Gene Non-Hits	Background TFBS Hits	Background TFBS rate	Target TFBS Hits	Target TFBS rate	Z-score	Fisher score	P-value
SP1	ZN-FINGER, C2H2	vertebrate	9.719	10177	4973	15	4	47233	0.0152	64	0.0186	5.075	2.009e-01	0.84078
Pax6	PAIRED	vertebrate	13.798	1345	13805	3	16	1546	0.0007	3	0.0012	3.568	2.355e-01	0.81382
NFKB1	REL	vertebrate	15.627	3457	11693	6	13	5383	0.0019	8	0.0026	2.69	2.531e-01	0.80019
TEAD	TEA	vertebrate	15.678	4762	10388	8	11	7436	0.0029	10	0.0035	2.06	2.217e-01	0.82455
MYC-MAX	bHLH-ZIP	vertebrate	14.237	3722	11428	6	13	5316	0.0019	7	0.0022	1.442	3.163e-01	0.75177

One-tailed Fisher Exact Probability Analysis Table

This table contains the results of the Fisher analysis. The results are ordered by p-value from most to least significant (lower to higher p-value). The columns of the table are:

Column Name	Description
TF	The name of the transcription factor.
TF Class	The class of transcription factors to which the transcription factor belongs.
TF SuperGroup	The taxonomic supergroup to which this transcription factor belongs.
IC	The information content or specificity of this TFBS profile's position weight matrix.
Background Gene Hits	The number of genes in the background set for which this TFBS was predicted within the conserved non-coding regions.
Background Gene Non-Hits	The number of genes in the background set for which this TFBS was NOT predicted within the conserved non-coding regions.
Target Gene Hits	The number of genes in the included target set for which this TFBS was predicted within the conserved non-coding regions.
Target Gene Non-Hits	The number of genes in the included target set for which this TFBS was NOT predicted within the conserved non-coding regions.
Background TFBS Hits	The number of times this TFBS was detected within the conserved non-coding regions of the background set of genes.
Background TFBS rate	The rate of occurrence of this TFBS within the conserved non-coding regions of the background set of genes. The rate is equal to the number of times the site was predicted (background hits) multiplied by the with of the TFBS profile, divided by the total number of nucleotides in the conserved non-coding regions of the background gene set.
Target TFBS Hits	The number of times this TFBS was detected within the conserved non-coding regions of the target set of genes.
Target TFBS rate	The rate of occurrence of this TFBS within the conserved non-coding regions of the included target set of genes. The rate is equal to the number of times the site was predicted (target hits) multiplied by the with of the TFBS profile, divided by the total number of nucleotides in the conserved non-coding regions of the included target gene set.
Z-Score	The likelihood that the number of TFBS nucleotides detected for the included target genes is significant as compared with the number of TFBS nucleotides detected for the background set. Z-score is expressed in units of magnitude of the standard deviation.
Fisher Score	The probability that the number of hits vs. non-hits for the included target genes could have occured by random chance based on the hits vs. non-hits for the background set.
P-value	The probability that the number of hits vs. non-hits for the included target genes could have occurred by random chance based on the hits vs. non-hits for the background set.

Deleting an oPOSSUM Analysis

1. Select **Results menu > View All oPOSSUM Results**. The *oPOSSUM Results* page displays.
2. Select a checkbox (or boxes) in the **Delete** column beside the oPOSSUM analysis you want to delete.
3. Click **Delete Selected oPOSSUM Results**. A confirmation box displays.
4. Click **OK** to delete the selected results. Click **Cancel** if you do not want to delete the selected results.

Viewing MEME Results

You can view MEME results for all MEME analyses you have performed.

1. Select **Results menu > View All MEME Results**. The *MEME Results* page displays.

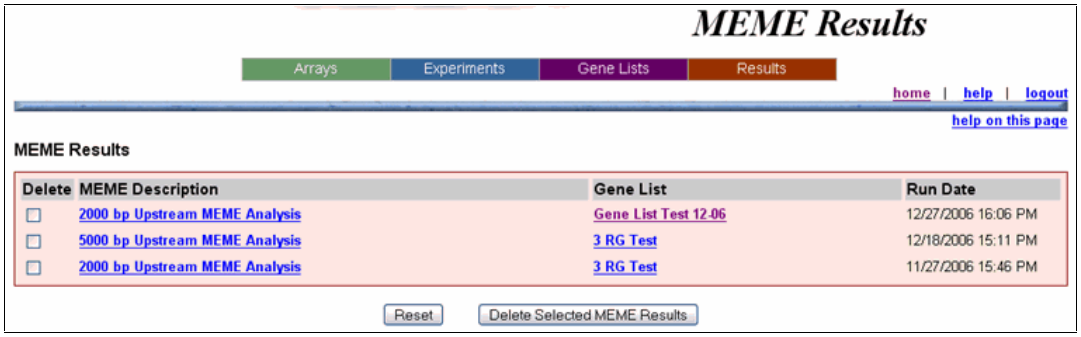

You can:

- Click a MEME description in the **MEME Description** column to view the results of that MEME analysis.
- Click a gene list in the **Gene List** column to view the *Gene List Details* page for that gene list.
- Select the **Delete** checkbox and click **Delete Selected MEME Results** to delete existing MEME results.

MEME Description Results

The *MEME Results* page for the specific gene list displays the MEME results. An explanation of the MEME results is located at the bottom of the results.

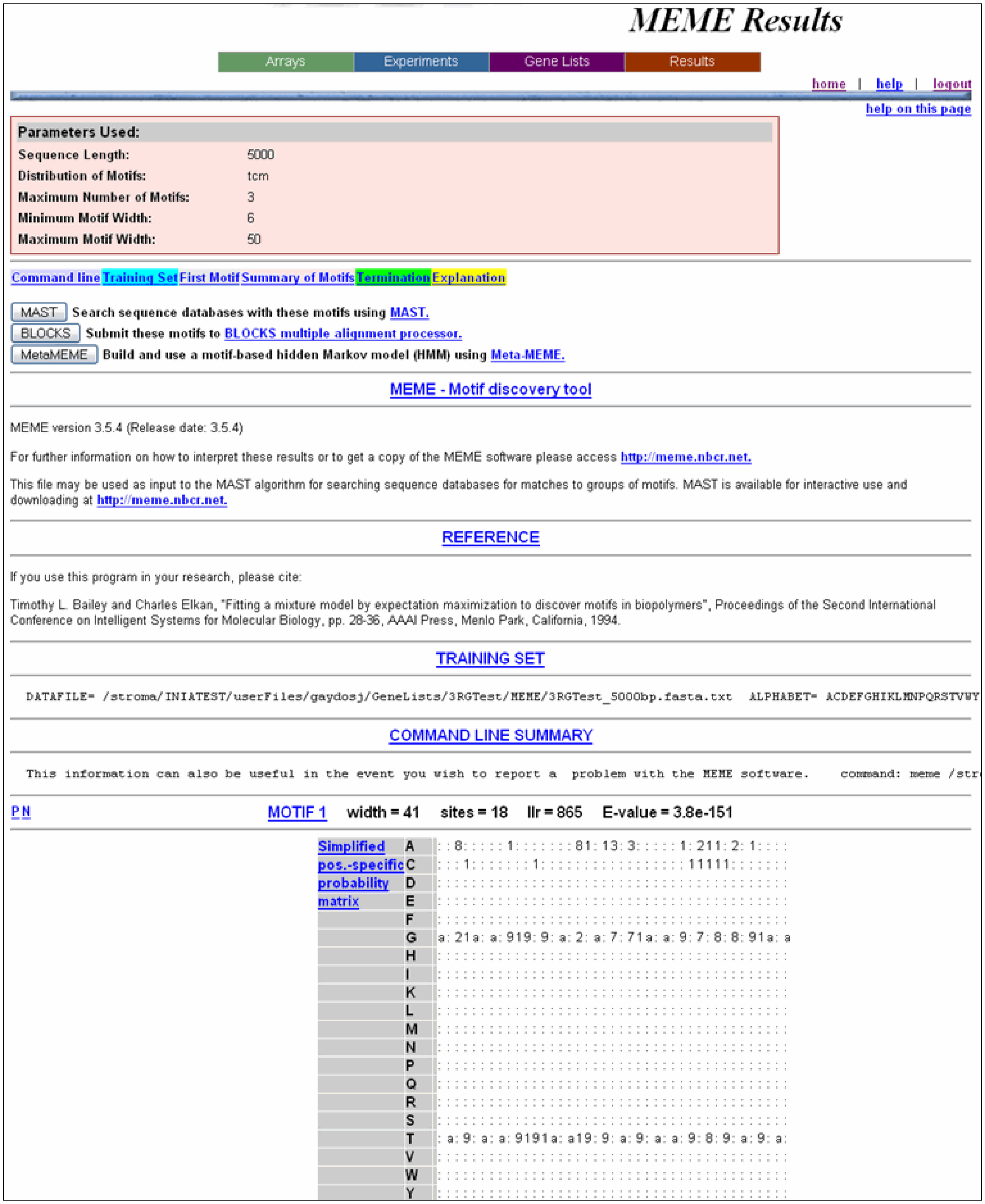

Deleting a MEME Analysis

5. Select **Results menu > View All MEME Results**. The *MEME Results* page displays.
6. Select a checkbox (or boxes) in the **Delete** column beside the MEME analysis you want to delete.
7. Click **Delete Selected MEME Results**. A confirmation box displays.
8. Click **OK** to delete the selected results. Click **Cancel** if you do not want to delete the selected results.

Viewing Upstream Sequence Extraction Results

You can view the upstream sequence extraction results for all of the upstream sequence extractions you have performed.

1. Select **Results menu > View All Upstream Extraction Results**. The *Extraction Results* page displays.

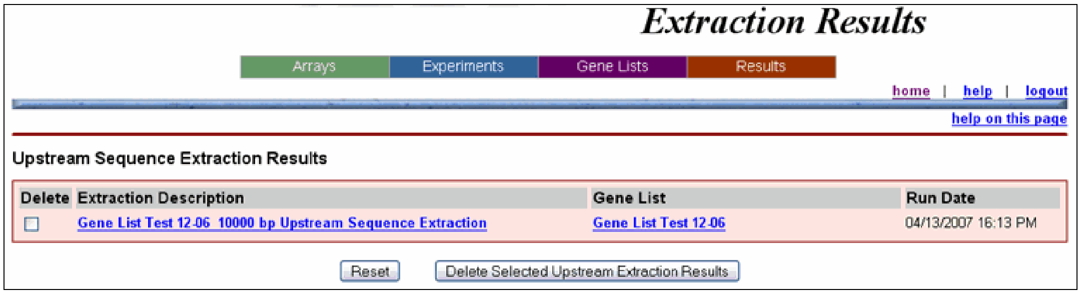

You can:

- Click an extraction description in the **Extraction Description** column to view the upstream sequence extraction results for that upstream sequence extraction.
- Click a gene list in the **Gene List** column to view the *Gene List Details* page for that gene list.
- Select the **Delete** checkbox and click **Delete Selected Upstream Extraction Results** to delete existing upstream sequence extraction results.

Upstream Sequence Extraction Results

The *Results* page for the specific gene list displays the parameters used in the upstream sequence extraction. It also displays the gene identifiers and the upstream sequences for all of the genes in the gene list used. Results can be downloaded using the **Download** button.

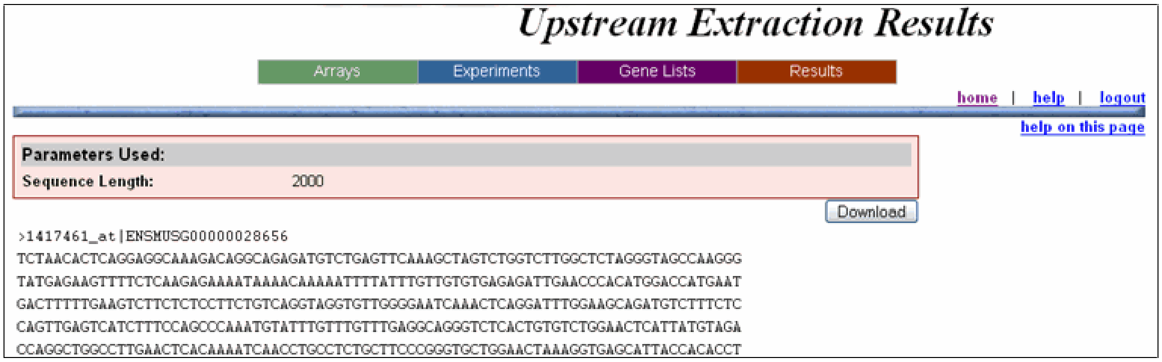

Deleting an Upstream Sequence Extraction

9. Select **Results menu > View All Upstream Sequence Extraction Results**. The *Extraction Results* page displays.
10. Select a checkbox (or boxes) in the **Delete** column beside the upstream sequence extraction you want to delete.
11. Click **Delete Selected Upstream Sequence Extraction Results**. A confirmation box displays.
12. Click **OK** to delete the selected results. Click **Cancel** if you do not want to delete the selected results.

Viewing Literature Search Results

You can view literature search results for all literature searches you have performed, or results for specific gene lists. The Literature Search uses a tool to determine the synonyms for all genes in the gene list that is searched. The search looks for PubMed articles for all synonyms and all the keywords that were entered in the search page. The results provide links to PubMed articles organized by category. In addition, links are provided to articles where more than one gene is referenced in the same article. The PubMed results contain titles and the associated abstracts that are found for the search terms.

1. Select **Results menu** > **View All Literature Results**. The *Literature Search* page displays.

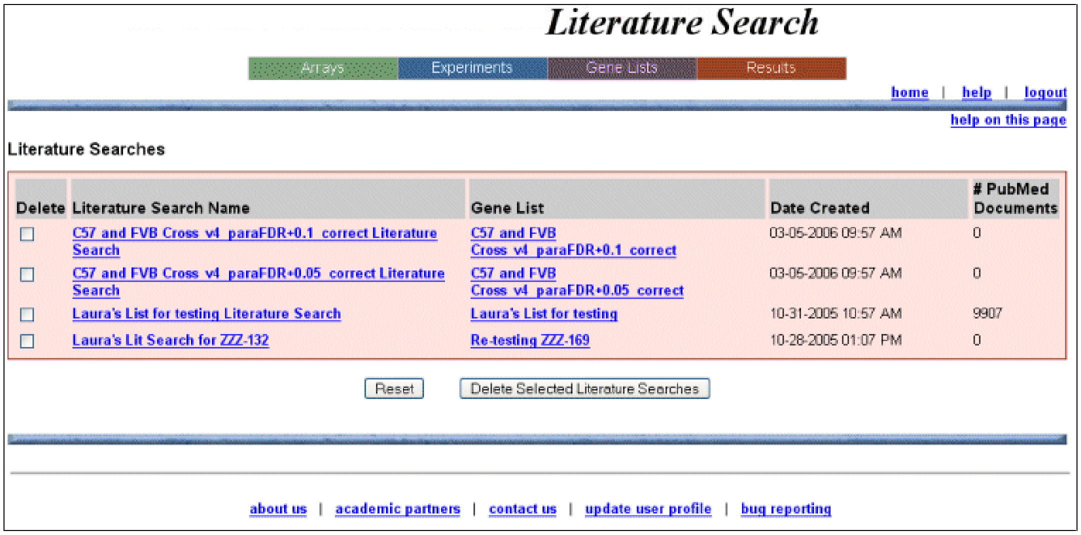

You can:

- Select the **Delete** checkbox and click **Delete Selected Literature Searches** to delete existing Literature Searches.
- Click a name in the **Gene List** column to view the *Gene List Details* page for the selected gene list.
- Click a literature search in the **Literature Search Name** column to view the results of the selected literature search.

Literature Search Name Results

When you click the **Literature Search Name** the following *Literature Search* page, specific to a gene list, displays. It shows the categories and keywords entered. It also displays the results summary where you can click on any of the available **links** to see the PubMed results by gene, category, or gene/category combination. Click **More** to display the synonyms for a gene that were also listed in the search. Below the results summary is a list of coreferenced genes.

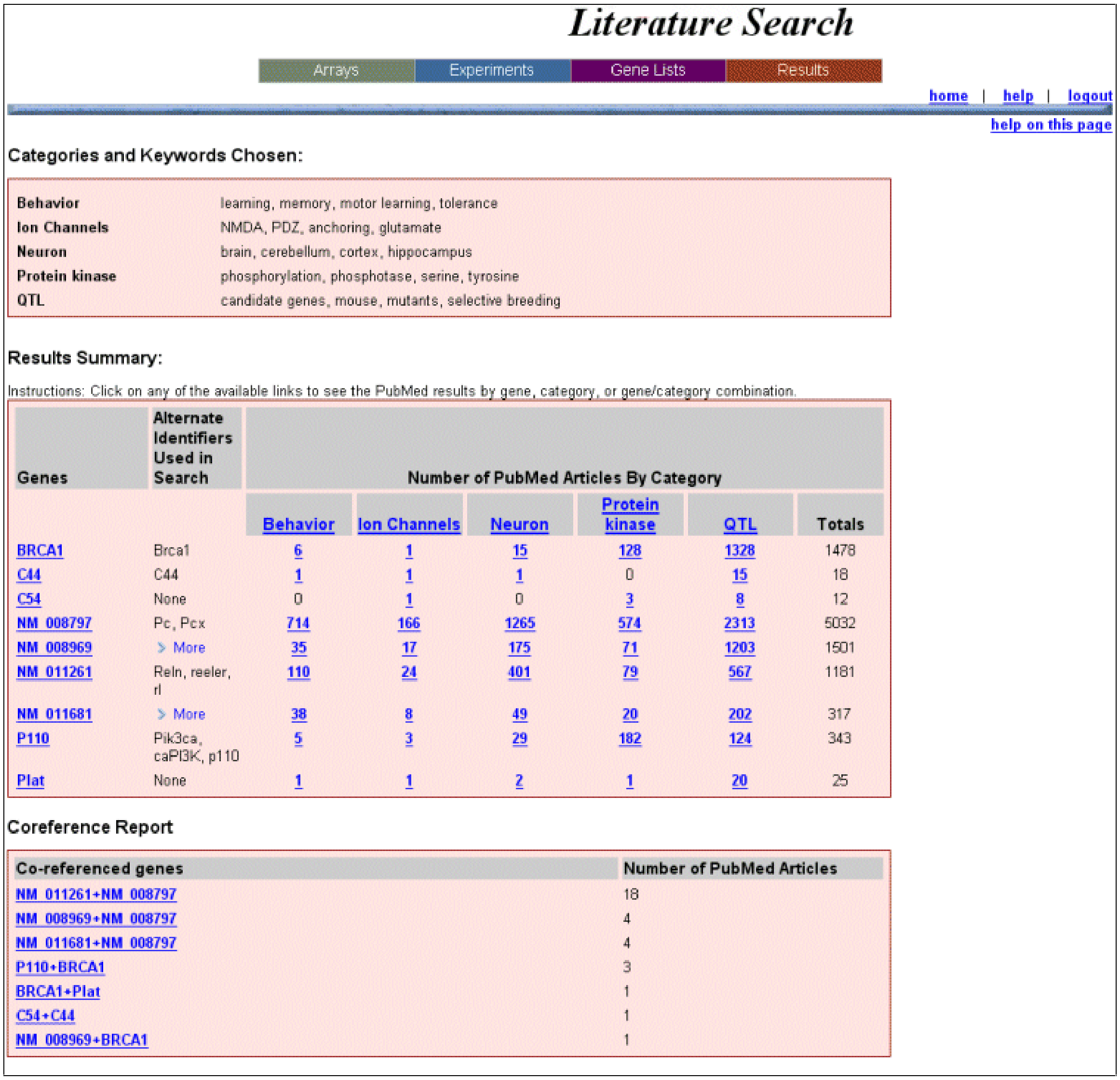

Literature Search

Arrays | Experiments | Gene Lists | Results

home | help | logout

help on this page

Categories and Keywords Chosen:

**Behavior**	learning, memory, motor learning, tolerance
**Ion Channels**	NMDA, PDZ, anchoring, glutamate
**Neuron**	brain, cerebellum, cortex, hippocampus
**Protein kinase**	phosphorylation, phosphotase, serine, tyrosine
**QTL**	candidate genes, mouse, mutants, selective breeding

Results Summary:

Instructions: Click on any of the available links to see the PubMed results by gene, category, or gene/category combination.

Genes	Alternate Identifiers Used in Search	Number of PubMed Articles By Category					
		Behavior	Ion Channels	Neuron	Protein kinase	QTL	Totals
BRCA1	Brca1	6	1	15	128	1328	1478
C44	C44	1	1	1	0	15	18
C54	None	0	1	0	3	8	12
NM_008797	Pc, Pcx	714	166	1265	574	2313	5032
NM_008969	> More	35	17	175	71	1203	1501
NM_011261	Reln, reeler, rl	110	24	401	79	567	1181
NM_011681	> More	38	8	49	20	202	317
P110	Pik3ca, caPI3K, p110	5	3	29	182	124	343
Plat	None	1	1	2	1	20	25

Coreference Report

Co-referenced genes	Number of PubMed Articles
NM_011261+NM_008797	18
NM_008969+NM_008797	4
NM_011681+NM_008797	4
P110+BRCA1	3
BRCA1+Plat	1
C54+C44	1
NM_008969+BRCA1	1

If the results are not specific enough, you can search by gene in the PubMed database for more detailed information.

Deleting a Literature Search

1. Select **Results menu > View All Literature Results**. The *Literature Search* page displays.
2. Select a checkbox (or boxes) in the **Delete** column beside the literature search you want to delete.
3. Click **Delete Selected Literature Searches**. A confirmation box displays.
4. Click **OK** to delete the selected search(es). Click **Cancel** if you do not want to delete the selected search(es).

Principal Investigator

Principal Investigator Overview

Only users with **Principal Investigator permissions** can see the Principal Investigator pages.

The Principal Investigator (PI) is often the head of a lab, and is responsible for the array data. The PI is responsible for granting permission to other users to view the arrays. When arrays are uploaded, the PI can assign permission, individually, to any users that they want to have access to the data. Subsequently, if a user wishes to gain access to the array, they must request permission from the PI of the experiment. The PI can also make array data accessible to all.

A user who is a Principal Investigator sees a Principal Investigator button on the Main page, that provides administrative access for the PI. The PI can:

- Approve array requests.
- Approve registration requests.
- Grant array access to an individual.
- Grant open access to array data.
- Create MAGE-ML files.
- Publish MAGE-ML files to ArrayExpress.

Principal Investigator Menu

The Principal Investigator menu only displays for users with **Principal Investigator permissions**.

The *Principal Investigator* menu displays when you move your mouse over the **Principal Investigator** button.

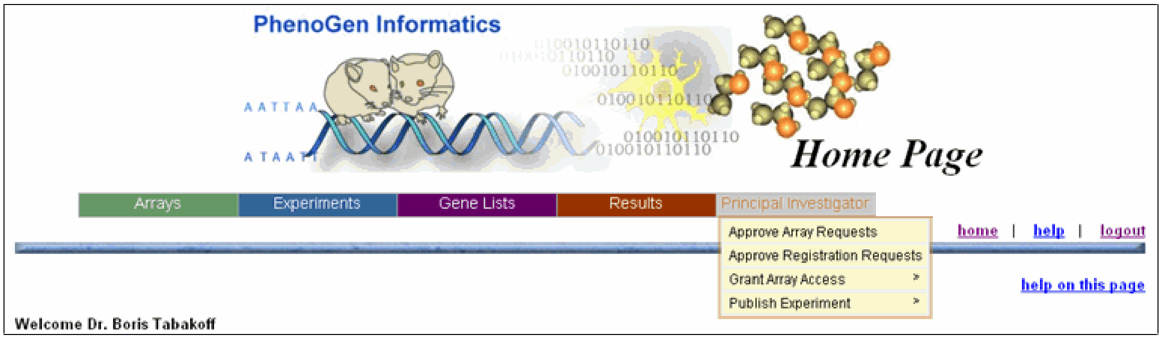

Drop-down Menu Option	Description
Approve Array Requests	Allows you to review requests for access to experiment arrays and approve or deny those requests.
Approve Registration Requests	Allows you to review requests for access to the PhenoGen website and approve or deny those requests.
Grant Array Access	Opens a sub-menu where you can:<br>• *Grant Individual Access* - Allows you to grant array access to an individual (including yourself).<br>• *Grant Open Access* - Allows you to grant open access to array data in a MIAMExpress experiment for **all** registered users of the PhenoGen website.
Publish Experiment	Opens a sub-menu where you can:<br>• *Create MAGE-ML File* - Allows you to create a MAGE-ML file for a specified MIAMExpress experiment.<br>• *Submit to ArrayExpress* - Allows you to submit a MAGE-ML file to EBI's public repository for microarray data, ArrayExpress.

Approving Array Requests

> Only users with **Principal Investigator permissions** can approve pending requests for arrays.

The *Pending Requests* page allows a Principal Investigator to review the users who have requested access to his arrays. The PI can approve or deny access to the array.

1. Select **Principal Investigator menu > Approve Array Requests**. The *Pending Requests* page displays.
2. Select **Approve** or **Deny** next to each request.
3. Click **Approve Array Access**. An email is sent to each requestor that informs them whether their array request(s) has been approved or denied.

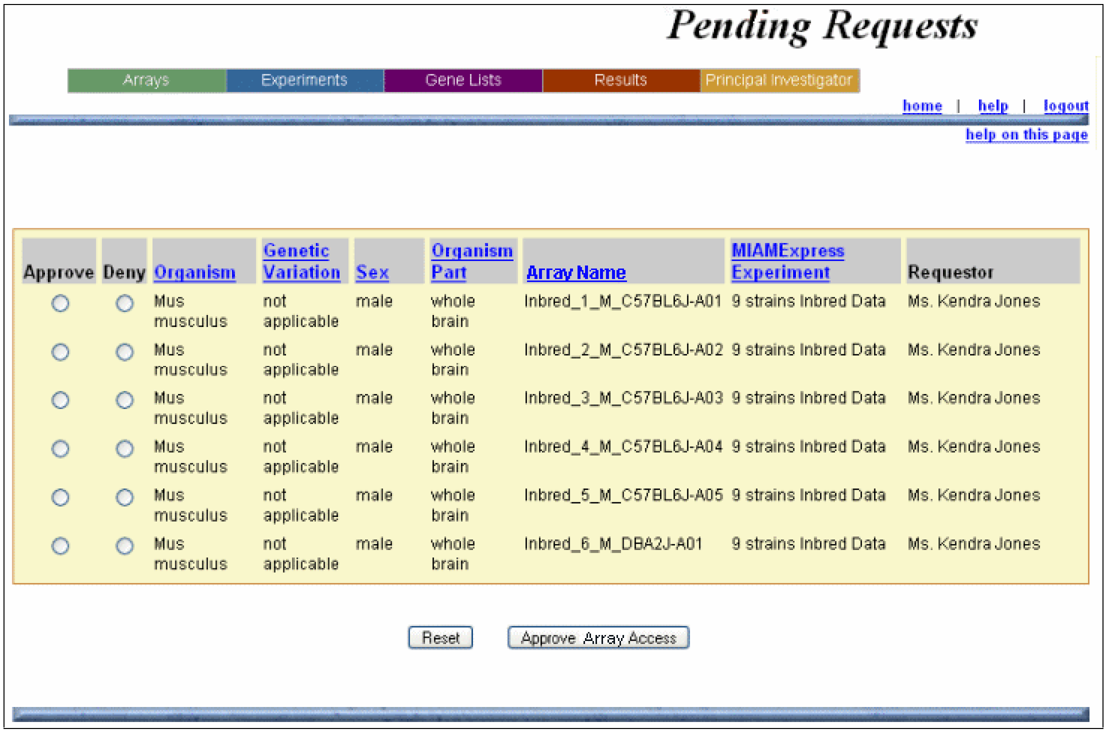

See the topic *Browse All arrays (Logged In)* for details on requesting access to an array.

Approving Registration Requests

Only users with **Principal Investigator permissions** can approve registration requests.

The *Registration Approval* page allows a Principal Investigator to review the users from their laboratory who have requested access to the PhenoGen website. During the registration process, the user must select a Principal Investigator, or declare "self" as the PI. The PI selected receives an email for each user who has submitted a registration stating:

> "*[User Name]* from the *[Institution]* in *[location]* (Email Address: *[user@uchsc.edu]*, Phone Number: *[xxx-yyy-yyyy]*) has completed the registration form and is requesting access to the PhenoGen website.
>
> Please go to *[http://INIAwebsite]* and select 'Approve Registration' from the Principal Investigator menu to approve or deny this person access to the site."

Approving or Denying a Registration Request

1. Select Principal Investigator menu > Approve Registration Requests. The *Registration Approval* page displays.
2. Select **Approve** or **Deny** next to each request. You can leave **Do Nothing** selected if you do not want to approve or deny a request.
3. Click **Approve Registration**. If you approved or denied a user's registration, an email is sent to the user indicating your decision. If you chose to do nothing, no emails are sent.

Granting Array Access

Only users with **Principal Investigator permissions** can grant array access.

Granting Individual Access

The *Grant Individual Access* page allows a Principal Investigator to grant an individual user access to the arrays he is responsible for, and also grant himself access.

1. Select **Principal Investigator menu** > **Grant Array Access** > **Grant Individual Access**. The *Grant Individual Access* page displays.
2. Select a MIAMExpress experiment. Click **Go**.
3. Select a grantee. Click **Select Grantee**.
4. Select from the appropriate buttons whether you want to:
    - Grant Access to the grantee chosen
    - Grant Access to a new individual
    - Grant Access to none

    Notes:
    - If the grantee has access, there is an X in the *Grant to Grantee* column.
    - If the grantee has requested access, the *Grant to Grantee* column says "Pending".
5. Click the **Grant Array Access** button at the bottom of the page.

Note: This only gives access to the current arrays in the PhenoGen array database. Access will have to be re-granted for future additions to the array database.

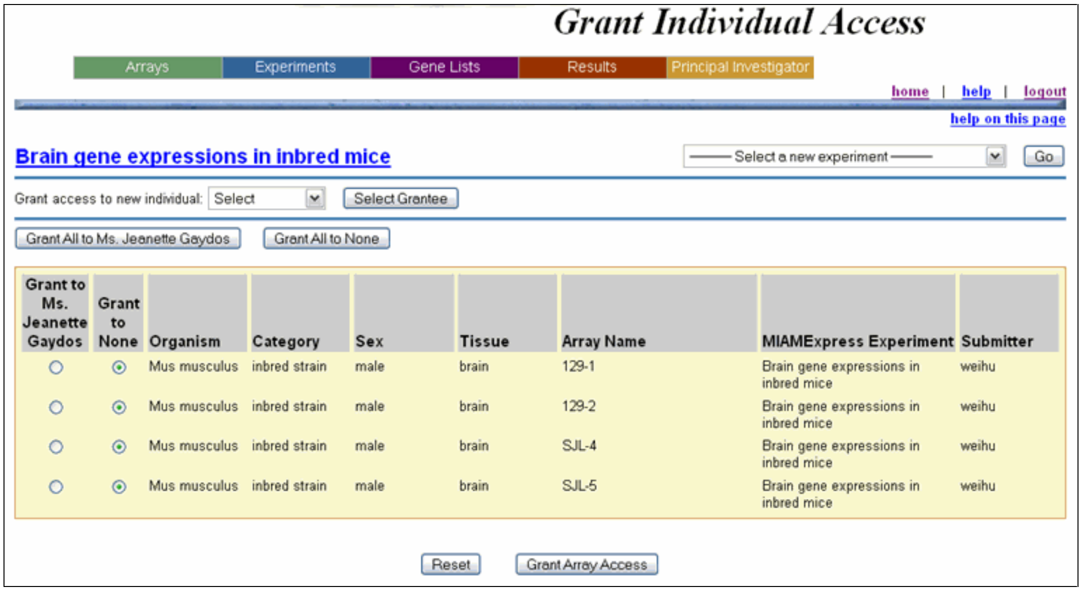

Granting Open Access

The *Grant Open Access* page allows a PI to grant open access to the array data in a MIAMExpress experiment for the use of **ALL** registered users of the PhenoGen website.

1. Select **PI menu > Grant Array Access > Grant Open Access**. The *Grant Open Access* page displays.
2. Select a MIAMExpress experiment name from the drop-down menu.
3. Click **Go**. The MIAMExpress experiment name displays as a hyperlink. The *Experiment Details* page for that experiment can be accessed by clicking on the MIAMExpress experiment name.
4. Click **Grant Open Access**. A *Success* page then displays and the data in the chosen experiment is then available to all registered users of the PhenoGen website.

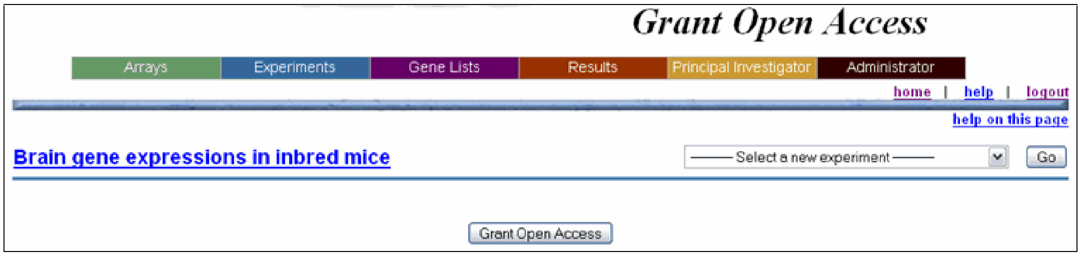

Publishing an Experiment

Only users with **Principal Investigator permissions** can create and publish MAGE-ML files.

Microarray Gene Expression Markup Language (MAGE-ML) is a language created to communicate information about microarray experiments. Based on XML, MAGE-ML can describe microarray designs and manufacturing specifications, experiment set up and protocols, gene expression data, and data analysis results. With the PhenoGen website, PI's can create MAGE-ML files for microarray experiments that can then be submitted to EBI's ArrayExpress. Publishing to ArrayExpress is a two step process. First, a MAGE-ML file for an experiment is created and checked for possible errors. Once correctly created, the MAGE-ML file is then submitted to ArrayExpress.

Creating a MAGE-ML File

1. Select **PI menu** > **Publish Experiment** > **Create MAGE-ML file**. The *Create MAGE-ML file* page displays.
2. Select a **MIAMExpress experiment** from the drop-down menu.
3. Click **Go**. The MIAMExpress experiment name is then displayed as a hyperlink. The *Experiment Details* page for that experiment can be accessed by clicking on the MIAMExpress experiment name.
4. Click **Create MAGE-ML File**. Creating and checking the MAGE-ML file for errors will take some time. An email will be sent to the email you provided in the Registration page informing you if there were any errors.

Note: Errors can be fixed in MIAMExpress before re-running Create MAGE-ML file. Once correctly created, the MAGE-ML file can be submitted to ArrayExpress.

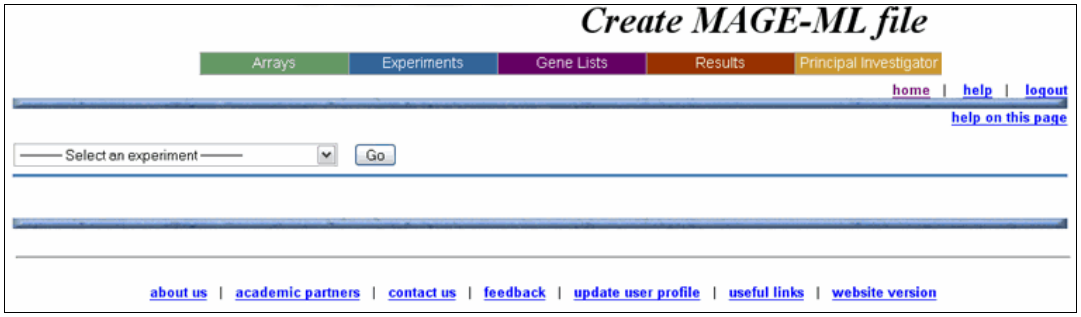

Submitting to ArrayExpress

1. Select **PI menu > Publish Experiment > Submit to Array Express**. The *Submit to ArrayExpress* page displays.
2. Select a MIAMExpress experiment from the drop-down menu.
3. Click **Go**. Submitting the files to ArrayExpress takes some time. An email will be sent to you once your files have been successfully submitted. ArrayExpress is also notified by email of your completed submission.

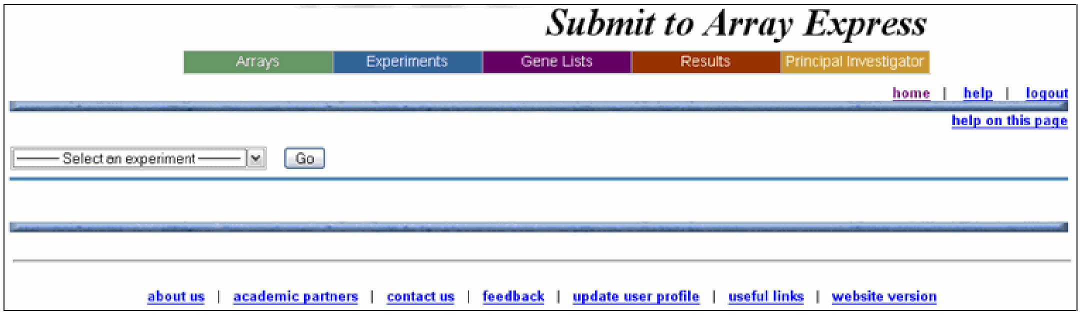

Security

Security Overview

Although data sharing is the primary goal, most researchers prefer to work on their data privately before releasing it to the rest of the Consortium and the scientific community in general. For details, see *Accessing Data* and *Permissions*.

Accessing Data

There are three types of data in the PhenoGen database:

- Arrays
- Experiments and normalized versions of experiments
- Gene lists

Arrays

Arrays are grouped by organism, type (e.g., inbred), sex, tissue, and sample treatment. Each array is the responsibility of a Principal Investigator (PI). The arrays are uploaded for use in the PhenoGen website using MIAMExpress.

If you wish to gain access to the non-public data, you must request permission from the PI who is responsible for the array data. The PI receives email notification of the request and can log into the PhenoGen website to either grant or deny the access request. You are notified via email whether your access request is approved or denied.

A list of all the arrays in the PhenoGen database, grouped by species, can be viewed by all users.

Experiments

You cannot share data at the experiment level. However, you can replicate an experiment:

1. User 1 tells User 2 about the experiment.
2. User 2 requests permission to access the arrays from the appropriate PI(s), if needed.
3. When permission is granted, User 2 selects the exact data as User 1 to replicate the experiment. Then, User 2 normalizes the data in the same way as User 1.
4. Both User 1 and User 2 can view their own copy of the experiment data and version.

While both users have their own experiment and version, the data and normalization are the same for both experiments.

Gene Lists

Data security requirements for gene lists depend on the origin of the gene lists. There are two types of gene lists:

- **A gene list acquired from an experiment.** A gene list generated from the statistical analysis of an experiment is linked to the experiment. You can view gene lists that you created and ones to which you have been given access.
- **An uploaded gene list.** A gene list which is uploaded to the PhenoGen website. The person who uploads the data is defined as the Uploader. When a gene list is uploaded, the Uploader can grant permission to other users to use the gene list for interpretation.

When you own a gene list, you can grant access to other users so they can see your gene list. You can also download any gene list that you own or have permission to view.

Supplementary Information

Supplementary Information contains additional details about various topics.

Reporting Problems, Suggestions and Comments

The quality, functionality, and continued maintenance of the Colorado INIA Informatics (PhenoGen) website depends on feedback from the website users. To facilitate such interactions, an issue management tool called JIRA is used. Users of the PhenoGen website will have access to this tool and can use it to report problems, send suggestions, or request that additional functionality be added to the website. This tool can be accessed by clicking on the **Feedback** link provided at the bottom of the web page.

Once logged into JIRA, you can report a problem (called a "bug") and suggest improvements or new features by clicking on **Create New Issue**. This will start a two step creation process. Once a new issue has been submitted, it will be assigned to a member of the PhenoGen group for resolution. If necessary, you will be contacted for more information.

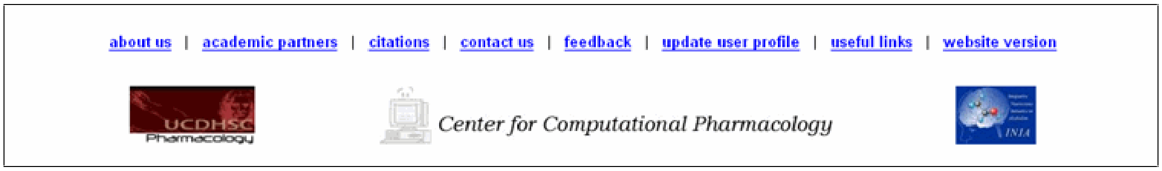

Note: Users need to sign up for an account in JIRA before using this tool. Click on the **Signup** link on the main JIRA page and fill in the required information. Please use the same user name and password that was used for your main PhenoGen website registration.

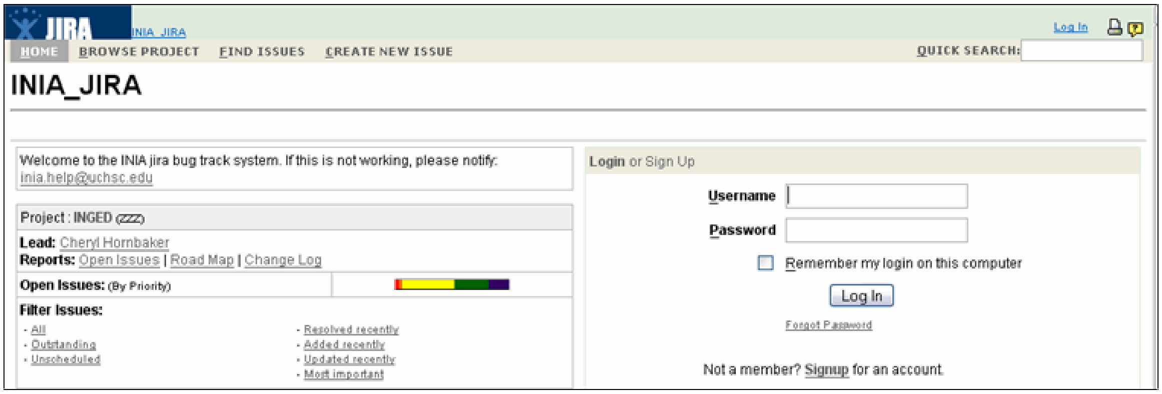

Creating a New Issue in JIRA

1. To access JIRA, click on **Feedback** at the bottom of the web page. This will open the *Feedback* page.
2. Click on **Here**. This will open up the *JIRA Home* page.

Note: You will need to register when accessing JIRA for the first time. Click on **Signup** on the *JIRA Home* page and fill in the required information. Please use your same PhenoGen user name and password when registering.

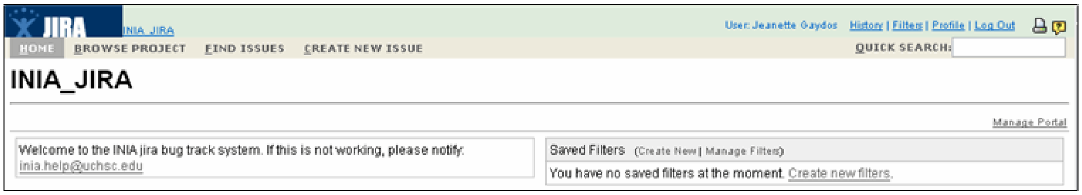

3. Enter your **PhenoGen User Name.**
4. Enter your **PhenoGen Password.**
5. Click on **Create New Issue**. This opens the *Create Issue Step 1* page.

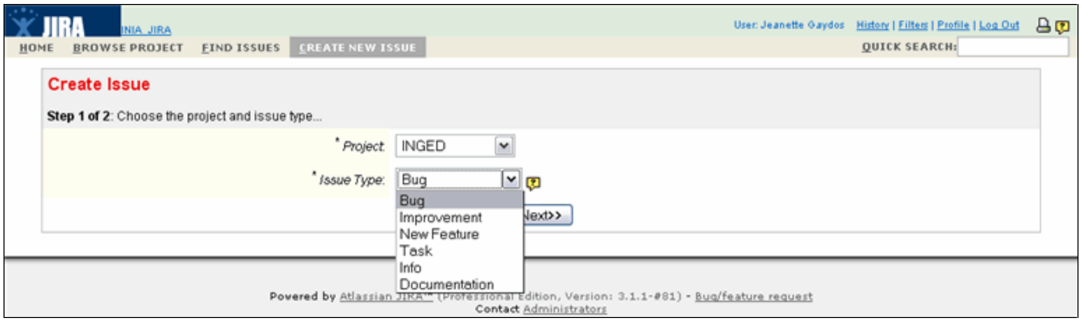

6. Select the project. **INGED** refers to the main PhenoGen website, while **MIAMExpress** is used for issues relating to the PhenoGen local copy of MIAMExpress only.
7. Select the type of issue you would like to create:

- **Bug -** Used for any errors or problems you wish to report.
- **Improvement -** Used for suggestions and comments.
- **New Feature -** Used for requesting additional functionality.
- **Task**
- **Info**
- **Documentation**

8. Click on **Next**. This brings you to the *Create Issue Step 2* page.

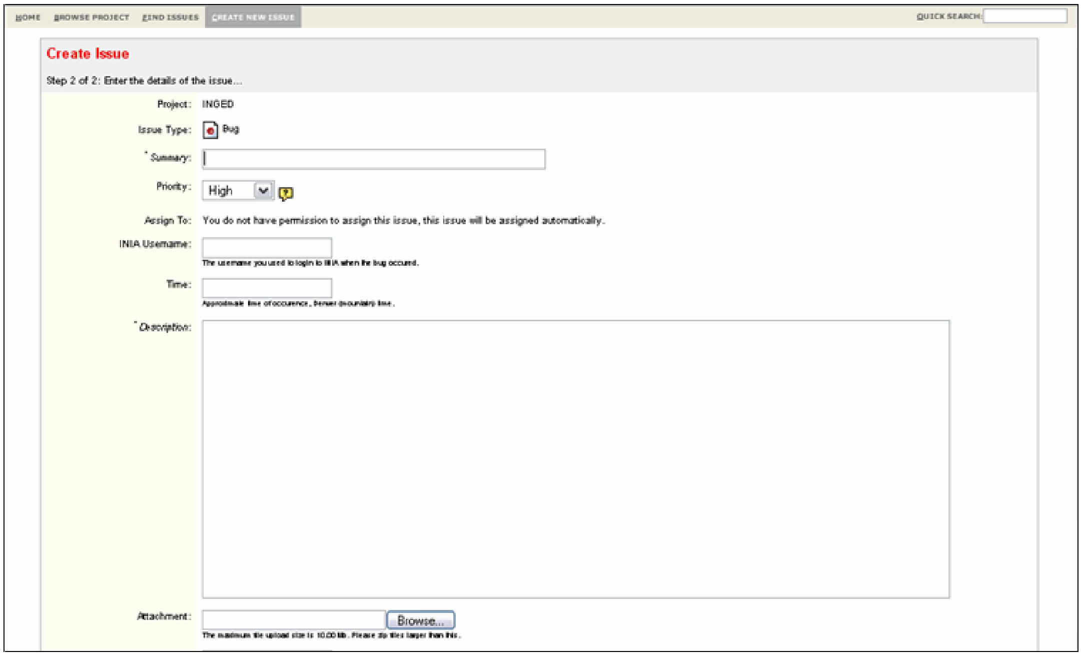

9. Enter the details regarding the issue being addressed. The **Summary** and **Details** fields are mandatory.
10. Click **Create** to submit the issue. If necessary, you will be contacted for further details.

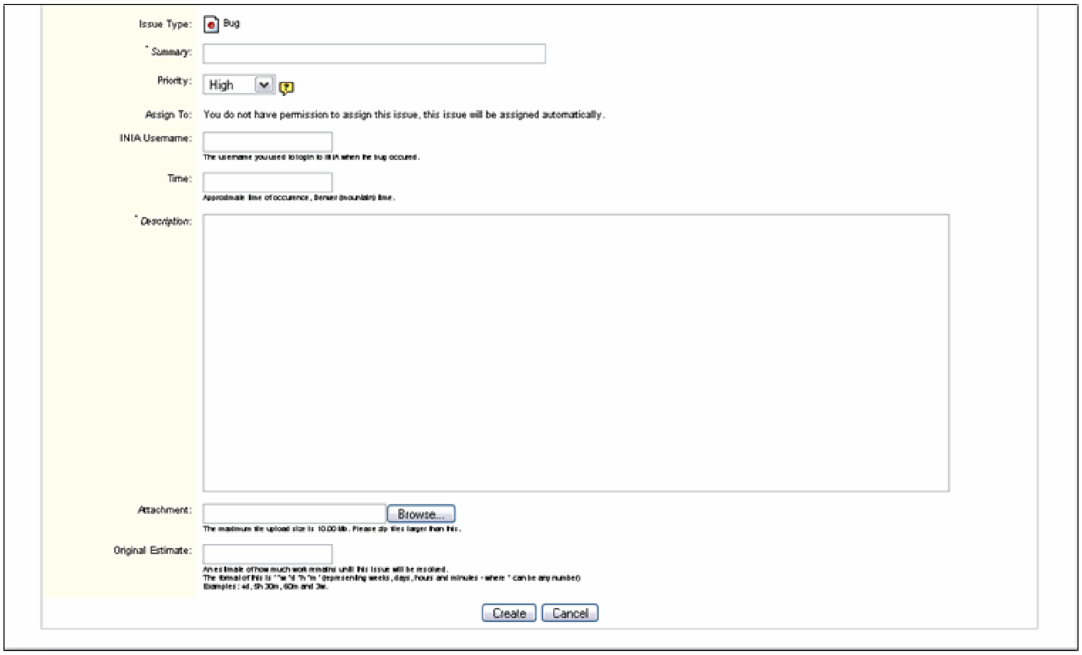

Attaching Files or Screenshots in JIRA

To further clarify an issue in JIRA, you can attach files or screenshots to an issue that you have created. This is especially useful to show specific error messages or illustrate a point you would like to get across. Multiple screenshots can be included together in a Power Point presentation to give a clearer idea of the issue.

1. To create a screenshot, press the **Control** and **Print Screen** buttons on your keyboard at the same time.
2. Open up a new presentation in Power Point and paste the image. Repeat as necessary, then save your presentation.
3. Create the issue in JIRA. Before clicking the **Create** button to finalize the issue, attach your file by clicking on the **Browse** button next to the *Attachment* field at the bottom of the screen. Choose the Power Point presentation you created for this issue and click **Open**.
4. The file path should now be displayed in the *Attachment* field. Click on **Create** and finalize the issue.

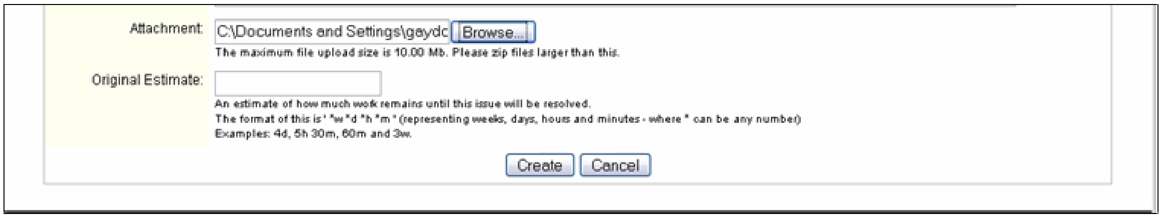

Additional Quality Control Sources

Some of the QC procedures for commonly used microarrays can be found on their manufacturer's websites. For further details, visit the following URLs:

- **cDNA:** http://genearrays.uchsc.edu/
- **Affymetrix:** http://www.affymetrix.com
- **Amersham/GE Healthcare (CodeLink):** http://www.codelinkbioarrays.com

Additional computational QC tools for the R Statistical Package can be found on:

http://arrays.ucsf.edu/analysis/arrayquality.html

http://www.biostat.ucsf.edu/jean/

All About R

R is a language and environment for statistical computing and graphics. R provides a wide variety of statistical (linear and nonlinear modeling, classical statistical tests, time-series analysis, classification, clustering, ...) and graphical techniques, and is highly extensible. R is available as Free Software under the terms of the Free Software Foundation's GNU General Public License in source code form. It compiles and runs on a wide variety of UNIX platforms and similar systems (including FreeBSD and Linux), Windows and MacOS.

For more details about R, see the topics *Viewing the R Project Homepage* and *Viewing R Manuals*.

Citation for R

R Development Core Team (2006). R: A Language and Environment for Statistical Computing. R Foundation for Statistical Computing, Vienna, Austria. http://www.R-project.org

Citation for Bioconductor

Gentleman RC, Carey VJ, Bates DM, Bolstad B, Dettling M, Dudoit S, Ellis B, Gautier L, Ge Y, Gentry J, Hornik K, Hothorn T, Huber W, Iacus S, Irizarry R, Leisch F, Li C, Maechler M, Rossini AJ, Sawitzki G, Smith C, Smyth G, Tierney L, Yang JYH, and Zhang J (2004). Bioconductor: Open software development for computational biology and bioinformatics. Genome Biology 5:R80.

Viewing the R Project Homepage

1. Open a new browser window.
2. Enter the URL http://lib.stat.cmu.edu/R/CRAN/
3. Click **R Homepage** in the vertical menu on the left. The *R Homepage* displays.

Viewing R Manuals

1. Open a new browser window.
2. Enter the URL http://lib.stat.cmu.edu/R/CRAN/
3. Click **Manuals** in the vertical menu on the left. The *R Manuals* page displays.
4. Select and click the manual you want to view.

MIAME Overview

MIAME (Minimum Information About a Microarray Experiment) is a standard for exchanging microarray experimental data in such a way as to make it easily interpreted and allow for easy and independent verification. It is described in the abstract of the original MIAME proposal [1] as follows:

> Microarray analysis has become a widely used tool for the generation of gene expression data on a genomic scale. Although many significant results have been derived from microarray studies, one limitation has been the lack of standards for presenting and exchanging such data. Here we present a proposal, the Minimum Information About a Microarray Experiment (MIAME), that describes the minimum information required to ensure that microarray data can be easily interpreted and that results derived from its analysis can be independently verified. The ultimate goal of this work is to establish a standard for recording and reporting microarray-based gene expression data, which will in turn facilitate the establishment of databases and public repositories and enable the development of data analysis tools. With respect to MIAME, we concentrate on defining the content and structure of the necessary information rather than the technical format for capturing it.

Thus MIAME compliance provides the minimum information required to interpret unambiguously and potentially reproduce and verify an array-based gene expression monitoring experiment. Although details for particular experiments may be different, MIAME aims to define the core that is common to most experiments. MIAME is not a formal specification, but a set of guidelines.

One of the major objectives of MIAME is to guide the development of microarray databases and data management software. A standard microarray data model and exchange format MAGE [2], which is able to capture information specified by MIAME, has been submitted by EBI (for MGED) and Rosetta Biosoftware, and recently became an Adopted Specification of the OMG standards group. Many organizations, including Agilent, Affymetrix, and Iobion, have contributed ideas to MAGE.

Although MIAME concentrates on the content of the information and should not be confused with a data format, it also tries to provide a conceptual structure for microarray experiment descriptions [3].

It is therefore of crucial importance that all users of the PhenoGen site closely conforms to these guidelines. To ensure this, the web site is structured in such a way that very little room is given for non-conformity.

References

[1] Nature Genetics, vol 29 (December 2001), pp 365 – 371. http://www.nature.com/cgi-taf/DynaPage.taf?file=/ng/journal/v29/n4/abs/ng1201-365.html

[2] http://www.mged.org/Workgroups/MAGE/mage.html

[3] http://www.mged.org/Workgroups/MIAME/miame_1.1.html

Promoter Extraction Tools

Program	Operating Principle	Technical Data and URL	Reference
AlignACE	Gibbs sampling algorithm that returns a series of motifs as weight matrices that are over-represented in the input set.	Judges alignments sampled during the course of the algorithm using a maximum a priori likelihood score, which gauges the degree of over-representation. Provides an adjunct measure (group specificity score) that takes into account the sequence of the entire genome and highlights those motifs found preferentially in association with the genes under consideration.<br>http://atlas.med.harvard.edu	1
ANN-Spec	Models the DNA binding specificity of a transcription factor using a weight matrix.	Objective function based on log likelihood that a transcription factor binds at least once in each sequence of the positive training data compared with the number of times it is estimated to bind in the background training data. Parameter fitting is accomplished with a gradient descent method, which includes Gibbs sampling of the positive training examples.<br>http://www.cbs.dtu.dk/~workman/ann-spec/	2
Consensus	Models motifs using weight matrices searching for lhe matrix with maximum information content.	Uses a greedy method, first finding the pair of sequences that share the motif with greatest information content, then finding the third sequence that can be added to the motif, resulting in greatest information content.<br>http://bifrost.wustl.edu/consensus/	3
GLAM	Gibbs sampling-based algorithm that automatically optimizes the alignment width and evaluates the statistical significance of its output.	Since the basic algorithm cannot find multiple motif instances per sequence, long sequences are fragmented into shorter ones, and the alignment is transformed into a weight matrix and used to scan the sequences to obtain the final site predictions.<br>http://zlab.bu.edu/glam/	4
Improbizer	Uses expectation maximization to determine weight matrices of DNA motifs that occur improbably often in the input sequences.	As a background (null) model it uses up to a second-order Markov model of background sequence. Optionally, Improbizer constructs a Gaussian model of motif placement so that motifs that occur in similar positions in the input sequences are more likely to be found.<br>http://www.soe.ucsc.edu/~kent/improbizer	5
MEME	Optimizes the E-value of a statistic related to the information content of the motif.	Rather than sum of information content of each motif column, the statistic used is the product of the p-values of column information contents. The motif search consists of performing expectation maximization from starting points derived from each subsequence occurring in the input sequences. MEME differs from MEME3 mainly in using a correction factor to improve the accuracy of the objective function.	6

Program	Operating Principle	Technical Data and URL	Reference
		http://meme.sdsc.edu	
MITRA	Uses an efficient data structure to traverse the space of IUPAC patterns.	For each pattern, MITRA computes the hypergeometric score of the occurrences in the target sequences relative to the background sequence and reports the highest scoring patterns.<br>http://fluff.cs.columbia.edu:8080/domain/mitra.html	7
MotifSampler	Matrix-based motif finding algorithm that extends Gibbs sampling by modeling the background with a higher order Markov model.	The probabilistic framework is further exploited to estimate the expected number of motif instances in the sequence.<br>http://www.esat.kuleuven.ac.be/~dna/BioI/Software.html	8
Oligo/dyad analysis	Detects over-represented oligonucleotides with oligo analysis and spaced motifs with dyad analysis.	These algorithms detect statistically significant motifs by counting the number of occurrences of each word or dyad and comparing these with expectation. The most crucial parameter is the choice of an appropriate probabilistic model for the estimation of occurrence significance.<br>http://rsat.scmbb.ulb.ac.be/rsat/	9, 10
QuickScore	Based on an exhaustive searching algorithm that estimates probabilities of rare or frequent words in genomic texts.	Incorporates an extended consensus method allowing well-defined mismatches and uses mathematical expressions for efficiently computing z-scores and p-values depending on the statistical models used in their range of applicability. Special attention is paid to the drawbacks of numerical instability. The background model is Markovian, with order up to 3.<br>http://algo.inria.fr/dolley/QuickScore/	11
SeSiMCMC	Modification of Gibbs sampler algorithm that models the motif as a weight matrix, optionally with the symmetry of a palindrome or of a direct repeat and optionally, with spacers.	Includes two alternating stages. The first one optimizes the weight matrix for a given motif and spacer length. The algorithm changes the positions of the motif occurrence in the sequences and infers the motif model from the current occurrences. These changes are used to optimize the likelihood of sequences as being segmented into the (Bernoulli) background and the motif occurrences. The optimization is organized via a Gibbs-like Markov chain that samples positions in sequences one-by-one until the Markov chain converges. The second stage looks for best motif and spacer lengths for obtained motif positions. It optimizes the common information content of motif and of distributions of motif occurrence positions.<br>http://favorov.imb.ac.ru/SeSiMCMC/	12
Weeder	Consensus-based method that enumerates exhaustively all the oligos up to maximum length and collects their	Each motif is evaluated according to the number of sequences in which it appears and how well conserved it is in each sequence, with respect to expected values derived from the oligo frequency analysis of all the available upstream sequences of the same organism. Different combinations of canonical motif parameters derived from the analysis of known instances of yeast transcription factor binding sites (length ranging from 6 to l2, number of substitutions from 1 to 4) are	13

Program	Operating Principle	Technical Data and URL	Reference
	occurrences (with substitutions) from input sequences.	automatically tried by the algorithm in different runs. It also analyzes and compares the top-scoring motifs of each run with a simple clustering method to detect which ones could be more likely to correspond to transcription factor binding sites. Best instances of each motif are selected from sequences using a weight matrix built with sites found by consensus-based algorithms. http://159.149.109.16/Tool/ind.php	
YMF	Uses an exhaustive search algorithm to find motifs with the greatest *z*-scores.	A *p*-value for the *z*-score is used to assess the significance of the motif. Motifs themselves are short sequences over the IUPAC alphabet with spacers ("N"s) constrained to occur in the middle of the sequence. http://bio.cs.washington.edu/software.html#yms	14
Composite Module Analyst (CMA)	Uses a multi-component fitness function for selection of the promoter model which fits best to the observed gene expression profile.	Defines a promoter model based on composition of transcription factor binding sites and their pairs. Adjusts the results of the fitness function using a genetic algorithm for the analysis of functionally related or coexpressed genes. http://www.gene-regulation.com/cgi-bin/CMA/cma.cgi	15
REDUCE	Motif-based regression method for microarray analysis.	The only required inputs are (i) a single genome-wide set of absolute or relative mRNA abundances and (ii) the DNA sequence of the regulatory region associated with each gene that is probed. REDUCE uses unbiased statistics to identify oligonucleotide motifs whose occurrence in the regulatory region of a gene correlates with the level of mRNA expression. Regression analysis is used to infer the activity of the transcriptional module associated with each motif. http://bussemaker.bio.columbia.edu/reduce/	16
MotifRegressor	Combines the advantages of matrix-based motif finding and oligomer motif-expression regression analysis.	MotifRegressor first constructs candidate motifs and then applies regression analysis to select motifs that are strongly correlated with changes in gene expression. It is particularly effective in discovering expression-mediating motifs of medium-to-long width with multiple degenerate positions. MotifRegressor relies in part on MDScan, a software package developed by the Brutlag Lab at Stanford University. http://www.techtransfer.harvard.edu/Software/MotifRegressor/	17
CisModule	Employs a hierarchical mixture approach to model the cis-regulatory module structure.	It is based on the hierarchical mixture model, followed by a*de novo* motif-module discovery algorithm using the Bayesian inference of module locations and within-module motif sites. Dynamic programming-like recursions are developed to reduce the computational complexity from exponential to linear in sequence length. http://www.people.fas.harvard.edu/~qingzhou/CisModule/	18

References

1. Hughes JD, Estep PW, Tavazoie S, Church GM (2000). Computational identification of *cis*-regulatory elements associated with functionally coherent groups of genes in *Saccharomyces cerevisiae*. J Mol Biol 296:1205–1214.
2. Workman CT and Stormo GD (2000). ANN-Spec: a method for discovering transcription factor binding sites with improved specificity. Pacific Symposium on Biocomputing (ed. Altman R, Dunker AK, Hunter L, Klein TE). 467–478 (Stanford University, Stanford, CA).
3. Hertz GZ and Stormo GD (1999). Identifying DNA and protein patterns with statistically significant alignments of multiple sequences. Bioinformatics 15:563–577.
4. Frith MC, Hansen U, Spouge JL, Weng Z (2004). Finding functional sequence elements by multiple local alignment. Nucleic Acids Res 32:189–200.
5. Ao W, Gaudet J, Kent WJ, Muttumu S, Mango SE (2004). Environmentally induced foregut remodeling by PHA-4/FoxA and DAF-12/NHR. Science 305:1743–1746.
6. Bailey TL and Elkan C (1995). The value of prior knowledge in discovering motifs with MEME. Proceedings of the Third International Conference on Intelligent Systems for Molecular Biology. 21–29 (AAAI Press, Menlo Park, CA).
7. Eskin E and Pevzner P (2001). Finding composite regulatory patterns in DNA sequences. Bioinformatics (Supplement 1) 18:S354–S363.
8. Thijs G, *et al* (2001). A higher-order background model improves the detection of promoter regulatory elements by Gibbs sampling. Bioinformatics 17:1113–1122.
9. van Helden J, Andre B, Collado-Vides J (1998). Extracting regulatory sites from the upstream region of yeast genes by computational analysis of oligonucleotide frequencies. J Mol Biol 281:827–842.
10. van Helden J, Rios AF, Collado-Vides J (2000). Discovering regulatory elements in noncoding sequences by analysis of spaced dyads. Nucleic Acids Res 28:1808–1818).
11. Régnier M and Denise A (2004). Rare events and conditional events on random strings. Discrete Math Theor Comput Sci 6:191–214.
12. Favorov AV, Gelfand MS, Gerasimova AV, Mironov AA, Makeev VJ (2004). Gibbs sampler for identification of symmetrically structured, spaced DNA motifs with improved estimation of the signal length and its validation on the ArcA binding sites. Proceedings of BGRS 2004 (BGRS, Novosibirsk).
13. Pavesi G, Mereghetti P, Mauri G, and Pesole G (2004). Weeder Web: discovery of transcription factor binding sites in a set of sequences from co-regulated genes. Nucleic Acids Res 32:W199–W203).
14. Sinha S and Tompa M (2003). YMF: a program for discovery of novel transcription factor binding sites by statistical overrepresentation. Nucleic Acids Res 31:3586–3588.
15. Konovalova T, Valeev T, Cheremushkin E, Kel AE (2005). Composite Module Analyst: Tool for Prediction of DNA Transcription Regulation. Testing on Simulated Data. ICNC 2:1202-1205.
16. Roven C and Bussemaker HJ (2003). REDUCE: an online tool for inferring cis-regulatory elements and transcriptional module activities from microarray data. Nucleic Acids Research 31(13):3487-3490.
17. Conlon EM, Liu XS, Lieb JD, Liu JS(2003). Proc Natl Acad Sci USA 100 (6):3339.
18. Zhou Q and Wong WH (2004). CisModule: De novo discovery of cis-regulatory modules by hierarchical mixture modeling. Proc Natl Acad Sci USA 101:12114-12119.

Glossary

Array: See Microarray.

bQTL: Behavioral QTL.

Chip: See Microarray.

Experiment: 1. In the PhenoGen website: a collection of arrays from one or more MIAMExpress experiments OR a phenotype list to be correlated with the gene expression of a PhenoGen data set. 2. In MIAMExpress: a group of chips that are analyzed at the same time. Each array in the experiment represents a hybridization and is linked to a sample.

iDecoder: iDecoder is a tool that maps gene identifiers between databases. For instance, if database 1 contains entry A and database 2 contains entry B, and both A and B refer to entry C in database 3, but not to each other, iDecoder will identify that A and B are related.

INIA: See Integrative Neuroscience Initiative on Alcoholism.

Integrative Neuroscience Initiative on Alcoholism: A consortium created to identify the alcohol related molecular, cellular, and behavioral neuroadaptations that occur in the brain reward circuits associated with the extended amygdala and its connections.

LIMMA: Linear Models for Microarrays. Collection of tools used for normalization and statistical analysis of cDNA and oligonucleotide arrays.

MIAME: Minimum Information About a Microarray Experiment. MIAME is a standard for exchanging microarray experimental data in such a way as to make it easily interpreted and allow for easy and independent verification.

Microarray: A 2D array, typically on a glass, filter, or silicon wafer, upon which genes or gene fragments are deposited or synthesized in a predetermined spatial order allowing them to be made available as probes in a high-throughput, parallel manner.

Motif: A motif is a sequence pattern that occurs repeatedly in a group of related protein or DNA sequences.

PI: See Principal Investigator.

Principal Investigator: (a.k.a. PI) 1. Often the head of a lab. 2. Typically the array owner. 3. The person responsible for granting permission to other users to view the arrays.

QTL: Quantitative Trait Locus. An area of the genome that is associated with variation in a quantitative trait.

Sample: The MIAMExpress term for array.

Version: After selecting run-groups and normalizing data for a saved experiment or uploading a phenotype list, a "version" of the experiment is created.

Appendix A – eQTL Explorer Software User Guide

Appendix A contains the eQTL Explorer Software User Guide.

eQTL Explorer Software

USER GUIDE

Version 4.0 September 2005

© 2005, MRC

Table of Contents

1. Logging In **154**
Menu Bar 154
2. Main Page Tab **154**
Fetching (Retrieving) Data 155
Changing cis, trans, undefined, and plot by Parameters *155*
Changing p-value/tissue Parameters *155*
Changing max eQTL size *155*
3. Advanced Options Tab **156**
Probeset Options 156
pQTL Options 156
4. Warnings Tab **157**
5. Export Tab **158**
6. The Genome Panel **159**
7. Chromosome Panel **160**
Visualising eQTL Information in Table Format 160
Viewing eQTL and pQTL annotations 161
8. Troubleshooting **165**
9. Additional Information **165**
Contact Us 165
System Requirements 165
Licence 165
Disclaimer 165

1. Logging In

When eQTL Explorer is launched you are presented with the following login window:

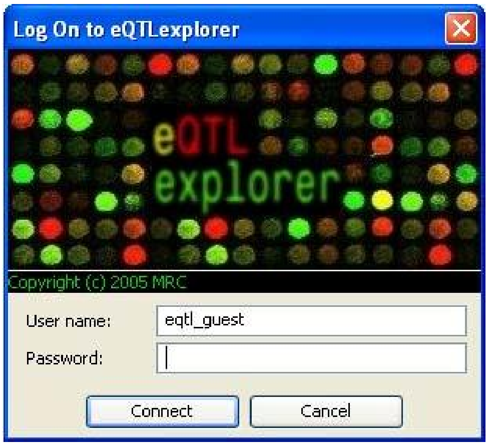

1. Enter **guest** as the User name.
2. Leave the password field blank.
3. Click **Connect** or press **Enter** to start the database connection.

Menu Bar

eQTL Explorer has a menu bar at the top of the page.

- Use the **File** menu to export a jpeg image of the genome panel (at 96dpi) or exit the program.
- Use the **Display** menu to select the number of columns in the Genome Panel, from 1 chromosome to 5 chromosomes wide, in order to visualize large clusters of eQTLs more easily.
- Use the **Help** menu to access a simplified help document and information about the program.

There are also four tabs, *Main Page*, *Advanced Options*, *Warnings*, and *Export*. See the appropriate section for details about each tab.

2. Main Page Tab

When eQTL Explorer has loaded and retrieved experiment information from the database, you are presented with the main screen, where you can retrieve detailed information.

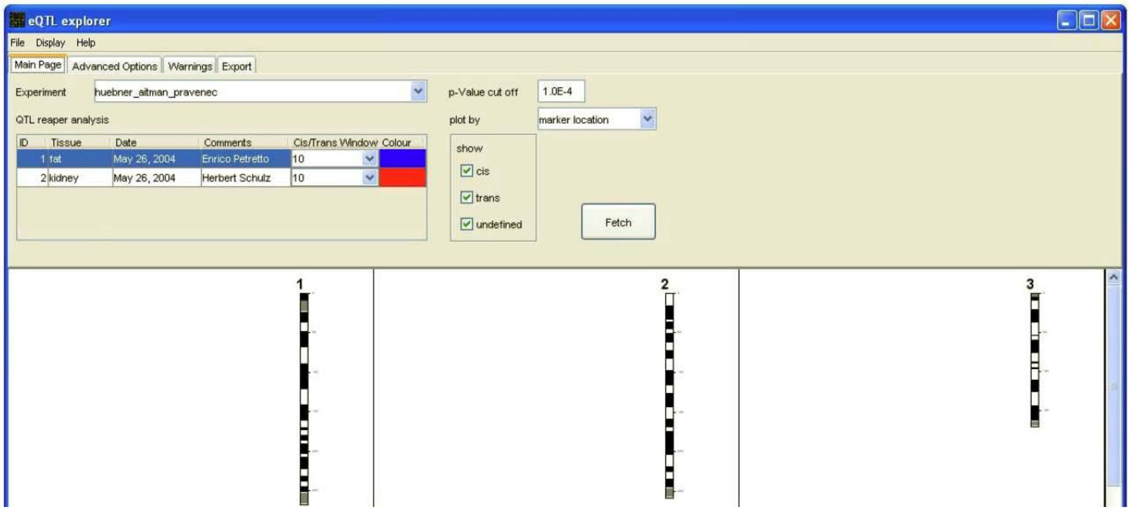

Fetching (Retrieving) Data

1. Choose an experiment from the drop-down list.
2. Change the p-value, if necessary, to any number between 0 and 1. The default p-value is 1.0E-4.
3. Select one of the **plot by** options. The default is by marker location.
4. Choose any of the options in the *Show* block. By default, all options (cis, trans, and undefined) are selected.
5. Click the **Fetch** button after all parameters are selected. eQTL retrieves the data from the database and displays it on the Genome panel.

Changing cis, trans, undefined, and plot by Parameters

After the eQTLs display on the Genome panel, you can change the **cis**, **trans**, and **undefined** checkboxes, or **plot by** option. You do not have to click the **Fetch** button again.

Changing p-value/tissue Parameters

You can change the tissue/p-value parameters, if you choose. Click the **Fetch** button to retrieve eQTLs using the new parameters. If you do not click the **Fetch** button**,** no changes occur on the Genome panel.

*Note: If you select a p-value greater than 0.01, a warning appears. Click the **Fetch** button again to confirm the p-value. This feature uses a p-value of greater than 0.01 may return many data points and take a long time.*

Changing max eQTL size

By default, the window size for defining *cis* and *trans* is set to 10 Mb. You can change the max eQTL window size.

1. Click the drop-down in the **Cis/Trans Window** field.
2. Select a new window size, either 5 Mb or 15 Mb.
3. Click the **Fetch** button to change the graphical output.

3. Advanced Options Tab

You can use the *Advanced Options* tab to filter the information that is retrieved even further.

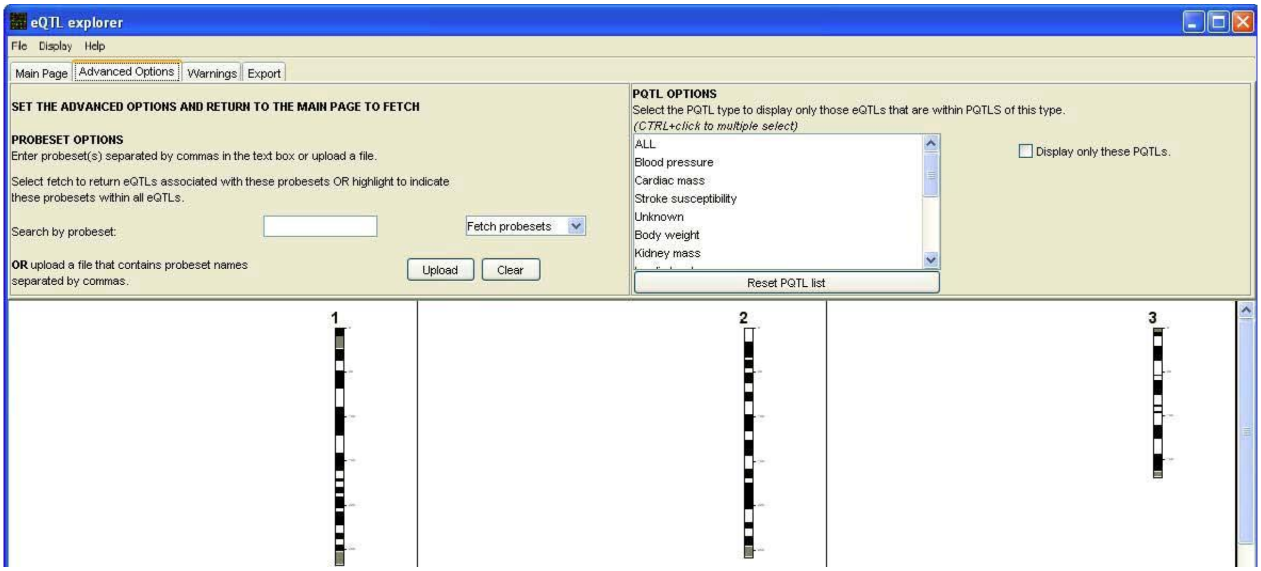

Probeset Options

If you are interested in specific probesets, or a list of probesets, you can enter them as a comma-separated list or upload a file that contains the probeset names separated by commas (on one line).

It is possible to either:

- Highlight eQTLs that contain these probesets This option highlights eQTLs that contain this probeset, with a red circle.
- Fetch only eQTLs that contain these probesets This option displays only those eQTLs that contain the required probesets.

Click the **Fetch** button after you enter the probeset list.

pQTL Options

pQTLs are displayed on the genome panel. When you select a pQTL type or several pQTL types, only those eQTLs that are within the bounds of a pQTL of that type are displayed. You can also display only the pQTLs of a specific type and eQTLs within those pQTLs.

Click the **Fetch** button after you select specific pQTL(s).

4. Warnings Tab

Any information about the retrieval of data displays in this pane.

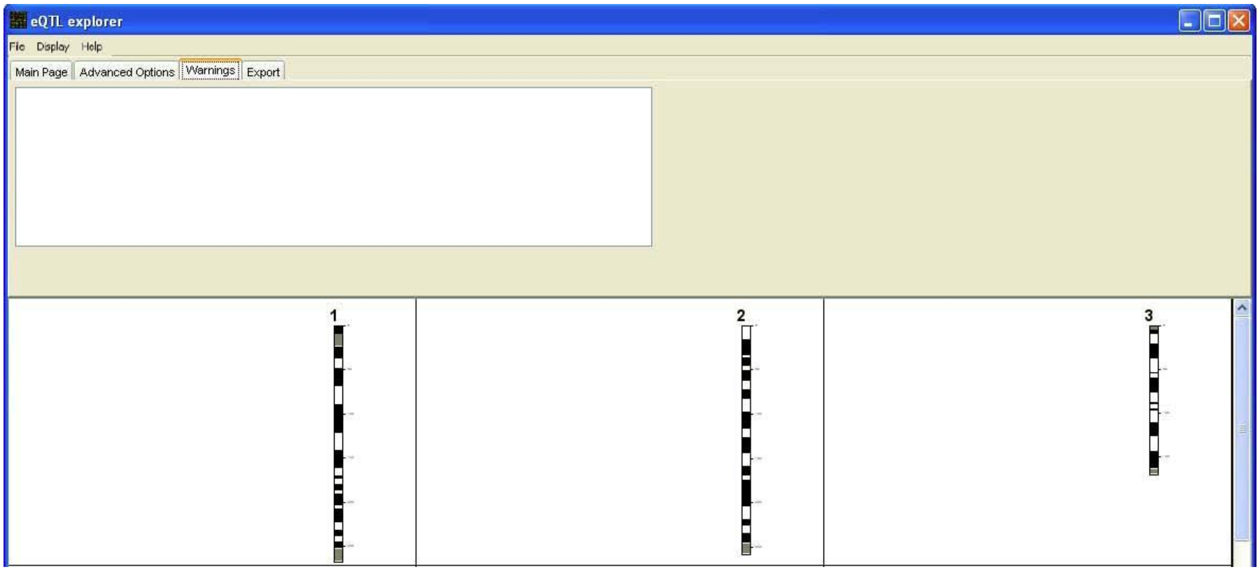

For example, if you select a probeset to fetch and the probeset is not present in an eQTL of the required criteria, you receive a warning in the *Warnings* window. Similarly, if you enter a probeset to highlight, and the probeset is not present amongst the eQTLs, this is reported.

In addition, probeset identifiers that are not in the required format (DIGITS_letters) are reported as warnings.

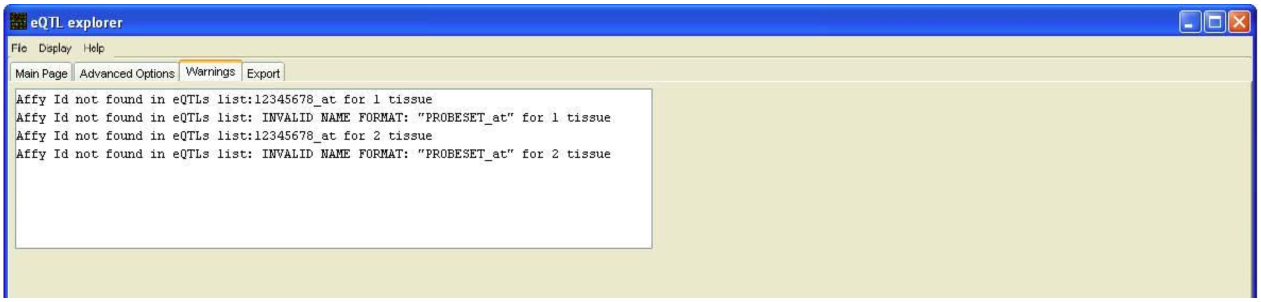

An example of the warnings is displayed above.

5. Export Tab

The export tab gives the option to export information from particular chromosomes, all chromosomes, or specific chromosome regions.

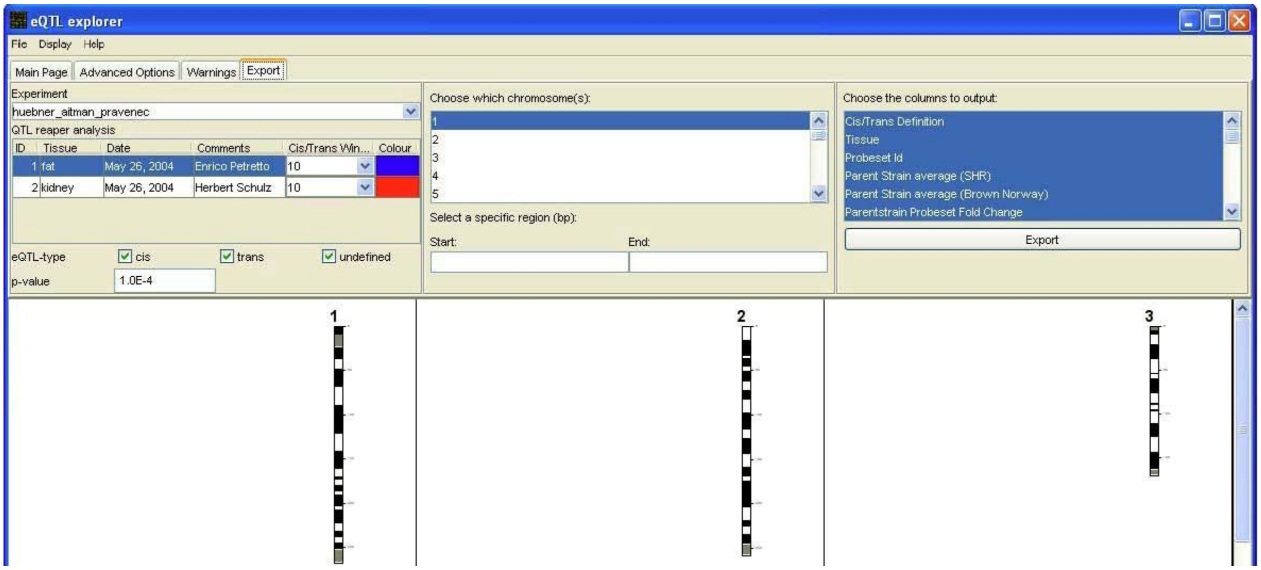

1. Choose an experiment from the drop down list.
2. Click the tissue you are interested in. If you wish to visualize eQTL information for all three tissues, click and drag all three tissue rows under the **QTL reaper analysis** heading.
3. Choose the value of the max eQTL size. By default, the p-value is 1.0E-4. You can change the p-value to any number between 0 and 1.
4. Select one of the **plot by** options. The default is by *marker location*.
5. Choose any of the options in the *Show* block. All options (cis, trans, and undefined) are selected by default.
6. Select a single chromosome, or multiple chromosomes (using CTRL + click), or the ALL option which is at the end of the list. It is also possible to select a specific bp region for one chromosome (or the same region on several/all chromosomes).

   By default, all columns are exported but it is possible to select which columns you want to be written to the file. Selection is in a similar manner to the chromosomes.
7. Click the **Export** button after all parameters are selected. The software asks you for an output file using the typical file request box. The data is fetched from the database and written to the file.

6. The Genome Panel

After the data is fetched, you are presented with a display of eQTLs and pQTLs on the *Genome Panel*. This panel displays all the chromosomes in three columns. You can scroll through the chromosomes using the scrollbar on the right side of the panel. Each chromosome is one chromosome panel demarcated by a black boundary line.

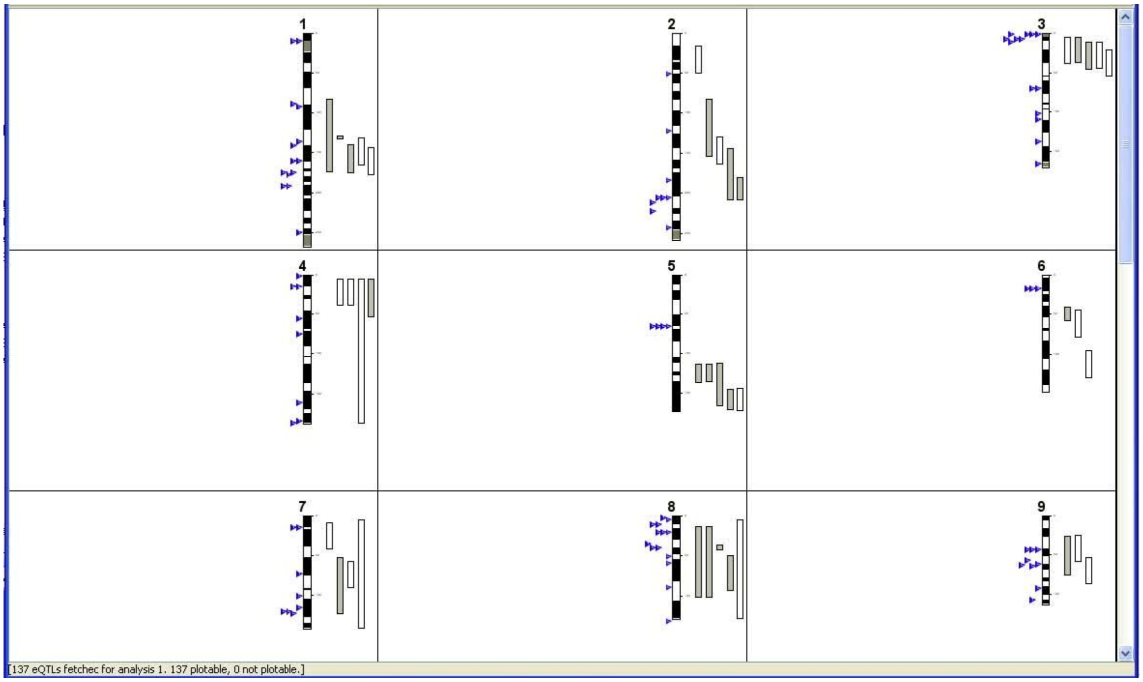

eQTLs in each row are ordered by their p-value. The eQTL with the lowest p-value is nearest to the chromosome. At certain p-values, the number of eQTLs is so large at a particular locus that they reach the extreme left of the boundary of the chromosome panel.

Click a particular chromosome to zoom in and visualize all the eQTLs and their information. The number of eQTLs fetched for each analysis (tissue type) is displayed in the information bar at the bottom of the genome panel.

7. Chromosome Panel

The *Chromosome Panel* is a detailed view of a chromosome.

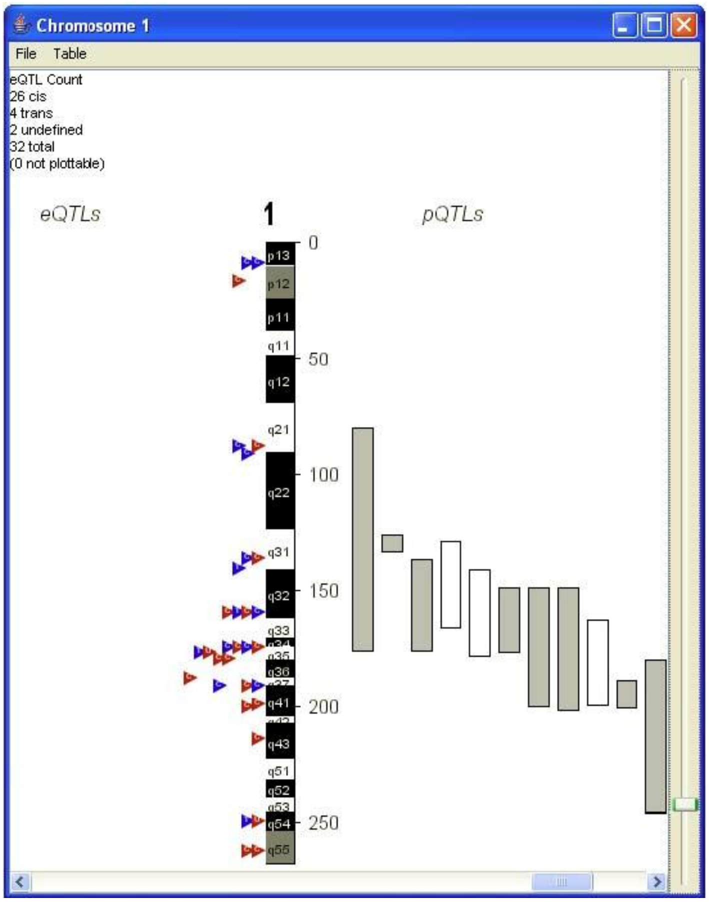

If the eQTLs appear to reach the extreme left boundary of the chromosome panel, click the chromosome anywhere within the black boundary to zoom in on that chromosome. You can scroll towards the left using the scroll bar at the bottom of the chromosome panel.

When you zoom in on a particular chromosome, it minimizes the main window of the software. After you close the zoomed chromosome panel, you have to restore the main software window.

There is a slider on the right side of the chromosome panel and dragging the slider upwards zooms-in on the chromosome further along its y-axis.

Visualizing eQTL Information in Table Format

The chromosome panel has a **Table** option in the menu-bar with two sub-menus:

- **Show Table** allows you to visualize eQTL data in table format.
- **Save Table** allows you to import data in an Excel file to a local disk.

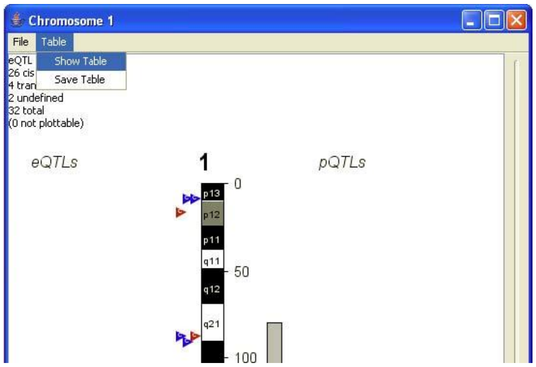

When you click the **Show Table** option, the software imports all the relevant data from the database and populates the table. After the table is populated, it displays on screen. The last four columns have links to external databases. Click any of the cells in those columns to open the respective website.

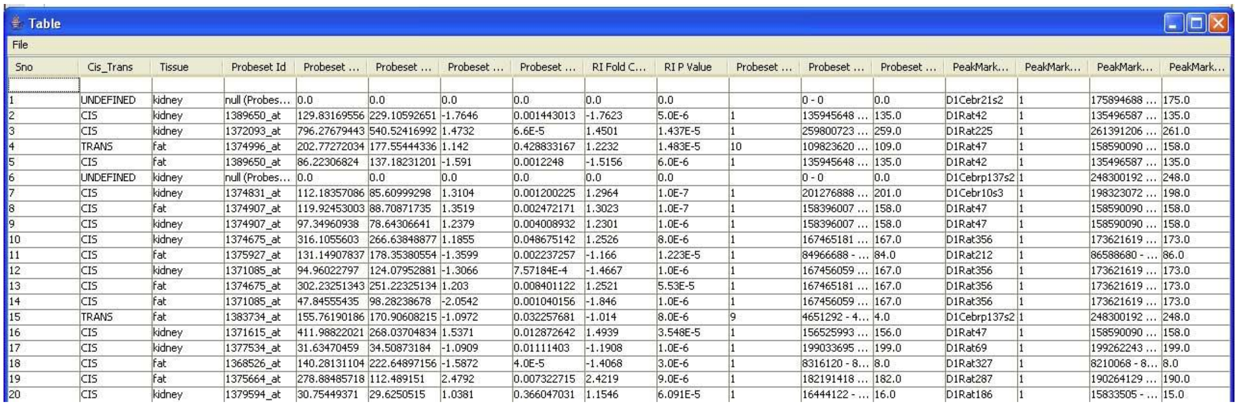

Table
File

Sno	Cis_Trans	Tissue	Probeset Id	Probeset …	Probeset …	Probeset …	Probeset …	RI Fold C…	RI P Value	Probeset …	Probeset …	Probeset …	PeakMark…	PeakMark…	PeakMark…	PeakMark…
1	UNDEFINED	kidney	null (Probes…	0.0	0.0	0.0	0.0	0.0	0.0		0 - 0	0.0	D1Cebr21s2	1	175894688 …	175.0
2	CIS	kidney	1389650_at	129.83169556	229.10592651	-1.7646	0.001443013	-1.7623	5.0E-6	1	135945648 …	135.0	D1Rat42	1	135496587 …	135.0
3	CIS	kidney	1372093_at	796.27679443	540.52416992	1.4732	6.6E-5	1.4501	1.437E-5	1	259800723 …	259.0	D1Rat225	1	261391206 …	261.0
4	TRANS	fat	1374996_at	202.77272034	177.55444336	1.142	0.428833167	1.2232	1.483E-5	10	109823620 …	109.0	D1Rat47	1	158590090 …	158.0
5	CIS	fat	1389650_at	86.22306824	137.18231201	-1.591	0.0012248	-1.5156	6.0E-6	1	135945648 …	135.0	D1Rat42	1	135496587 …	135.0
6	UNDEFINED	kidney	null (Probes…	0.0	0.0	0.0	0.0	0.0	0.0		0 - 0	0.0	D1Cebrp137s2	1	248300192 …	248.0
7	CIS	kidney	1374831_at	112.18357086	85.60999298	1.3104	0.001200225	1.2964	1.0E-7	1	201276888 …	201.0	D1Cebr10s3	1	198323072 …	198.0
8	CIS	fat	1374907_at	119.92453003	88.70871735	1.3519	0.002472171	1.3023	1.0E-7	1	158396007 …	158.0	D1Rat47	1	158590090 …	158.0
9	CIS	kidney	1374907_at	97.34960938	78.64306641	1.2379	0.004008932	1.2301	1.0E-6	1	158396007 …	158.0	D1Rat47	1	158590090 …	158.0
10	CIS	kidney	1374675_at	316.1055603	266.63848877	1.1855	0.048675142	1.2526	8.0E-6	1	167465181 …	167.0	D1Rat356	1	173621619 …	173.0
11	CIS	fat	1375927_at	131.14907837	178.35380554	-1.3599	0.002237257	-1.166	1.223E-5	1	84966688 - …	84.0	D1Rat212	1	86588680 - …	86.0
12	CIS	kidney	1371085_at	94.96022797	124.07952881	-1.3066	7.57184E-4	-1.4667	1.0E-6	1	167456059 …	167.0	D1Rat356	1	173621619 …	173.0
13	CIS	fat	1374675_at	302.23251343	251.22325134	1.203	0.008401122	1.2521	5.53E-5	1	167465181 …	167.0	D1Rat356	1	173621619 …	173.0
14	CIS	fat	1371085_at	47.84555435	98.28238678	-2.0542	0.001040156	-1.846	1.0E-6	1	167456059 …	167.0	D1Rat356	1	173621619 …	173.0
15	TRANS	fat	1383734_at	155.76190186	170.90608215	-1.0972	0.032257681	-1.014	8.0E-6	9	4651292 - 4…	4.0	D1Cebrp137s2	1	248300192 …	248.0
16	CIS	kidney	1371615_at	411.98822021	268.03704834	1.5371	0.012872642	1.4939	3.548E-5	1	156525993 …	156.0	D1Rat47	1	158590090 …	158.0
17	CIS	kidney	1377534_at	31.63470459	34.50873184	-1.0909	0.01111403	-1.1908	1.0E-6	1	199033695 …	199.0	D1Rat69	1	199262243 …	199.0
18	CIS	fat	1368526_at	140.28131104	222.64897156	-1.5872	4.0E-5	-1.4068	3.0E-6	1	8316120 - 8…	8.0	D1Rat327	1	8210068 - 8…	8.0
19	CIS	fat	1375664_at	278.88485718	112.489151	2.4792	0.007322715	2.4219	9.0E-6	1	182191418 …	182.0	D1Rat287	1	190264129 …	190.0
20	CIS	kidney	1379594_at	30.75449371	29.6250515	1.0381	0.366047031	1.1546	6.091E-5	1	16444122 - …	16.0	D1Rat186	1	15833505 - …	15.0

Viewing eQTL and pQTL Annotations

Place the mouse cursor over the eQTL or pQTL and a tool tip displays information about that particular eQTL or pQTL.

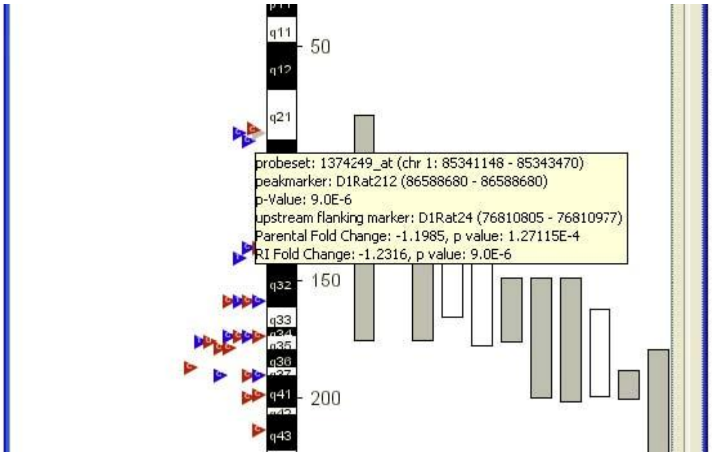

In the image above, the following information is displayed by placing the mouse cursor over eQTL symbol -

- **Probe set:** Probe set ID and genomic location of the probe sequence.
- **Peakmarker:** Marker ID and genomic location of the marker.

- **P-value:** p-value, for the peak LOD score associated with the marker, obtained from QTL Reaper analysis.
- **Upstream flanking marker:** Marker ID for the upstream flanking marker.
- **Parental fold change:** Differences in the gene expression intensity values obtained for that probe set, between parental strains, were calculated using 6 naïve male mice per strain, C57BL/6J and DBA/2J. Fold change was calculated as the difference in mean values of RMA log base 2 transformed intensity data.
  A positive fold change represents a higher intensity in C57 strain then in the DBA strain. A negative fold change represents the opposite, higher in the DBA strain. P-values were calculated based on a two-sample t-test assuming equal variances. P-values have NOT been adjusted for multiple comparisons.
- **RI fold change:** Differences in the gene expression intensity values, obtained for that probe set, in the recombinant inbred strains were based on the 160 naïve male mice from 30 RI strains used to calculate eQTLs. Mean expression values were calculated within strain for each transcript independently, using the RMA log base 2 transformed intensity data. The genotype of the strain at the marker with the maximum LOD (LRS) score for that transcript was used to separate strains into two groups. A fold change was calculated as the difference between the means of these two genotype groups, which represents a difference in the means (within genotype group) of the means (within strain). This was done because the number of mice per strain was not consistent across strains and each strain should contribute equally to the genotype group mean. A positive fold change represents a higher intensity in the RI strains with the C57 allele at the marker with the maximum LOD (LRS) than in RI strain with the DBA allele at that marker. A negative fold change represents the opposite, higher in the RI strains with the DBA allele. P-values were calculated based on a two-sample t-test assuming equal variances. P-values have NOT been adjusted for multiple comparisons.

And similarly, for pQTLs:

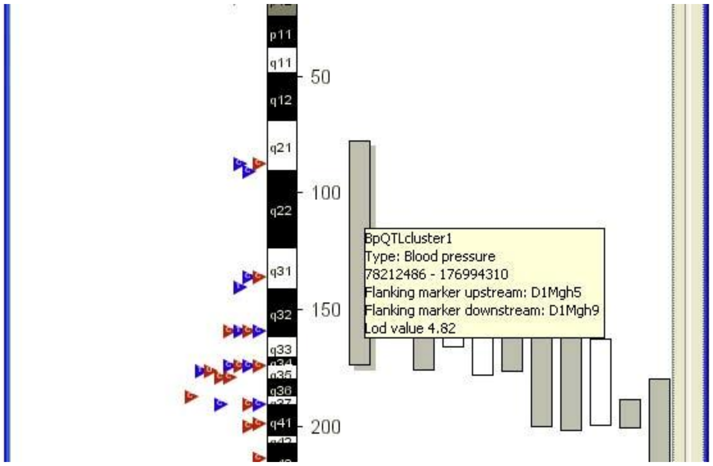

eQTLs Probeset Information

You can access links to external sources of information when you left-clicking on the eQTL or pQTL. eQTLs link to information for probesets and markers. A pop-up box displays that provides links to Ensembl/ RGD/ Entrez databases. Please select any of the options to view information about that particular eQTL/pQTL from the database.

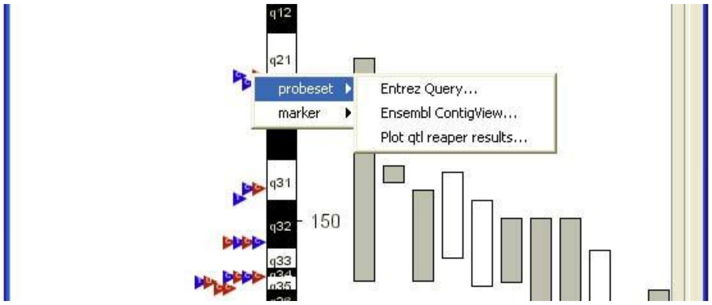

eQTLs Marker Information

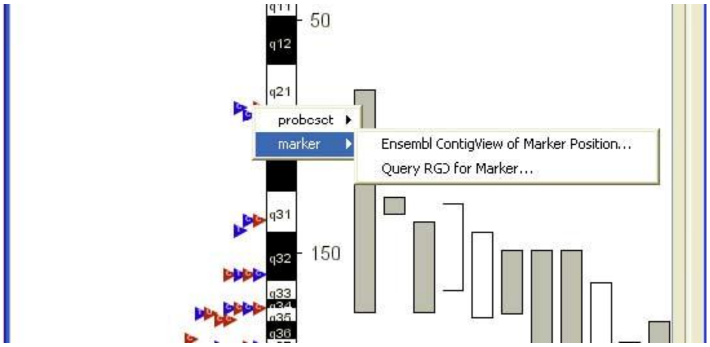

pQTLs

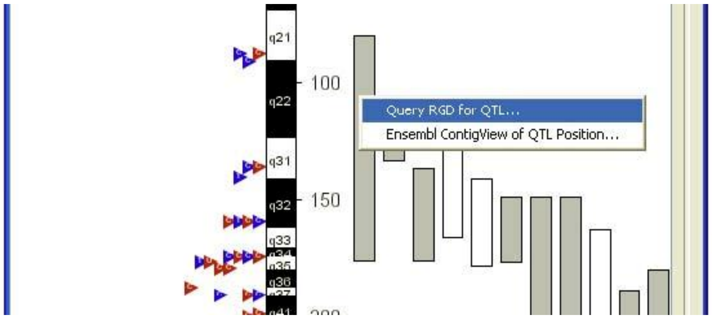

Plot QTL Reaper Results

For eQTL probesets, the **Plot qtl reaper results…** option gives a Genome-wide plot of the linkage for that particular probeset in that particular tissue.

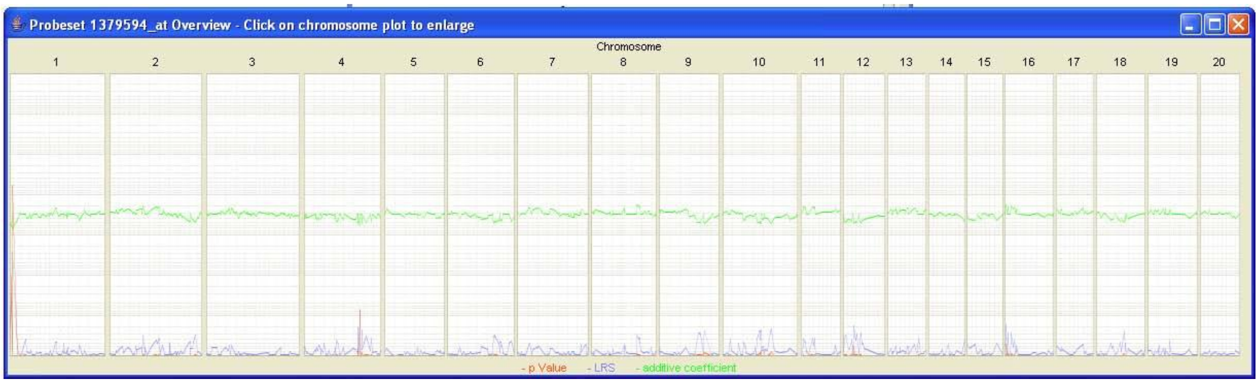

This shows the p-value, LRS, and additive coefficient across the whole genome. The linkage plot panel is subdivided into chromosomes and when you click a chromosome, the panel zooms in o the data for that chromosome.

8. Troubleshooting

1. I clicked the "Show Table" option and the table does not appear.

Please be patient. The software extracts all the information from the database and populates the table. The number of eQTLs it has to fetch governs the time it takes to populate the table.

2. I clicked the "Fetch" button and it the software does not appear to be doing anything.

It depends on your internet speed, the amount of data to be fetched from the database, and the number of people using this database at the same time. If it takes a lot of time, please close the application and re-run it.

3. I closed the zoomed image of the chromosome and when I maximize the main window of the software, the images do not display.

There is probably less graphics memory. Please click the **File** menu to refresh the main window.

4. I clicked the "Fetch" button and I do not see all the pQTLs or eQTLs.

The text field in the advanced options may not be clear; e.g., it still has a list of Affymetrix probeset ids. Similarly, some of the pQTLs may be selected in the previous Fetch. Click the **Reset** button to deselect the entire pQTLs list and click the **Fetch** button again.

9. Additional Information

Contact Us

Please contact jon.mangion@csc.mrc.ac.uk for any problems or suggestions.

System Requirements

Minimum system requirements are 256Mb RAM and the ability to run Java 1.4.2. The software has been tested on Windows XP, Linux 2.4+, and Mac OS X.

Licence

The eQTL Explorer software is distributed under the GNU Public License. It is freely available for non-commercial use.

Disclaimer

eQTL Explorer is distributed in the hope that it will be useful but WITHOUT ANY WARRANTY; without even the implied warranty of MERCHANTABILITY or FITNESS FOR A PARTICULAR PURPOSE. See the GNU General Public License for more details.

Index

A

Absolute Call Filter 50
Access Permissions 6
Accessing
  Arrays 131
Accessing Data and Tracking Data Use 135
Advanced Annotation
  Overview 73
  Performing 79
Analysis
  Filtering 50
  Promoter
    Overview 89
    Performing 90
    Results 115
  Statistical Analysis 53
Analysis 50
Annotation
  Advanced Annotation
    Overview 73
    Performing 79
  Basic Annotation
    Overview 73
    Performing 78
Annotation 73
Applying Filters 57
Approving
  Array Access 129
  Array Requests 129
  Registration Requests 130
Array Integrity Quality Control Check 31
Arrays
  Approve Array Requests 129
  Browsing
    Searching Arrays 10
  Grant Access 131
  Menu 10
  MIAMExpress
    Viewing Array Details 14
    Viewing Experiment Details 14
  Uploading MIAMExpress Data 16
Arrays 9

B

Basic Annotation
  Overview 73
  Performing 78
Browsing Arrays
  Browse All 26
  Granting Access to Arrays 129

C

Chips 14
Cloning and Mutating
  Gene Lists 73
Comparing Gene Lists 101
Copying
  Gene Lists 73
Creating
  Experiments
    Running Quality Control 42
  Experiments 26
  Gene Lists
    Enter Manually 71
    From Existing Lists 73
    From Experiment 68
    Uploading 102
  Gene Lists 68

D

Data Analysis
- Filtering 50
- Statistical Analysis 53

Data Analysis 50

Data Grouping and Normalization
- Creating Run Groups 48
- Normalizing Data 48

Data Grouping and Normalization 43

Defining QTLs 81

Deleting
- Gene Lists 106

Deleting QTL Lists 82

Details
- Gene Lists 66

Downloading
- Experiments 61
- Gene Lists 103

E

eQTL Explorer 86

Experiments
- Arrays
  - Approving Access to 129
  - Overview 9
  - Searching Arrays 10
- Creating
  - Running Quality Control 42
- Creating 26
- Data Analysis
  - Filtering 50
  - Statistical Analysis 53
- Data Analysis 50
- Downloading 61
- Gene Lists 61
- Grouping and Normalizing 48
- Menu 23
- Normalizing and Grouping 48
- Overview 21
- Process Flow 21
- Quality Control
  - Array Integrity 31
  - MIAMExpress Data Comparison 31
  - Running 42
  - Viewing Results 110
- Quality Control 31
- Statistical Analysis 53
- Viewing 24

Extraction
- Running Upstream Sequence Extraction 94
- Tools 144
- Upstream Sequence Extraction Overview 92

F

Feedback 137

Filtering
- Overview 50

Filtering 50

G

Gene Expression Data 60

Gene Lists
- Annotation 73, 78, 79
- Cloning and Mutating 73
- Comparing 101
- Creating
  - Enter Manually 71
  - From Existing Gene List 73
  - From Experiment 68
  - Uploading 102
- Creating 68
- Defining QTLs 81
- Deleting 106
- Downloading 103
- Gene List Details 66
- Literature Searches
  - Overview 98

Performing a Search 98
Viewing Results 124
Menu 64
Overview
Literature Searches 98
Promoter 89
Performing
Annotation 78, 79
Literature Searches 98
Performing Analysis 90
Upstream Sequence Extraction 94
Promoter
Analysis
Overview 89
Performing 90
Results 115
Extraction
Overview 92
Running 94
Tools 144
QTL
Defining 81
eQTL Explorer 86
QTL Query 83
Tools 80
Uploading 102
User Access 107
Viewing
Gene Lists 66
Gene Lists for Experiments 61
User Access to a Gene List 107
Gene Lists 63
Gene Lists Menu 100
Genetic Variation 96, 97
Getting Started
Logging Into the Website 8
Registration 6

H

Homolog 87
Homolog Search 87
Homologous genes 87

I

INIA Website
Logging In 8
Logging Out 8
Purpose 1
Registration 6
Security 135
Updating Your Information 7
INIA Website 2
Interpretation Results 100, 109

L

Literature Searches
Performing a Search 98
Viewing Results 124
Literature Searches 98
Logging Into the Website 8
Logging Out of the Website 8

M

Manually Enter a Gene List 71
MEME 92, 119
Menus
Arrays 10
Experiments 23
Gene Lists 64
Principal Investigator 128
Results 109
MIAMExpress
Data Comparison Quality Control Check 31
Uploading Arrays 16
Viewing
Array Details 14
Experiment Details 14
MIAMExpress 143

motif 92, 119
Mutating and Cloning
Gene Lists 73
N
Normalization and Data Grouping
Creating Run Groups 48
Normalizing Data 48
Normalization and Data Grouping 43
O
Overview
Annotation 73
Arrays 9
Data Analysis 50
Data Grouping and Normalization 43
Experiments 21
Gene Lists 63
INIA Website 2
Interpretation Results 109
Literature Searches 98
MIAMExpress 143
Principal Investigator 127
Promoter 89, 92
QTL 80
Quality Control 31
Security 135
P
Performing
Annotation 78, 79
Literature Searches 98
Promoter Analysis 90
Quality Control 42
Upstream Sequence Extraction 94
Performing 87
Permissions 6
Principal Investigator
Approve Registration Requests 130
Approving Array Requests 129
Granting Array Access 131
Menu 128
Principal Investigator 127
Promoter
Analysis
Overview 89
Performing 90
Viewing Results 115
Extraction
Overview 92
Running 94
Tools 144
Q
QTL
Tools 80
QTL 80
QTL
Defining QTLs 81
QTL
QTL Query 83
QTL
eQTL Explorer 86
Quality Control Checks
Additional Sources 141
All About R 142
Array Integrity 31
MIAMExpress Data Comparison 31
Running 42
Viewing Results 110
Quality Control Checks 31
R
R 142
Registration
Approve Request 130
Registration 6, 7
Reporting Problems 137
Results

- Literature Search 124
- Menu 109
- Promoter Analysis 115
- Quality Control 110

Results 109

Results 119

Results 119

Run Groups

- Creating 48

Run Groups 43

Running

- Quality Control Check 42
- Upstream Sequence Extraction 94

Running MEME 119

S

Samples 9, 14

Searching Arrays 10

Security

- Permissions 6

Security 135

SNPs 96, 97

Statistical Analysis

- Overview 53

Statistical Analysis 50

T

Tools 80

U

Updating Your Information 7

Uploading

- Arrays 16
- Gene Lists 102

Upstream Sequence Extraction 92, 119

User Access to Gene Lists

- Permissions 6
- Viewing 107

User Permissions 6

V

Versions 24

Viewing

- Experiments 24
- Gene Lists 66
- Gene Lists for Experiments 61
- Interpretation Results
  - Literature Search 124
  - Promoter Analysis 115
  - Quality Control 110
- Interpretation Results 109
- MIAMExpress
  - Array Details 14
  - Experiment Details 14
- User Access to Gene Lists 107

Viewing All QTL Lists 82

Notes
